# Efficacy and Safety of Vamorolone vs Placebo and Prednisone Among Boys With Duchenne Muscular Dystrophy: A Randomized Clinical Trial

**DOI:** 10.1001/jamaneurol.2022.2480

**Published:** 2022-08-29

**Authors:** Michela Guglieri, Paula R. Clemens, Seth J. Perlman, Edward C. Smith, Iain Horrocks, Richard S. Finkel, Jean K. Mah, Nicolas Deconinck, Nathalie Goemans, Jana Haberlova, Volker Straub, Laurel J. Mengle-Gaw, Benjamin D. Schwartz, Amy D. Harper, Perry B. Shieh, Liesbeth De Waele, Diana Castro, Michelle L. Yang, Monique M. Ryan, Craig M. McDonald, Mar Tulinius, Richard Webster, Hugh J. McMillan, Nancy L. Kuntz, Vashmi K. Rao, Giovanni Baranello, Stefan Spinty, Anne-Marie Childs, Annie M. Sbrocchi, Kathryn A. Selby, Migvis Monduy, Yoram Nevo, Juan J. Vilchez-Padilla, Andres Nascimento-Osorio, Erik H. Niks, Imelda J.M. de Groot, Marina Katsalouli, Meredith K. James, Johannes van den Anker, Jesse M. Damsker, Alexandra Ahmet, Leanne M. Ward, Mark Jaros, Phil Shale, Utkarsh J. Dang, Eric P. Hoffman

**Affiliations:** 1 John Walton Muscular Dystrophy Research Centre, Newcastle Hospitals NHS Foundation Trust and Newcastle University, Newcastle, United Kingdom; 2 University of Pittsburgh School of Medicine, Pittsburgh, Pennsylvania; 3 University of Washington School of Medicine, Seattle; 4 Duke University School of Medicine, Durham, North Carolina; 5 Royal Hospital for Children, Glasgow, United Kingdom; 6 Nemours Children’s Hospital, Orlando, Florida; 7 St Jude Children’s Research Hospital, Memphis, Tennessee; 8 Alberta Children’s Hospital Research Institute, University of Calgary, Calgary, Alberta, Canada; 9 Neuromuscular Reference Center, UZ Ghent, Ghent, Belgium; 10 Department of Development and Regeneration, KU Leuven, Leuven, Belgium; 11 Department of Paediatric Neurology, University Hospitals Leuven, Leuven, Belgium; 12 Neuromuscular Centre, Department of Pediatric Neurology, Motol University Hospital, 2nd Medical School, Charles University, Prague, Czech Republic; 13 The Camden Group, St Louis, Missouri; 14 Richmond Children’s Hospital, Richmond, Virginia; 15 UCLA Medical School, Los Angeles, California; 16 UT Southwestern Medical Center, Dallas, Texas; 17 University of Colorado School of Medicine, Children’s Hospital Colorado, Aurora; 18 The Royal Children’s Hospital, Melbourne, Australia; 19 Murdoch Children’s Research Institute, Melbourne, Australia; 20 University of California, Davis, Sacramento; 21 Queen Silvia Children’s Hospital, Gothenburg, Sweden; 22 Kids Neuroscience Centre, The Children’s Hospital at Westmead, Westmead, Australia; 23 University of Ottawa, Ottawa, Ontario, Canada; 24 Ann & Robert H. Lurie Children’s Hospital, Chicago, Illinois; 25 The Dubowitz Neuromuscular Centre, National Institute for Health Research Great Ormond Street Hospital Biomedical Research Centre, Great Ormond Street Institute of Child Health University College London, London, United Kingdom; 26 Alder Hey Children’s NHS Foundation Trust, Liverpool, United Kingdom; 27 Leeds Teaching Hospitals Trust, Leeds, United Kingdom; 28 Montreal Children’s Hospital, Montreal, Quebec, Canada; 29 BC Children’s Hospital Research Institute, Vancouver, British Columbia, Canada; 30 Nemours Children’s Hospital, Orlando, Florida; 31 Schneider Children’s Medical Center, Tel Aviv University, Tel Aviv, Israel; 32 Hospital Quirónsalud Valencia, Valencia, Spain; 33 Neuropaediatrics Department, Institut de Recerca Pediàtrica Hospital Sant Joan de Déu, Barcelona, Spain; 34 Leiden University Medical Center, Leiden, the Netherlands; 35 UMC St Radboud, Nijmegen, the Netherlands; 36 P&A Kyriakou Children’s Hospital, Athens, Greece; 37 ReveraGen BioPharma, Rockville, Maryland; 38 Children’s National Medical Center, Washington, DC; 39 ReveraGen BioPharma, Rockville, Maryland; 40 Children’s Hospital of Eastern Ontario Research Institute, Ottawa, Ontario, Canada; 41 Summit Analytical, Denver, Colorado; 42 Carleton University, Ottawa, Ontario, Canada; 43 Department of Pharmaceutical Sciences, School of Pharmacy and Pharmaceutical Sciences Binghamton University—State University of New York, Binghamton

## Abstract

**Importance:**

Corticosteroidal anti-inflammatory drugs are widely prescribed but long-term use shows adverse effects that detract from patient quality of life.

**Objective:**

To determine if vamorolone, a structurally unique dissociative steroidal anti-inflammatory drug, is able to retain efficacy while reducing safety concerns with use in Duchenne muscular dystrophy (DMD).

**Design, Setting, and Participants:**

Randomized, double-blind, placebo- and prednisone-controlled 24-week clinical trial, conducted from June 29, 2018, to February 24, 2021, with 24 weeks of follow-up. This was a multicenter study (33 referral centers in 11 countries) and included boys 4 to younger than 7 years of age with genetically confirmed DMD not previously treated with corticosteroids.

**Interventions:**

The study included 4 groups: placebo; prednisone, 0.75 mg/kg per day; vamorolone, 2 mg/kg per day; and vamorolone, 6 mg/kg per day.

**Main Outcomes and Measures:**

Study outcomes monitored (1) efficacy, which included motor outcomes (primary: time to stand from supine velocity in the vamorolone, 6 mg/kg per day, group vs placebo; secondary: time to stand from supine velocity [vamorolone, 2 mg/kg per day], 6-minute walk distance, time to run/walk 10 m [vamorolone, 2 and 6 mg/kg per day]; exploratory: NorthStar Ambulatory Assessment, time to climb 4 stairs) and (2) safety, which included growth, bone biomarkers, and a corticotropin (ACTH)–challenge test.

**Results:**

Among the 133 boys with DMD enrolled in the study (mean [SD] age, 5.4 [0.9] years), 121 were randomly assigned to treatment groups, and 114 completed the 24-week treatment period. The trial met the primary end point for change from baseline to week 24 time to stand velocity for vamorolone, 6 mg/kg per day (least-squares mean [SE] velocity, 0.05 [0.01] m/s vs placebo −0.01 [0.01] m/s; 95% CI, 0.02-0.10; *P* = .002) and the first 4 sequential secondary end points: time to stand velocity, vamorolone, 2 mg/kg per day, vs placebo; 6-minute walk test, vamorolone, 6 mg/kg per day, vs placebo; 6-minute walk test, vamorolone, 2 mg/kg per day, vs placebo; and time to run/walk 10 m velocity, vamorolone, 6 mg/kg per day, vs placebo. Height percentile declined in prednisone-treated (not vamorolone-treated) participants (change from baseline [SD]: prednisone, −1.88 [8.81] percentile vs vamorolone, 6 mg/kg per day, +3.86 [6.16] percentile; *P* = .02). Bone turnover markers declined with prednisone but not with vamorolone. Boys with DMD at baseline showed low ACTH-stimulated cortisol and high incidence of adrenal insufficiency. All 3 treatment groups led to increased adrenal insufficiency.

**Conclusions and Relevance:**

In this pivotal randomized clinical trial, vamorolone was shown to be effective and safe in the treatment of boys with DMD over a 24-week treatment period. Vamorolone may be a safer alternative than prednisone in this disease, in which long-term corticosteroid use is the standard of care.

**Trial Registration:**

ClinicalTrials.gov Identifier: NCT03439670

## Key Points

**Question:**

For steroidal anti-inflammatory drugs, can efficacy be retained while safety concerns are reduced among boys with Duchenne muscular dystrophy (DMD) with the novel partial receptor agonist vamorolone?

**Findings:**

A randomized, double-blind, placebo- and prednisone-controlled trial of vamorolone (2 dose groups) was carried out in 121 patients with DMD. The trial met the primary (time to stand velocity after 24 weeks for vamorolone, 6 mg/kg per day vs placebo) and first 4 sequential secondary motor function end points; vamorolone showed loss of bone morbidities compared with prednisone, with no stunting of growth and no deleterious changes in bone biomarkers.

**Meaning:**

This study found that vamorolone, a dissociative steroidal anti-inflammatory, was able to reduce bone morbidities while retaining efficacy.

## Teaser

This randomized clinical trial investigates the efficacy and safety of partial receptor agonist vamorolone compared with placebo and prednisone in boys with Duchenne muscular dystrophy.

## Introduction

Duchenne muscular dystrophy (DMD) is an X-linked recessive neuromuscular disorder affecting 1 in 3600 to 9300 male newborns.^1^ Treatment with oral corticosteroids (prednisone, deflazacort) delays loss of ambulation,^2^ but long-term corticosteroid treatment causes weight gain, stunting of growth, osteoporosis, mood disturbances, adrenal insufficiency, and other safety concerns leading to poor adherence to practice guidelines.^3,4^

Vamorolone is a first-in-class dissociative steroidal anti-inflammatory drug that binds to the same target receptors as the corticosteroid class (glucocorticoid receptor, mineralocorticoid receptor), but shows a distinct chemical structure and differences in mechanism of action. Vamorolone shows less positive gene transcriptional activity (transactivation) than corticosteroids but retains inhibition of nuclear factor κB proinflammatory pathways (transrepression). Vamorolone uniquely lacks a 11β-hydroxyl/carbonyl moiety on the steroidal C ring, changing structure and activity relationships with the receptors.^5^ Further, vamorolone cannot be acted on by modulatory 11β-hydroxysteroid dehydrogenase enzymes known to be necessary for mediating corticosteroid-associated bone morbidities in mice.^6^ Lastly, vamorolone is a potent antagonist of the mineralocorticoid receptor, whereas most corticosteroids are agonists.^7^

First-in-patient, open-label, dose-ranging studies of vamorolone in DMD (n = 48) suggested improvements in motor outcomes similar to corticosteroids, without stunting of growth over a 2.5-year treatment period, compared with external corticosteroid-treated comparators.^8,9,10,11^ In the study reported here, we present results of a pivotal 24-week double-blind, placebo- and prednisone- controlled clinical efficacy and safety trial of vamorolone in boys 4 to younger than 7 years of age with DMD who were not previously treated with corticosteroids.

## Methods

### Participants

Boys 4 to younger than 7 years of age with DMD were enrolled at 33 academic medical sites in 11 countries. Inclusion criteria included a *DMD* gene loss-of-function variation or lack of muscle dystrophin. Race and ethnicity data were gathered using National Institutes of Health guidelines, as required by federal funding for this study. Race data were gathered from query of parents of the children for the following groups: American Indian/Alaska Native, Asian, Black or African American, Native Hawaiian or Other Pacific Islander, White or Caucasian, Unknown, and Multiple. Ethnicity data gathered were Hispanic or Latino, and Not Hispanic or Latino. Participants were not previously treated with corticosteroids and were able to perform time to stand from supine in less than 10 seconds. Full eligibility criteria are provided in the study protocol (Supplements 1, 2, 3, 4, 5, and 6). The health care proxy for each participant provided written informed consent. The trial, conducted from June 29, 2018, to February 24, 2021, was approved by the competent ethics committee at each institution and was conducted in accordance with the International Conference on Harmonisation guidelines for Good Clinical Practice and the World Medical Association Declaration of Helsinki. This study followed the Consolidated Standards of Reporting Trials (CONSORT) reporting guidelines.

### Trial Design and Treatment

Sample sizes were determined based on published prednisone-treatment efficacy from a Cooperative International Neuromuscular Research Group prednisone trial in the same age range and then reanalyzed from analysis of vamorolone open-label trial data.^9^ The trial was designed for efficacy as placebo-controlled, as requested by US Food and Drug Administration guidance. The trial included two, 24-week treatment periods. For treatment period 1, participants were randomly assigned to the placebo, prednisone (0.75 mg/kg per day), vamorolone (2 mg/kg per day), and vamorolone (6 mg/kg per day) groups in a 1:1:1:1 ratio. In treatment period 2, participants in the placebo and prednisone groups crossed over to receive vamorolone treatment (2 or 6 mg/kg per day). We report results of treatment period 1 (the statistical analysis plan [SAP] was submitted to the Investigational New Drug file before treatment period 1 unblinding and is included in Supplement 7).

### Randomization and Blinding

Randomization was done using an Interactive Voice/Web Response System held by the central pharmacy (Almac). As DMD is a progressive disease, we sought to keep age range distribution similar between treatment groups and included randomization by age group within the 4 to younger than 7-year age range (<6 vs ≥6 years). Vamorolone was supplied as a flavored suspension (1.3% for 2 mg/kg per day; 4.0% for 6 mg/kg per day), and volumes were matched for blinding. Prednisone and placebo were supplied as tablets (5-mg tablet). All participants took both tablets and a suspension each morning to maintain the study blinding.

### Trial Procedures and Outcomes

Efficacy motor outcomes were time to stand from supine velocity (TTSTAND), 6-minute walk test (6MWT), time to run/walk 10 m (TTRW), time to climb 4 stairs (TTCLIMB), and NorthStar Ambulatory Assessment (NSAA) total score.^12^ Strength outcomes were handheld myometry (elbow flexors, knee extensors). Parent-reported outcomes were Pediatric Outcomes Data Collection Instrument (PODCI), Psychosocial Adjustment and Role Skills Scale III (PARS III), and Treatment Satisfaction Questionnaire (TSQM). Motor assessments by trained clinical evaluators were done at screening, baseline, 12 weeks, and 24 weeks.

Safety end points (clinical and laboratory) were assessed at screening, baseline, and weeks 2, 6, 12, 18, and 24. A standard-dose corticotropin (ACTH) stimulation test measuring cortisol at baseline and 30 and 60 minutes after tetracosactide (Synacthen), 250 μg, diagnostic testing was done at screening and week 24.

Pharmacodynamic safety biomarkers (bone turnover and morning cortisol) were done at baseline and weeks 12 and 24. Dual-energy x-ray absorptiometry (for lumbar spine and total body bone mineral density and content and total body composition) and lateral spine radiography (for vertebral fractures from T4 to L4 according to the modified Genant semiquantitative method) were done at screening and week 24, with results analyzed centrally. The full protocol is provided in Supplements 1, 2, 3, 4, 5, and 6, and 24-week SAP in Supplement 7.

The primary efficacy end point was mean change from baseline to week 24 for TTSTAND velocity for vamorolone, 6 mg/kg per day, vs placebo (mixed model for repeated measures [MMRM]). The ranked (hierarchical) secondary outcomes were mean change from baseline to week 24 for TTSTAND velocity for vamorolone, 2 mg/kg per day, vs placebo; 6MWT for vamorolone, 6 mg/kg per day, vs placebo; 6MWT for vamorolone, 2 mg/kg per day, vs placebo; TTRW velocity for vamorolone, 6 mg/kg per day, vs placebo; TTRW velocity for vamorolone, 2 mg/kg per day, vs placebo; 6MWT for vamorolone, 6 mg/kg per day, vs prednisone, 0.75 mg/kg per day; and 6MWT for vamorolone, 2 mg/kg per day, vs prednisone, 0.75 mg/kg per day.

The COVID-19 pandemic necessitated protocol modifications that included remote assessment of efficacy and safety. When participants were unable to attend scheduled on-site visits owing to COVID-19 pandemic–related limitations, safety visits were done by telephone or video conference and remote safety laboratory collection. Remote efficacy assessments were limited to the primary outcome (TTSTAND) and undertaken with the clinical evaluator instructing and observing the test by videoconference, while a parent or caregiver recorded the test for upload to a secure website (ChiliPharm) for evaluator timing of test. Secondary and exploratory efficacy and safety outcomes were not assessed remotely (missing data).

### Statistical Analysis

SAS, release 9.4 (SAS Institute), for Windows was used for analyses with both SAS and R statistical software, version 4.1.2 (R Foundation), used for figures. In accordance with the SAP, all measurements were analyzed based on the type of distribution, and descriptive statistics were presented by treatment group and assessment time point, as appropriate. No formal interim statistical analyses were done, apart from the interim data reviews and presentations created for the data safety monitoring board. Analyses were summarized for the 4 treatment groups: vamorolone, 2 mg/kg per day; vamorolone, 6 mg/kg per day; prednisone, 0.75 mg/kg per day; and placebo. For functional outcome efficacy analyses, a fixed sequential testing approach was used, where each test in the prespecified sequence was conducted using a 2-sided α level of .05.

Efficacy outcomes were tested via a restricted maximum likelihood–based MMRM. This model included fixed effects for treatment, week, baseline outcome, age group (per randomization stratification), and the treatment-by-week interaction. Study week was included in the model as a categorical variable along with the treatment-by-week interaction. Within this model, comparisons of outcomes (using least-squares mean [LSM] contrasts) were made at 24 weeks for the vamorolone vs the placebo groups as prespecified (both primary and secondary outcomes). Comparisons of relative drug effect using percentage change from baseline was done as a post hoc analysis with the same MMRM setup. All *P* values were 2 sided, and *P* < .05 was considered significant.

## Results

### Patients

**Figure 1.**
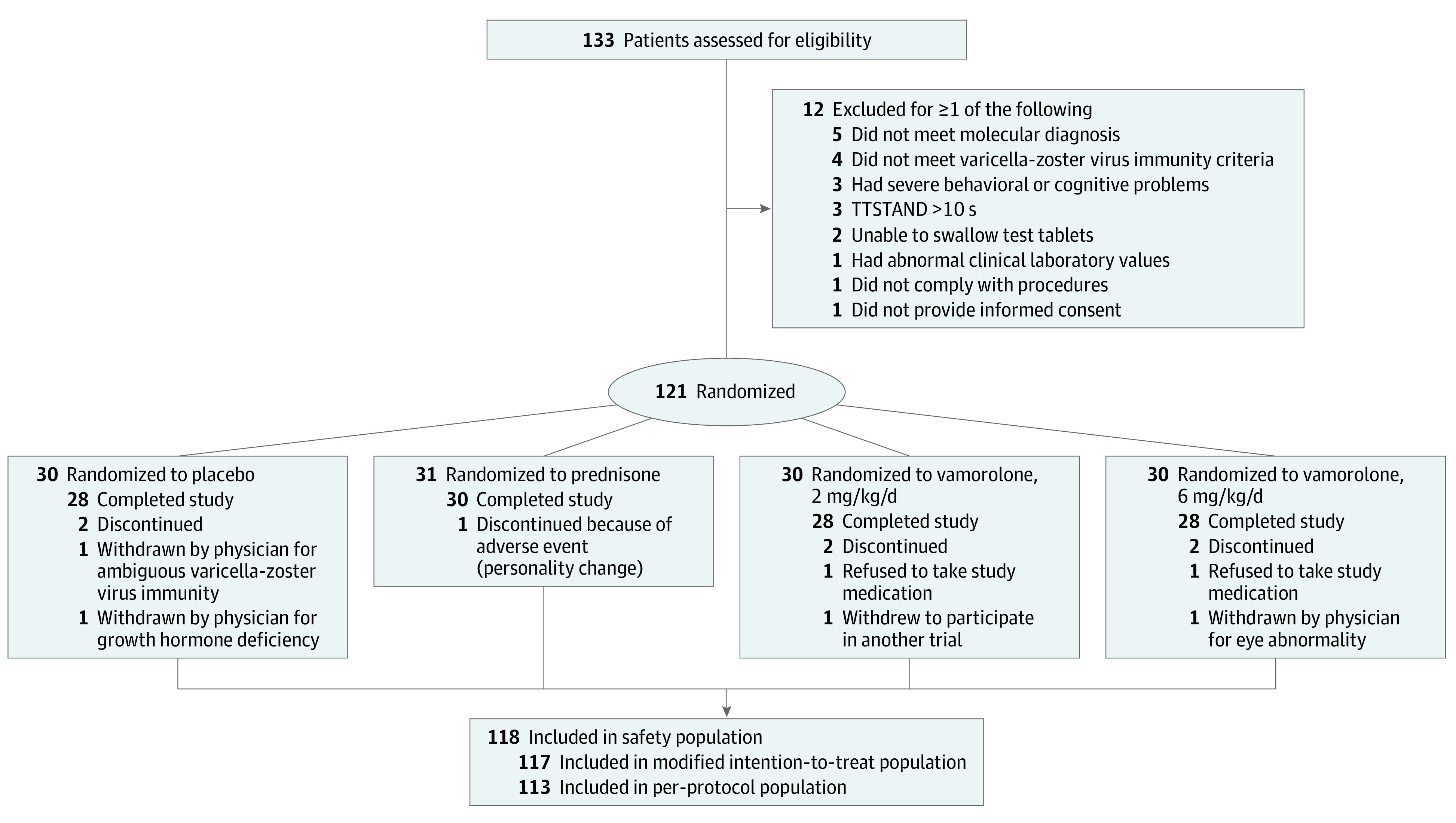
Study Participant Flowchart

A total of 133 boys with DMD (mean [SD] age, 5.4 [0.9] years) were screened; 121 were randomly assigned to 1 of the 4 treatment groups, and 114 participants completed the study (Figure 1, Table 1, and eTable 1 in Supplement 8). The first participant was enrolled on June 29, 2018, and the last patient’s final visit for period 1 (24-week treatment) was February 24, 2021. Race demographics included the following groups: 1 American Indian/Alaska Native (0.9%), 12 Asian (10.3%), 2 Black or African American (1.7%), 97 White or Caucasian (82.9%), 1 unknown (0.9%), and 4 multiple (3.4%). Ethnicity data gathered included the following groups: 5 Hispanic or Latino (4.3%) and 112 not Hispanic or Latino (95.7%). No participant withdrew owing to COVID-19 pandemic–related issues. For the primary outcome 24-week assessment, there was 1 assessment missing owing to the COVID-19 pandemic, with 9.6% of the 24-week TTSTAND assessments (11 of 114) done remotely. The 4 additional motor outcomes (6MWT, TTRW, TTCLIMB, NSAA) were not done remotely. Missing data percentages for secondary outcomes owing to COVID-19 remote assessments were 14.5% (17 of 117) for 6MWT and 12.0% (14 of 117) for TTRW. Baseline characteristics were balanced between the 4 groups, inclusive of pharmacodynamic safety biomarkers, with the exception of baseline motor function, which appeared to be better in the prednisone group vs the vamorolone groups (Table 1).

**Table 1.** Characteristics of the Participants at Baseline (Modified Intention-to-Treat Population)

Characteristic	Mean (SD)			
	Vamorolone		Prednisone 0.75 mg/kg/d (n = 31)	Control (placebo) group (n = 28)
	6 mg/kg/d (n = 28)	2 mg/kg/d (n = 30)		
**Quality rating scheme = 1**				
Age, y	5.4 (0.9)	5.3 (0.9)	5.5 (0.9)	5.4 (0.8)
Height, cm	107 (7)	108 (9)	111 (6)	109 (9)
Height percentile	23 (25)	30 (29)	37 (29)	33 (29)
Weight, kg	19 (3)	19 (4)	21 (3)	20 (3)
BMI^a^	16.6 (1.4)	16.2 (1.2)	16.8 (1.3)	16.3 (1.1)
TTSTAND velocity, event/s	0.19 (0.06)	0.18 (0.05)	0.22 (0.06)	0.20 (0.06)
6MWT, m	313 (56)	316 (58)	343 (56)	355 (78)
TTRW velocity, m/s	1.6 (0.4)	1.6 (0.3)	1.9 (0.4)	1.7 (0.3)
NSAA score	18.9 (4.1)	17.2 (4.7)	21.2 (5.5)	18.9 (5.3)
TTCLIMB velocity, event/s	0.21 (0.09)	0.20 (0.05)	0.29 (0.11)	0.25 (0.09)
Osteocalcin level, ng/mL^b^	59.7 (14.8)	57.2 (18.3)	55.9 (12.9)	55.0 (13.8)
P1NP level, μg/L	490 (145)	521 (204)	480 (116)	483 (161)
CTX1 level, pg/mL^c^	1074 (206)	1128 (382)	1125 (162)	1079 (258)
Morning cortisol level, nmol/L^d^	235 (67)	238 (83)	212 (66)	199 (62)
**Standard dose ACTH stimulation test**				
Serum cortisol level, nmol/L				
30 min (Normal range >500)	547 (119)	555 (86)	532 (101)	550 (104)
60 min (Normal range >500)	659 (105)	648 (94)	612 (97)	628 (112)
<500 nmol/L at 30 and 60 min, No. (%)	0 (0)	2 (7.4)	5 (17.2)	3 (10.7)

Abbreviations: 6MWT, 6-minute walk test; ACTH, corticotropin; BMI, body mass index; CTX1, type 1 collagen cross-linked C-telopeptide; NSAA, NorthStar Ambulatory Assessment; P1NP, procollagen 1 intact N-terminal propeptide; TTCLIMB, time to climb 4 stairs; TTRW, time to run/walk 10 m; TTSTAND, time to stand from supine.

SI conversion factor: To convert osteocalcin to micrograms per liter, multiply by 1; to convert serum cortisol to micrograms per deciliter, divide by 27.588.

^a^ Calculated as weight in kilograms divided by height in meters squared.

^b^ Normal range, 39-121 ng/mL.

^c^ Normal range, 500-1700 pg/mL.

^d^ Normal range, 138-690 nmol/L.

### Efficacy

#### Primary End Point

**Figure 2.**
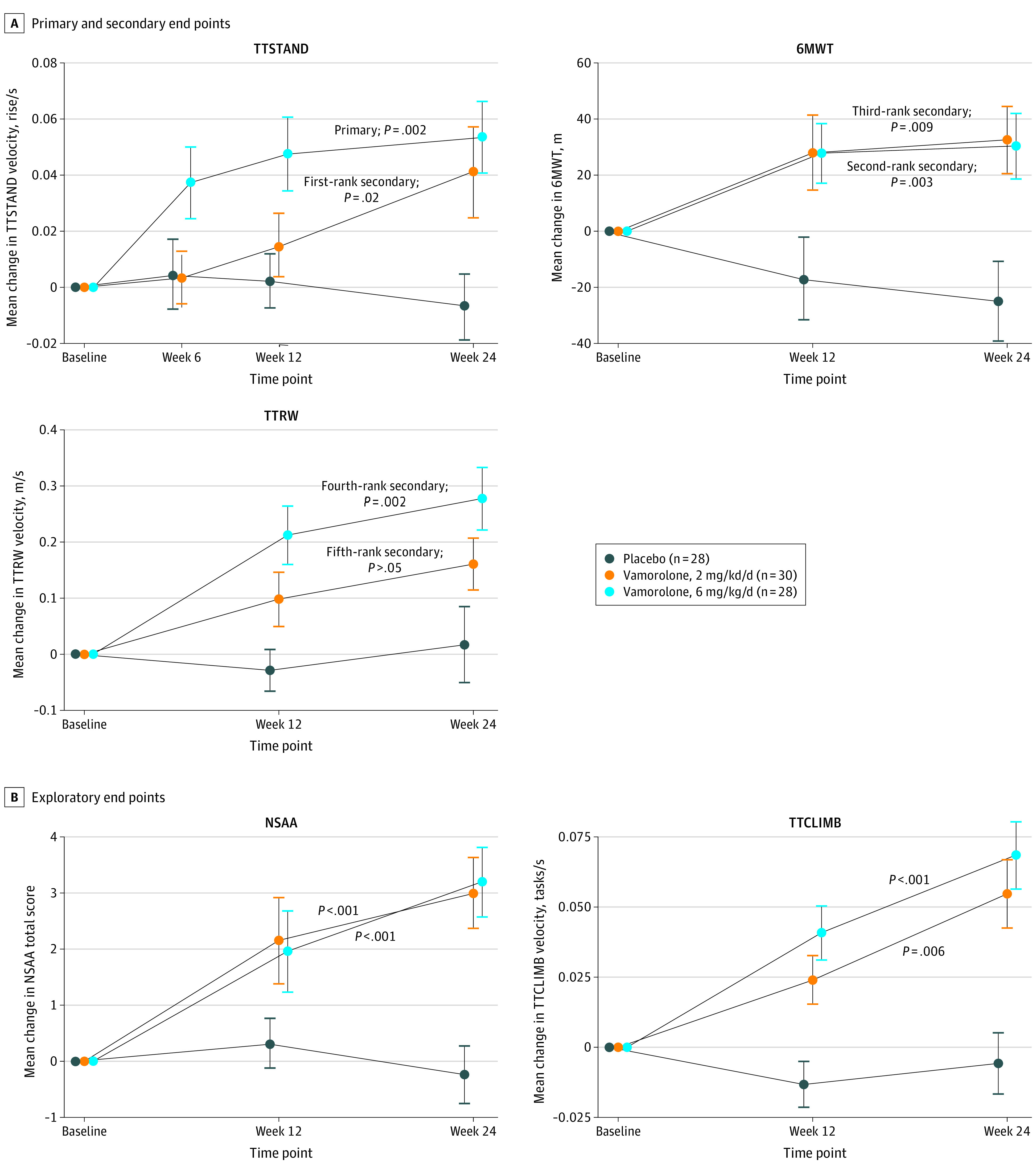
Motor End Points Over the 24-Week Treatment Period A, Shown are motor outcomes and treatment effect in the placebo, vamorolone, 6 mg/kg per day, and vamorolone, 2 mg/kg per day, groups. Sequential (hierarchical) secondary end points were prespecified in the statistical analysis plan as discussed with the US Food and Drug Administration in the indicated order as shown (first secondary end point through fifth secondary end point). The trial met the conditions of *P* < .05 for the first 4 sequential secondary end points but failed on fifth secondary end point, and further formal testing of secondary end points was halted. B, Time to climb 4 stairs (TTCLIMB) and NorthStar Ambulatory Assessment (NSAA) were exploratory end points. Error bars represent ±SE. 6MWT indicates 6-minute walk test; TTRW, time to run/walk 10 m; TTSTAND, time to stand from supine.

All end points were prespecified in the study protocol and SAP (Supplements 1, 2, 3, 4, 5, 6, and 7). The primary end point of change from baseline to week 24 for TTSTAND velocity for vamorolone, 6 mg/kg per day, vs placebo was met (LSM [SE] velocity, 0.05 [0.01] m/s vs −0.01 [0.01] m/s; LSM difference, 0.06 m/s; 95% CI, 0.02-0.10 m/s; *P* = .002) (modified intention-to-treat [mITT] population) (Figure 2, Table 2). The placebo group showed a stable course with a slight decline relative to baseline, whereas the vamorolone, 6 mg/kg per day, group vs placebo showed improvement by 6 weeks of treatment (LSM [SE] velocity, 0.03 [0.01] m/s vs 0 [0.01] m/s; LSM difference, 0.03 m/s; 95% CI, 0.01-0.06 m/s; *P* = .02), continued improvement to 12 weeks of treatment (LSM [SE] velocity, 0.04 [0.01] m/s vs −0.01 [0.01] m/s; LSM difference, 0.02 m/s; 95% CI, 0.02-0.08 m/s; *P* = .001), and maintained to 24 weeks of treatment. Analyses of the mITT population (n = 117) vs per protocol population (n = 113) led to similar findings.

**Table 2.** Primary and Secondary Efficacy End Points vs Placebo and Safety End Points vs Prednisone^a^

End point	Vamorolone						Placebo group, change from baseline, mean (SD) [No.]	Prednisone group, change from baseline, mean (SD) [No.]
	6 mg/kg/d group			2 mg/kg/d group				
	Change from baseline, mean (SD) [No.]	End point rank LSM difference (95% CI)	*P* value	Change from baseline, mean (SD) [No.]	End point rank LSM difference (95% CI)	*P* value		
**Efficacy vs placebo** ^b^								
TTSTAND velocity, rise/s	0.05 (0.07) [27]	Primary: 0.06 (0.02 to 0.10)	.002	0.04 (0.09) [29]	First-rank secondary: 0.05 (0.01 to 0.08)	.02	−0.01 (0.06) [28]	NA
6MWT, m	28.8 (49.7) [20]	Second-rank secondary: 41.6 (14.2 to 68.9)	.003	31.0 (51.1) [20]	Third-rank secondary: 37.1 (9.6 to 64.7)	.009	−23.9 (59.6) [19]	
TTRW velocity, m/s	0.28 (0.28) [25]	Fourth-rank secondary: 0.24 (0.09 to 0.39)	.002	0.16 (0.23) [24]	Fifth-rank secondary: 0.13 (−0.03 to 0.28)	>.05	0.02 (0.33) [24]	
**Safety vs prednisone** ^c^								
Height percentile	3.86 (6.16) [26]	4.98 (0.75 to 9.21)	.02	0.26 (9.22) [27]	1.86 (−2.27 to 6.00)	>.05	NA	−1.88 (8.81) [30]
BMI *z* score	0.52 (0.62) [27]	0.09 (−0.19 to 0.36)	>.05	0.40 (0.45) [27]	−0.06 (−0.34 to 0.22)	>.05		0.41 (0.51) [30]

Abbreviations: BMI, body mass index; LSM, least-squares mean; MMRM, mixed model for repeated measures; NA, not applicable; TTRW, time to run/walk 10 m; TTSTAND, time to stand from supine.

^a^ Week 24 changes from baseline are shown. *P* values are from MMRM using all assessment time points. LSM difference is vamorolone groups vs placebo group (efficacy) or vs prednisone (safety).

^b^ Modified intention-to-treat population.

^c^ Safety population.

#### Secondary End Points

The first-rank secondary end point was change from baseline to week 24 for TTSTAND velocity for vamorolone, 2 mg/kg per day, vs placebo, was met (LSM [SE] velocity, 0.03 [0.01] m/s vs −0.01 [0.01] m/s; LSM difference, 0.05 m/s; 95% CI, 0.01-0.08 m/s; *P* = .02) (Figure 2, Table 2). Vamorolone, 2 mg/kg per day, showed a larger latency to peak rise compared with vamorolone, 6 mg/kg per day. The subsequent 2 secondary end points were also met, change from baseline to week 24 for 6MWT for vamorolone, 6 mg/kg per day, vs placebo (second-rank secondary end point in Table 2, Figure 2) (LSM [SE] distance, 28.3 [9.6] m vs −13.3 [10.0] m; LSM difference, 41.6 m; 95% CI, 14.2-68.9 m; *P* = .003), and vamorolone, 2 mg/kg per day, vs placebo (third-rank secondary end point in Table 2, Figure 2) (LSM [SE] distance, 23.9 [9.7] m vs −13.3 [10.0] m; LSM difference, 37.1 m; 95% CI, 9.6-64.7 m; *P* = .009) (Figure 2, Table 2). The next secondary end point, change from baseline to week 24 for TTRW velocity, was met for vamorolone, 6 mg/kg per day, vs placebo (fourth-rank secondary end point in Table 2, Figure 2) (LSM [SE] velocity, 0.26 [0.05] m/s vs 0.01 [0.06] m/s; LSM difference, 0.24 m/s; 95% CI, 0.09-0.39 m/s; *P* = .002). The fifth secondary end point was not met for TTRW velocity vamorolone, 2 mg/kg per day, vs placebo (fifth secondary end point in Table 2, Figure 2), ending hierarchical testing.

#### Exploratory End Points

NSAA total score and TTCLIMB velocity were exploratory end points. Both end points showed improvement in favor of vamorolone, in both the 2 and 6 mg/kg per day vs placebo groups (NSAA total score: vamorolone, 6 mg/kg per day LSM [SE], 2.85 [0.61] vs −0.73 [0.62]; LSM difference, 3.57; 95% CI, 1.90-5.25; *P* < .001; vamorolone, 2 mg/kg per day LSM [SE], 2.52 [0.63] vs −0.73 [0.62]; LSM difference, 3.25; 95% CI, 1.53-4.97; *P* < .001) (TTCLIMB velocity: vamorolone, 6 mg/kg per day LSM [SE], 0.06 [0.02] vs −0.01 [0.02]; LSM difference, 0.07; 95% CI, 0.03-0.11; *P* < .001; vamorolone, 2 mg/kg per day LSM [SE], 0.05 [0.02] vs 0.11 [0.02]; LSM difference, 0.06; 95% CI, 0.02-0.10; *P* = .006) (Figure 2).

Parent-reported outcomes (PODCI, TSQM) and measures of muscle strength (handheld myometry) showed no significant differences between vamorolone and placebo groups. Prespecified analysis of PARS III was limited to 4 of 5 subscales (peer relations, dependency, anxiety and depression, withdrawal), using MMRM, and suggested that vamorolone, 2 mg/kg per day, showed better adjustment for anxiety and depression compared with prednisone (eTable 2 in Supplement 8); however, this was not adjusted for multiple testing (24 tests done).

Relative efficacy of prednisone and vamorolone, 6 mg/kg per day, were similar for all 5 motor outcomes (eFigure in Supplement 8). Vamorolone, 2 mg/kg per day, showed similar effectiveness as prednisone for TTSTAND, 6MWT, and NSAA but less effectiveness for TTRW and TTCLIMB.

#### Clinical Safety End Points

The number of participants reporting at least 1 treatment-emergent adverse event (TEAE) was similar between groups (placebo group, 79.3% [23 of 29]; prednisone group, 83.9% [26 of 31]; vamorolone, 2 mg/kg per day group, 83.3% [25 of 30]; vamorolone, 6 mg/kg per day group, 89.3% [25 of 28]) (eTable 3 in Supplement 8). The total count of TEAEs was lowest in the placebo group (n = 77), highest in the prednisone group (n = 121), and intermediate in the 2 vamorolone groups (2 mg/kg per day, n = 97; 6.0 mg/kg per day, n = 91). A single participant receiving prednisone, 0.75 mg/kg per day, withdrew from the study owing to an AE (personality change, Common Terminology Criteria for AEs [CTCAE] grade 2) that was viewed by the investigator (I.H.) as possibly related to the drug and abated after cessation of the drug. There was a single TEAE in the study considered by the investigator to be severe (aggression, CTCAE grade 3) experienced by a participant receiving prednisone, 0.75 mg/kg per day; the participant remained in the study. One participant in the vamorolone, 2 mg/kg per day, group experienced a serious AE of viral gastroenteritis, viewed as not related to the study drug.

Height percentile declined in prednisone-treated, but not vamorolone-treated, participants (change from baseline [SD]: prednisone −1.88 [8.81] percentile vs vamorolone, 6 mg/kg per day, +3.86 [6.16] percentile; *P* = .02). There was linear growth delay in the prednisone group but not in the vamorolone groups (vamorolone, 6 mg/kg per day, vs prednisone; LSM difference, 4.98; 95% CI, 0.75-9.21; *P* = .02) (Table 2), consistent with 2.5-year open-label data.^10,11^ The vamorolone and prednisone groups showed similar overall gain in body mass index (increase of 0.4-0.5 body mass index *z* score over the 24-week treatment period), with high intragroup variability (Table 2).

Two participants had 3 prevalent vertebral fractures at baseline. There were 2 treatment-emergent vertebral fractures at week 24; 1 participant in the prednisone group had a total of 4 incident vertebral fractures, and 1 participant in the placebo group had a single incident vertebral fracture. All vertebral fractures observed in this trial were mild (Genant grade 1) and in the thoracic region. There were no incident long-bone fractures reported. For dual-energy x-ray absorptiometry, only total body lean mass index (calculated as weight in kilograms divided by height in meters squared) for the prednisone group (n = 24) vs the vamorolone, 2 mg/kg per day, group (n = 18) of 18 comparisons showed significance that survived post hoc adjustment for multiple testing (LSM [SE], vamorolone, 2.61 [1.42] vs prednisone 9.62 [1.29]; unadjusted *P* < .001; Bonferroni-Holm adjusted *P* = .007) in favor of prednisone.

#### Biomarker Safety End Points

Serum biomarkers of bone formation (osteocalcin, procollagen 1 intact N-terminal propeptide [P1NP]) and bone turnover (type 1 collagen cross-linked C-telopeptide [CTX1]) showed marked reductions with prednisone treatment but not vamorolone treatment (mean [SD] osteocalcin: prednisone vs vamorolone, 6 mg/kg per day, −15.5 [15.8] ng/mL vs −0.17 [17.7] ng/mL; mean [SD] P1NP: prednisone vs vamorolone, 6 mg/kg per day, −143.7 [124.6] ng/mL vs −7.9 [122.1] ng/mL; mean [SD] CTX1: prednisone vs vamorolone, 6 mg/kg per day, −320 [174] pg/mL vs 110 [267] pg/mL; all comparisons *P* < .001; to convert osteocalcin to micrograms per liter, multiply by 1) (Table 3; eTable 4 in Supplement 8).

**Table 3.** Secondary Biomarker Safety End Points (Safety Population)

End point	Vamorolone						Prednisone group, change from baseline, mean (SD) [No.]	Placebo group
	6 mg/kg/d group			2 mg/kg/d group				
	Change from baseline, mean (SD) [No.]	LSM difference (95% CI)	*P* value^a^	Change from baseline, mean (SD) [No.]	LSM difference (95% CI)	*P* value^a^		
Osteocalcin level,^b^ ng/mL	−0.17 (17.7) [22]	17.1 (9.3 to 24.9)	<.001	8.7 (17.6) [18]	23.8 (15.5 to 32.1)	<.001	−15.5 (15.8) [23]	NA
P1NP level, ng/mL	−7.9 (122.1) [23]	128.8 (67.2 to 190.4)	<.001	77.2 (151.3) [16]	188.6 (120.7 to 256.4)	<.001	−143.7 (124.6) [23]	
CTX1 level, pg/mL	110 (267) [23]	394 (272 to 516)	<.001	189 (290) [17]	481 (349 to 614)	<.001	−320 (174) [24]	
Morning cortisol level, nmol/L	−195 (84) [26]	−36 (−68 to −4)	.03^c^	−99 (84) [21]	59 (25 to 93)	<.001^c^	−143 (80) [25]	
**Standard dose ACTH stimulation test**								
Serum cortisol level <500 nmol/L, No./total No. (%)	20/21 (95)			18/21 (86)			26/26 (100)	4/20 (20)

Abbreviations: ACTH, corticotropin; CTX1, type 1 collagen cross-linked C-telopeptide; LSM, least-squares mean; MMRM, mixed model for repeated measures; NA, not applicable; P1NP, procollagen 1 intact N-terminal propeptide.

SI conversion factor: To convert osteocalcin to micrograms per liter, multiply by 1; to convert serum cortisol to micrograms per deciliter, divide by 27.588.

^a^ MMRM of vamorolone groups vs prednisone group.

^b^ Bone biomarkers (osteocalcin, P1NP, CTX1) and morning cortisol level are MMRM vamorolone dose group vs prednisone group. ACTH challenge is percentage of participants at 24 weeks with both 30-minute and 60-minute cortisol levels less than 500 nmol/L.

^c^ Fisher exact test (2 tailed) of vamorolone groups vs prednisone group.

Participants showed evidence of adrenal insufficiency at baseline, with approximately 10% of morning cortisol and 20% of ACTH-stimulated measures flagged as “LOW” (eTable 5 in Supplement 8). All drug treatment groups showed significant reductions of both morning cortisol at both 12-week and 24-week assessments, and ACTH-stimulation tests (eTable 6 in Supplement 8). By morning cortisol, the vamorolone, 2 mg/kg per day, group showed less adrenal suppression than prednisone (mean [SD] change from baseline, −99 [84] nmol/L vs −143 [80] nmol/L; *P* < .001; to convert serum cortisol to micrograms per deciliter, divide by 27.588), whereas vamorolone, 6 mg/kg per day, showed greater adrenal suppression than prednisone (mean [SD] change from baseline, −195 [84] nmol/L vs −143 [80] nmol/L; *P* = .03) (Table 3).

## Discussion

In this double-blind randomized clinical trial, boys with DMD receiving vamorolone, 2 mg/kg per day, and vamorolone, 6 mg/kg per day, showed improvements in multiple functional end points over the 24-week treatment period compared with placebo (Figure 2). The statistical thresholds for the primary outcome and first 4 secondary outcomes for vamorolone treatment were met, and vamorolone demonstrated efficacy across a 3-fold dose range (2 mg/kg per day to 6 mg/kg per day). The differences in TTSTAND velocity (0.06 rises per second for vamorolone, 6 mg/kg per day, vs placebo and 0.05 rises per second for vamorolone, 2 mg/kg per day, vs placebo) were clinically meaningful (>0.02 rises per second).^13^ The differences in 6MWT (42 m for vamorolone, 6 mg/kg per day, vs placebo and 37 m for vamorolone, 2 mg/kg per day, vs placebo) were also clinically meaningful (>30 m).^14^

This trial also validated previous open-label findings of normal growth trajectories over an 18-month period^10^ and 30-month period^11^ in vamorolone-treated boys with DMD. In contrast, prednisone treatment slowed growth trajectories in this 24-week trial, confirming multiple studies of corticosteroid treatment in DMD.^3,4,11^ Furthermore, bone turnover markers support the improved safety profile of vamorolone on bone health, as none showed mean declines in either vamorolone dose group (Table 3). Of note, the 11β-hydroxysteroid dehydrogenase enzymes have been found to be necessary for corticosteroid-induced bone morbidities in mice; vamorolone is not a substrate for these enzymes as it lacks the 11β moiety acted upon by these enzymes.^6,15^ This observation may explain the favorable bone biomarker profile observed in the vamorolone-treated groups compared with corticosteroids.

Corticosteroid drugs (and endogenous cortisol) potently, broadly, and acutely inhibit the hypothalamic-pituitary-adrenal (HPA) axis, and long-term use can lead to adrenal insufficiency.^16^ In this trial, boys with DMD showed an unexpected high incidence of adrenal insufficiency at baseline by both ACTH-stimulation and morning cortisol measures. All drug treatment groups showed further suppression of the HPA axis from baseline ACTH stimulation tests and morning cortisol compared with placebo (Table 3). The incidental finding of adrenal insufficiency at baseline needs further study. Clinical symptoms of adrenal insufficiency overlap with those of DMD (poor growth, fatigue), and the treatment for adrenal insufficiency is supplemental glucocorticoids. It is intriguing to speculate that some of the efficacy of both corticosteroids and vamorolone may be treatment of adrenal insufficiency. In addition, a gene variation that causes congenital adrenal hypoplasia, *NR0B1* (encoding DAX1), is adjacent to the *DMD* gene on the X chromosome, providing a potential mechanistic link between DMD and adrenal insufficiency.

### Limitations

Limitations of the study include the relatively short study period (24 weeks)—in part to limit length of the placebo group and the withholding of standard of care—use of a single corticosteroid regimen, narrow age range of the study population (4 to <7 years at enrollment), relatively small number of participants per group (although well powered), and missing data on some secondary efficacy outcomes owing to COVID-19 pandemic limitations on participant research visits. The analysis presented here was limited to treatment period 1 (24 weeks). Analysis of treatment period 2 (24 weeks) inclusive of longer-term treatment and crossover groups (placebo to vamorolone; prednisone to vamorolone), and more complete risk/benefit assessments are underway.

## Conclusions

In this randomized clinical trial, vamorolone was shown to be effective and safe in the treatment of boys with DMD over a 24-week treatment period. Vamorolone is a dissociative steroid that separates efficacy (improvement of motor outcomes in DMD) from some safety concerns seen with the corticosteroid class (growth deceleration, bone biomarkers abnormalities). The proven efficacy over a broad dose range (2-6 mg/kg per day) may enable physicians to adjust dose based on clinical observations and patient preferences.

## Notes

Supplement 1. Trial Protocol

Supplement 2. Protocol Amendment 1

Supplement 3. Protocol Amendment 2

Supplement 4. Protocol Amendment 3

Supplement 5. Protocol Amendment 4

Supplement 6. Protocol Clarification Letter

Supplement 7. Statistical Analysis Plan

Supplement 8. **eTable 1. **Study Populations **eTable 2. **Parent-Reported PARS III Outcome of Anxiety and Depression **eFigure. **Post hoc Analysis of Percentage Change in Motor End Points at 24 Weeks vs Baseline **eTable 3. **Treatment-Emergent Adverse Events (TEAEs) **eTable 4. **Laboratory Measures of Pharmacodynamic Safety Biomarkers **eTable 5. **Central Laboratory “LOW” Calls for Cortisol Measures in Placebo Group and at Screening in Drug-Treated Groups **eTable 6. **Percentage of Participants With Peak ACTH-Stimulated Cortisol Measures <500 nmol/L at Both 30 Minutes and 60 Minutes

Supplement 9. Data Sharing Statement
