# Supplementary material for: Efficacy and Safety of Vamorolone vs Placebo and Prednisone Among Boys With Duchenne Muscular Dystrophy: A Randomized Clinical Trial
Source: JAMA Neurol. 2022 Aug 29;79(10):1005–14. doi: 10.1001/jamaneurol.2022.2480 (PMC9425287; doi:10.1001/jamaneurol.2022.2480)
Supplement: Supplement 3. — Protocol Amendment 2 [file jamaneurol-e222480-s003.pdf]

This project has received funding from the European Union' Horizon 2020 research and innovation programme under grant agreement No 667078

# CLINICAL STUDY PROTOCOL

## Amendment #2

**Document Title:** **Amendment #2 for a Phase IIb Randomized, Double-blind, Parallel Group, Placebo- and Active-controlled Study with Double-Blind Extension to Assess the Efficacy and Safety of Vamorolone in Ambulant Boys with Duchenne Muscular Dystrophy (DMD)**

**Protocol Number:** VBP15-004

**Document Number:** VBP15-004-A2 (Version 1.2)

**FDA IND No.:** 118,942

**Investigational Product:** Vamorolone

**Sponsor:** ReveraGen BioPharma, Inc.
155 Gibbs St.
Suite 433
Rockville, MD 20850

**Medical Monitors:**

**Outside North America** John van den Anker, M.D., Ph.D.
Phone: (202) 309 3735
Email: john.vandenanker@reveragen.com

**North America** Benjamin Schwartz, M.D., Ph.D.
Phone: (314) 220 7067
Email: bdschw@camdengroup.com

**Document Date:** 05 March 2019

**This document represents proprietary information belonging to ReveraGen BioPharma, Inc. Unauthorized reproduction of the whole or part of the document by any means is strictly prohibited. This protocol is provided to you as a Principal Investigator, potential Investigator or consultant for review by you, your staff and Institutional Review Board or Independent Ethics Committee. The information contained in this document is privileged and confidential and, except to the extent necessary to obtain informed consent, must not be disclosed unless such disclosure is required by federal or state law or regulations. Persons to whom the information is disclosed in confidence must be informed that the information is confidential and must not be disclosed by them to a third party.**

# SIGNATURES OF AGREEMENT FOR VBP15-004-A2 (Version 1.2)

## Amendment #2 for a Phase IIb Randomized, Double-blind, Parallel Group, Placebo- and Active-controlled Study with Double-Blind Extension to Assess the Efficacy and Safety of Vamorolone in Ambulant Boys with Duchenne Muscular Dystrophy (DMD)

**Reviewed and Approved by:**

| | |
|---|---|
| ____________________________________ | ________________________________ |
| Eric Hoffman, Ph.D.<br>Chief Executive Officer<br>ReveraGen BioPharma, Inc. | Date |
| ____________________________________ | ________________________________ |
| Michela Guglieri, M.D.<br>Study Chair<br>John Walton Muscular Dystrophy Research Centre | Date |
| ____________________________________ | ________________________________ |
| Paula R. Clemens, M.D.<br>Study Vice Chair<br>University of Pittsburgh School of Medicine | Date |
| ____________________________________ | ________________________________ |
| John van den Anker, M.D., Ph.D.<br>Medical Monitor<br>Chief Medical Officer<br>ReveraGen BioPharma, Inc. | Date |
| ____________________________________ | ________________________________ |
| Benjamin D. Schwartz, M.D., Ph.D.<br>Consulting Medical Monitor<br>The Camden Group, LLC | Date |
| ____________________________________ | ________________________________ |
| Laurel J. Mengle-Gaw, Ph.D.<br>Consulting Clinical Monitor<br>The Camden Group, LLC | Date |
| ____________________________________ | ________________________________ |
| Mark J. Jaros, Ph.D.<br>Consulting Statistician<br>Summit Analytical, LLC | Date |

# INVESTIGATOR PROTOCOL AGREEMENT

**Amendment #2 for a Phase IIb Randomized, Double-blind, Parallel Group, Placebo- and Active-controlled Study with Double-Blind Extension to Assess the Efficacy and Safety of Vamorolone in Ambulant Boys with Duchenne Muscular Dystrophy (DMD)**

**PROTOCOL NUMBER:** **VBP15-004**

**DOCUMENT NUMBER:** **VBP15-004-A2 (Version 1.2)**

**SPONSOR:** **ReveraGen BioPharma, Inc.**

**DOCUMENT DATE:** **05 March 2019**

***By my signature, I confirm that my staff and I have carefully read and understand this protocol, protocol amendment, amended protocol, or revised protocol and agree to comply with the conduct and terms of the study specified herein and with any other study conduct procedures provided by ReveraGen BioPharma, Inc.***

***I agree to conduct the study according to this protocol and the obligations and requirements of clinical Investigators and all other requirements set out in the Declaration of Helsinki listed in 21 CFR part 312, and ICH principles of Good Clinical Practice (GCP) and in accordance with all applicable laws, guidances and directives of the jurisdiction where the study is being conducted. I will not initiate this study without the approval of an Institutional Review Board (IRB) or Independent Ethics Committee (IEC).***

***I understand that, should the decision be made by ReveraGen BioPharma, Inc. to terminate prematurely or suspend the study at any time for whatever reason, such decision will be communicated to me in writing. Conversely, should I decide to withdraw from execution of the study, I will communicate immediately such decision in writing to ReveraGen BioPharma, Inc.***

***For protocol amendments, I agree not to implement the amendment without agreement from ReveraGen BioPharma, Inc. and prior submission to and written approval (where required) from the IRB/IEC, except when necessary to eliminate an immediate hazard to the subjects, or for administrative aspects of the study (where permitted by all applicable regulatory requirements).***

____________________________________ ____________________
Investigator's Signature Date

____________________________________
Investigator's Name (Please print)
Address (Please print):

**RETAIN THE ORIGINAL SIGNED AGREEMENT AT YOUR SITE AND RETURN AN ELECTRONIC SIGNED COPY TO REVERAGEN BIOPHARMA, INC., OR DESIGNEE**

## SERIOUS ADVERSE EVENT CONTACT INFORMATION

**In the event of a serious adverse event (SAE) (see Section 7.5), the Investigator will complete the SAE electronic case report form within 24 hours of first awareness of the event. In the unlikely event that the electronic study database is inaccessible and the Investigator is unable to complete the SAE electronic case report form within 24 hours, the SAE Notification Form (pdf) should be completed and emailed or printed/faxed to the PRA safety management team within 24 hours, using the contact information below:**

**In United States and Canada:**

**Email: CHOSafety@prahs.com**

**Drug Safety Fax: 1 888 772 6919 or 1 434 951 3482**

**SAE Questions: Drug Safety Helpline: 1 800 772 2215**

**In Europe, Asia, Pacific, Africa and Australia:**

**Email: MHGSafety@prahs.com**

**Drug Safety Fax: +44 1792 525720**

**SAE Questions: Drug Safety Helpline: +49 621 878 2154**

# PROTOCOL AMENDMENT TRACKING

| Document | Document Number | Approval Date |
|---|---|---|
| Original Protocol | VBP15-004 | 15 December 2017 |
| Amendment #1 | VBP15-004-A1 (Version 1.1) | 04 May 2018 |
| Amendment #2 | VBP15-004-A2 (Version 1.2) | 05 March 2019 |

## Reasons for Protocol Amendment #2:

1. To correct contact information for the Medical Monitor;
2. To update Section 1.3 Clinical Experience with results from the VBP15-002 and VBP15-003 clinical trials in DMD boys;
3. To update Section 1.5 Overall Benefit/Risk;
4. To revise exclusion criterion #7 regarding prior use of glucocorticoids;
5. To add an exclusion criterion excluding use of live attenuated vaccines within 14 days prior to first dose of study medication;
6. To exclude subjects who have siblings currently enrolled in, or intending to enroll in during the subject's participation in this study, any vamorolone study or Expanded Access Program;
7. To delete use of the Child Behavior Checklist behavioral assessment tool;
8. To clarify that the PARS III assessment tool is a measure of behavior/neuropsychology, and the PODCI is a measure of physical functioning;
9. To specify that the PODCI results will be analyzed for vamorolone vs. placebo, and the PARS III results will be analyzed for vamorolone vs. prednisone;
10. To add assessment of spine BMD, total body BMD, spine and total body bone mass, and total body composition to safety endpoints assessed by DXA, and to add assessment of lean mass, Lean Mass Index, and Fat Mass Index to total body composition measurements;
11. To allow each daily dose of study medication suspension and tablets during Weeks 1-28 to be taken in any order;
12. To remove the requirement that the single dose of hydrocortisone taken 24 hours after the final dose of study medication in Treatment Periods #1 and #2 must be taken at home;
13. To add time windows of ± 5 minutes to the 30- and 60-minute post-Cosyntropin blood draws for ACTH stimulation testing;
14. To clarify that all subjects should be covered with" stress dosing" of hydrocortisone (or prednisone) during times of illness, injury, or surgery;
15. To clarify that results of the Week 24 ACTH stimulation test will not be released to the study sites;
16. To add collection of 6 mL of blood at the Week 24 Visit for DNA testing for candidate genetic modifiers of DMD;

17. To clarify that subjects who opt to continue treatment with vamorolone at study completion may be given access to vamorolone through an additional vamorolone study or general access program;
18. To remove the responsibility of the DSMB to review accumulating study efficacy data, in response to FDA feedback;
19. To clarify the circumstances under which additional subjects may be enrolled;
20. To clarify that an email with each subject randomization number will not be sent to the site investigator at the time of randomization;
21. To clarify the blinding status following the Week 24 analyses;
22. To add sensitivity analyses for missing data;
23. To add the 6MWT (comparison of vamorolone vs. prednisone) to the sequential testing procedure for the secondary efficacy endpoints;
24. To specify that statistical analyses will be performed using SAS® version 9.4 or later;
25. To define time windows around scheduled study visits;
26. To update the Schedule of Study Activities to clarify that study entry criteria should be rechecked and confirmed at Baseline Day -1;
27. To update the Schedule of Study Activities to clarify that study medication dosing on in-clinic dosing days should occur after all fasting blood draws;
28. To add additional flexibility in the scheduling of some Week 24 and Week 48 safety assessments;
29. To revise the Schedule of Study Activities to specify that subject diaries dispensed at Week 24 should be returned and redispensed at Week 24 F/U, and diaries dispensed at Week 48 should be returned at Week 48 F/U for all subjects and redispensed to subjects participating in the Dose-tapering Period;
30. To clarify that final disposition of unused CTM at the study sites may occur throughout and at the end of the study;
31. To update version of the Investigator's Brochure referenced from Version 7, March 26, 2018 to Version 8, September 24, 2018; and
32. To correct typographical and grammatical errors.

The Sections changed by amendment are listed in was/is format in Appendix 15.1.

# STUDY SYNOPSIS

| | |
|---|---|
| **Protocol Title** | A Phase IIb Randomized, Double-blind, Parallel Group, Placebo- and Active-controlled Study with Double-Blind Extension to Assess the Efficacy and Safety of Vamorolone in Ambulant Boys with Duchenne Muscular Dystrophy (DMD) |
| **Name of Sponsor** | ReveraGen BioPharma, Inc. |
| **Protocol Number** | VBP15-004 |
| **Drug Substance** | delta-1,4,9(11)-pregnatriene-17-alpha,21-dihydroxy-16-alpha-methyl-3,20-dione |
| **Investigational Drug Product** | Vamorolone, 1.33% and 4.0% wt/wt suspension for oral dosing |
| **Phase of Development** | Phase IIb |
| **Indication** | Treatment of Duchenne muscular dystrophy (DMD) |
| **Primary Objectives** | 1. To compare the efficacy of vamorolone administered orally at daily doses of 2.0 mg/kg and 6.0 mg/kg over a 24-week treatment period vs. placebo in ambulant boys ages 4 to <7 years with DMD; and<br>2. To evaluate the safety and tolerability of vamorolone administered orally at daily doses of 2.0 mg/kg and 6.0 mg/kg in ambulant boys ages 4 to <7 years with DMD. |
| **Secondary Objectives** | 1. To compare the safety of vamorolone administered orally at daily doses of 2.0 mg/kg and 6.0 mg/kg over a 24-week treatment period vs. daily prednisone 0.75 mg/kg in ambulant boys ages 4 to <7 years with DMD;<br>2. To compare the efficacy of vamorolone administered orally at daily doses of 2.0 mg/kg and 6.0 mg/kg over a 24-week treatment period vs. daily prednisone 0.75 mg/kg in ambulant boys ages 4 to <7 years with DMD;<br>3. To compare the efficacy of vamorolone administered orally at daily doses of 2.0 mg/kg vs. vamorolone administered orally at daily doses of 6.0 mg/kg over a 24-week treatment period in ambulant boys ages 4 to <7 years with DMD;<br>4. To compare the efficacy of vamorolone administered orally at daily doses of 2.0 mg/kg and 6.0 mg/kg over a 48-week treatment period in ambulant boys ages 4 to <7 years with DMD vs. untreated DMD historical controls;<br>5. To compare the safety of vamorolone administered orally at daily doses of 2.0 mg/kg and 6.0 mg/kg over a 48-week treatment period in ambulant boys ages 4 to <7 years with DMD vs. prednisone-treated DMD historical controls; and<br>6. To evaluate the population pharmacokinetics (PK) of vamorolone administered orally at daily doses of 2.0 mg/kg and 6.0 mg/kg in ambulant boys ages 4 to <7 years with DMD. |
| **Exploratory Objectives** | 1. To evaluate the satisfaction with treatment of vamorolone administered orally at daily doses of 2.0 mg/kg and 6.0 mg/kg over a 24-week treatment period vs. daily prednisone 0.75 mg/kg in ambulant boys ages 4 to <7 years with DMD;<br>2. To evaluate the effect of vamorolone administered orally at daily doses of |

2.0 mg/kg and 6.0 mg/kg over a 24-week treatment period vs. daily prednisone 0.75 mg/kg on behavior and neuropsychology;

3. To evaluate the effect of vamorolone administered orally at daily doses of 2.0 mg/kg and 6.0 mg/kg over a 24-week treatment period vs. placebo on physical functioning;
4. To assess the ease of administration of the study medication suspension to ambulant boys ages 4 to <7 years with DMD;
5. To compare the effects of vamorolone administered orally at daily doses of 2.0 mg/kg and 6.0 mg/kg over a 24-week treatment period vs. placebo on potential serum pharmacodynamics (PD) biomarkers of safety and efficacy in ambulant boys ages 4 to <7 years with DMD;
6. To compare the effects of vamorolone administered orally at daily doses of 2.0 mg/kg and 6.0 mg/kg over a 24-week treatment period vs. daily prednisone 0.75 mg/kg on potential serum PD biomarkers of safety and efficacy in ambulant boys ages 4 to <7 years with DMD; and
7. To determine if candidate genetic modifiers of DMD (gene polymorphisms associated with disease severity, or response to glucocorticoid treatment) are similarly associated with vamorolone-treated DMD patients (baseline disease severity, or response to vamorolone or prednisone treatment).

**Study Design**

This Phase IIb study is a randomized, double-blind, parallel group, placebo and active-controlled study to evaluate the efficacy, safety, PD, and population PK of vamorolone administered orally at daily doses of 2.0 mg/kg and 6.0 mg/kg versus prednisone 0.75 mg/kg/day and placebo over a Treatment Period of 24 weeks, and to evaluate persistence of effect over a Treatment Period of 48 weeks in ambulant boys ages 4 to <7 years with DMD.

The study is comprised of a 5-week Pretreatment Screening Period, a 1-day Pretreatment Baseline Period, a 24-week Treatment Period #1 (Weeks 1-24), a 4-week Transition Period (Weeks 25-28), a 20-week Treatment Period #2 (Weeks 28 + 1 day to 48), and a 4-week Dose-tapering Period (Weeks 49-52).

Subjects will be randomized to one of six treatment groups in a 2:2:1:1:1:1 ratio, where the two prednisone groups in Treatment Period #1 (Groups 3 and 4) will be combined and the two placebo groups in Treatment Period #1 (Groups 5 and 6) will be combined, effectively resulting in a 1:1:1:1 randomization (vamorolone 2.0 mg/kg/day : vamorolone 6.0 mg/kg/day : prednisone 0.75 mg/kg/day : placebo) for Treatment Period #1:

**Study Randomization Schedule**

| Group | # | Treatment Period #1 | Treatment Period #2 |
|---|---|---|---|
| 1 | 30 | Vamorolone, 2.0 mg/kg/day → | Vamorolone, 2.0 mg/kg/day |
| 2 | 30 | Vamorolone, 6.0 mg/kg/day → | Vamorolone, 6.0 mg/kg/day |
| 3 | 15 | Prednisone, 0.75 mg/kg/day → | Vamorolone, 2.0 mg/kg/day |
| 4 | 15 | Prednisone, 0.75 mg/kg/day → | Vamorolone, 6.0 mg/kg/day |
| 5 | 15 | Placebo → | Vamorolone, 2.0 mg/kg/day |
| 6 | 15 | Placebo → | Vamorolone, 6.0 mg/kg/day |

# = number of planned randomized subjects in each group

Subjects will be stratified based on age at study entry (<6 vs. ≥ 6 years). During the 4-week Transition Period between Treatment Period #1 and Treatment Period #2, all subjects will continue on the same oral suspension (vamorolone

| | |
|---|---|
| | 2.0 mg/kg or 6.0 mg/kg, or matching placebo) they received during Treatment Period #1 and all subjects will have their tablet dose tapered to zero. Thus, subjects randomized to receive vamorolone during Treatment Period #1 (Groups 1 and 2) will continue to receive vamorolone at the same dose, while subjects randomized to receive prednisone will have their dose tapered to zero, and subjects randomized to placebo will continue to receive placebo.<br>The prednisone group will be used as an active control comparison for safety and efficacy endpoints as requested by the European Medicines Agency (EMA). The placebo group will be used as comparator for efficacy endpoints (superiority model) as requested by the EMA and Food and Drug Administration (FDA) protocol advisory board. Although glucocorticoids are part of the care recommendations for DMD, their adverse effect profile has limited their use. The age at which glucocorticoids should be started in DMD boys is uncertain, ranging from 4 to 7 years, based on a balance between benefits and side effects. In view of the age inclusion criteria and duration of the placebo-controlled study period (6 months), the use of a placebo group has been considered acceptable as in clinical practice it will not cause a real delay in prescription of an accepted treatment for this condition. Any exposure of placebo longer than 6 months was considered unethical.<br>At the end of the Treatment Period #2, subjects may be given access to vamorolone through an additional study or general access program, or given the option to transition to standard of care treatment for DMD (may include glucocorticoids). Subjects completing VBP15-004 and enrolling directly into an additional vamorolone study or general access program to receive vamorolone will not need to taper their vamorolone dose prior to enrollment. All other subjects will begin a 4-week double-blind Dose-tapering Period during which the dose of study medication will be progressively reduced and discontinued. |
| **Planned Sample Size** | A total of approximately 120 subjects will be randomized (2:2:1:1:1:1) to treatment as follows (treatment assignment in Treatment Period #1 → treatment assignment in Treatment Period #2):<br>Vamorolone 2.0 mg/kg/day → Vamorolone 2.0 mg/kg/day (n=30);<br>Vamorolone 6.0 mg/kg/day → Vamorolone 6.0 mg/kg/day (n=30);<br>Prednisone 0.75 mg/kg/day → Vamorolone 2.0 mg/kg/day (n=15);<br>Prednisone 0.75 mg/kg/day → Vamorolone 6.0 mg/kg/day (n=15);<br>Placebo → Vamorolone 2.0 mg/kg/day (n=15); or<br>Placebo → Vamorolone 6.0 mg/kg/day (n=15). |
| **Inclusion Criteria** | 1. Subject's parent(s) or legal guardian(s) has (have) provided written informed consent and Health Insurance Portability and Accountability Act (HIPAA) authorization, where applicable, prior to any study-related procedures; participants will be asked to give written or verbal assent according to local requirements<br>2. Subject has a centrally confirmed (by TRiNDS central genetic counselor[s]) diagnosis of DMD as defined as:<br>• Dystrophin immunofluorescence and/or immunoblot showing complete dystrophin deficiency, and clinical picture consistent with typical DMD, OR<br>• Identifiable mutation within the DMD gene (deletion/duplication of one or more exons), where reading frame can be predicted as 'out-of-frame,' and clinical picture consistent with typical DMD, OR<br>• Complete dystrophin gene sequencing showing an alteration (point mutation, duplication, other) that is expected to preclude production of |

| | |
|---|---|
| | the dystrophin protein (i.e., nonsense mutation, deletion/duplication leading to a downstream stop codon), with a clinical picture consistent with typical DMD;<br>3. Subject is ≥ 4 years and <7 years of age at time of enrollment in the study;<br>4. Subject weighs >13.0 kg and ≤ 39.9 kg at the Screening Visit;<br>5. Subject is able to walk independently without assistive devices;<br>6. Subject is able to complete the Time to Stand Test (TTSTAND) without assistance in <10 seconds, as assessed at the Screening Visit;<br>7. Clinical laboratory test results are within the normal range at the Screening Visit, or if abnormal, are not clinically significant, in the opinion of the Investigator. [Note: Serum gamma glutamyl transferase (GGT), creatinine, and total bilirubin all must be ≤ upper limit of the normal range at the Screening Visit];<br>8. Subject has evidence of chicken pox immunity as determined by presence of IgG antibodies to varicella, as documented by a positive test result from the local laboratory at the Screening Visit;<br>9. Subject is able to swallow tablets, as confirmed by successful test swallowing of placebo tablets during the Screening Period; and<br>10. Subject and parent(s)/guardian(s) are willing and able to comply with scheduled visits, study drug administration plan, and study procedures. |
| **Exclusion Criteria** | 1. Subject has current or history of major renal or hepatic impairment, diabetes mellitus or immunosuppression;<br>2. Subject has current or history of chronic systemic fungal or viral infections;<br>3. Subject has had an acute illness within 4 weeks prior to the first dose of study medication;<br>4. Subject has used mineralocorticoid receptor agents, such as spironolactone, eplerenone, canrenone (canrenoate potassium), prorenone (prorenoate potassium), mexrenone (mexrenoate potassium) within 4 weeks prior to the first dose of study medication;<br>5. Subject has a history of primary hyperaldosteronism;<br>6. Subject has evidence of symptomatic cardiomyopathy [Note: Asymptomatic cardiac abnormality on investigation would not be exclusionary];<br>7. Subject is currently being treated or has received previous treatment with oral glucocorticoids or other immunosuppressive agents [Notes: Past transient use of oral or inhaled glucocorticoids or other oral immunosuppressive agents for no longer than 1 month cumulative, with last use at least 3 months prior to first dose of study medication, will be considered for eligibility on a case-by-case basis, unless discontinued for intolerance. Inhaled and/or topical glucocorticoids are permitted if last use is at least 4 weeks prior to first dose of study medication or if administered at stable dose beginning at least 4 weeks prior to first dose of study medication and anticipated to be used at the stable dose regimen for the duration of the study];<br>8. Subject has an allergy or hypersensitivity to the study medication or to any of its constituents;<br>9. Subject has used idebenone within 4 weeks prior to the first dose of study medication;<br>10. Subject has severe behavioral or cognitive problems that preclude participation in the study, in the opinion of the Investigator; |

| | |
|---|---|
| | 11. Subject has previous or ongoing medical condition, medical history, physical findings or laboratory abnormalities that could affect safety, make it unlikely that treatment and follow-up will be correctly completed or impair the assessment of study results, in the opinion of the Investigator;<br>12. Subject is taking (or has taken within 4 weeks prior to the first dose of study medication) herbal remedies and supplements which can impact muscle strength and function (e.g., Co-enzyme Q10, creatine, etc);<br>13. Subject is taking (or has taken within 3 months prior to the first dose of study medication) any medication indicated for DMD, including Exondys51 and Translarna;<br>14. Subject has been administered a live attenuated vaccine within 14 days prior to the first dose of study medication;<br>15. Subject is currently taking any other investigational drug or has taken any other investigational drug within 3 months prior to the first dose of study medication;<br>16. Subject has a sibling who is currently enrolled in any vamorolone study or Expanded Access Program, or who intends to enroll in any vamorolone study or Expanded Access Program during the subject's participation in the VBP15-004 study; or<br>17. Subject has previously been enrolled in the study.<br>Note: Any parameter/test may be repeated at the Investigator's discretion during Screening to determine reproducibility. In addition, subjects may be rescreened if ineligible due to a transient condition which would prevent the subject from participating, such as an upper respiratory tract infection or injury, or if ineligible due to negative anti-varicella IgG antibody test result. |
| **Number of Centers** | The study will be conducted at approximately 30 study sites in approximately 15 countries, predominantly in the European Union (EU). |
| **Study Period** | First subject screened: 1Q 2018<br>Last subject last visit: 2Q 2020 |
| **Study Duration** | Up to approximately 36 months total duration |
| **Individual Subject Study Duration** | Up to approximately 57 weeks:<br>• Screening Period: up to 5 weeks<br>• Treatment Period #1: 24 weeks<br>• Transition Period: 4 weeks<br>• Treatment Period #2: 20 weeks<br>• Dose-tapering Period: 4 weeks (only for subjects who will transition off vamorolone at the end of the study)<br>Subjects who complete the Treatment Period Week 48 assessments may be given the option of continuing vamorolone treatment in an additional vamorolone study or general access program under separate protocol. Subjects who continue directly with vamorolone treatment in the additional vamorolone study or general access program will be discharged from the VBP15-004 study following completion of all Week 48 assessments and the Week 48 Follow-up Visit ACTH Stimulation Test, and will not participate in the Dose-tapering Period. |
| **Study Drug Formulation, Dosage &** | **Treatment Period #1**<br>Vamorolone 1.33% and vamorolone 4.0% wt/wt oral suspensions (investigational medicine), prednisone (active-control) or placebo will be |

| **Administration** | administered once daily over the 24-week Treatment Period #1. Subjects receiving 2.0 mg/kg/day will be administered the vamorolone 1.33% wt/wt oral suspension, and subjects receiving 6.0 mg/kg/day will be administered the vamorolone 4.0% wt/wt oral suspension. To maintain the blind, matched suspension (vamorolone or placebo) and matched tablets (prednisone or placebo) will be administered. Each subject will receive a dose of suspension (vamorolone or placebo) and tablets (prednisone or placebo) each day. The number of tablets of prednisone 5 mg or matching placebo to be administered, based on subject weight and a prednisone dose of 0.75 mg/kg, is shown in the table below: |
|---|---|

**Prednisone and Placebo Tablet Dosing**

| Band | Weight range in kg | Weight used for calculation of dose per kg | Dose in mg based on 0.75 mg/kg prednisone | Number tablets of prednisone (5 mg) or matching placebo for given weight range |
|---|---|---|---|---|
| A | 13-19.9 | 13.33 kg | 10 mg | 2 |
| B | 20-25.9 | 20.00 kg | 15 mg | 3 |
| C | 26-32.9 | 26.67 kg | 20 mg | 4 |
| D | 33-39.9 | 33.33 kg | 25 mg | 5 |

Study drugs will be administered in the study unit on Day 1, and at the Week 2, Week 12 and Week 24 study visits; all other doses will be administered at home. Study drug oral suspensions will be administered by mouth using a volumetric syringe. Following administration of the dose of study drug suspension, the syringe will be filled once with water and the water will be administered by mouth using the volumetric syringe. Prednisone or matching placebo tablets will be taken either immediately before or immediately after the dose of suspension. The subject will then drink approximately 50 mL (approximately 2 ounces) of water to ensure the full dose has been ingested.

The daily dose of study medication should be taken with breakfast including at least 8 g of fat (approximately 8 ounces [240 mL] of full-fat milk or equivalent high-fat food portion). There are no other food or drink restrictions before or after dosing.

**Transition Period**

Vamorolone 1.33% wt/wt or 4.0% wt/wt (investigational medicine) or placebo oral suspension will continue to be administered once daily over the 4-week Transition Period. Prednisone (active control) and placebo tablets will be tapered over the 4-week Transition Period. All study medication will be administered once daily, in the same manner as during Treatment Period #1.

**Treatment Period #2 and Dose-tapering Period**

Vamorolone 1.33% wt/wt or 4.0% wt/wt oral suspension (investigational medicine) will be administered once daily over the 20-week Treatment Period #2, and during the 4-week Dose-tapering Period, as applicable. No study drug tablets are administered during Treatment Period #2 or the Dose-tapering Period. The oral suspension study medication will be administered once daily, in the same manner as during Treatment Period #1.

| **Study Summary** | This Phase IIb study is a randomized, double-blind, parallel group, placebo- and active-controlled study with double-blind extension to evaluate the long-term efficacy, safety, tolerability, PD, and population PK of vamorolone (the investigational medicine) compared to prednisone (active control) and placebo |
|---|---|

| | |
|---|---|
| | over a Treatment Period of 24 weeks in boys ages 4 to <7 years with DMD, and determine the persistence of effect over a total Treatment Period of 48 weeks.<br><br>Study drug dosing will occur from Day 1 until the Week 48 Visit. Study drug dosing will occur at home on all days except the Day 1, Week 2, Week 12, Week 24, Week 28, Week 30, Week 40, and Week 48 study visits, when dosing will occur at the study site.<br><br>Subjects will be assessed for safety and tolerability, clinical efficacy, PD, and population PK at scheduled visits throughout the study. Treatment Period #1, Transition Period, and Treatment Period #2 study visits will occur at Day 1, Week 2, Week 6, Week 12, Week 18, Week 24, Week 28, Week 30, Week 34, Week 40 and Week 48; all subjects will return to the clinical site for a Week 24 Follow-up Visit and for a Week 48 Follow-up Visit, 48 ± 3 hours after administration of the final dose of Treatment Period #1 and Treatment Period #2 study medication, respectively, for ACTH Stimulation testing. Adverse events, including SAEs, and concomitant medications will be recorded throughout the study.<br><br>Subject diaries will be dispensed at the Day 1 Visit and at each study visit thereafter through Week 48 to record AEs, changes to concomitant medications taken during the study, and any missed or incomplete doses of study medication.<br><br>There is flexibility in the timing of completion of some of the scheduled Week 24 and Week 48 assessments. The scheduled physical examination, weight, vital signs, clinical laboratory tests, blood draws for PD biomarker analysis, blood draw for DNA testing (Week 24 only), Ease of Study Medication Administration Assessment, PODCI, PARS III, and functional assessments (TTSTAND, TTCLIMB, TTRW, NSAA, 6MWT, hand-held myometry, ROM) must all be performed on the date of the Week 24 or Week 48 dose of study medication. The 12-lead ECG may be performed on the date of the Week 24 or Week 48 dose of study medication, the day following the Week 24 or Week 48 dose of study medication, or the day of the Week 24 or Week 48 Follow-up Visit. For the Week 24 assessments, completion of the DXA scan, spine X-rays, Fracture Questionnaire, 2-D echocardiography, eye examination, TSQM, and Blindedness Assessment may be performed up to 7 days following the date of the Week 24 dose of study medication to accommodate need for additional scheduling flexibility. For the Week 48 assessments, completion of the DXA scan, Fracture Questionnaire, 2-D echocardiography, eye examination, and TSQM may be performed on the date of the final Week 48 dose of study medication, the day following the Week 48 dose of study medication, or the day of the Week 48 Follow-up Visit for subjects who will receive additional vamorolone therapy by enrolling directly into an additional vamorolone study or general access program, or up to 7 days following the date of the final Week 48 dose of study medication for subjects participating in the Dose-tapering Period.<br><br>A Transition Period of 4 weeks in duration follows the end of Treatment Period #1 for all subjects. During this Transition Period, all subjects will continue to receive the liquid formulation (vamorolone 2.0 mg/kg or 6.0 mg/kg, or matching placebo) they received during Treatment Period #1 and will be tapered off their study medication tablets (prednisone or matching placebo). Site study staff will contact the parent(s)/guardian(s) by telephone at Week 26 to ensure that the tablet tapering is proceeding according to protocol, to assess potential signs or symptoms indicative of adrenal suppression, and to address any questions the parent(s)/guardian(s) may have. All subjects will return to the clinical site for the Week 28 assessments, prior to receiving their first dose of Treatment Period #2 study medication on the day after the Week 28 Visit (Week 28 + 1 day).<br><br>Subjects who complete the VBP15-004 study assessments through the Week 48 |

<table>
<tr><td></td><td>Visit and Week 48 Follow-up Visit may be given access to vamorolone through an additional study or general access program, or given the option to transition to standard of care treatment (including glucocorticoids) for DMD. Standard of care treatment for DMD may be offered to the subject following completion of the Phase IIb VBP15-004 study, if the subject’s parent or guardian does not wish to enroll the subject in the additional vamorolone study or general access program and/or the Investigator feels it to be in the best interest of the subject.<br><br>Subjects who complete the VBP15-004 study and will enroll directly into an additional vamorolone study or general access program to continue vamorolone treatment will be discharged from the VBP15-004 study following completion of all Week 48 assessments and the Week 48 Follow-up Visit ACTH Stimulation Test. Subjects who will not continue vamorolone treatment in an additional vamorolone study or general access program, including those subjects who will transition to standard of care treatment for DMD, will have their vamorolone dose tapered during a 4-week Dose-tapering Period, prior to discharge from the study. Site study staff will contact the parent(s)/guardian(s) by telephone at Week 50 to ensure that the dose tapering is proceeding according to protocol, to assess potential signs or symptoms of adrenal suppression, and to address any questions the parent(s)/guardian(s) may have.<br><br>In the event that any clinical or laboratory parameters remain abnormal at the time of discharge from the study, the subject will be followed medically, as clinically indicated.<br><br>Any subject who discontinues the study prior to the Week 24 Visit should return to the study unit for scheduled Week 24 assessments and the Week 24 Follow-up Visit ACTH Stimulation Test at the time of early withdrawal, whenever possible; any subject who prematurely discontinues the study after Week 24 but prior to Week 28 should complete the scheduled Week 28 assessments at the time of early withdrawal, whenever possible; and any subject who prematurely discontinues the study after Week 28 but prior to Week 48 should complete the scheduled Week 48 assessments and the Week 48 Follow-up Visit ACTH Stimulation Test at the time of early withdrawal, whenever possible, assuming the subject has not withdrawn consent. Any subject who withdraws early from the study should undergo Early Discontinuation Dose-tapering.</td></tr>
<tr><td>Safety Measures</td><td>• Body Mass Index (BMI)<br>• Weight and Height<br>• Vital signs (sitting blood pressure, heart rate, respiratory rate, and body temperature [modality for determining temperature should be consistent for each subject at all assessment time points throughout the study])<br>• Physical examination<br>• Cushingoid features<br>• Clinical laboratory tests:<br>    ◦ Hematology and clinical chemistry<br>    ◦ Urinalysis<br>    ◦ Lipid profile (triglycerides, total cholesterol, low density lipoprotein [LDL], high density lipoprotein [HDL])<br>    ◦ Vitamin D level<br>• ACTH Stimulation Test<br>• 12-lead electrocardiogram (ECG)<br>• 2-D echocardiography</td></tr>
</table>

| | |
|---|---|
| | • Eye examination<br>• Dual-energy x-ray absorptiometry (DXA) scan<br>• Spine X-ray<br>• Fracture Questionnaire<br>• Clinical signs and symptoms (AEs and SAEs)<br>  ◦ Grading of clinical and clinical laboratory AEs will be according to the Common Terminology Criteria for Adverse Events (CTCAE), v.4.03 |
| **Pharmacodynamic Measures** | • Blood will be collected for serum PD biomarker testing to explore effects of vamorolone on biomarkers of muscle cellular pathology, and biomarkers associated with acute and chronic glucocorticoid treatment (aspects of both safety and efficacy). Secondary outcomes are serum biomarkers bridged to clinical outcomes of safety, including adrenal suppression, insulin resistance, bone turnover, and immune suppression. Exploratory outcomes are serum biomarkers that have been shown to be glucocorticoid-responsive in DMD patients, but not yet bridged to clinical outcomes (safety and efficacy). Samples for analysis of acute and chronic PD biomarker response will be collected at Day 1, Week 12, and Week 24, Week 28, Week 40, and Week 48 (pre-dose), and final Dose-tapering Period Visit. Blood remaining from collected samples may be stored for future exploratory biomarker studies. Blood samples for PD biomarkers, including insulin and glucose, will be collected after subjects have fasted for ≥ 6 hours, and prior to the daily dose of study medication where applicable. |
| **Pharmacokinetic Measures** | • Blood will be collected from all subjects at the Week 30 Visit, at 2 hours post-dose, for vamorolone population PK analysis. |
| **Clinical Efficacy Measures** | • Time to Stand Test (TTSTAND)<br>• Time to Climb 4 steps (TTCLIMB)<br>• Time to Run/Walk 10 meters Test (TTRW)<br>• North Star Ambulatory Assessment (NSAA)<br>• Six-minute Walk Test (6MWT)<br>• Hand-held Myometry (elbow flexors/knee extensors)<br>• Range of Motion (ROM) in the ankles |
| **Exploratory Measures** | • Treatment satisfaction questionnaire (TSQM)<br>• Pediatric Outcome Data Collection Instrument (PODCI)<br>• PARS III questionnaire<br>• Ease of Study Medication Administration Assessment<br>• Blindedness Assessment<br>• DNA testing for candidate genetic modifiers of DMD |
| **Statistical Methods** | **Sample Size:**<br>This is a randomized, double-blind, parallel group, placebo- and active-controlled study. Study medication is administered daily in this Phase IIb trial. Data for untreated subjects from the Cooperative International Neuromuscular Research Group (CINRG) Duchenne Natural History Study and data for prednisone-treated subjects from the CINRG Prednisone study were used to help estimate sample sizes for this study.<br>The primary efficacy outcome is TTSTAND (velocity) change from baseline to Week 24. A sample size of 30 subjects per treatment group (120 total subjects) |

| | |
|---|---|
| | will detect a 0.0674 point difference in mean change from baseline to Week 24 in TTSTAND (velocity) between a vamorolone dose level and placebo, assuming a common standard deviation of 0.08, a two-sided t test, and a Type-I error of 0.025 with approximately 83% power. The Bonferroni adjustment method will be used to control the Type-I error rate at 0.05 due to the multiple comparisons (2 vamorolone dose levels will be tested against placebo).<br>Based on this calculation, a total of approximately 120 subjects will be randomized to treatment with vamorolone 2.0 mg/kg/day (n=30), vamorolone 6.0 mg/kg/day (n=30), prednisone 0.75 mg/kg/day (n=30), or placebo (n=30).<br>Note that subjects in the prednisone and placebo groups will actually be randomized into two groups each:<br>• Prednisone 0.75 mg/kg/day → Vamorolone 2.0 mg/kg/day (n=15);<br>• Prednisone 0.75 mg/kg/day → Vamorolone 6.0 mg/kg/day (n=15);<br>• Placebo → Vamorolone 2.0 mg/kg/day (n=15); or<br>• Placebo → Vamorolone 6.0 mg/kg/day (n=15).<br>These groups will be pooled by initial treatment (prednisone or placebo) for the Treatment Period #1 analyses.<br>If the number of subjects who withdraw early from the study is high, additional subjects may be enrolled to achieve approximately 120 subjects completing the Week 24 Visit assessments.<br>**Analysis Populations:**<br>Three populations will be defined for data analysis: the Safety Population, the modified Intent-to-Treat Population, and the Pharmacokinetic Population.<br>Safety Population<br>All subjects who receive at least one dose of study medication will be included in the Safety Population. The Safety Population is the primary analysis population for safety and PD assessments. Results will be presented "as treated."<br>Modified Intent-to-Treat (mITT) Population<br>All subjects who receive at least one dose of study medication and have at least one post-baseline assessment will be included in the mITT Population. The mITT Population is the primary analysis population for clinical efficacy. Subjects who receive at least one dose of study medication but never have post-baseline assessments will be excluded. Results will be presented "as randomized."<br>Pharmacokinetic (PK) Population<br>All subjects who receive at least one dose of vamorolone study medication and have sufficient data for PK analysis will be included in the PK Population.<br>**General Statistical Considerations:**<br>All measurements will be analyzed based upon the type of distribution and descriptive statistics presented by treatment group and time point, as appropriate. No formal interim statistical analyses are planned, apart from the interim unblinded safety data views and presentations to be created for the Data and Safety Monitoring Board (DSMB). The Week 24 analyses are the primary analyses for this study and will be performed after all subjects have completed Week 24 of Treatment Period #1. The Week 48 analyses (vamorolone versus untreated historical controls and vamorolone versus prednisone-treated historical controls) will be performed after all subjects have completed Treatment Period #2. Missing values for safety outcomes will be treated as missing, unless |

| | |
|---|---|
| | stated otherwise.<br>Baseline measurement is defined as the last non-missing value prior to the first dose of study drug.<br>Treatment Period #1 analyses will be summarized by four treatment groups:<br>• Vamorolone 2.0 mg/kg/day (n=30);<br>• Vamorolone 6.0 mg/kg/day (n=30);<br>• Prednisone 0.75 mg/kg/day (n=30); and<br>• Placebo (n=30).<br>Treatment Period #2 analyses (besides historical control comparison data) will be summarized by six treatment groups:<br>• Vamorolone 2.0 mg/kg/day → Vamorolone 2.0 mg/kg/day (n=30);<br>• Vamorolone 6.0 mg/kg/day → Vamorolone 6.0 mg/kg/day (n=30);<br>• Prednisone 0.75 mg/kg/day → Vamorolone 2.0 mg/kg/day (n=15);<br>• Prednisone 0.75 mg/kg/day → Vamorolone 6.0 mg/kg/day (n=15);<br>• Placebo → Vamorolone 2.0 mg/kg/day (n=15); and<br>• Placebo → Vamorolone 6.0 mg/kg/day (n=15).<br>**Adjustment for Multiple Comparisons:**<br>The primary efficacy endpoint tests each dose of vamorolone vs. placebo using TTSTAND velocity at Week 24 and occurs during Treatment Period #1 of the study. The primary efficacy endpoint supports the primary objective of this study. The study is thus powered for the efficacy comparisons.<br>A multi-branched gatekeeping procedure will be utilized for the primary efficacy endpoint. The primary efficacy endpoint (TTSTAND velocity at Week 24) will be tested first using a Bonferroni adjustment. Any dose that is significant for the primary efficacy endpoint will then have the secondary efficacy endpoints tested sequentially. Further details are provided below.<br>For primary efficacy (TTSTAND velocity), the two vamorolone dose levels will be compared with placebo. To account for these comparisons (two vamorolone dose levels vs. placebo), Bonferroni multiple comparison adjustments will be utilized. Each comparison will be conducted at the 0.025 (0.05/2) alpha level.<br>Secondary efficacy endpoints will be tested sequentially on change from baseline to Week 24 values (each dose of vamorolone vs. placebo or prednisone [6MWT only]). Only the doses that are significant for the primary efficacy endpoint (change in TTSTAND velocity at Week 24) will have the secondary endpoints tested. A 0.025 alpha level will be used for the sequential testing. Testing will stop once a p-value is >0.025 for one of the secondary efficacy endpoints. The Week 24 change from baseline values will be tested using this sequential testing procedure. The order of the secondary efficacy endpoints is as follows.<br>1. Six-minute Walk Test (6MWT) vs. placebo<br>2. Time to Run/Walk 10 meters Test (TTRW) velocity vs. placebo<br>3. 6MWT vs. prednisone<br>4. North Star Ambulatory Assessment (NSAA) vs. placebo<br>5. Hand-held Myometry (knee extensors) vs. placebo<br>6. Hand-held Myometry (elbow flexors) vs. placebo<br>7. Time to Climb 4 Steps (TTCLIMB) velocity vs. placebo<br>8. Range of Motion (ROM) in the ankles vs. placebo<br>All other analyses will not be corrected for multiple comparisons (tests will be |

| | |
|---|---|
| | performed at the 0.05 alpha level), as they will be viewed and handled in the perspective of not testing a formal hypothesis.<br>**Efficacy Analyses:**<br>All evaluations of clinical efficacy will be listed and presented using descriptive statistics per treatment group and time point. The primary efficacy outcome is TTSTAND (velocity). Secondary efficacy outcomes are the NSAA assessment, TTCLIMB, TTRW, 6MWT, hand-held myometry (elbow flexors and knee extensors), and ROM. TTSTAND, TTCLIMB, and TTRW will be analyzed using raw scores and velocity.<br>The primary efficacy outcome TTSTAND (velocity) change from baseline to Week 24 will be compared between each of the two different vamorolone dose groups and the placebo group using a restricted maximum likelihood (REML)-based mixed model for repeated measures (MMRM). This model includes fixed effects for treatment (vamorolone 2.0 mg/kg/day, vamorolone 6.0 mg/kg/day, prednisone 0.75 mg/kg/day, and placebo), week, baseline TTSTAND, age group (per stratification), and the treatment-by-week interaction. Study week will be included in the model as a categorical variable (Weeks 6, 12, and 24) along with the treatment-by-week interaction. Within this model, pairwise comparisons (using least squares [LS] mean contrasts) will be made to compare TTSTAND at 24 weeks for each vamorolone dose level with placebo separately (primary efficacy outcome), for each vamorolone dose level with prednisone separately (secondary analysis), and for the high vamorolone dose level with the low vamorolone dose level (secondary analysis). Treatments will also be compared at other weeks as secondary analyses. An unstructured covariance matrix will be used, and underlying modelling assumptions will be checked. If differences between baseline characteristics exist between the three treatment groups in this comparison, it will be investigated if adjustment for these characteristics is clinically relevant and necessary. The secondary outcome measures will be compared using similar models.<br>Subjects who are randomized to receive vamorolone 2.0 mg/kg/day or vamorolone 6.0 mg/kg/day for both treatment periods will have TTSTAND velocity change from baseline data captured over 48 weeks compared with untreated DMD historical control data. Full details will be provided in the Statistical Analysis Plan (SAP).<br>**Patient Reported Outcome Analyses:**<br>Patient Reported Outcomes including the TSQM, PODCI, PARS III, Ease of Study Medication Administration Assessment, and the Blindedness Assessment will be listed and presented using descriptive statistics by treatment and time point. In addition, comparison will be made for each vamorolone dose group with placebo for the PODCI and for each vamorolone dose group with prednisone for the PARS III, with p values computed for the comparisons. Full details will be provided in the SAP.<br>**Safety Analyses:**<br>All evaluations of clinical safety will be listed and presented using descriptive statistics per treatment group and time point. For the safety analyses, the vamorolone dose levels will be compared to prednisone.<br>Safety data will include BMI, weight, vital signs, 2D-echocardiogram, DXA scan, spine x-ray, eye examination results, ACTH Stimulation Test results, and ECG results, and these will be presented using descriptive statistics. Safety laboratory data will be summarized using descriptive statistics, and out-of-range values will be listed. Adverse events will be summarized overall and by treatment group, system organ class (SOC) and preferred term (using the |

| | |
|---|---|
| | Medical Dictionary for Regulatory Activities [MedDRA]); by treatment group and relationship to study medication; and by treatment group and intensity (CTCAE grade).<br>In Treatment Period #1, BMI z-score change from baseline data captured over 24 weeks will be compared for subjects who are randomized to receive vamorolone 2.0 mg/kg/day or vamorolone 6.0 mg/kg/day vs. subjects randomized to receive prednisone. The same MMRM that is used for the primary efficacy analysis will be used for BMI z-score. The test for statistical significance will be performed at the 0.05 level. Subjects who are randomized to receive vamorolone 2.0 mg/kg/day or vamorolone 6.0 mg/kg/day for both treatment periods will have their BMI z-score change from baseline data captured over 48 weeks compared with prednisone-treated DMD historical control data. Full details will be provided in the SAP.<br>**Pharmacodynamics Analyses:**<br>Serum PD biomarkers of adrenal axis suppression, insulin resistance, bone turnover, and immune suppression will be assessed. PD biomarkers will be analyzed using MMRMs similar to the primary efficacy model. Plots will be created. Additional exploratory PD biomarkers of both safety and efficacy may be assessed. Vamorolone-treated groups will be compared to both prednisone-treated and placebo groups.<br>**Pharmacokinetic Analyses:**<br>The 2-hr post-dose plasma concentration measurements of vamorolone at Week 30 will be used for comparison of drug exposures between the two dosing groups. They will be added to PK data from previous studies in DMD boys for comparison with measurements obtained in healthy adult male subjects. All PK data will be combined in a population assessment of plasma concentrations in relation to dose and age of subjects. |

# TABLE OF CONTENTS


**SERIOUS ADVERSE EVENT CONTACT INFORMATION .....4**

**PROTOCOL AMENDMENT TRACKING.....5**

**STUDY SYNOPSIS .....7**

**LIST OF TABLES.....28**

**LIST OF FIGURES.....29**

**LIST OF ABBREVIATIONS .....30**

**1 INTRODUCTION.....34**

**1.1 BACKGROUND AND UNMET NEED.....34**

**1.2 NONCLINICAL EXPERIENCE .....36**

**1.2.1 Safety Pharmacology .....37**

**1.2.2 Pharmacokinetics and Metabolism .....38**

**1.2.3 Toxicology.....42**

**1.3 CLINICAL EXPERIENCE .....55**

**1.3.1 Phase I Study in Healthy Adult Male Volunteers (VBP15-001).....55**

**1.3.2 Pharmacokinetics in Phase IIa Study in 4 to 7 years Duchenne Muscular Dystrophy Boys (VBP15-002).....67**

**1.3.3 Safety in Phase II Studies in 4 to7 years Duchenne Muscular Dystrophy Boys (VBP15-002 and VBP15-003).....70**

**1.4 RATIONALE FOR STUDY DESIGN.....72**

**1.5 OVERALL BENEFIT/RISK.....76**

**2 STUDY OBJECTIVES AND ENDPOINTS.....77**

**2.1 STUDY OBJECTIVES.....77**

**2.1.1 Primary Objectives .....77**

2.1.2 Secondary Objectives....77
2.1.3 Exploratory Objectives....78
2.2 Study Endpoints....79
2.2.1 Safety Endpoints....79
2.2.2 Clinical Efficacy Endpoints....81
2.2.3 Exploratory Endpoints....82
2.2.4 Pharmacodynamic Endpoints....82
2.2.5 Endpoints for Patient-Reported Outcomes....83
3 STUDY DESIGN....84
3.1 Overall Study Design....84
3.2 Randomization....87
3.3 Blinding....88
3.4 Unblinding....89
4 SELECTION AND WITHDRAWAL OF STUDY SUBJECTS....91
4.1 Subject Enrollment and Identification Log....91
4.2 Inclusion Criteria....92
4.3 Exclusion Criteria....93
4.4 Withdrawal of Subjects from Study....95
4.5 Termination of Study....96
5 TREATMENT OF STUDY SUBJECTS....97
5.1 Study Medications Administered....97
5.1.1 Study Medications Administered During Treatment Period #1....97
5.1.2 Study Medications Administered During Transition Period....98

5.1.3 Study Medications Administered During Treatment Period #2 and the Dose-tapering Period ....99
5.2 IDENTITY OF INVESTIGATIONAL PRODUCT ....100
5.3 DOSAGE SCHEDULE AND ADMINISTRATION OF STUDY MEDICATION ....101
5.4 RATIONALE FOR DOSE SELECTION ....104
5.5 TREATMENT COMPLIANCE ....105
5.6 STUDY DRUG DOSE INTERRUPTION OR DISCONTINUATION ....106
5.7 PRIOR AND CONCOMITANT MEDICATIONS AND THERAPIES ....107
5.7.1 Prior Therapy ....107
5.7.2 Concomitant Therapies ....107
5.7.3 Prohibited Therapies ....107
5.7.4 Permitted Therapies ....109
5.7.5 Hydrocortisone ....109
5.7.6 Vitamin D ....109
5.8 STUDY MEDICATION MANAGEMENT ....109
5.8.1 Packaging and Labeling of Study Medication ....109
5.8.2 Storage of Study Medication ....111
5.8.3 Study Medication Shipping and Handling ....112
5.8.4 Study Medication Accountability ....113
5.9 PROCEDURES FOR ASSIGNING SUBJECT STUDY NUMBERS ....114
6 STUDY SCHEDULE ....114
6.1 TIME AND EVENTS SCHEDULE ....114
6.2 INFORMED CONSENT AND ASSENT PROCEDURES ....122
6.2.1 HIPAA and Protected Health Information ....123

6.3 VISIT SCHEDULE AND PROCEDURES ....................123
6.3.1 Screening Period (Day -33 to -2)....................124
6.3.2 Baseline Period (Day -1) Visit....................126
6.3.3 Treatment Period #1 Day 1 Visit....................127
6.3.4 Treatment Period #1 (Weeks 1-24)....................129
6.3.5 Transition Period (Weeks 25-28)....................131
6.3.6 Treatment Period #2 (Week 28 + 1 Day through Week 48)....................133
6.3.7 Dose-tapering Period (Weeks 49-52)....................136
6.4 SUBJECT DISCONTINUATION....................137
6.4.1 Early Discontinuation Prior to Week 24....................139
6.4.2 Early Discontinuation After Week 24 and Prior to Week 28 ....................140
6.4.3 Early Discontinuation After Week 28 and Prior to Week 48 ....................140
6.5 SUBJECT AND STUDY COMPLETION....................141
7 STUDY ASSESSMENTS AND MEASUREMENTS ....................141
7.1 DEMOGRAPHIC ASSESSMENTS ....................141
7.1.1 Genetic Modifiers of DMD....................141
7.2 SAFETY AND TOLERABILITY ASSESSMENTS ....................141
7.2.1 Medical History....................141
7.2.2 Physical Examination, Cushingoid Features, Weight, and Height ...142
7.2.3 Vital Signs....................142
7.2.4 Clinical Laboratory Tests....................143
7.2.5 Chicken Pox Immunity....................147
7.2.6 Pharmacodynamic Biomarker Panel....................147
7.2.7 ACTH Stimulation Test....................148

7.2.8 Population PK Assessment ....150
7.2.9 Total Blood Volume Required ....150
7.2.10 12-Lead ECG ....152
7.2.11 2D-echocardiography ....152
7.2.12 Eye Examination ....153
7.2.13 Bone Health and DXA Scan (total body and spine) ....153
7.2.14 Spine X-rays ....154
7.3 Assessment of Muscle Strength and Function ....154
7.3.1 Time to Stand Test (TTSTAND) ....154
7.3.2 Time to Climb Test (TTCLIMB) ....155
7.3.3 Time to Run/Walk Test (TTRW) ....155
7.3.4 North Star Ambulatory Assessment (NSAA) ....155
7.3.5 Six-minute Walk Test (6MWT) ....156
7.3.6 Hand-Held Myometry (elbow flexors and knee extensors) ....156
7.3.7 Range of Motion (ROM) ....156
7.4 Patient-Reported Outcome Measures ....157
7.4.1 Pediatric Outcomes Data Collection Instrument (PODCI) ....157
7.4.2 Treatment Satisfaction Questionnaire (TSQM) ....157
7.4.3 Behavioral Assessment ....157
7.4.4 Ease of Study Medication Administration Assessment ....158
7.4.5 Blindedness Assessment ....158
7.4.6 Subject Diary ....158
7.5 Adverse Events and Serious Adverse Events ....159
7.5.1 Intensity ....160

7.5.2 Relationship ....160
7.5.3 Clinical Laboratory Test Abnormalities ....162
7.5.4 Follow-Up of Adverse Events ....163
7.5.5 Dosing Error ....163
7.5.6 Serious Adverse Events ....164
8 STUDY COMMITTEES ....167
8.1 STUDY STEERING COMMITTEE ....167
8.2 DATA AND SAFETY MONITORING BOARD ....167
9 DATA COLLECTION ....168
9.1 SOURCE DOCUMENTS ....168
9.2 ELECTRONIC CASE REPORT FORM COMPLETION ....168
9.3 DATA PROCESSING ....169
9.4 SUBJECT DIARIES ....169
10 STATISTICAL METHODS AND PLANNED ANALYSES ....169
10.1 SAMPLE SIZE DETERMINATION ....169
10.2 STATISTICAL AND ANALYTICAL PLAN (SAP) ....170
10.2.1 Deviations from the Statistical Analysis Plan ....171
10.3 ANALYSIS POPULATIONS ....171
10.3.1 Safety Population ....171
10.3.2 Modified Intent-to-Treat (mITT) Population ....171
10.3.3 Pharmacokinetic (PK) Population ....171
10.4 MEASURES TAKEN TO AVOID/MINIMIZE BIAS ....171
10.5 INTERIM ANALYSIS ....171
10.6 WEEK 24 ANALYSIS ....172

**10.7 WEEK 48 ANALYSIS ........ 172**

**10.8 MISSING, UNUSED, AND SPURIOUS DATA ........ 172**

**10.9 STATISTICAL ANALYSIS ........ 172**

**10.9.1 General Considerations ........ 172**

**10.9.2 Adjustment for Multiple Comparisons ........ 174**

**10.9.3 Subject Disposition, Demographics, and Baseline Characteristics ... 175**

**10.9.4 Efficacy Analyses ........ 175**

**10.9.5 Safety Analyses ........ 177**

**10.9.6 Pharmacodynamic Analyses ........ 178**

**10.9.7 Patient-Reported Outcome Exploratory Analyses ........ 178**

**10.9.8 Pharmacokinetic Analyses ........ 178**

**10.9.9 Concurrent Medications ........ 179**

**11 STUDY MANAGEMENT AND ETHICAL AND REGULATORY REQUIREMENTS ........ 179**

**11.1 REGULATORY APPROVAL AND GOOD CLINICAL PRACTICE ........ 179**

**11.2 INVESTIGATOR RESPONSIBILITIES ........ 179**

**11.2.1 Subject Information and Informed Consent ........ 179**

**11.2.2 Institutional Review Board/Independent Ethics Committee Approval and Other Institutional Requirements ........ 180**

**11.2.3 Study Documentation ........ 181**

**11.2.4 Delegation of Investigator Responsibilities ........ 183**

**11.3 PROTOCOL DEVIATIONS ........ 183**

**11.3.1 Protocol Deviation Definitions ........ 183**

**11.3.2 Reporting Important Protocol Deviations ........ 184**

11.4 STUDY RECORDS RETENTION AND DIRECT ACCESS TO SOURCE DOCUMENTS ....184

11.5 STUDY MONITORING ....185

11.6 QUALITY ASSURANCE ....186

11.7 STUDY TERMINATION AND SITE CLOSURE ....187

11.8 SITE TERMINATION ....187

11.9 DISCONTINUATION OF STUDY ....188

12 DISCLOSURE OF DATA ....188

12.1 CONFIDENTIALITY ....188

12.2 PUBLICATION ....189

13 INVESTIGATOR PROTOCOL AGREEMENT ....190

14 REFERENCES ....191

15 APPENDICES ....196

APPENDIX 15.1 PROTOCOL AMENDMENT #2 COMPLETE LIST OF CHANGES ....197

# LIST OF TABLES

**Table 1.** **$AUC_{0\text{-}24hr}$ in Mice after 179 Days Treatment with 3 Dose Levels (5, 15 and 45 mg/kg/day)....48**

**Table 2.** **Summary of Pharmacokinetic Parameters for Vamorolone after Oral Administration of Single Doses of 0.1, 0.3, 1.0, 3.0, 8.0, and 20.0 mg/kg to Healthy Subjects Under Fasted Conditions....56**

**Table 3.** **Summary of Pharmacokinetic Parameters for Vamorolone After Single Dose Oral Administration of 8 mg/kg to Healthy Subjects Under Fed and Fasted Conditions....58**

**Table 4.** **Summary of Pharmacokinetic Parameters for Vamorolone During Oral Administration of 1, 3, 9, and 20 mg/kg Doses Once Daily for 14 Days to Healthy Subjects Under Fasted Conditions....60**

**Table 5.** **Summary of Pharmacokinetic Parameters for Vamorolone after Once Daily Oral Administration of 0.25, 0.75, 2.0 and 6.0 mg/kg Doses to DMD Boys....69**

**Table 6.** **Study Randomization Schedule....73**

**Table 7.** **Study Medications for the Six Treatment Groups During Treatment Period #1....97**

**Table 8.** **Study Medications for the Six Treatment Groups During the Transition Period....98**

**Table 9.** **Study Medications for the Six Treatment Groups During Treatment Period #2 and the Dose-tapering Period....99**

**Table 10** **Weight Bands for Prednisone or Matching Tablet Dosing....101**

**Table 11** **Schedule of Study Activities....117**

**Table 12.** **Tablet Dose Tapering....132**

**Table 13.** **Suspension Dose Tapering....136**

**Table 14.** **Tablet Dose Tapering for Subjects Discontinuing Prior to Week 24....139**

**Table 15.** **Suspension Dose Tapering for Subjects Discontinuing Prior to Week 24....140**

**Table 16.** **Suspension Dose-Tapering for Subjects Discontinuing After Week 24 and Prior to Week 28....140**

**Table 17.** **Hematology, Chemistry, and Lipids Clinical Laboratory Tests....145**

**Table 18.** **Urinalysis Clinical Laboratory Tests....146**

**Table 19.** **Pharmacodynamic Biomarkers – Secondary Safety Outcomes....148**

**Table 20.** **Blood Sample Number and Volume by Study Visit....151**

# LIST OF FIGURES

**Figure 1 Liver Function Enzymes in GLP Chronic Toxicology Study in Mice (26 weeks)....49**

**Figure 2. Plasma Concentrations of Vamorolone (VBP15) after Oral Administration of Single Doses of 0.1, 0.3, 1, 3, 8, and 20 mg/kg to Healthy Subjects Under Fasted Conditions....56**

**Figure 3. Relationship Between Individual Subject Vamorolone AUC($_{Inf}$) and Dose After Oral Administration of Single Doses of 0.1, 0.3, 1, 3, 8, and 20 mg/kg to Healthy Subjects Under Fasted Conditions....57**

**Figure 4. Plasma Concentrations of Vamorolone (VBP15) After Single Dose Oral Administration of 8 mg/kg to Healthy Subjects Under Fed and Fasted Conditions....58**

**Figure 5. Plasma Concentrations of Vamorolone (VBP15) on Days 1 and 14 During Oral Administration of 1, 3, 9, and 20 mg/kg Doses Once Daily for 14 Days to Healthy Subjects Under Fasted Conditions....61**

**Figure 6. Morning Cortisol Measurements in the Vamorolone Phase I Healthy Subjects....64**

**Figure 7. Fasting Serum Glucose During the Phase I MAD Period (Two Weeks Daily Treatment)....65**

**Figure 8 Mean Plasma Concentrations of Vamorolone after Once Daily Oral Administration of 0.25, 0.75, 2.0, and 6.0 mg/kg Doses....68**

**Figure 9 Regression Analysis of $C_{max}$ and $AUC_{inf}$ versus Dose....70**

# LIST OF ABBREVIATIONS

| Abbreviation | Definition/Term |
|---|---|
| %CV | percent coefficient of variation |
| ACTH | adrenocorticotropic hormone |
| ADL | activities of daily living |
| AE | adverse event |
| ALP | alkaline phosphatase |
| ALT | alanine aminotransferase |
| AST | aspartate aminotransferase |
| AUC | area under the concentration-time curve |
| $AUC_{0\text{-}24hr}$ | area under the concentration-time curve from time 0 to 24 hours |
| $AUC_{(0\text{-}t)}$ | area under the concentration-time curve from time 0 to time t |
| $AUC_{(inf)}$ | area under the concentration-time curve from time 0 to infinity |
| $AUC_{last}$ | area under the plasma concentration-time curve from time 0 to the last observed measurable concentration |
| BMI | body mass index |
| BUN | blood urea nitrogen |
| C | Celsius |
| CFR | Code of Federal Regulations |
| CINRG | Cooperative International Neuromuscular Research Group |
| CK | creatine kinase |
| CL | clearance |
| ConA | Concanavalin A |
| cm | centimeter |
| $C_{max}$ | maximum observed plasma concentration |
| CTCAE | Common Terminology Criteria for Adverse Events |
| CTM | Clinical Trial Material |
| CTMS | Clinical Trial Management Software |
| CTX1 | C-terminal peptide fragment of collagen 1 |
| CYP | cytochrome P450 |
| dL | deciliter |
| DMD | Duchenne muscular dystrophy |
| DNA | deoxyribonucleic acid |
| DSMB | Data and Safety Monitoring Board |
| DXA | dual-energy x-ray absorptiometry |

| Abbreviation | Definition/Term |
|---|---|
| ECG | electrocardiogram |
| eCRF | electronic case report form |
| EDC | electronic data capture |
| EMA | European Medicines Agency |
| EU | European Union |
| F% | percent bioavailability |
| FDA | Food and Drug Administration |
| GALT | gut-associated lymphoid tissue |
| GCP | Good Clinical Practice |
| GGT | gamma glutamyl transferase |
| GLDH | glutamate dehydrogenase |
| GLP | Good Laboratory Practice |
| HbA1c | hemoglobin A1c |
| HDL | high density lipoprotein |
| HEENT | head, eyes, ears, nose and throat |
| HIPAA | Health Insurance Portability and Accountability Act |
| hr | hour |
| ICF | Informed Consent Form |
| ICH | International Conference on Harmonisation |
| ID | identification |
| IEC | Independent Ethics Committee |
| IND | Investigational New Drug |
| IRB | Institutional Review Board |
| IXRS | Interactive Voice/Web Response System |
| kg | kilogram |
| L | liter |
| LLC | Limited Liability Company |
| LDH | lactate dehydrogenase |
| LDL | low density lipoprotein |
| LS | least squares |
| µg | microgram |
| m | meter |
| $m^2$ | square meter |
| MAD | multiple ascending dose (study) |
| MCMC | Markov Chain Monte Carlo |

| Abbreviation | Definition/Term |
|---|---|
| MD | Medical Doctor (physician) |
| *Mdx* | mouse model lacking dystrophin |
| MedDRA | Medical Dictionary for Regulatory Activities |
| mg | milligram |
| Min | minute |
| mITT | modified Intent-to-Treat |
| mL | milliliter |
| MMRM | mixed model for repeated measures |
| MTD | maximum tolerated dose |
| N, No. | number |
| NADPH | nicotinamide adenine dinucleotide phosphate |
| NCA | non-compartmental analysis |
| NF-κB | nuclear factor kappa-light-chain-enhancer of activated B cells |
| ng | nanogram |
| nM | nanomolar |
| nmol | nanomole |
| NOAEL | no observed adverse effect level |
| NSAA | North Star Ambulatory Assessment |
| OTC | over-the-counter (non-prescription medication) |
| oz | ounce |
| P1NP | serum aminoterminal propeptide of type I collagen |
| PARS III | Personal Adjustment and Role Skills Scale III |
| PBL | peripheral blood leukocytes |
| PD | pharmacodynamics |
| PHI | Protected Health Information |
| PK | pharmacokinetics |
| PODCI | Pediatric Outcomes Data Collection Instrument |
| PR [PQ] | time from onset of P wave to start of the QRS complex |
| QD | once daily (dosing) |
| QRS | in electrocardiography, the complex consisting of Q, R, and S waves, corresponding to depolarization of ventricles [complex] |
| QSAR | quantitative structure-activity relationship |
| QT | in cardiology, the time between the start of the Q wave and end of the T wave |
| $QT_c$ | corrected QT interval |
| RBC | Red Blood Cell |

| Abbreviation | Definition/Term |
|---|---|
| REML | restricted maximum likelihood |
| ROM | Range of Motion |
| RR | in electrocardiography, the interval between successive Rs (peaks of QRS complexes) |
| 6MWT | Six-minute Walk Test |
| SAD | single ascending dose (study) |
| SAE | serious adverse event |
| SAP | statistical analysis plan |
| SD | standard deviation |
| SOC | system organ class |
| SOP | standard operating procedures |
| SSC | Study Steering Committee |
| SUSAR | Suspected Unexpected Serious Adverse Reaction |
| $t_{1/2}$ | terminal half-life |
| TEAE | treatment-emergent adverse event |
| $T_{max}$ | time to maximum observed plasma concentration |
| TRiNDS | Therapeutic Research in Neuromuscular Disorders Solutions |
| TSQM | Treatment Satisfaction Questionnaire for Medication |
| TTCLIMB | Time to Climb (Test) |
| TTSTAND | Time to Stand (Test) |
| TTRW | Time to Run/Walk (Test) |
| ULN | upper limit of normal |
| US | United States |
| vol | volume |
| vs. | versus |
| $V_{ss}$ | volume of distribution at steady state |
| WBC | White Blood Cell |
| WHO | World Health Organization |
| wt | weight |

# 1 INTRODUCTION

## 1.1 Background and Unmet Need

Duchenne muscular dystrophy (DMD) is a rapidly progressive form of muscular dystrophy that occurs primarily in males and manifests prior to the age of six years. Duchenne muscular dystrophy affects approximately 1 in 3,600 to 9,300 male births worldwide.[1] Duchenne muscular dystrophy is caused by mutations in the dystrophin gene which codes for a protein that provides structural stability to the dystroglycan complex on muscle cell membranes.[2] The lack of dystrophin reduces plasma membrane stability. Membrane destabilization results in altered mechanical properties and aberrant signaling, which contribute to membrane fragility, necrosis, inflammation, and progressive muscle wasting.[3]

In addition to the significant contribution of membrane destabilization and mechanical injury in DMD, aberrant intracellular signaling cascades that regulate inflammatory and immune processes also contribute to DMD pathophysiology. Up-regulated inflammatory gene expression and activated immune cell infiltrates, at least partially mediated by nuclear factor kappa-light-chain-enhancer of activated B cells (NF-κB) activation, are evident during early disease stages and play a significant role in muscle wasting.[3] NF-κB has been shown to regulate the expression of numerous inflammatory genes in immune cells and muscle fibers,[4,5,6,7] and the infiltration and activation of these cells can trigger muscle fiber death.[8,9]

Although significant advances have been made in understanding the etiology of DMD, a cure has not been found, and until recently treatment options were medications used "off-label" to alleviate the symptoms of DMD. Despite scientific advances, only glucocorticoids, such as prednisone or deflazacort, have consistently demonstrated efficacy in clinical trials.[10] Indeed, the United States Food and Drug Administration (US FDA) recently approved deflazacort as a treatment for DMD. Further, many disease modifying technologies that are currently in development focus on subsets of dystrophin mutations and therefore do not address the unmet need in all persons with DMD. However, it is likely that glucocorticoids will need to be co-administered with many of

these compounds for maximum effect and glucocorticoids have extensive side effect profiles, often limiting long-term administration. The current goal of DMD research is to find a mutation-independent treatment that matches or exceeds the efficacy of glucocorticoids with a significantly lower side effect profile.

Vamorolone is a first generation delta-9, 11 chemical compound belonging to the structural class of synthetic steroidal drugs, which includes the glucocorticoids prednisone, prednisolone, methylprednisolone, and dexamethasone.[11] The chemical structure of vamorolone has optimized four subactivities of traditional glucocorticoid drugs, namely transactivation, transrepression, physiochemical membrane properties, and mineralocorticoid receptor antagonism.[11] By reducing transactivation subproperties, retaining transrepression, imparting membrane stabilizing properties, and inhibiting the mineralocorticoid receptor pathway, vamorolone has favorable efficacy and side effect profiles relative to classic glucocorticoids in nonclinical models and is anticipated to be an attractive candidate for the treatment of DMD in pediatric patients.

*In vitro* and nonclinical data to date suggest that vamorolone may offer a much needed alternative to the current glucocorticoids which are standard of care for DMD[12] with administration beginning around the age of 5 years in most developed countries, or even earlier in some cases.

The significant effects of glucocorticoids on growth and development, however, prevent their routine administration in infancy or 'toddler' years, despite evidence that the earlier the administration, the better the overall functional outcome.[13] The cumulative adverse effects of glucocorticoids, including excess weight, delayed puberty, fragile skin, loss of bone mineral density, bruising, and cushingoid appearance continue to negatively impact on the quality of life of the individual, leading to significant variations in clinical practice.[14] Glucocorticoids also contribute to further muscle damage with long-term administration. Vamorolone has shown few if any of the adverse effects of traditional glucocorticoids in mouse models of DMD.[11,15,16]

This study is targeted to explore whether vamorolone will show at least equal efficacy to glucocorticoids with a more favorable adverse effect profile, thereby improving the

quality of life for DMD patients. This profile would enable use of vamorolone in DMD boys at a younger age than when glucocorticoid treatment is currently initiated. In addition, vamorolone could be prescribed in later stage non-ambulant young men with DMD and for a longer period of time, where the risk:benefit balance of glucocorticoids is often less favorable.

Efficacy may also be improved over classic glucocorticoids in the longer term. In addition to the anti-inflammatory properties of vamorolone as a result of NF-κB pathway inhibition, vamorolone may also improve efficacy over conventional glucocorticoids due to the lack of interference in the AKT1/FOXO pathway, a key feature of glucocorticoid therapy which leads in the long term to muscle wasting and atrophy.[17] Further, vamorolone has been recently demonstrated to improve asynchronous remodeling, believed to be a component of progressive muscle weakness and wasting in DMD[18] and may also prevent muscle membrane damage, thereby delaying progression of the disease further. Vamorolone is an antagonist to the mineralocorticoid receptor, whereas glucocorticoids are typically agonists. An antagonist for the mineralocorticoid receptor, epleronone, was recently shown to significantly improve DMD heart function.[19] Finally, vamorolone imparts physical stability to myofiber plasma membranes, whereas prednisolone destabilizes membranes. This property addresses the primary defect of membrane instability in dystrophin deficient myofibers in DMD.[15]

Potentially, the administration of vamorolone to a DMD patient may begin soon after birth to slow the dystrophic process of muscle, retaining regenerative capacity and substantially improving patient quality of life.

## 1.2 Nonclinical Experience

The safety pharmacology, pharmacokinetics (PK) and metabolism, and toxicology of vamorolone have been evaluated in multiple nonclinical studies *in vitro* and in mice, rats, beagle dogs, and cynomolgus monkeys *in vivo*.

All Good Clinical Laboratory (GLP) studies were conducted in, or inspected by, a country that has implemented the Organisation for Economic Cooperation and Development (OECD) Mutual Acceptance of Data (MAD) system.

### 1.2.1 Safety Pharmacology

Stunted growth is a significant side effect of chronic glucocorticoid use in children.[20,21] Chronic treatment with glucocorticoids negatively affects bone growth and development and can cause osteoporosis.[22,23]

The effect of vamorolone as compared to prednisolone on bone growth and development was evaluated in the *mdx* mouse model of DMD that lacks dystrophin due to a premature chain-terminating mutation in the mouse homologue of the dystrophin gene. In the pre-symptomatic *mdx* study, tibia length was measured to determine if vamorolone inhibited bone growth. Prednisolone significantly decreased tibia length whereas vamorolone did not affect tibia length at any concentration tested. Micro-computed tomography was performed on femurs to examine bone density and structure. Comparison of vehicle, prednisolone, and the highest vamorolone dose showed prednisolone to significantly reduce trabecular thickness compared to vehicle, while vamorolone did not.[15]

In normal, male CD-1® mice, these effects were reproduced. Unlike CD-1 mice treated with prednisolone, CD-1 mice receiving vamorolone did not experience tibia length shortening.[16] However, at the highest vamorolone dose tested, mice did have significantly reduced body length, though to a lesser extent as compared to prednisolone.

Duchenne muscular dystrophy is associated with cardiomyopathy that can become life threatening, and increased fibrosis with prednisolone treatment in heart muscle of the *mdx* mouse has been reported.[24] Histologically, clear fibrosis was evident in 50% of young (8-week) prednisolone-treated mouse hearts compared to no incidence of fibrosis identified in the other groups (wild type; *mdx* vehicle, and vamorolone-treated).

Pharmacologically, glucocorticoids show immunosuppressive and immunotoxic properties that limit therapeutic windows and long-term use. Vamorolone (5, 15, 30 mg/kg/day) was benchmarked against prednisolone (5 mg/kg/day) to determine if similar properties were observed.[15] Untreated *mdx* mice showed increased numbers of peripheral blood leukocytes (PBL) and enlarged spleens resulting from ongoing muscle damage compared to wild type mice. Vamorolone treatment reduced spleen mass and PBL counts in a dose-dependent manner. This finding is attributed to a reduction in

muscle damage by vamorolone that decreases spleen size to levels resembling those in wild type mice. Prednisolone reduced these measures below wild type, suggesting immunosuppressive and/or immunotoxic properties. Further, prednisolone significantly decreased viable splenocytes per gram of tissue ($p<0.005$), whereas this decrease was not observed for any vamorolone dose tested (ReveraGen Report No. MDX-RBP-VBP15-02).[15]

To further query the potential immune modulation, the effects of vamorolone and prednisolone on counts of splenic B and T-lymphocytes isolated from treated *mdx* mice were examined. CD4+ T-cell activation was assayed by stimulation of splenocytes with the T-cell mitogen, concanavalin A (ConA). Splenocytes obtained from prednisolone-treated mice displayed a significant reduction of the percentage of splenic activated CD4+CD25+ T-cells upon ConA stimulation while splenocytes derived from vamorolone-treated mice did not (ReveraGen Report No. MDX-RBP-VBP15-02).

Taken together, these findings suggest that while prednisolone treatment leads to a reduction in T-cell number and activation status, vamorolone modulates inflamed *mdx* immune systems towards a wild type state without compromising T-cell activation status.

### *1.2.2 Pharmacokinetics and Metabolism*

#### *Single Dose*

Vamorolone PK profiles were determined in male CD-1 mice, Sprague Dawley rats and beagle dogs after a single intravenous injection of 10 mg/kg and after a single oral dose of 50 mg/kg in mice and rats and 30 mg/kg in dogs.

Pharmacokinetic results for vamorolone following a single intravenous administration of 10 mg/kg in Crl:CD1(ICR) mice demonstrated a clearance (CL) of 18.8 mL/min/kg. The terminal half-life ($t_{½}$) was 0.35 hours. Volume of distribution at steady state ($V_{ss}$) was 0.76 L/kg. Following oral administration of 50 mg/kg in mice, the maximum observed plasma concentration ($C_{max}$) of 6787 ng/mL was observed at 2 hours (time to maximum observed plasma concentration [$T_{max}$]) after drug administration, and percent bioavailability (F%) was 74.5%. Following oral administration of 15 mg/kg via cherry

syrup, the $C_{max}$ of 1527 ng/mL was observed at 2 hours after drug administration and bioavailability was 47.7% (ReveraGen Report No. PH-DPMK-VBP-10-004).

Pharmacokinetic results for vamorolone following a single intravenous administration of 50 mg/kg in Sprague Dawley rats indicated a CL of 20.2 mL/min/kg. The $t_{½}$ was 0.58 hours. $V_{ss}$ was 0.77 L/kg, which was similar to that observed in mice. After oral administration of 50 mg/kg in rats, a $C_{max}$ of 2543 ng/mL was observed at 4 hours after dose administration, and bioavailability was 47.8% (ReveraGen Report No. PH-DPMK-VBP-10-007).

In beagle dogs, vamorolone had a CL of 24.7 mL/min/kg. The $t_{½}$ was 5.42 hours and $V_{ss}$ was 1.93 L/kg. After oral administration of 30 mg/kg in dogs, a $C_{max}$ of 814 ng/mL was observed at 6 hours after dose administration and bioavailability was 53.2% (ReveraGen Report No. 48504-10-464).

Vamorolone clearance was therefore comparable in all 3 species studied (19-25 mL/min/kg). Bioavailability ranged from approximately 50% in mouse (cherry syrup), rat, and dog to 75% in the mouse (30% Labrafil) (ReveraGen Report Nos. PH-DPMK-VBP-10-004, PH-DPMK-VBP-10-007, 48504-10-464).

***Multiple Dose***

Crl:CD1(ICR) mice were administered vamorolone or vehicle once daily (QD) for 28 consecutive days. Vamorolone exposure (as assessed by the $C_{max}$ and area under the plasma concentration-time curve from time 0 to the last observed measurable concentration [$AUC_{last}$]) increased with increasing dose on Study Days 1 and 28. Repeated dosing of vamorolone over a 28-day duration was associated with decreases in mean vamorolone $AUC_{last}$ values in the 30 and 100 mg/kg dose groups compared to Day 1, indicating possible enzyme induction. On Study Day 28, mean $AUC_{last}$ values were 1.81-fold and 5.02-fold lower compared to Study Day 1 for the 30 and 100 mg/kg dose groups, respectively. The observed difference in exposure relative to Day 1 increased with the increase in administered dose of vamorolone (ReveraGen Report No. 1998-009).

Beagle dogs were either administered vamorolone or vehicle QD for 28 consecutive days. Vamorolone exposure in dogs (as assessed by $C_{max}$ and $AUC_{last}$) generally increased with increasing dose on Study Days 1 and 28. For the 2 and 10 mg/kg dose groups, exposure on Day 28 was generally higher than on Day 1, indicating possible inhibition of metabolism of vamorolone at these dose levels. On Day 28, mean $AUC_{last}$ values were 2.35-fold and 2.43-fold (males) and 3.03-fold and 3.23-fold (females) higher compared to Study Day 1 for the 2 and 10 mg/kg/day dose groups, respectively. For the 50 mg/kg dose group, exposure on Day 28 was similar to that on Day 1. At the 50 mg/kg dose, $AUC_{last}$ values in males were 1.71-fold lower whereas females were 1.22 higher on Day 28 compared to Day 1 (ReveraGen Report No. 031302).

Beagle dogs were administered vehicle or vamorolone at doses of 2 mg/kg/day, 10 mg/kg/day, or 50 mg/kg/day for 39 weeks. Systemic exposure (area under the plasma concentration-time curve from time 0 to 24 hours [$AUC_{0\text{-}24hr}$]) to vamorolone appeared to be independent of sex. Mean $AUC_{0\text{-}24hr}$ and $C_{max}$ values for vamorolone increased with increasing dose in an approximately dose proportional manner on Days 1 and 270. Mean systemic exposure ($AUC_{0\text{-}24hr}$) to vamorolone appeared to increase following repeated administration of vamorolone. Due to the alterations in the feeding regimen, changes in systemic exposure following repeated administration should be viewed with caution due the influence of feeding on exposure. For the 2 and 10 mg/kg/day dose groups, exposure on Day 270 was generally higher than on Day 1, indicating possible inhibition of metabolism of vamorolone at these dose levels. On Day 270, mean $AUC_{0\text{-}24hr}$ values were 2.34-fold and 2.98-fold higher compared to Study Day 1 for the 2 and 10 mg/kg/day dose groups, respectively. For the 50 mg/kg dose group, exposure on Day 270 was 2.07-fold higher compared to that on Day 1 (ReveraGen Report No. 1998-014).

Non-naïve cynomolgus monkeys were administered vamorolone (300 and 600 mg/kg/day) or vehicle once daily for 7 consecutive days. Vamorolone exposure (as assessed by $C_{max}$ and $AUC_{last}$) generally increased with increasing dose on Study Days 1 and 7 with the exception of male monkeys on Day 7, which showed no clear increase in exposure between the 300 and 600 mg/kg/day dose levels. Repeated dosing over the

7-day study duration was associated with decreases in mean plasma vamorolone $AUC_{last}$ values for female and male monkeys indicating possible metabolic induction. On Day 7, mean $AUC_{last}$ values were 1.60-fold, 2.19-fold, and 2.02-fold lower in females and 1.20-fold, 2.09-fold, and 2.88-fold lower in males compared to Study Day 1 for the 100, 300 and 600 mg/kg/day dose groups, respectively (ReveraGen Report Nos. 1998-001, SW11-0418).

***Distribution***

In the plasma protein binding studies, percent bound was similar in human and mouse cells in culture (88.06% and 86.71%, respectively). In the blood partition experiment done *ex vivo*, the blood to plasma ratio was similar between human and mouse (0.87 and 0.68, respectively), but the red blood cell to plasma ratio for the mouse (0.33) was less than half that of the human (0.74). Human *in vivo* data are presented in **Section 1.3** (VBP15-001). In the blood/brain concentration mouse experiment *in vivo*, the plasma concentrations of vamorolone were higher than brain concentrations with the area under the plasma concentration-time curve (AUC) and $C_{max}$ approximately 2-fold higher in plasma than in brain (ReveraGen Report Nos. ADME-NCG-PPB-NC135, ADME-VBP-PPB-V002, ADME-NCG-BP-NC134, NCATS 2013-38).

***Metabolism***

The *in vitro* and *in vivo* data demonstrate that vamorolone can be metabolized via multiple metabolic pathways, including glucuronidation, hydroxylation, and reduction. Glucuronidation appeared to be the major metabolic pathway in human cells *in vitro*. All metabolites observed in human *in vitro* were observed in monkey *in vitro*. Most human metabolites identified *in vitro* were also found in mouse and dog. Thus, there is no unique human metabolite identified for vamorolone.

The metabolic stability of vamorolone was assessed in non-Good Laboratory Practice (GLP) studies. Based on the data generated, vamorolone was highly stable for up to 60 minutes in human, monkey, dog, and mouse liver microsomes in the presence or absence of nicotinamide adenine dinucleotide phosphate (NADPH) and stable for up to 60 minutes in rat liver in the absence of NADPH. Moderate metabolism was apparent in

rat liver microsomes in the presence of NADPH stimulation (35% remaining), suggesting that rat was a high metabolizer of vamorolone relative to other species (mouse, dog, human) (ReveraGen Report Nos. NIH-R2526, and ADME-VBP-LM-V003).

Vamorolone did not significantly inhibit any of the cytochrome P450 (CYP) enzyme isoforms tested (CYP 1A2, 2B6, 2C8, 2C9, 2C19, 2D6, and 3A4). Vamorolone moderately induced CYP3A4 (24% to 42%), indicating that vamorolone is a potential inducer of CYP3A4 (ReveraGen Report Nos. ADME-VBP-Inhibition-V005, ADME-VBP-Induction-V006, ADME-VBP-Induction-V009).

***Excretion***

Vamorolone showed high plasma clearance in rats but, consistent with the extensive metabolism in hepatocytes from this species, the biliary and urinary excretion of the parent compound was low with an average of <0.05% of the dose recovered in bile and approximately 0.1% in urine. Overall, vamorolone showed high plasma clearance and extremely low biliary and urinary excretion (ReveraGen Report No. NCATS 2013-44).

### *1.2.3 Toxicology*

***Single Dose***

Crl:CD1(ICR) mice were administered vamorolone once via oral gavage at 50, 125, 250, and 500 mg/kg and observed for abnormalities. All animals survived to their scheduled termination, and there were no significant abnormalities observed. However, a slight decrease in body weight related to vamorolone was observed in males and females at doses above 125 mg/kg. A dose dependent decrease in food consumption related to vamorolone was also observed in males and females. There were no other clinical observations (ReveraGen Report No. 1998-002).

Beagle dogs received single 60, 180, 360, and 750 mg/kg doses of vamorolone using an escalation study design with a 4-day washout period between doses. All animals survived dose escalation. Clinical signs attributed to vamorolone (750 mg/kg) included red discoloration of the ears and face. This effect occurred within a few hours of dosing and was transient. The highest dose also resulted in increased white blood cell count

(increased neutrophils and monocytes [female only] and decreased lymphocytes and eosinophils [male and female]). At the 360 and 750 mg/kg dose levels, slight elevations in albumin were observed. A mild elevation in cholesterol at the 750 mg/kg (and possibly 360 mg/kg) dose level was also observed (ReveraGen Report No. 13788.01.01).

In cynomolgus monkeys, single oral doses of up to 500 mg/kg were well tolerated with no significant clinical observations (ReveraGen Report No. 1998-001).

***Multiple Dose***

Acute Toxicity Studies

Vamorolone or vehicle was administered to Crl:CD1(ICR) mice QD for 28 consecutive days at doses of 10, 30 and 100 mg/kg/day. All animals survived to their scheduled necropsy with the exception of a female mouse (100 mg/kg/day dose group) that was found dead on Day 16. The cause of death was considered incidental and attributed to a dosing injury based on the amount of red fluid in the thoracic cavity.

No effects attributable to vamorolone were observed on clinical observations, food consumption, ophthalmic examination, or urinalysis during the study. A dose-dependent decrease in body weight gain was observed at all doses; however, weight was fully regained during the recovery period. Adrenal gland weights were variable between groups and generally decreased, but without a dose response relationship, and correlated microscopically with minimal to moderate vacuolar degeneration and cortical atrophy. After the 2-week recovery period there was evidence of vacuolar degeneration. Liver weights were significantly increased at the 100 mg/kg/day dose level. Hepatocellular hypertrophy, increased vacuolation, and necrosis (single cell) were seen in a few male mice at 30 mg/kg/day. There was evidence of lipid and glycogen accumulation. Serum alanine aminotransferase and aspartate aminotransferase levels were higher with associated microscopic hypertrophy/vacuolation/necrosis at 100 mg/kg/day. Spleen weights decreased in a dose-dependent manner and correlated with a decreased number of lymphocytes in spleen. Thymus weights decreased in a dose dependent manner and were associated microscopically with lymphoid atrophy. Mice had dose-dependent reductions in serum lymphocytes which were significant in the 100 mg/kg dose group.

After the recovery period, all parameters returned to normal (untreated) except for thymus weights, which were increased.

Based on the liver-related findings in this study, the no observed adverse effect level (NOAEL) for vamorolone in mice is 30 mg/kg/day (ReveraGen Report No. 1998-009).

Vamorolone or vehicle was administered to beagle dogs QD for 28 consecutive days at doses of 2, 10 and 50 mg/kg/day. All animals survived to their scheduled termination and no effect of vamorolone was noted on body weight, body temperature, food consumption, ophthalmology, electrocardiography, macroscopic, or urinalysis parameters at necropsy. A dose-dependent decrease in body weight gain was observed at all doses but weights generally returned to normal during the recovery period.

Adrenal gland weights decreased which correlated with mild or moderate diffuse bilateral atrophy of the adrenal cortex, mild multifocal bilateral vacuolation of the adrenal cortex, increased white blood cell and neutrophil counts, and decreased eosinophil counts. Liver weights increased in the 50 mg/kg/day dose group, which correlated with diffuse hypertrophy and vacuolation and increased levels of alkaline phosphatase and gamma glutamyltransferase. Spleen weights decreased, which correlated with lymphoid depletion. Thymus weights decreased, which corresponded to diffuse lymphoid depletion. With the exception of diffuse depletion of lymphocytes in thymus in the 50 mg/kg group, all abnormal parameters returned to normal during the recovery period.

The NOAEL was considered by the study director to be 10 mg/kg/day. Although reversible, the liver changes were considered adverse at 50 mg/kg/day because the severity score was moderate and the changes were diffuse in nature in all animals treated at the high dose. This is in contrast to the conclusion drawn by the study pathologist, who considered the NOAEL to be 50 mg/kg/day due to reversibility following cessation of dosing (ReveraGen Report No. 31302).

Non-naive cynomolgus monkeys were administered vamorolone or vehicle QD for 7 consecutive days at doses of 100, 300, and 600 mg/kg. All animals survived until the end of the study period. There were effects on clinical observations, food consumption, and urinalysis attributable to vamorolone.

There was a dose proportional decrease in body weight gain observed in males and females at each dose (up to 11% and 9% respectively) related to vamorolone. A cessation of the body weight loss in treatment was observed during the recovery phase but no recovery of body weight lost during the 7 days of dosing was observed.

At termination there were non-significant increases in red cell mass and decreases in lymphocytes (up to 56%) in the 600 mg/kg/day dose group. However, most individual animals, including controls, had decreases in lymphocytes (up to 81%) at termination relative to their respective pretest. They had resolved by the recovery interval in both sexes.

In both sexes receiving ≥ 300 mg/kg/day, there was increased urea nitrogen (up to 141%), creatinine (up to 58%), total protein (up to 15%), albumin (up to 11%), globulin (up to 25%), and/or potassium (up to 39%) with concurrent decreases in sodium (up to 10%) and chloride (up to 10%) relative to controls. At the recovery interval, the majority of these effects had resolved (ReveraGen Report No. 1998-001).

Chronic toxicity studies

*26-week chronic toxicity study in mice*

A 26-week chronic toxicity study was carried out in Crl:CD1®(ICR) mice to evaluate the reversibility, progression, or delayed appearance of any observed changes following a 4-week postdose observation period. Doses of vamorolone tested were 5, 15, and 45 mg/kg/day. Assessment of toxicity was based on mortality, clinical observations, body weight, and food consumption; ophthalmoscopic examinations; and clinical and anatomic pathology. Toxicokinetic assessment was conducted for the test article.

There were no vamorolone-related effects on mortality, detailed clinical observations, food consumption, ophthalmology, sperm evaluations, or bone lengths (femur or tibia).

Five test article-treated mice were unscheduled deaths (euthanized *in extremis* or found dead) during the dosing phase. Three of these were considered to be potentially due to dosing injury based on microscopic findings in mediastinum, epicardium, or lung. One of these unscheduled deaths was attributed to moderate progressive nephropathy; a

spontaneous background finding. The death of one male at 5 mg/kg/day was undetermined since there were no major pathologic findings to explain the unscheduled death of this animal; there was no target organ toxicity in the mouse. Target organ toxicity was not considered a contributor to the death of these animals and there was no dose-relationship in incidence.

A vamorolone-related increase in body weight gain was observed relative to controls in males (+14%) and females (+23%) at 45 mg/kg/day. Increases in body weights at 45 mg/kg/day were not considered to be adverse due to the general health of the animals overall. During the recovery phase, bodyweights in males returned to comparable levels with controls, however female body weights remained increased compared to female controls.

Evidence of a minimal to mild vamorolone-related hepatic effects were observed in males at ≥ 5 mg/kg/day and females at 45 mg/kg/day, indicated by mild to moderate increases in alkaline phosphatase, alanine aminotransferase, aspartate aminotransferase activities, and/or total bilirubin, as related to microscopic hepatocellular vacuolation, inflammation, and/or necrosis in males at ≥ 15 mg/kg/day and females at 45 mg/kg/day; these changes had generally resolved at recovery collections with the exception of minimal increases in alanine aminotransferase activity in females at 45 mg/kg/day, which may have correlated to microscopic liver pathology.

A mild vamorolone-related increase in neutrophil counts was observed in both sexes at 45 mg/kg/day with concurrent decreases in lymphocyte counts in females at 45 mg/kg/day consistent with a glucocorticoid-like effect, as related to microscopic lymphoid depletion, although an inflammatory stimulus may have contributed to increases in neutrophil counts, as related to microscopic liver inflammation; these changes had generally resolved at recovery collections.

A mild vamorolone-related decrease in chloride was observed in males at ≥ 5 mg/kg/day and females at ≥ 15 mg/kg/day that lacked correlative findings among other study endpoints; resolution for this endpoint could not be determined.

A mild vamorolone-related increase in albumin was observed in males at ≥ 5 mg/kg/day and females at 45 mg/kg/day with concurrent mild increases in globulin in females at 45 mg/kg/day; these changes had resolved at recovery collections.

A minor vamorolone-related alteration in lipid metabolism was observed in both sexes at 45 mg/kg/day and females at 15 mg/kg/day indicated by increases in triglyceride and/or cholesterol; these changes had resolved at recovery collections.

Vamorolone-related macroscopic findings occurred in the liver of mice at 45 mg/kg/day. Tan discoloration occurred in one female and four males at this dose in the dosing phase. This correlated with microvesicular/macrovesicular hepatocyte vacuolation. There were no test article-related macroscopic findings in recovery animals.

Test article-related microscopic findings occurred in adrenal gland (cortical atrophy- with correlating decreases in adrenal weights in females), liver (increased severity of centrilobular hypertrophy; hepatocyte vacuolation; hepatocyte vacuolation; and inflammation/necrosis), lymphoid tissues (thymus, spleen, mandibular lymph node, mesenteric lymph node, and gut-associated lymphoid tissue [GALT]) skin, and pancreatic islets (minimal to mild hypertrophy). Observed changes in these tissues are considered pharmacologically-mediated and not adverse.

An increased incidence of decreased anagen hair follicles occurred in mice at 45 mg/kg/day. Decreased anagen hair follicles was documented for individual animals when there were no anagen hair follicles in the section of skin. Incidence in controls and mice at 5 and 15 mg/kg/day were similar. A severity score was not given to the decrease as this may have been somewhat dependent on size of skin sample. This change is not considered adverse.

There was full reversibility of lymphoid changes in thymus, spleen, mesenteric lymph node, mandibular lymph node, and GALT. There were no meaningful differences between treated and controls at the end of the recovery tissues for these lymphoid tissues. There was recovery of adrenal gland findings in females and partial recovery of adrenal gland findings in males. In addition, there was partial reversibility of liver findings for males and females. Minor changes persisted in the pancreas and skin.

Systemic exposure to vamorolone appeared to be sex-dependent on Day 1 (males > females) and appeared to be independent of sex on Day 179. Following daily administration of vamorolone in females and males, systemic exposure ($AUC_{0\text{-}24hr}$) and $C_{max}$ values of vamorolone increased with increasing dose in a greater than dose proportional manner on Day 1 and in an approximately dose proportional manner on Day 179. Systemic exposure to vamorolone in females appeared to increase following repeated administration of vamorolone at 5 mg/kg, did not appear to change following repeated administration of vamorolone at 15 mg/kg, and appeared to decrease following repeated administration of vamorolone at 45 mg/kg. Systemic exposure to vamorolone in males appeared to decrease following repeated administration of vamorolone (**Table 1**).

**Table 1. $AUC_{0\text{-}24hr}$ in Mice after 179 Days Treatment with 3 Dose Levels (5, 15 and 45 mg/kg/day)**

| Average $AUC_{0\text{-}24hr}$ (hr*ng/mL) | | | | |
|---|---|---|---|---|
| Dose (mg/kg/day) | Male | | Female | |
| | Day 1 | Day 179 | Day 1 | Day 179 |
| 5 | 5700 | 1150 | 159 | 991 |
| 15 | 11600 | 3710 | 3450 | 4240 |
| 45 | 50700 | 10200 | 27000 | 12700 |

The once daily administration of vamorolone via oral gavage to mice for 26 weeks at 5, 15, and 45 mg/kg/day did not produce any adverse effects. Therefore, the No-Observable-Adverse-Effect Level (NOAEL) is considered to be 45 mg/kg/day under the conditions of this study.

As liver is considered a primary target organ, the liver enzymes (aspartate aminotransferase [AST], alanine aminotransferase [ALT]) of mice in this study were studied at study termination (**Figure 1**).

**Figure 1 Liver Function Enzymes in GLP Chronic Toxicology Study in Mice (26 weeks)**

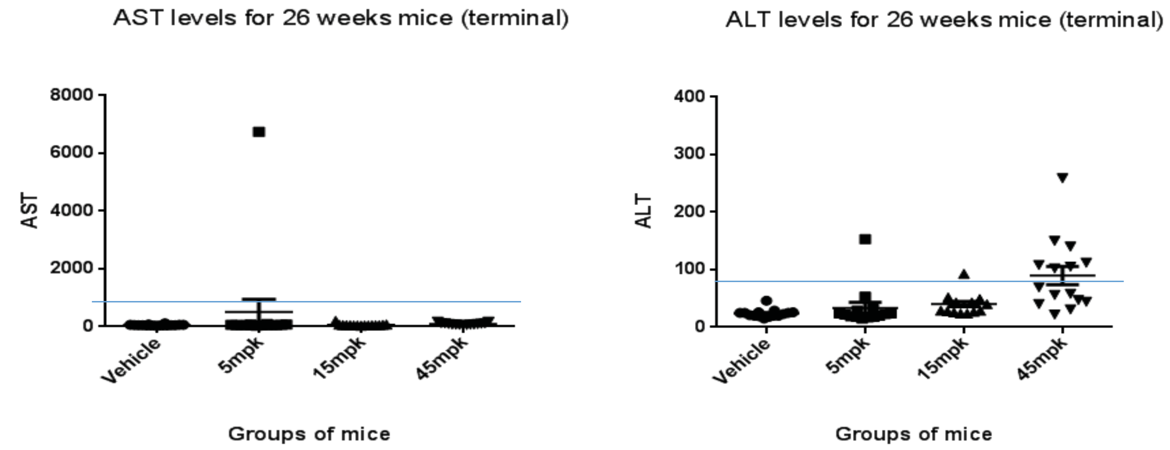


There were no differences between male and female mice regarding liver function enzymes. There were no drug-related elevations of AST at any dose. The highest dose tested (45 mg/kg/day) showed mild elevations of ALT with about half of values above the upper limit of normal range. The mild elevations of ALT were reversible, returning to within normal range after cessation of drug.

The Study Director concluded that the once daily administration of vamorolone via oral gavage to mice for 26 weeks at 5, 15, and 45 mg/kg/day did not produce any adverse effects. Therefore, the No-Observable-Adverse-Effect Level (NOAEL) is considered to be 45 mg/kg/day under the conditions of this study.

*39-week chronic toxicity study in beagle dogs*

Vamorolone or vehicle was administered to beagle dogs once daily for 39 weeks at doses of 2, 10 and 50 mg/kg/day. Six dogs of each sex received each dose or placebo, and two of the six dogs of each sex at each dose or placebo were followed for an additional 4 weeks to evaluate reversibility, progression, or delayed appearance of any observed changes. One male dog that received 50 mg/kg/day was euthanized in extremis on Day 273 due to paraphimosis (an extended penis). All other animals survived to their scheduled termination.

Detailed clinical observations considered test article-related at 50 mg/kg/day, and reversible, included decreased activity (considered adverse), struggling during dosing, feces soft, limb function impaired, interdigital cysts, and unkempt appearance (considered adverse). Test article-related, dose-dependent increases in body weight gains correlating with increases in food consumption were observed relative to controls in males at all dose levels and in females at 10 and 50 mg/kg/day. Test article-related, reversible increases in average mean food consumption were observed relative to controls over the course of the 39-week dose phase in both sexes at 10 and 50 mg/kg/day.

No test article-related ophthalmological effects were noted. No test-article-related changes were noted in respiratory rates or rectal temperatures. There may have been a mild dose-related reversible increase in the heart rate at the terminal post-dose interval that was significantly different from vehicle in both sexes following the 50 mg/kg/day dose. Semen analysis/evaluation for test article affects could not be conducted as there were not enough viable samples collected.

Test article-related effects on clinical pathology endpoints with microscopic correlates included the following:

- A hepatocellular and hepatobiliary effect in males at 10 mg/kg/day and both sexes at 50 mg/kg/day, which included increased alkaline phosphatase, gamma glutamyltransferase, alanine aminotransferase, and aspartate aminotransferase activity. These changes correlated with microscopic changes in the liver, bile duct, and gall bladder. This spectrum of changes was considered adverse in both sexes at 50 mg/kg/day.
- There was also evidence of an inflammatory response in both sexes at 50 mg/kg/day, which included increased total leukocyte, neutrophil, and monocyte counts, and increased fibrinogen and/or globulin concentrations. The inflammatory response was likely secondary to inflammation in the liver associated with hepatocellular necrosis. Platelet counts were also increased in both sexes at 50 mg/kg/day and may have been secondary to the inflammatory response.

Following a 4-week recovery period, all noted clinical pathological changes resolved, with the exceptions of increased alanine aminotransferase activity in both sexes at 50 mg/kg/day, and increased globulin in males at 50 mg/kg/day.

Reversible, test article-related macroscopic findings included mildly to moderately enlarged livers in males and females at 50 mg/kg/day, which correlated microscopically with panlobular hepatocellular hypertrophy and/or hepatocellular vacuolation; hemorrhage in the gall bladder of one 50 mg/kg/day female, that was associated with moderate acute inflammation and mild vascular necrosis, and considered to be adverse; red focus/foci within the pylorus of the stomach of one 50 mg/kg/day female and one male at 10 mg/kg/day, which correlated microscopically with mild acute inflammation in the female.

Test article-related organ weight changes at the terminal necropsy included decreases in adrenal gland weights in both sexes at ≥ 2 mg/kg/day (microscopic correlate of bilateral cortical atrophy); increases in liver weights in both sexes at ≥ 10 mg/kg/day (microscopic correlates of panlobular hepatocellular hypertrophy and/or hepatocellular vacuolation); increases in kidney weights in females at ≥ 10 mg/kg/day and males at 50 mg/kg/day (microscopic correlate of bilateral tubular vacuolation); decreases in prostate gland weights in males at 50 mg/kg/day (microscopic correlate of decreased secretory product). These organ weight changes were all reversible, except for the decreases in the prostate gland. Microscopic evaluation revealed the following test article-related changes: adrenal glands (atrophy of the zona fasciculata and zona reticularis and hypertrophy/hyperplasia of the zona glomerulosa in both sexes at ≥ 10 mg/kg/day and atrophy was considered adverse); esophagus and pylorus of the stomach (erosion/ulceration in a few animals of both sexes at 50 mg/kg/day); gallbladder (hypertrophy/hyperplasia of the mucosal epithelium in both sexes at ≥ 10 mg/kg/day and cytoplasmic vacuolation of the mucosal epithelium in males at ≥ 10 mg/kg/day and females at ≥ 2 mg/kg/day); liver (hepatocellular vacuolation in males at ≥ 10 mg/kg/day and females at ≥ 2 mg/kg/day, panlobular hypertrophy in males at 50 mg/kg/day and females at ≥ 10 mg/kg/day, and inflammation/necrosis in both sexes at 50 mg/kg/day and considered adverse, bile duct hyperplasia in both sexes at 50 mg/kg/day, bile duct

hypertrophy in males at 50 mg/kg/day and females at ≥ 10 mg/kg/day, and cytoplasmic vacuolation of the bile duct epithelium in both sexes at ≥ 10 mg/kg/day); kidneys (bilateral tubular vacuolation in males at 50 mg/kg/day and females at ≥ 10 mg/kg/day and an increased incidence of bilateral basophilic tubules in males and females at 50 mg/kg/day); lymphoid depletion in both sexes at 50 mg/kg/day in mandibular and mesenteric lymph nodes, thymus and spleen (with extramedullary hematopoiesis in 50 mg/kg/day females); bone marrow in the sternum (increased adipocytes in males at ≥ 2 mg/kg/day and females at 50 mg/kg/day); testes (spermatocyte/spermatid degeneration in males at 50 mg/kg/day); epididymides (oligospermia/germ cell debris in males at 50 mg/kg/day); ovaries (absent corpora lutea in females at ≥ 2 mg/kg/day and considered adverse); the mammary gland and other tissues in the female reproductive tract (uterus, cervix, and vagina) of these animals were consistent with animals that have not ovulated; vacuolation in the epithelium of the mammary gland duct in females at 50 mg/kg/day; parotid salivary gland (cytoplasmic alteration in both sexes at ≥ 10 mg/kg/day); biceps femoris (atrophy of the skeletal muscle in both sexes at 50 mg/kg/day); skin (atrophy and alopecia/hypotrichosis in males at 50 mg/kg/day and females at ≥ 10 mg/kg/day); prostate gland (decreased secretory product in males at 50 mg/kg/day); thyroid glands (bilateral increased colloid in males at ≥ 10 mg/kg/day).

Many of the findings were considered by the Study Director to be consistent with the pharmacology of the test article including cortical atrophy of the adrenal glands (affecting the zona fasciculata and reticularis), generalized lymphoid depletion in lymphoid tissues (thymus, spleen, and lymph nodes), increased adipocytes in the bone marrow, atrophy of the skeletal muscle, alopecia/hypotrichosis and atrophy of the skin (thinning of the dermal collagen and atrophy of hair follicles and adnexa), an absence of corpora lutea in the ovary (likely indicative of delayed puberty), decreased secretory product in the prostate gland, and multiple changes in the liver. The liver had panlobular hypertrophy and vacuolation of hepatocytes consistent with glycogen accumulation. Due to the magnitude of hypertrophy and vacuolation, there were (likely secondary) foci of hepatocellular necrosis and inflammation.

Test article-related microscopic findings at the recovery necropsy were present in the adrenal glands, liver, gallbladder, kidneys, stomach (pylorus), female reproductive tract (ovaries), male reproductive tract (testes, epididymides, prostate gland), mesenteric lymph node, skeletal muscle (biceps femoris), and parotid salivary gland.

The No Observed Adverse Effect Level was 2 mg/kg/day for males; a No Observed Adverse Effect Level was not observed for females (ReveraGen Report No. 1998-014).

*7-day study in cynomolgus monkeys*

Non-naive cynomolgus monkeys were administered vamorolone or vehicle QD for 7 consecutive days at doses of 100, 300, and 600 mg/kg. All animals survived until the end of the study period. There were effects on clinical observations, food consumption, and urinalysis attributable to vamorolone that are described below.

There was a dose proportional decrease in body weight gain observed in males and females at each dose (up to 11% and 9% respectively) related to vamorolone. A cessation of the body weight loss in treatment was observed during the recovery phase but no recovery of body weight lost during the 7 days of dosing was observed.

At termination there were nonsignificant increases in red cell mass and decreases in lymphocytes (up to 56%) in the 600 mg/kg/day dose group. However, most individual animals, including controls, had decreases in lymphocytes (up to 81%) at termination relative to their respective pretest. These decreases had resolved by the recovery interval in both sexes.

In both sexes receiving ≥ 300 mg/kg/day, there was increased urea nitrogen (up to 141%), creatinine (up to 58%), total protein (up to 15%), albumin (up to 11%), globulin (up to 25%), and/or potassium (up to 39%) with concurrent decreases in sodium (up to 10%) and chloride (up to 10%) relative to controls. At the recovery interval, the majority of these effects had resolved (ReveraGen Report No. 1998-001).

***Genotoxicity***

The mutagenic and genotoxic potential of vamorolone was assessed in several assays. A non-GLP Ames screen was negative for bacterial mutations (ReveraGen Report No.

BIO-VBP-001-AMES). In a GLP Ames test, no background lawn toxicity was observed; however, a reduction in revertant counts was observed (ReveraGen Report No. AD79DT.502ICH.BTL). Vamorolone was negative for inducing chromosomal aberrations in cultured mouse lymphocytes without and with metabolic activation (ReveraGen Report No. AD79DT.704.BTL).

Femoral bone marrow was microscopically evaluated for the presence of polychromatic erythrocytes (PCEs) containing micronuclei. No significant reductions in the PCEs/EC (total erythrocytes) ratio were observed in the vamorolone groups compared to the vehicle control group. Although statistically significant increases in the incidence of micronucleated PCEs in the vamorolone treated groups were observed, no dose response was observed with respect to other groups and the values of micronuclei for the individual animals were within the historical range. Therefore, the statistically significant increase was considered as biologically insignificant (ReveraGen Report No. AD76BK.123012ICH.BTL).

A study was performed to evaluate the potential mutagenicity of two theoretical epoxide impurities related to the drug substance vamorolone (formerly VBP15), which is a steroid-like structure containing a delta 9,11 double bond. The delta 9,11 epoxide structures evaluated were VBP15-B-3, which is structurally similar to vamorolone except for the epoxide moiety, and VBP15-B-2, which has a 21-acetate substitution (vamorolone and VBP15-B-3 contain a 21-hydroxy moiety). Two validated and complementary *in silico* prediction methodologies were used for assessing mutagenic potential. The statistics-based quantitative structure-activity relationship (QSAR) program MultiCASE CASE Ultra was used, employing four different modules (GT1_A7B, GT1_AT_ECOLI, PHARM_ECOLI, and PHARM_SAL) designed to cover a wide range of molecular substructures collected from both proprietary and public compounds. In addition, the expert rule-based SAR program Derek Nexus was used to determine if the theoretical impurities contained structural alerts associated with known genotoxicants. CASE Ultra predicted both VBP15-B-2 and VBP15-B-3 as negative for mutagenicity (ReveraGen Report “In Silico Mutagenicity Evaluation of Delta 9,11 Epoxide Structures of VBP15: VBP15-B-2 [21-Acetate] and VBP15-B-3 [21-Hydroxy]”).

Taken together, these data indicate vamorolone is negative for any mutagenic signal.

## 1.3 Clinical Experience

Clinical experience with vamorolone is comprised of a completed Phase I clinical trial of vamorolone in healthy adult volunteers (VBP15-001), completed Phase IIa (VBP15-002) and Phase II extension (VBP15-003) trials in DMD boys, and an ongoing long-term extension (VBP15-LTE) trial in DMD boys.

### *1.3.1 Phase I Study in Healthy Adult Male Volunteers (VBP15-001)*

***Study Design and Objectives***

The safety, tolerability, and PK of vamorolone were initially evaluated in a Phase I randomized, placebo-controlled, double-blind, single ascending dose (SAD) and multiple ascending dose (MAD) study. In the SAD portion of the study, Cohorts 1 through 5 and Cohort 7 were comprised of eight subjects each; six subjects in each cohort received a single oral dose of vamorolone (0.1 mg/kg, 0.3 mg/kg, 1.0 mg/kg, 3.0 mg/kg, 8.0 mg/kg, and 20 mg/kg, respectively) and two subjects in each cohort received placebo under fasted conditions. In Cohort 6, six subjects received a single oral dose of 8.0 mg/kg vamorolone within 30 minutes of beginning a high fat/high calorie meal. The MAD portion of the study had four cohorts of 8 subjects each; six subjects in each cohort received 14 daily doses of vamorolone (1.0, 3.0, 9.0 and 20.0 mg/kg/day) and two subjects in each cohort received placebo.

The primary objectives of the Phase I study were to evaluate the safety and tolerability of single and multiple oral doses of vamorolone, and to evaluate the PK of single doses and multiple doses of vamorolone. A secondary objective was to evaluate the effect of food on the absorption and PK of vamorolone. Other objectives were to obtain samples from subjects on Day 1 (pre-dose) and Day 14 of the MAD cohorts for use in Metabolites in Safety Testing (MIST) assessments, and to test back-up PK samples from a subset of MAD subjects for pharmacodynamic (PD) biomarkers.

***Pharmacokinetics***

SAD Cohorts – Pharmacokinetics (Fasted)

Vamorolone PK data shows strong adherence to dose linearity and dose proportionality, with relatively little subject-subject variation (**Figure 2**, **Table 2**, **Figure 3**). The half-life was about 2 hours for doses 0.1-1.0 mg/kg. Doses at 3.0, 8.0 and 20.0 mg/kg showed an extended tail, increasing half-life to 2.5, 3.3 and 4.3 hours, respectively (**Figure 2, Table 2**).

**Figure 2. Plasma Concentrations of Vamorolone (VBP15) after Oral Administration of Single Doses of 0.1, 0.3, 1, 3, 8, and 20 mg/kg to Healthy Subjects Under Fasted Conditions**

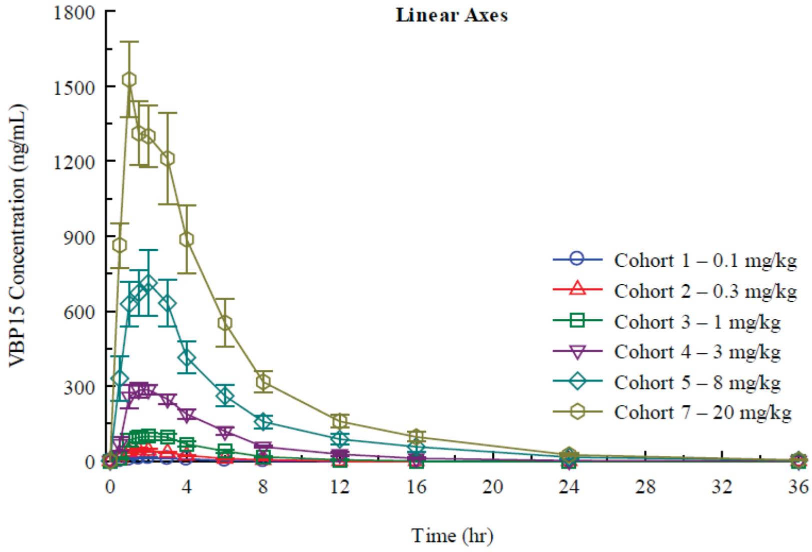


Data presented as arithmetic mean ± standard error

**Table 2. Summary of Pharmacokinetic Parameters for Vamorolone after Oral Administration of Single Doses of 0.1, 0.3, 1.0, 3.0, 8.0, and 20.0 mg/kg to Healthy Subjects Under Fasted Conditions**

| | Dose | | | | | |
|---|---|---|---|---|---|---|
| Parameter* | 0.1 mg/kg | 0.3 mg/kg | 1 mg/kg | 3 mg/kg | 8 mg/kg | 20 mg/kg |
| Cmax (ng/mL) | 13.1 (12.8) (6) | 50.8 (16.5) (6) | 122 (32.8) (6) | 305 (24.4) (6) | 718 (42.5) (6) | 1,648 (16.7) (6) |
| Tmax (hr) | 1.50 (6) | 1.50 (6) | 1.75 (6) | 1.75 (6) | 1.78 (6) | 1.50 (6) |
| | [1.50 – 2.03] | [1.00 – 3.00] | [1.00 – 3.00] | [1.00 – 2.00] | [1.00 – 2.00] | [1.00 – 3.03] |
| AUC(0-t) (hr×ng/mL) | 41.9 (16.8) (6) | 161 (15.9) (6) | 486 (19.7) (6) | 1,577 (20.7) (6) | 3,997 (55.0) (6) | 8,545 (29.5) (6) |
| AUC(inf) (hr×ng/mL) | 49.5 (12.5) (6) | 170 (16.5) (6) | 500 (19.2) (6) | 1,600 (20.3) (6) | 3,602 (60.2) (4) | 8,653 (37.0) (4) |
| λz (1/hr) | 0.4060 (12.5) (6) | 0.4325 (17.8) (6) | 0.3828 (17.9) (6) | 0.2773 (16.3) (6) | 0.2136 (40.9) (4) | 0.1629 (25.2) (4) |
| t½ (hr) | 1.71 (12.5) (6) | 1.60 (17.8) (6) | 1.81 (17.9) (6) | 2.50 (16.3) (6) | 3.25 (40.9) (4) | 4.26 (25.2) (4) |
| CL/F | | | | | | |
| (L/hr/kg) | 2.02 (12.5) (6) | 1.76 (16.5) (6) | 2.00 (19.2) (6) | 1.88 (20.3) (6) | 2.22 (60.2) (4) | 2.31 (37.0) (4) |
| (L/hr) | 168 (20.8) (6) | 142 (14.4) (6) | 165 (12.0) (6) | 152 (18.5) (6) | 196 (57.6) (4) | 180 (29.8) (4) |
| Vz/F | | | | | | |
| (L/kg) | 4.98 (6.14) (6) | 4.07 (20.5) (6) | 5.22 (18.5) (6) | 6.76 (28.7) (6) | 10.4 (61.8) (4) | 14.2 (37.2) (4) |
| (L) | 415 (17.4) (6) | 329 (19.6) (6) | 432 (22.8) (6) | 550 (28.7) (6) | 919 (63.1) (4) | 1,107 (34.6) (4) |

*Geometric mean (%CV) (N) except $T_{max}$ for which the median (N) [Range] is reported.
$C_{max}$ = maximum observed plasma concentration; $T_{max}$ = time to maximum observed plasma concentration; $AUC_{(0-t)}$ = area under concentration-time curve from time 0 to time t; $AUC_{(inf)}$ = area under concentration-time curve from time 0

to infinity; $\lambda_z$ = elimination rate constant; $t_{½}$ = terminal half-life; CL/F = apparent total clearance from plasma; $V_z/F$ = apparent volume of distribution during terminal phase.

**Figure 3. Relationship Between Individual Subject Vamorolone AUC(Inf) and Dose After Oral Administration of Single Doses of 0.1, 0.3, 1, 3, 8, and 20 mg/kg to Healthy Subjects Under Fasted Conditions**

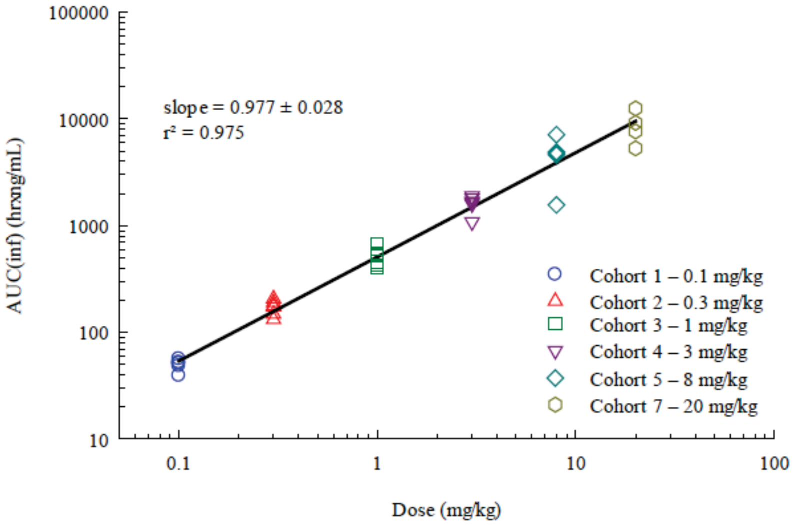


<u>SAD Cohorts – Pharmacokinetics (Fed)</u>

For the food effect group, a high fat meal (45 grams fat) was given to a cohort of Phase I SAD volunteers with the 8.0 mg/kg dose of vamorolone. These data were then compared to the fasted 8.0 mg/kg cohort data. This showed that absorption was increased by 2.5-fold by the high fat meal, consistent with the lipophilic character of vamorolone (steroidal compound) (**Figure 4**, **Table 3**).

**Figure 4. Plasma Concentrations of Vamorolone (VBP15) After Single Dose Oral Administration of 8 mg/kg to Healthy Subjects Under Fed and Fasted Conditions**

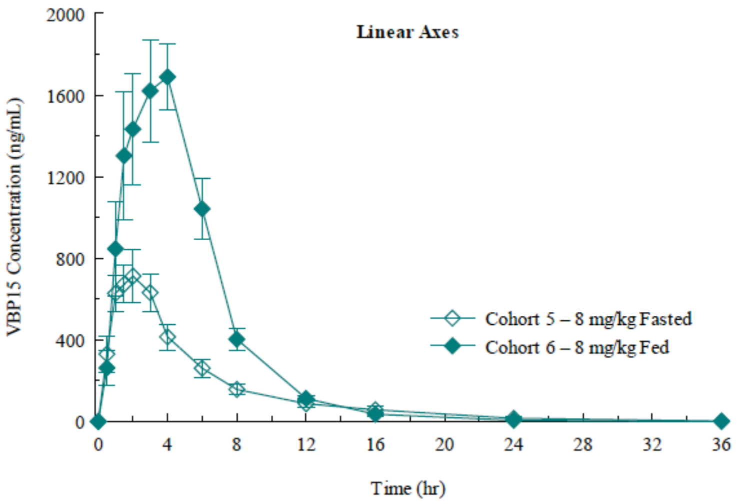


Data presented as arithmetic mean ± standard error

**Table 3. Summary of Pharmacokinetic Parameters for Vamorolone After Single Dose Oral Administration of 8 mg/kg to Healthy Subjects Under Fed and Fasted Conditions**

| Parameter* | 8 mg/kg | | Ratio† |
|---|---|---|---|
| | Fasted | Fed | |
| Cmax (ng/mL) | 718 (42.5) (6) | 1,817 (31.4) (6) | 2.53 |
| Tmax (hr) | 1.78 (6)<br>[1.00 – 2.00] | 4.00 (6)<br>[2.00 – 6.00] | |
| AUC(0-t) (hr×ng/mL) | 3,997 (55.0) (6) | 10,139 (25.1) (6) | 2.54 |
| AUC(inf) (hr×ng/mL) | 3,602 (60.2) (4) | 10,170 (24.9) (6) | 2.82 |
| λz (1/hr) | 0.2136 (40.9) (4) | 0.2950 (18.9) (6) | |
| t½ (hr) | 3.25 (40.9) (4) | 2.35 (18.9) (6) | |
| CL/F | | | |
| (L/hr/kg) | 2.22 (60.2) (4) | 0.79 (24.9) (6) | |
| (L/hr) | 196 (57.6) (4) | 66.7 (28.4) (6) | |
| Vz/F | | | |
| (L/kg) | 10.4 (61.8) (4) | 2.67 (23.4) (6) | |
| (L) | 919 (63.1) (4) | 226 (29.2) (6) | |

*Geometric mean (%CV) (N) except $T_{max}$ for which the median (N) is reported.

†Ratio of the geometric means.

$C_{max}$ = maximum observed plasma concentration; $T_{max}$ = time to maximum observed plasma concentration; $AUC_{(0-t)}$ = area under concentration-time curve from time 0 to time t; $AUC_{(inf)}$ = area under concentration-time curve from time 0 to infinity; $\lambda_z$ = elimination rate constant; $t_{½}$ = terminal half-life; CL/F = apparent total clearance from plasma; $V_z$/F = apparent volume of distribution during terminal phase: .

MAD Cohorts

The Phase I MAD treatment plan was discussed in light of the initial PK data. The relatively short half-life of vamorolone (2-4 hours), coupled with the planned daily dose schedule, would be expected to give PK data on each single dose, not cumulative dose, as the dosing interval was > 5 × $t_{½}$. Thus, the MAD component would be a study of individual daily doses, rather than dose-related accumulation and pharmacodistribution related to cumulative drug exposure. In other words, a typical goal of a MAD study is to determine steady state drug levels after multiple doses; yet with the short half-life of vamorolone, useful information would not be expected to be gained with the current daily dosing schedule. Safety and tolerability are additional goals of the MAD study, and these remain important endpoints independent of the PK studies.

MAD Cohorts – Pharmacokinetics Fasted

The original design for the Phase I MAD was modified to remove the two lowest doses (0.1, 0.3 mg/kg/day), and to begin dosing at 1.0 mg/kg/day. The clinical conduct of all four cohorts has been completed (1.0 mg/kg/day, 3.0 mg/kg/day, 9.0 mg/kg/day, 20.0 mg/kg/day) for the MAD study (**Table 4**).

**Table 4. Summary of Pharmacokinetic Parameters for Vamorolone During Oral Administration of 1, 3, 9, and 20 mg/kg Doses Once Daily for 14 Days to Healthy Subjects Under Fasted Conditions**

| | Vamorolone Dose | | | |
|---|---|---|---|---|
| | 1 mg/kg | 3 mg/kg | 9 mg/kg | 20 mg/kg |
| Day 1<br>$C_{max}$ (ng/mL) | 153 (15.9) | 281 (36.9) | 1,082 (23.3) | 2,416 (51.1) |
| $T_{max}$ (hr) | 3.04<br>[1.50 – 4.00] | 2.01<br>[1.00 – 3.00] | 1.75<br>[1.00 – 6.00] | 1.00<br>[0.50 – 3.00] |
| $AUC_{(0-t)}$ (hrxng/mL) | 686 (22.4) | 1,471 (23.6) | 5,709 (29.9) | 10,182 (28.1) |
| $AUC_{(0-24)}$ (hr×ng/mL) | 686 (22.4) | 1,471 (23.6) | 5,709 (29.9) | 10,182 (28.1) |
| $AUC_{(inf)}$ (hr×ng/mL) | 695 (22.1) | 1,487 (23.7) | 5,745 (29.5) | 10,190 (27.0) |
| $\lambda_z$ (1/hr) | 0.3848 (10.9) | 0.2918 (18.1) | 0.2317 (22.6) | 0.1747 (44.3) |
| $t_{½}$ (hr) | 1.80 (10.9) | 2.38 (18.1) | 2.99 (22.6) | 3.97 (44.3) |
| CL/F (L/hr/kg) | 1.44 (22.1) | 2.02 (23.7) | 1.57 (29.5) | 1.96 (27.0) |
| $V_z$/F (L/kg) | 3.74 (16.9) | 6.91 (34.8) | 6.76 (46.9) | 11.2 (77.6) |
| Day 14<br>$C_{max}$ (ng/mL) | 203 (30.1) | 276 (35.6) | 935 (48.3) | 2,491 (27.9) |
| $T_{max}$ (hr) | 2.96<br>[1.50 – 3.00] | 2.50<br>[1.00 – 4.00] | 1.25<br>[0.55 – 3.00] | 2.00<br>[1.00 – 2.00] |
| $AUC_{(0-24)}$ (hr×ng/mL) | 794 (22.3) | 1,494 (18.6) | 4,366 (20.2) | 9,309 (38.8) |
| $\lambda_z$ (1/hr) | 0.3993 (20.4) | 0.3273 (25.2) | 0.1629 (63.5) | 0.1879 (31.6) |
| $t_{½}$ (hr) | 1.74 (20.4) | 2.12 (25.2) | 4.25 (63.5) | 3.69 (31.6) |
| CL/F (L/hr/kg) | 1.26 (22.3) | 2.01 (18.6) | 2.06 (20.2) | 2.15 (38.8) |
| $V_z$/F (L/kg) | 3.15 (20.6) | 6.14 (39.7) | 12.7 (79.9) | 11.4 (49.1) |

$C_{max}$ = maximum observed plasma concentration; $T_{max}$ = time to maximum observed plasma concentration; $AUC_{(0-t)}$ = area under concentration-time curve from time 0 to time t; $AUC_{(0-24)}$ = area under concentration-time curve from time 0 to 24 hours; $AUC_{(inf)}$ = area under concentration-time curve from time 0 to infinity; $\lambda_z$ = elimination rate constant; $t_{½}$ = terminal half-life; CL/F = apparent total clearance from plasma; $V_z$/F = apparent volume of distribution during terminal phase.

Taking into account the small numbers and different subjects, the geometric mean values for $C_{max}$, AUC($_{0-t}$), and AUC($_{inf}$) are not different for the SAD and MAD cohorts. Within the MAD, there is good agreement between Days 1 and 14 at all dose groups. There is no accumulation — the geometric mean $C_{max}$ and $AUC_{(0-24)}$ on Days 1 and 14 are not different, consistent with the $t_{½}$ (~2-4 hours) and dosing interval (24 hours) (**Figure 5**; **Table 4**).

**Figure 5. Plasma Concentrations of Vamorolone (VBP15) on Days 1 and 14 During Oral Administration of 1, 3, 9, and 20 mg/kg Doses Once Daily for 14 Days to Healthy Subjects Under Fasted Conditions**

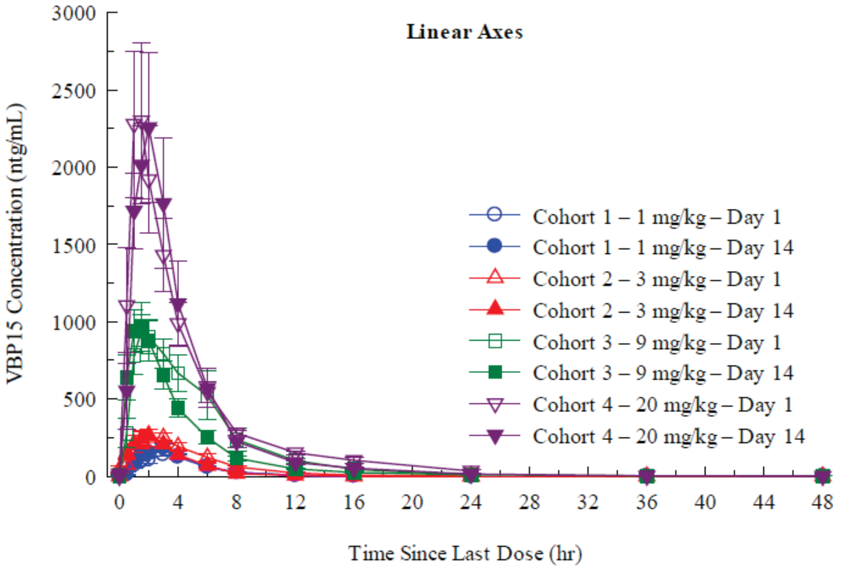


Data presented as arithmetic mean ± standard error

## *Safety*

### SAD Cohorts

In the SAD part, overall, 6 subjects (11%) administered vamorolone experienced a total of 10 treatment-emergent adverse events (TEAEs); no subject in the placebo group experienced any TEAEs. There was no dose-related trend in the incidence or severity of TEAEs; the dose group with the highest number of subjects reporting TEAEs was the 0.1 mg/kg dose group (2 subjects, 33%), and the highest number of TEAEs (3 events) was reported by 1 subject in the 1.0 mg/kg vamorolone dose group. In the 0.3 and 3.0 mg/kg vamorolone fasted and the 8.0 mg/kg fed dose groups, 1 subject per group (17%) experienced TEAEs, and no subjects in the 8.0 mg/kg and 20 mg/kg, fasted, dose groups experienced any TEAEs. The most common TEAEs were dizziness and headache, each reported by 2 subjects overall (4%); all other TEAEs were reported by only 1 subject (2%) and included ear pain, nausea, non-cardiac chest pain, and blood bilirubin increased. Three subjects (6%) had TEAEs that were considered possibly related to treatment. Possibly related TEAEs included nausea (1 subject, 2%), dizziness (2 subjects, 4%), and headache (2 subjects, 4%). One subject (2%) had a moderate

TEAE of blood bilirubin increased, which was considered unrelated to study drug. All other TEAEs were mild in severity.

MAD Cohorts

In the MAD part, overall, a total of 6 subjects (19%) administered vamorolone or placebo experienced a total of 10 TEAEs: 2 subjects each in the 1.0 mg/kg vamorolone, 20 mg/kg vamorolone, and placebo groups, and none in the 3.0 mg/kg and 9.0 mg/kg dose groups. There was no dose-related trend in the incidence or severity of TEAEs. The most common TEAE was headache (2 subjects, 6%); all other TEAEs occurred in only 1 subject (3%) per group, and included nausea, toothache, vomiting, ALT increased, hepatic enzyme increased, arthralgia, dizziness, and syncope. TEAEs were considered possibly related in 2 subjects (6%) and remotely related in 1 subject (3%). Possibly related AEs were ALT increased and hepatic enzyme increased, occurring in 1 subject in the 20 mg/kg vamorolone and placebo groups, respectively. The remotely related TEAEs were dizziness and syncope, both occurring in the same subject (1.0 mg/kg vamorolone). All TEAEs were mild in severity.

With the exception of the AEs related to hepatic enzymes, there were no other meaningful changes in clinical laboratory parameters. Of note, glucose levels remained stable at all doses of vamorolone in both the SAD and MAD parts, suggesting that vamorolone does not induce insulin resistance at the doses and dosing duration studied; in addition, no changes in the white blood cell count differential were observed, suggesting that vamorolone did not induce immunosuppressive effects in this study population.

***Pharmacodynamic Safety Biomarkers***

Vamorolone has shown improved safety profiles relative to prednisone in nonclinical testing, both *in vitro* and *in vivo*.[15,17] Safety concerns with glucocorticoids include suppression of the adrenal axis and insulin resistance. Pharmacodynamic biomarker assays of suppression of the adrenal axis (serum cortisol) and insulin resistance (serum glucose) were measured in the Phase I MAD studies of vamorolone.

Suppression of the adrenal axis. Prednisone directly impinges on cortisol regulatory pathways (adrenal axis) both acutely and chronically. Acute suppression of adrenal function is seen within hours of doses of a single 0.1 mg/kg/day (approximate) dose of prednisone, as evidenced by reductions in adrenocorticotropic hormone (ACTH) levels in normal volunteers.[25] More chronic suppression of the adrenal axis, characterized as severe, is typically diagnosed when morning cortisol is < 100 nmol/L (< 3.6 µg/dL) when drawn > 24 hrs after the last dose of pharmacological steroids.

Morning serum cortisol levels were measured in the vamorolone Phase I MAD cohorts, at baseline (prior to drug administration), 24 hours after the first dose (Day 1), and 24 hours after the 14-day dose (Day 15) (**Figure 6**). Active substance volunteers at four MAD dose levels are shown (1.0 mg/kg/day; 3.0 mg/kg/day; 9.0 mg/kg/day; 20.0 mg/kg/day); all subjects were treated for 14 days with daily dosing. The red hatched line on each graph shows a typical threshold for adrenal axis suppression (< 100 nmol/L, or < 3.6 µg/dL). P values shown are for paired T test, indicating significance of the consistency of longitudinal changes of subjects relative to their own individual baseline values. Acute adrenal axis suppression is measured at 24 hours (after first dose), whereas chronic adrenal axis suppression is measured after 14 days of daily dosing (24 hours after last dose).

**Figure 6. Morning Cortisol Measurements in the Vamorolone Phase I Healthy Subjects**

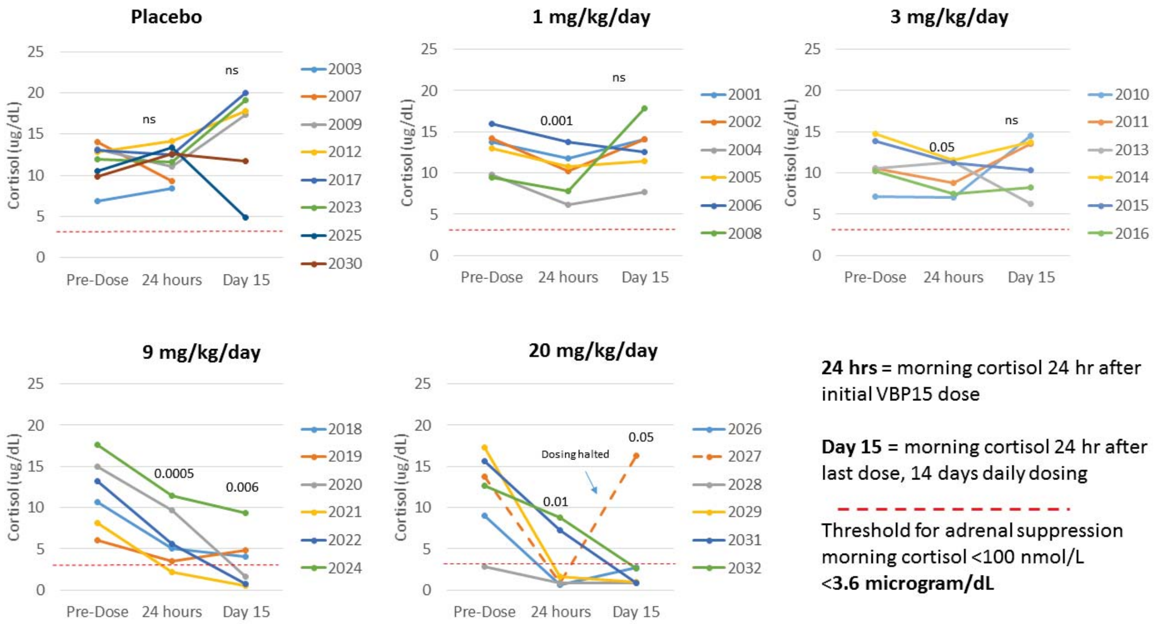


Note: Placebo subjects from each of the four MAD cohorts are graphed together.

Vamorolone showed little evidence of either acute (24 hour data), or chronic (Day 15 data) suppression of the adrenal axis at doses of either 1.0 mg/kg/day or 3.0 mg/kg/day. The data suggest that vamorolone induces variable, mild, acute and chronic suppression of the adrenal axis at 9.0 mg/kg/day, and stronger evidence of both acute and chronic adrenal axis suppression at 20.0 mg/kg/day. Prednisone typically shows both acute and chronic adrenal axis suppression approximately at 0.1 mg/kg/day,[25] suggesting that vamorolone has an improved safety window regarding adrenal axis suppression.

Vamorolone thus shows approximately a 10-fold improvement in safety window compared to prednisone on a mg/kg comparative basis. These data are consistent with *in vitro* and *ex vivo* nonclinical mouse data comparing vamorolone to prednisone for adrenal suppression.[15]

Insulin resistance. Prednisone induces the safety signal of insulin resistance, where glucose is not efficiently taken up from the blood by target tissues, such as muscle and liver, leading to hyperglycemia.[25] Insulin resistance may be an important safety signal for dystrophic muscle, where the dysfunctional myofibers have been shown to have

inadequate energy stores,[18,26] and insulin resistance likely limits availability of glycogen substrates for glycolysis. The hyperglycemia, in turn, leads to chronic increases in insulin levels (hyperinsulinemia).

Levels of fasting glucose and insulin are reasonably sensitive and reliable measures of insulin resistance in non-diabetic individuals. Glucose is acutely (single dose) and chronically (multiple doses) elevated after treatment with pharmacological glucocorticoids. Glucose is elevated 24 hours after a single administration of glucocorticoids (2.0 mg/kg).[27,28]

In the Phase I MAD of vamorolone, fasting serum glucose was measured at 10 time points during the 2-week study; each sample was taken 24 hours after the previous dose of vamorolone (**Figure 7**).

**Figure 7. Fasting Serum Glucose During the Phase I MAD Period (Two Weeks Daily Treatment)**

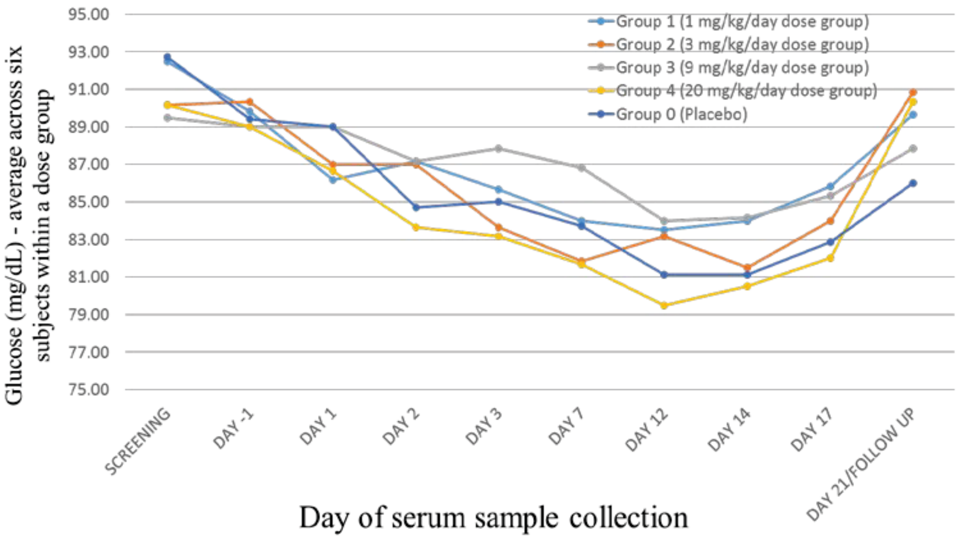


Glucose levels for all vamorolone dose groups were similar to those of the placebo group. There was no evidence of elevations of glucose levels at any time point or any dose of vamorolone, suggesting that the side effect of insulin resistance was not seen with vamorolone. These data are consistent with a nonclinical study in a dystrophin-deficient mouse model, where chronic treatment of prednisolone (5 mg/kg/day) versus vamorolone

(15 mg/kg/day; 30 mg/kg/day) showed development of insulin resistance with prednisolone, but not vamorolone.[29]

***Summary of Phase I Data***

Pharmacokinetics

- Vamorolone PK data show strong adherence to dose linearity and dose proportionality, with relatively little subject-subject variation (both SAD and MAD).
- The half-life was about 2 hours for doses 0.1-1.0 mg/kg. Doses at 3.0, 8.0, and 20.0 mg/kg showed an extended tail, increasing half-life to 2.5, 3.8, and 3.8 hours, respectively. The PK for the MAD cohorts was very similar to the SAD cohorts, showing little if any drug accumulation, consistent with the short half-life and daily dosing schedule.
- There were no apparent relationships between CL/F and body size, either in terms of body weight or BMI.
- For the food effect group, a high fat meal was given to a cohort of Phase I SAD volunteers with the 8.0 mg/kg dose of vamorolone. Comparison of the data from the high fat meal with the fasted 8.0 mg/kg cohort data showed that absorption was increased by 2.5-fold by the high fat meal, consistent with the lipophilic character of vamorolone (steroidal compound).

Safety

- Single and multiple daily doses of vamorolone up to 20 mg/kg were well tolerated by healthy subjects, and a maximum tolerated dose (MTD) was not identified.
- Regarding the primary target organ, liver, one subject in the 20 mg/kg vamorolone MAD dose group who had an elevation of serum bilirubin at baseline (pre-dose) experienced an AE of ALT increased after 9 days of dosing; this AE was judged to be possibly related to vamorolone and drug dosing was halted. No other subjects in the vamorolone dose groups experienced AEs related to liver function.

- Results of post-dose morning cortisol levels suggest that vamorolone causes acute adrenal suppression after single dosing and chronic adrenal suppression after multiple dosing, but only at the higher (≥ 8.0 mg/kg) single and multiple doses studied.
- Safety PD biomarker studies showed that vamorolone had an improved safety window for adrenal axis suppression (100-fold increase in therapeutic window), no evidence of insulin resistance, no changes in bone turnover markers (osteocalcin, C-terminal peptide fragment of collagen 1[CTX1]), compared to prednisone studies reported in the literature[25,30]
- There were no clinically significant changes in vital signs, ECGs, or physical examinations.

### *1.3.2 Pharmacokinetics in Phase IIa Study in 4 to 7 years Duchenne Muscular Dystrophy Boys (VBP15-002)*

Forty-eight boys with DMD received oral doses of vamorolone once daily for 14 days at doses of 0.25 mg/kg/day, 0.75 mg/kg/day, 2.0 mg/kg/day, and 6.0 mg/kg/day, 12 subjects receiving vamorolone at each dose level. The drug was administered with a glass of whole milk or other high-fat food. Six blood samples were collected on Day 1 and Day 14 at 0, 1, 2, 4, 6, and 8 hours post-dose and plasma was analyzed for vamorolone. The time-course of plasma drug concentrations was assessed using noncompartmental analytical (NCA) methods using the WinNonlin software (Certara).

Of the 48 DMD subjects enrolled in the study (12 at each dosing level), 47 had evaluable PK data. The average Day 1 and Day 14 vamorolone concentration versus time profiles are shown in **Figure 8** for the four dose levels. Overall, as expected, higher doses resulted in higher plasma concentrations of vamorolone. In particular, plasma vamorolone concentrations versus time showed a rapid to moderate rate of absorption, a maximum concentration ($C_{max}$) at a $T_{max}$ of 2 to 4 hours, and a decline phase with a typical half-life ($t_{1/2}$) of 2 hours. For all 4 doses, the mean PK profiles on Day 1 and Day14 appear similar. Many individual subjects had closely matching Day 1 and Day 14

profiles, but several did not. There was no accumulation of drug owing to the relatively short half-life and once-daily (morning) dosing.

**Figure 8 Mean Plasma Concentrations of Vamorolone after Once Daily Oral Administration of 0.25, 0.75, 2.0, and 6.0 mg/kg Doses**

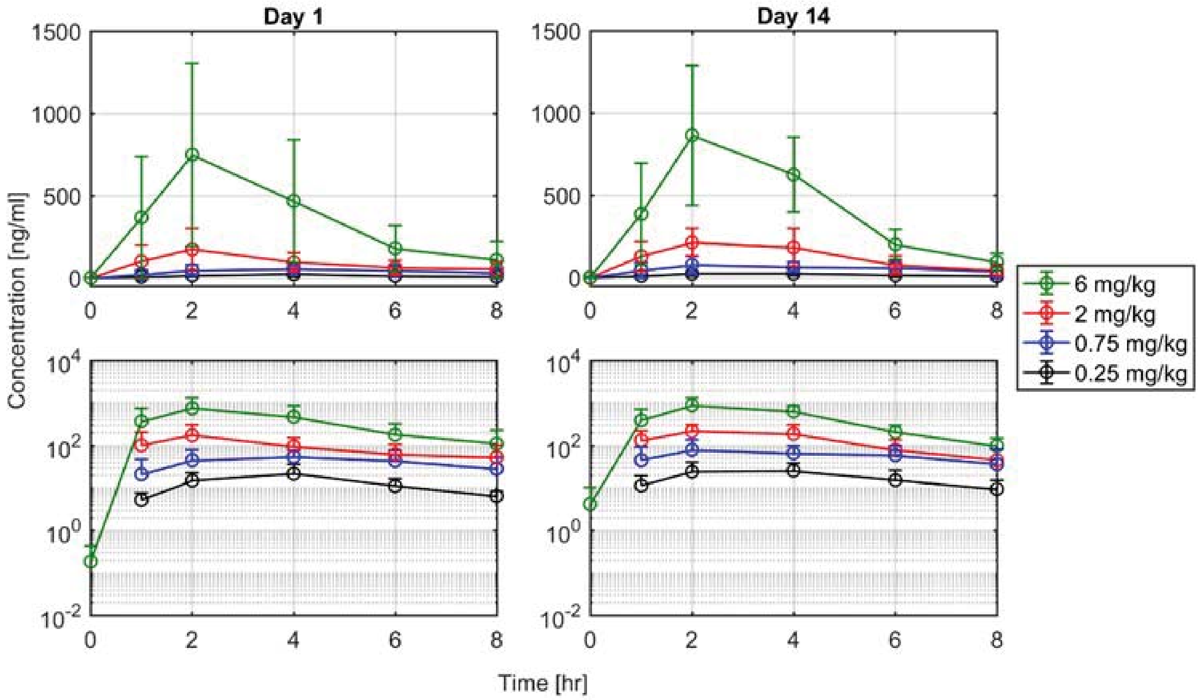


Vamorolone concentrations shown as mean ± standard deviation on linear (top panel) and semi-logarithmic (bottom panel) graphs.

A summary of the observed ($C_{max}$ and $T_{max}$) and calculated (AUC, $t_{1/2}$, and CL) PK parameters for all doses on Day 1 and Day 14 is provided in **Table 5**. Even with only 6 blood samples collected from each subject, the PK profiles adequately capture the overall exposures, but must be considered approximate. This particularly applies to the peak values and the half-lives. Nevertheless, there is good consistency in the data with moderate variability as SD values are reasonable and the coefficient of variation values are typically less than 50%.

The drug is absorbed at a moderate rate with peak concentrations typically occurring at 2 to 4 hours on both Days 1 and 14. The median $T_{max}$ across all doses is typically 2 or 4 hours. The half-lives were similar on both days, averaging about 2 hours, although some subjects had irregular profiles without a clear decline phase. The apparent clearance (CL

and CL/F) values were consistent across the dose levels for Day 1 as well as for Day 14, but the latter tended to average slightly lower (about 2400 mL/hr/kg on Day 1 and 1900 mL/hr/kg on Day 14).

**Table 5. Summary of Pharmacokinetic Parameters for Vamorolone after Once Daily Oral Administration of 0.25, 0.75, 2.0 and 6.0 mg/kg Doses to DMD Boys**

| | Day 1 | | | | Day 14 | | | |
|---|---|---|---|---|---|---|---|---|
| | Dose, mg/kg/day | | | | Dose, mg/kg/day | | | |
| Parameters | **0.25** | **0.75** | **2.0** | **6.0** | **0.25** | **0.75** | **2.0** | **6.0** |
| Cmax [ng/ml] | 22.9 (13.4) | 75.9 (25.9) | 199 (111) | 855.6 (471) | 32.2 (15.2) | 124.7 (42.5) | 252.5 (96) | 970 (270) |
| $T_{max}$ [h] | 3.6 [4] (1.2) | 4.6 [4] (2.1) | 2.5 [2] (1.3) | 2.7 [2] (1.3) | 3.8 [4] (1.8) | 3.8 [4] (2.2) | 2.8 [2] (1) | 2.3 [2] (0.86) |
| $AUC_{inf}$ [hr·ng/ml] | 118 (48) | 379 (117) | 761 (352) | 3279 (1693) | 164 (61) | 544 (155) | 1138 (467) | 3606 (897) |
| $t_{1/2}$ [h] | 2.1 (0.85) | 1.8 (0.43) | 1.9 (0.79) | 1.9 (0.95) | 1.9 (0.96) | 2.1 (0.8) | 1.9 (1.02) | 1.4 (0.35) |
| CL [ml/hr/kg] | 2459 (897) | 2285 (1103) | 2697 (1285) | 2320 (1375) | 1828 (919) | 1509 (482) | 2047 (771) | 1777 (476) |

Values are mean (standard deviation) and for $T_{max}$, which are shown as mean [median] (standard deviation).

An assessment of the linearity of the PKwith dose using $C_{max}$ and AUC is shown in **Figure 9**. Data for all individual subjects were used which also depict the variability for each dose level. While larger doses appear to present greater variability, this is a visual size distortion as the standard deviation is similar at each dose level (**Table 5**). Fitting the power equation of the form ($C_{max}$ , $AUC_{inf}$ = a x·Dose$^{b}$) resulted in 95% confidence intervals for *b* values that include 1.0 for $C_{max}$ and $AUC_{inf}$ for both Days 1 and 14 indicative of linear relationships across this range of dose levels.

**Figure 9 Regression Analysis of $C_{max}$ and $AUC_{inf}$ versus Dose**

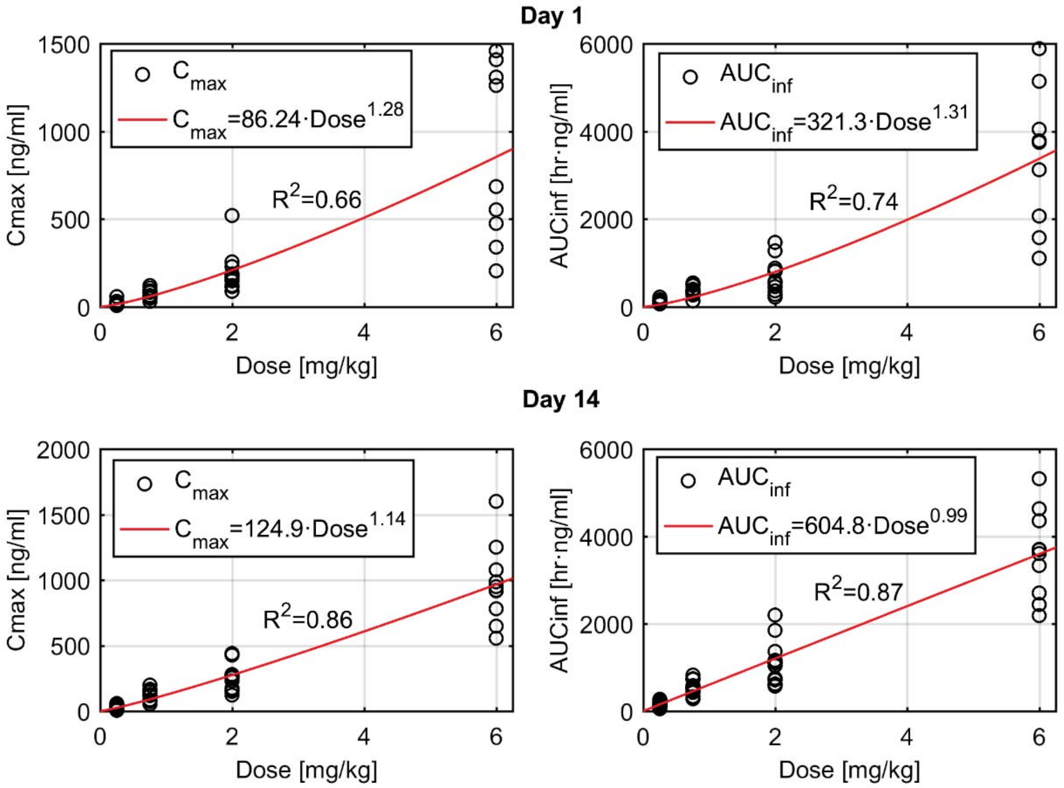


Regression was performed by fitting the indicated power equations to the data for Day 1 and Day 14. The fitted parameters are shown in the figure boxes.

Compared with fasting healthy adult volunteers (**Section 1.3.1**), the boys with DMD showed lower $C_{max}$ values and more variation in the time to reach the maximal concentration of vamorolone for any given dose. This observation might be due to the fact that the boys with DMD received vamorolone after 8 ounces of whole milk or other high fat food and a so-called food effect might have played a role. The apparent clearance of vamorolone seems higher in the boys with DMD but this finding is not significant.

### *1.3.3 Safety in Phase II Studies in 4 to7 years Duchenne Muscular Dystrophy Boys (VBP15-002 and VBP15-003)*

*Adverse events:* There were no serious adverse events (SAEs) reported over the 14-day treatment in the Phase I clinical trial in healthy adult volunteers, nor in the four cohorts (0.25 mg/kg, 0.75 mg/kg, 2.0 mg/kg, and 6.0 mg/kg) of the Phase IIa study (VBP15-002; 14-day treatment) in boys ages 4 to <7 years with DMD. There were a total of 4 SAEs in

the Phase II VBP15-003 study and three SAEs to date in the VBP15-LTE extension study: two SAEs of pneumonia in two different subjects (both subjects receiving vamorolone 0.75 mg/kg/day), one SAE of bilateral testicular torsion and one SAE of hypoxia in the same subject receiving 6.0 mg/kg/day, one SAE of influenza associated dehydration in a subject receiving 6.0 mg/kg/day, and two SAEs of acute myoglobinuria in the same subject receiving 6.0 mg/kg/day. Each of these SAEs was considered unrelated to study drug, and none of them resulted in discontinuation from the study. In the VBP15-003 study, there were a total of 218 TEAEs among 42 of the 48 subjects (87.5%). In VBP15-003 study, the TEAEs with the highest incidence were viral upper respiratory tract infection (41.7%); pyrexia (35.4%); cough (18.8%); vomiting (14.6%); and diarrhea (10.4%).

*Body Mass Index (BMI):* Body Mass Index was measured throughout the VBP15-003 study. The mean change from baseline to Week 24 for BMI was 0.03, 0.20, 0.23, and 1.15 for the 0.25, 0.75, 2.0, and 6.0 mg/kg/day dose level groups, respectively. Body Mass Index increases generally reflect an increase in weight. Body Mass Index z-score was monitored in the VBP15-003 study. The mean change from baseline to Week 24 for BMI z-score for the 6.0 mg/kg/day group showed a statistically significant increase compared to the mean change from baseline to Week 24 for the 0.25 mg/kg/day and 0.75 mg/kg/day dose level groups. In contrast, the mean change from baseline to Week 24 for the 2.0 mg/kg/day dose level group in BMI z-score was minimal and comparisons with the other vamorolone dose level groups lacked statistical significance. The mean increase from baseline to Week 24 for BMI z-score was similar for the 6.0 mg/kg/day group and a daily prednisone-treated historical control group.

*Potential liver toxicity:* In the Phase I clinical trial in adult volunteers, vamorolone showed mild elevations of liver enzymes in one subject receiving 20.0 mg/kg in the fasted state, and dosing was halted. All DMD subjects have elevated serum ALT and AST enzymes because of the muscle condition. For that reason, two enzymes, glutamate dehydrogenase (GLDH) and gamma glutamyl transferase (GTT), that are preferentially expressed in liver were evaluated in the VBP15-003 study. None of the mean changes in GLDH from baseline to any of the VBP15-003 on-treatment assessment time points were

statistically significant for any dose level group. Although the mean changes from baseline to Week 8, Week 16, and Week 24 in GLDH levels did not show a dose response, shift analysis of GLDH levels did suggest a possible dose-related shift to higher GLDH levels at 2.0 mg/kg/day and 6.0 mg/kg/day at Weeks 16 and 24. Mean GGT levels and individual subject values at each VBP15-003 assessment time point across the four dose level groups remained at or below the normal range. On the basis of these mean GLDH and GGT data, vamorolone at dose levels up to 6.0 mg/kg/day does not appear to induce liver toxicity over a 24-week treatment period.

*Adrenal suppression:* In the VBP15-003 study, after 24 weeks of treatment, 0 of 8 tested participants (0.25 mg/kg/day), 1 of 12 (8.3%) tested participants (0.75 mg/kg/day), 5 of 12 (41.7%) tested participants (2.0 mg/kg/day), and 8 of 9 (88.9%) tested participants (6.0 mg/kg/day) had a depressed morning cortisol (<3.6 µg/dL [100 nM]) consistent with chronic adrenal suppression.[29]

*Insulin resistance:* In the VBP15-003 study, mean changes from baseline for fasting insulin showed dose-and time-related changes for all dose level groups at Week 12 and Week 24. Statistical significance was observed for mean increase from baseline for the 6.0 mg/kg/day dose level group at Week 12 and Week 24.

*Bone Turnover:* In the VBP15-003 study, pharmacodynamic biomarker testing for bone turnover markers suggested that vamorolone does not have the detrimental bone effects observed with prednisone and deflazacort.

## 1.4 Rationale for Study Design

The proposed Phase IIb clinical trial (VBP15-004) is designed as a pivotal study to show that vamorolone treatment over a Treatment Period of 24 weeks leads to superior improvements in strength and mobility versus placebo (efficacy), with a reduced adverse effect profile versus prednisone treatment (safety), and to demonstrate persistence of effect over a Treatment Period of 48 weeks. To determine efficacy, functional outcomes in DMD patient groups receiving one of two doses of vamorolone over 24 weeks will be compared to functional outcomes of DMD patients receiving placebo. To determine safety, body mass index (BMI) and PD safety biomarker findings in DMD patients

receiving one of two dose levels of vamorolone over 24 weeks will be compared to DMD patients receiving prednisone. The two dose levels of vamorolone to be studied have been chosen based upon data obtained in the Phase IIa and Phase II extension studies (VBP15-002; VBP15-003).

This Phase IIb study is a double-blind study of two dose levels of vamorolone, with placebo and prednisone-treated control arms.

Subjects who meet all eligibility criteria in this study (VBP15-004) will be randomized to one of six treatment groups as shown in **Table 6.**

**Table 6. Study Randomization Schedule**

| Group | Planned Number of Subjects | Treatment Period #1 (24 Weeks) | Treatment Period #2 (20 Weeks) |
|---|---|---|---|
| 1 | 30 | Vamorolone, 2.0 mg/kg/day → | Vamorolone, 2.0 mg/kg/day |
| 2 | 30 | Vamorolone, 6.0 mg/kg/day → | Vamorolone, 6.0 mg/kg/day |
| 3 | 15 | Prednisone, 0.75 mg/kg/day → | Vamorolone, 2.0 mg/kg/day |
| 4 | 15 | Prednisone, 0.75 mg/kg/day → | Vamorolone, 6.0 mg/kg/day |
| 5 | 15 | Placebo → | Vamorolone, 2.0 mg/kg/day |
| 6 | 15 | Placebo → | Vamorolone, 6.0 mg/kg/day |

Evaluation of the two dose levels of vamorolone, prednisone, and placebo during the 24-week Treatment Period #1 in this study will allow comparison of change from baseline in safety parameters, muscle strength and functional efficacy parameters, and PD biomarker levels over 24 weeks of treatment as compared to no treatment (placebo) or standard of care treatment (prednisone). In particular, evaluation of change of the PD biomarkers from baseline over a longer (24-week) period may aid in the clinical validation of biomarkers which exhibit small changes over time. In addition, evaluation of the two dose levels of vamorolone over the total 48-week period of the study will allow assessment of the persistence of effect.

The primary efficacy outcome is the Time to Stand (TTSTAND) from the floor (velocity), and comparisons will be made between each dose level of vamorolone and the placebo group at Week 24. Multiple secondary efficacy outcomes will be measured, including Time to Run/Walk 10 meters (TTRW), Time to Climb four stairs (TTCLIMB), North Star Ambulatory Assessment (NSAA), 6-Minute Walk Test (6MWT), Range of

Motion test (ROM), and hand-held myometry (elbow flexors and knee extensors). Exploratory measures of efficacy include PD biomarkers that have previously been shown to be glucocorticoid-responsive in DMD boys and inflammatory bowel disease in children.[31] Moreover, physical functioning, behavior, neuropsychology, and satisfaction with treatment will be measured as exploratory efficacy outcomes using the parent proxy-report of Pediatric Outcomes Data Collection Instrument (PODCI), PARS III, Treatment Satisfaction Questionnaire (TSQM), and Ease of Study Medication Administration Assessment for the study medication suspension, respectively.

For the safety measure of body mass index (BMI) z-score, comparisons will be made between each dose level of vamorolone and the prednisone-treated group. Additional secondary safety measures are serum biomarkers bridged to later clinical safety concerns. These include:

1. **Adrenal suppression.** Pharmacological doses of glucocorticoids cause suppression of the hypothalamo-pituitary-adrenal axis, leading to low concentration of endogenous cortisol and other steroidal hormones in serum. Adrenal suppression is directly associated with risk of adrenal crisis, delay of puberty and stunting of growth. Measurement of morning cortisol concentrations will reflect the degree of adrenal suppression. Plasma cortisol secretion typically follows a circadian pattern with the highest concentrations early in the morning; a morning serum cortisol concentration less than 3.6 µg/dL (or 100 nM) is highly suggestive of adrenal suppression. A single cortisol measurement at other times of the day is of limited value and dynamic testing with Cosyntropin (a synthetic peptide of ACTH, also known as tetracosactide) is a standard approach to the assessment of endogenous cortisol production.[32] Serum cortisol levels less than 18 µg/dL (500 nM) 30 or 60 minutes after stimulation with Cosyntropin (250 µg) are considered diagnostic of adrenal suppression.
2. **Bone turnover.** Pharmacological doses of glucocorticoids cause an imbalance of bone formation and bone resorption, leading to later osteopenia and bone fragility.[33] Bone fragility is a significant adverse effect of chronic pharmacologic

glucocorticoids in DMD as this can lead to fracture, which increases the likelihood of premature loss of ambulation. Serum biomarkers that have been bridged to later clinical outcomes of osteopenia are osteocalcin (bone formation; glucocorticoids decrease serum levels), and CTX1 (bone resorption; glucocorticoids increase serum levels). Decreases of osteocalcin and increases of CTX1 are reflective of abnormal bone turnover, a risk factor bridged to later bone fragility.

3. **Insulin resistance.** Insulin resistance is the term where increased blood glucose triggers increased insulin secretion from the pancreatic islet cells, but the elevated serum insulin fails to sufficiently trigger glucose uptake by muscle and/or liver. Thus, peripheral tissues are resistant to insulin signaling (insulin resistance). Insulin resistance has been bridged to later clinical outcomes, including heart disease, type 2 diabetes, and vascular disease. Serum biomarkers that are accepted as measures of insulin resistance are increased serum glucose and insulin. This can be measured after acute (hours after first dose) or chronic (after weeks or months of dosing) glucocorticoid treatment.
4. **Immune suppression.** Glucocorticoids can cause immunosuppression. The measure of differential lymphocyte percentage can be a biomarker for immune suppression.

Additional exploratory safety outcomes are measures of additional serum safety biomarkers that have been defined in glucocorticoid-treated DMD and inflammatory bowel disease patients.

This trial will be conducted in compliance with this protocol, and in accordance with the ethical principles that have their origin in the Declaration of Helsinki, and that are consistent with Good Clinical Practice (GCP) and the applicable regulatory requirements, and the recently issued FDA guidance on developing drugs for treatment for DMD and related dystrophinopathies.[34]

It is obligatory that the Investigator become familiar with all sections of the vamorolone Investigator's Brochure.[29]

## 1.5 Overall Benefit/Risk

It is anticipated that the adverse effect profile of the investigational product will be more favorable than standard of care glucocorticoids in the long term. There were no serious adverse events (SAEs) reported over the 14-day treatment in the Phase I clinical trial in healthy adult volunteers, nor in the four cohorts (0.25 mg/kg, 0.75 mg/kg, 2.0 mg/kg, and 6.0 mg/kg) of the Phase IIa study (VBP15-002; 14-day treatment) in boys ages 4 to <7 years with DMD. There have been a total of 7 SAEs in the Phase II VBP15-003 and VBP15-LTE extension studies in DMD boys: two SAEs of pneumonia in two different subjects (both subjects receiving vamorolone 0.75 mg/kg/day), one SAE of bilateral testicular torsion and one SAE of hypoxia in the same subject receiving 6.0 mg/kg/day, one SAE of influenza associated dehydration in a subject receiving 6.0 mg/kg/day, and two SAEs of acute myoglobinuria in the same subject receiving 6.0 mg/kg/day. Each of these SAEs was considered unrelated to study drug, and none of them resulted in discontinuation from the study. In the Phase I clinical trial in adult volunteers, vamorolone showed mild elevations of liver enzymes in one subject receiving 20.0 mg/kg in the fasted state. On the basis of mean GLDH and GGT data, vamorolone at dose levels up to 6.0 mg/kg/day did not appear to induce liver toxicity over a 24-week treatment period in the VBP15-003 study. In the VBP15-002 study, after 2 weeks of treatment, 0 of 11 tested participants who received vamorolone 0.25 mg/kg/day, 0 of 11 tested participants who received vamorolone 0.75 mg/kg/day, 2 of 11 (18.2%) tested participants who received vamorolone 2 mg/kg/day, and 6 of 10 (60.0%) tested participants who received vamorolone 6 mg/kg/day had a depressed morning cortisol (<3.6 μg/dL [100 nmol/L]) consistent with chronic adrenal suppression. In the VBP15-003 study, after 24 weeks of treatment, 0 of 8 tested participants (0.25 mg/kg/day), 1 of 12 (8.3%) tested participants (0.75 mg/kg/day), 5 of 12 (41.7%) tested participants (2.0 mg/kg/day), and 8 of 9 (88.9%) tested participants (6.0 mg/kg/day) had a depressed morning cortisol (<3.6 μg/dL [100 nM]) consistent with chronic adrenal suppression. Instructions for detecting adrenal crisis and the circumstances in which stress dose steroids should be provided will be included in the Informed Consent Form (ICF) and Manual of Operations, and Investigators should

monitor clinical study participants closely to identify elevations in liver-specific enzymes.

*Potential Health Benefits:* Subjects may or may not receive direct health benefit from participating in the study. Some subjects will be randomly assigned to vamorolone at one of two planned dose levels (2.0 mg/kg/day and 6.0 mg/kg/day) over the course of the 48-week trial. In the VBP15-003 study, clinical efficacy was assessed by Timed Function Tests. Improvement in Time to Stand, Time to Climb, Time to Run/Walk 10 Meters, and 6-Minute Walk Test were seen predominantly for the 2.0 and 6.0 mg/kg/day dose level groups, with many of the improvements showing statistical significance compared to an untreated Duchenne Natural History Group. In view of the initial clinical evidence of safety, the improvements observed in the assessments of efficacy, and the nature of potential adverse effects that can be monitored, the data support an acceptable benefit/risk profile for vamorolone.

# 2 STUDY OBJECTIVES AND ENDPOINTS

## 2.1 Study Objectives

### *2.1.1 Primary Objectives*

The primary objectives of this study are:

1. To compare the efficacy of vamorolone administered orally at daily doses of 2.0 mg/kg and 6.0 mg/kg over a 24-week treatment period vs. placebo in ambulant boys ages 4 to <7 years with DMD; and
2. To evaluate the safety and tolerability of vamorolone administered orally at daily doses of 2.0 mg/kg and 6.0 mg/kg in ambulant boys ages 4 to <7 years with DMD.

### *2.1.2 Secondary Objectives*

The secondary objectives of this study are:

1. To compare the safety of vamorolone administered orally at daily doses of 2.0 mg/kg and 6.0 mg/kg over a 24-week treatment period vs. daily prednisone 0.75 mg/kg in ambulant boys ages 4 to <7 years with DMD;
2. To compare the efficacy of vamorolone administered orally at daily doses of 2.0 mg/kg and 6.0 mg/kg over a 24-week treatment period vs. daily prednisone 0.75 mg/kg in ambulant boys ages 4 to <7 years with DMD;
3. To compare the efficacy of vamorolone administered orally at daily doses of 2.0 mg/kg vs. vamorolone administered orally at daily doses of 6.0 mg/kg over a 24-week treatment period in ambulant boys ages 4 to <7 years with DMD;
4. To compare the efficacy of vamorolone administered orally at daily doses of 2.0 mg/kg and 6.0 mg/kg over a 48-week treatment period in ambulant boys ages 4 to <7 years with DMD vs. untreated DMD historical controls;
5. To compare the safety of vamorolone administered orally at daily doses of 2.0 mg/kg and 6.0 mg/kg over a 48-week treatment period in ambulant boys ages 4 to <7 years with DMD vs. prednisone-treated DMD historical controls; and
6. To evaluate the population pharmacokinetics of vamorolone administered orally at daily doses of 2.0 mg/kg and 6.0 mg/kg in ambulant boys ages 4 to <7 years with DMD.

### *2.1.3 Exploratory Objectives*

The exploratory objectives of this study are:

1. To evaluate the satisfaction with treatment of vamorolone administered orally at daily doses of 2.0 mg/kg and 6.0 mg/kg over a 24-week treatment period vs. daily prednisone 0.75 mg/kg in ambulant boys ages 4 to <7 years with DMD;
2. To evaluate the effect of vamorolone administered orally at daily doses of 2.0 mg/kg and 6.0 mg/kg over a 24-week treatment period vs. daily prednisone 0.75 mg/kg on behavior and neuropsychology;

3. To evaluate the effect of vamorolone administered orally at daily doses of 2.0 mg/kg and 6.0 mg/kg over a 24-week treatment period vs. placebo on physical functioning;
4. To assess the ease of administration of the study medication suspension to ambulant boys ages 4 to <7 years with DMD;
5. To compare the effects of vamorolone administered orally at daily doses of 2.0 mg/kg and 6.0 mg/kg over a 24-week treatment period vs. placebo on potential serum PD biomarkers of safety and efficacy in ambulant boys ages 4 to <7 years with DMD;
6. To compare the effects of vamorolone administered orally at daily doses of 2.0 mg/kg and 6.0 mg/kg over a 24-week treatment period vs. daily prednisone 0.75 mg/kg on potential serum PD biomarkers of safety and efficacy in ambulant boys ages 4 to <7 years with DMD; and
7. To determine if candidate genetic modifiers of DMD (gene polymorphisms associated with disease severity, or response to glucocorticoid treatment) are similarly associated with vamorolone-treated DMD patients (baseline disease severity, or response to vamorolone or prednisone treatment).

## 2.2 Study Endpoints

### *2.2.1 Safety Endpoints*

1. BMI z-score: Comparison of each vamorolone dose level group with the prednisone group in change from baseline to each of the scheduled on-treatment and post-treatment assessment time points.
2. Treatment-emergent adverse events (TEAEs) and serious adverse events (SAEs) by system organ class (SOC): Overall by treatment, by treatment and relationship, and by treatment and intensity (see **Section 7.5**);
3. Vital signs (sitting blood pressure, heart rate, respiratory rate, and body temperature): Change from baseline to each of the scheduled on-treatment and post-treatment assessment time points;

4. Body weight and height: Change from baseline to each of the scheduled on-treatment and post-treatment assessment time points;
5. Cushingoid features: Change from baseline to each of the scheduled on-treatment and post-treatment assessment time points (changes from baseline will be recorded as AEs);
6. Clinical laboratory values: Change from baseline to each of the scheduled on-treatment and post-treatment assessment time points in:
    - Hematology and clinical chemistry
    - Lipid profile (triglycerides, total cholesterol, low density lipoprotein [LDL], high density lipoprotein [HDL])
    - Vitamin D level
    - Urinalysis;
7. 12-lead electrocardiogram (ECG): Change from baseline to each of the scheduled on-treatment and post-treatment assessment time points;
8. 2D-echocardiogram: Change from baseline to Week 24 and Week 48;
9. Dual-energy x-ray absorptiometry (DXA) scan: Change from baseline to Week 24 and Week 48 in spine BMD, spine BMD z-score, total body BMD, spine and total body bone mass, and total body composition (lean mass, fat mass, fat-free mass, Lean Mass Index, and Fat Mass Index);
10. Spine x-rays: Change from baseline to Week 24 assessment;
11. Eye examination for detection of clinically significant abnormalities (cataracts and/or glaucoma) at Week 24 and Week 48 assessments compared to baseline;
12. ACTH Stimulation Test: measure of adrenal suppression at Week 24 and Week 48. Adrenal suppression is likely if cortisol levels <18 µg/dL (or 500 nM) 30 or 60 minutes after stimulation with Cosyntropin.

Data for the following additional safety outcomes will be listed only:

1. Physical examination findings at each of the pretreatment, on-treatment, and post-treatment assessment time points.
2. Fracture Questionnaire results at pretreatment, Week 24, and Week 48.

### 2.2.2 Clinical Efficacy Endpoints

#### Primary Clinical Efficacy Endpoint

1. Time to Stand Test (TTSTAND) velocity (rise/second): Comparison of each vamorolone dose level group versus the placebo group in change from baseline to the Week 24 assessment.

#### Secondary Efficacy Endpoints

1. Change from baseline to each of the scheduled study assessment time points for each treatment group up to Week 48, with comparison of each vamorolone dose level group versus the placebo group at each of the scheduled study assessment time points up to and including Week 24 for:
    - Time to Stand Test (TTSTAND) velocity (rise/second) (other than Week 24);
    - Time to Climb (4 Steps) Test (TTCLIMB) velocity (tasks/second);
    - Time to Run/Walk Test (TTRW) velocity (meters/second) to complete 10 meters of a 14 meter course;
    - Total distance traveled, in meters, in completing the Six-minute Walk Test (6MWT);
    - North Star Ambulatory Assessment (NSAA);
    - Hand-held myometry (elbow flexors and knee extensors); and
    - Range of motion in the ankles (ROM).
2. Change from baseline with comparison of each vamorolone dose level group versus the prednisone group at each of the scheduled study assessment time points up to and including Week 24 for:

- Total distance traveled, in meters, in completing the Six-minute Walk Test (6MWT).

### 2.2.3 Exploratory Endpoints

1. Treatment satisfaction questionnaire (TSQM): Comparison of each vamorolone dose level group to the prednisone group at the Week 24 visit; comparison of each treatment group at the Week 48 visit;
2. Pediatric Outcomes Data Collection Instrument (PODCI): Comparison of each vamorolone dose level group to the placebo group for change from baseline to the Week 24 assessment; comparison of each treatment group for change from baseline to the Week 48 assessment;
3. Behavioral changes (PARS III): Comparison of each vamorolone dose level group to the prednisone group for change from baseline to each of the scheduled study assessment time points up to the Week 24 assessment; comparison of each treatment group for change from baseline to the Week 48 assessment;
4. Ease of study medication administration assessed at each of the scheduled study assessment time points;
5. Blindedness Assessment at each of the scheduled study assessment time points; and
6. DNA testing for candidate genetic modifiers of DMD.

### 2.2.4 Pharmacodynamic Endpoints

1. The following pharmacodynamic biomarkers are considered secondary outcome measures focusing on safety outcomes. In each case, the biomarkers studied reflect safety concerns of glucocorticoids:
    a. Adrenal suppression. First-in-morning serum cortisol levels will be measured. Cortisol measures falling below 3.6 μg/dL (or 100 nM) will be considered to be indicative of the development of adrenal suppression. ACTH Stimulation Test will be performed at the Screening Visit and at

the Week 24 Follow-up Visit (48 ± 3 hours after the final dose of Treatment Period #1 study medication) and at the Week 48 Follow-up Visit (48 ± 3 hours after the final dose of Treatment Period #2 study medication): cortisol levels <18 µg/dL (or 500 nM) 30 or 60 minutes after stimulation with Cosyntropin (250 µg) will be considered to be indicative of adrenal suppression.

b. Bone turnover. Measures of serum osteocalcin are reflective of bone formation, and measures of serum CTX1 are reflective of bone reabsorption. Ratios of osteocalcin and CTX1 predict later clinical safety concerns of osteopenia and bone fragility.

c. Insulin resistance. Glucocorticoids cause both acute and chronic insulin resistance, with serum elevations of both insulin and glucose. Measures of hyperinsulinemia and hyperglycemia are accepted measures of insulin resistance.

d. Immune suppression. Glucocorticoids can cause immunosuppression. Measure of differential lymphocyte percentage can be a biomarker for immune suppression.

2. Exploratory biomarkers for aspects of safety and efficacy[31]

### 2.2.5 Endpoints for Patient-Reported Outcomes

Safety endpoints based on subject reports of AEs are listed in **Section 2.2.1**.

The parent/legal guardian of each subject will be asked to assess the ease of administration of the study medication suspension (see **Section 7.4.4**).

Additionally, subjects' parents/legal guardians will be asked to complete the PODCI (see **Section 7.4.1**). Satisfaction with treatment will be measured using the Treatment Satisfaction Questionnaire (TSQM) which also will be completed by the parent/legal guardian (see **Section 7.4.2**). Additionally, the PARS III behavioral assessment will be completed by the parent/guardian (see **Section 7.4.3**). Finally, the subject's parent/legal

guardian will complete a Blindedness Assessment at the Week 24 Visit (see **Section 7.4.5**).

No other patient-reported outcomes are planned.

# 3 STUDY DESIGN

## 3.1 Overall Study Design

This Phase IIb study is a randomized, double-blind, parallel group, placebo- and active-controlled study with double-blind extension to evaluate the long-term efficacy, safety, tolerability, PD, and population PK of vamorolone (the investigational medicine) compared to prednisone (active control) and placebo over a Treatment Period of 24 weeks in boys ages 4 to <7 years with DMD, and determine the persistence of effect over a total Treatment Period of 48 weeks. Six treatment groups will receive either vamorolone at one of two doses (2.0 mg/kg or 6.0 mg/kg), prednisone (0.75 mg/kg), or placebo once daily for 24 weeks, and will receive vamorolone at one of two doses (2.0 mg/kg or 6.0 mg/kg) daily for an additional 20 weeks. A total of approximately 120 subjects will be randomized into the study as shown in **Table 6**.

To maintain the blind during Treatment Period #1, matched suspension (vamorolone or placebo) and matched tablets (prednisone or placebo) will be administered. Each subject will receive a dose of suspension (vamorolone or placebo) and tablets (prednisone and placebo) once daily during Treatment Period #1. The number of tablets and volume of suspension per dose will be determined by body weight.

The study is comprised of a Pretreatment Screening Period of up to 32 days duration, a 1-day Pretreatment Baseline Period, a 24-week Treatment Period #1, a 4-week Transition Period, a 20-week Treatment Period #2, and a 4-week Dose-tapering Period. Subjects will be enrolled into this study at the time written informed consent is given, and randomized to treatment only after completion of all Pretreatment Screening assessments.

Study drug dosing will occur from Day 1 until the Week 48 Visit (**Section 5.3**). Study drug dosing will occur at home on all days except the Day 1, Week 2, Week 12, Week

24, Week 28, Week 30, Week 40, and Week 48 study visits, when dosing will occur at the study site.

Subjects will be assessed for safety and tolerability, clinical efficacy, PD, and population PK at scheduled visits throughout the study (see **Section 6** for a schedule of study assessments). Treatment Period #1, Transition Period, and Treatment Period #2 study visits will occur at Day 1, Week 2, Week 6, Week 12, Week 18, Week 24, Week 28, Week 30, Week 34, Week 40 and Week 48 (**Table 11**); all subjects will return to the clinical site for a Week 24 Follow-up Visit and for a Week 48 Follow-up Visit, 48 ± 3 hours after administration of the final dose of Treatment Period #1 and Treatment Period #2 study medication, respectively, for ACTH Stimulation testing. Adverse events, including SAEs, and concomitant medications will be recorded throughout the study.

Subject diaries will be dispensed at the Day 1 Visit and at each study visit thereafter through Week 48 to record AEs, changes to concomitant medications taken during the study, and any missed or incomplete doses of study medication.

There is flexibility in the timing of completion of some of the scheduled Week 24 and Week 48 assessments. The scheduled physical examination, weight, vital signs, clinical laboratory tests, blood draws for PD biomarker analysis, blood draw for DNA testing (Week 24 only), Ease of Study Medication Administration Assessment, PODCI, PARS III, and functional assessments (TTSTAND, TTCLIMB, TTRW, NSAA, 6MWT, hand-held myometry, ROM) must all be performed on the date of the Week 24 or Week 48 dose of study medication. The 12-lead ECG may be performed on the date of the Week 24 or Week 48 dose of study medication, the day following the Week 24 or Week 48 dose of study medication, or the day of the Week 24 or Week 48 Follow-up Visit. For the Week 24 assessments, completion of the DXA scan, spine X-rays, Fracture Questionnaire, 2-D echocardiography, eye examination, TSQM, and Blindedness Assessment may be performed up to 7 days following the date of the Week 24 dose of study medication to accommodate need for additional scheduling flexibility. For the Week 48 assessments, completion of the DXA scan, Fracture Questionnaire, 2-D echocardiography, eye examination, and TSQM may be performed on the date of the

final Week 48 dose of study medication, the day following the Week 48 dose of study medication, or the day of the Week 48 Follow-up Visit for subjects who will receive additional vamorolone therapy by enrolling directly into an additional vamorolone study or general access program, or up to 7 days following the date of the final Week 48 dose of study medication for subjects participating in the Dose-tapering Period.

A Transition Period of 4 weeks in duration follows the end of Treatment Period #1 for all subjects. During this Transition Period, all subjects will continue to receive the liquid formulation (vamorolone 2.0 mg/kg or 6.0 mg/kg, or matching placebo) they received during Treatment Period #1 and will be tapered off their study medication tablets (prednisone or matching placebo). Site study staff will contact the parent(s)/guardian(s) by telephone at Week 26 to ensure that the tablet tapering is proceeding according to protocol, to assess potential signs or symptoms indicative of adrenal suppression, and to address any questions the parent(s)/guardian(s) may have. All subjects will return to the clinical site for the Week 28 assessments, prior to receiving their first dose of Treatment Period #2 study medication on the day after the Week 28 Visit (Week 28 + 1 day) (see **Section 6.3.6**).

Subjects who complete the VBP15-004 study assessments through the Week 48 Visit and Week 48 Follow-up Visit may be given access to vamorolone through an additional study or general access program, or given the option to transition to standard of care treatment (including glucocorticoids) for DMD. Standard of care treatment for DMD may be offered to the subject following completion of the Phase IIb VBP15-004 study, if the subject’s parent or guardian does not wish to enroll the subject in the additional vamorolone study or general access program and/or the Investigator feels it to be in the best interest of the subject.

Subjects who will enroll directly into the additional vamorolone study or general access program to continue vamorolone treatment will be discharged from the VBP15-004 study following completion of all Week 48 assessments and the Week 48 Follow-up Visit ACTH Stimulation Test. Subjects who will not continue vamorolone treatment in the additional vamorolone study or general access program, including those subjects who

will transition to standard of care treatment for DMD, will have their vamorolone dose tapered during a 4-week Dose-tapering Period, prior to discharge from the study. Site study staff will contact the parent(s)/guardian(s) by telephone at Week 50 to ensure that the dose tapering is proceeding according to protocol (see **Section 6.3.7**), to assess potential signs or symptoms of adrenal suppression, and to address any questions the parent(s)/guardian(s) may have.

In the event that any clinical or laboratory parameters remain abnormal at the time of discharge from the study, the subject will be followed medically, as clinically indicated.

Any subject who discontinues the study prior to the Week 24 Visit should return to the study unit for scheduled Week 24 assessments and the Week 24 Follow-up Visit ACTH Stimulation Test at the time of early withdrawal, whenever possible; any subject who prematurely discontinues the study after Week 24 but prior to Week 28 should complete the scheduled Week 28 assessments at the time of early withdrawal, whenever possible; and any subject who prematurely discontinues the study after Week 28 but prior to Week 48 should complete the scheduled Week 48 assessments and the Week 48 Follow-up Visit ACTH Stimulation Test at the time of early withdrawal, whenever possible, assuming the subject has not withdrawn consent. Any subject who withdraws early from the study should undergo Early Discontinuation Dose-tapering (see **Section 6.4**).

## 3.2 Randomization

Following consent and review of study entry criteria to confirm subject eligibility for the study, the subject can be randomized to treatment. Randomization should be performed at least 10 days prior to the baseline visit and will be achieved via the Interactive Voice/Web Response System (IXRS) system with user name and password access. Randomization will be stratified by participant's age (<6 vs. ≥ 6 years). Randomization will be stratified only by age; randomization will not be stratified by investigational site. Randomization will require the site investigator, or designee, to verify that the subject meets the inclusion/exclusion criteria of the study, and to verify that the child has not previously been randomized. The following information will need to be entered into the

IXRS system in order to assign the subject to a treatment group and the appropriate age stratification group:

- Participant's Study Subject Identification Number
- Participant's Date of Birth
- Participant's weight, as recorded at the Screening Visit.

When the site investigator/designee completes the randomization procedures via the IXRS system, an e-mail report with the randomization number and age stratification group will be generated and sent to the clinical trials supply company confirming randomization into the trial.

The randomization number will be assigned by the IXRS system and will only be used for study drug supply and shipment. Once the information has been received, the clinical trials supply company will prepare a subject-specific kit of study medication and ship it to the pharmacy at the study site.

Randomization procedures should be completed at least 10 days prior to the Baseline Visit in order to allow study drug supply to be shipped to the site in time to be dispensed to the participant by the site investigator for Day 1 dosing.

## 3.3 Blinding

To achieve double-blinding, the supplies company will manufacture identical liquid formulations for vamorolone and placebo, and identical tablet formulation for prednisone and placebo. Each dose of study medication will consist of doses of both a liquid formulation (vamorolone or placebo) and tablets (prednisone or placebo) through Week 28, and liquid formulation only following Week 28 through Week 48 (see **Table 7**).

To blind the liquid formulation, the supplies company will manufacture 1.33% and 4.0% wt/wt vamorolone suspension formulations, and placebo suspension formulation, identical in appearance. Subjects who are randomized to receive vamorolone 2.0 mg/kg will receive the 1.33% vamorolone suspension, and subjects who are randomized to receive vamorolone 6.0 mg/kg will receive the 4.0% vamorolone suspension. Study medication will be shipped to the sites in 100 mL bottles labeled with subject-specific

identifiers. Trained site study staff will calculate study medication dose volume based on subject body weight for all subjects as 0.15 mL/kg, regardless of treatment assignment.

Subjects, parents/guardians, site investigators and all other site study staff will not know to which treatment group the subject has been assigned and will remain blinded to the identity of the treatment assignment until the end of the study (last subject last visit) and the database has been locked.

## 3.4 Unblinding

Every attempt should be made to preserve the integrity of study drug blinding. All subjects will be provided with a card, to be carried at all times, stating “I am taking part in a clinical trial” (in the local language) to be presented to medical staff in the event of routine treatment or a medical emergency. Investigational medications can usually be withdrawn without the need for unblinding in a subject experiencing an AE that requires study medication withdrawal. In this case, the site investigator should provide adequate and necessary support to the subject without unblinding study treatment.

Subjects who experience a medical emergency, whether their treatment remains blinded or is unblinded, should be covered with stress steroids, except for unblinded subjects who were receiving placebo.

All subjects who have study medication withdrawn without unblinding will need to undergo dose-tapering according to the schedule in **Section 6.4**.

In the event that unblinding is necessary, an emergency unblinding procedure is provided to allow site investigators to disclose a treatment assignment for an individual subject if clinical circumstances should require this.

Unblinding will be performed and treatment assignment obtained through the IXRS system with username and password code. The expectation is that emergency unblinding will occur only very rarely; for example, when the subject needs emergency surgery and information about all treatment interventions is required. In the exceptional circumstance that knowledge of the study drug assignment appears essential for providing appropriate medical management, the site investigator should make every effort to contact the study

chair or the independent Medical Monitor to discuss the rationale for breaking the blind. If the site investigator still believes that unblinding is needed, or the study chair and independent Medical Monitor are not available for contact, the site investigator will follow the IXRS unblinding procedures for unblinding the subject in question (see the Manual of Operations for details on how to unblind a subject using the IXRS system).

After breaking the blind, the site staff should record details regarding the reasons for breaking the blind, including any AEs leading to the unblinding, in the source documents and electronic case report form (eCRF). Once the blind is broken for a given subject, study drug will be discontinued and the subject will be withdrawn from the study. Furthermore, as the subject might have received glucocorticoids (prednisone) as part of the study and as vamorolone appears to affect the adrenal axis, prednisone and vamorolone cannot be discontinued without a proper dose-tapering period.

Any subject whose treatment is unblinded prior to the Week 24 Visit should return to the clinic for Week 24 assessments at the time of unblinding, whenever possible, assuming the subject has not withdrawn consent; any subject whose treatment is unblinded after Week 24 and prior to the Week 28 Visit should return to the clinic for Week 28 assessments at the time of unblinding, whenever possible, assuming the subject has not withdrawn consent; any subject whose treatment is unblinded after Week 28 and prior to the Week 48 Visit should return to the clinic for Week 48 assessments at the time of unblinding, whenever possible, assuming the subject has not withdrawn consent. Since all subjects will receive vamorolone at one of two dose levels during Treatment Period #2 (Weeks 28 – 48), the identification, through unblinding, of the vamorolone dose level to which a given subject is assigned is unlikely to give additional useful information, and thus the need to unblind treatment assignment is remote during this study period. Any subject whose treatment, upon unblinding, is revealed to be either vamorolone or prednisone, will need to follow the dose-tapering protocol (**Section 6.4** specific to the time of unblinding); these subjects will also be asked to return to the clinic at the end of the tapering period for final Dose-tapering Period assessments, whenever possible. If the subject was taking placebo prior to unblinding, study drug tapering will not be required; however, the subject should return for final Week 24 assessments at the time of

unblinding and withdrawal from the study. After unblinding, a subject may be prescribed standard of care glucocorticoids, if clinically indicated.

Subjects who discontinue the study for any reason other than a medical emergency will not be unblinded.

# 4 SELECTION AND WITHDRAWAL OF STUDY SUBJECTS

## 4.1 Subject Enrollment and Identification Log

Subjects will be recruited through the clinics of participating site investigators and other mechanisms including patient registries, national and international networks and patient foundations. After identification of a possible subject, the site investigator will discuss the study with the subject's parent(s) or legal guardian(s). The subject's parent(s) or guardian(s) will be provided with a copy of the study subject information sheet document and allowed time to consider participation prior to signing. Individuals interested in participating will be asked to come to one of the participating study sites to complete the informed consent process with a site investigator or designee prior to initiation of screening procedures. Subjects will not be excluded on the basis of race, ethnicity, or age, except that the target population for the trial is 4 to <7 years of age.

A subject enrollment log will be maintained at each investigational site for all subjects who are screened for the study, including those not randomized to treatment. Limited data will be collected for these subjects, including date of birth, and reason for exclusion from the study. Subject enrollment logs will be maintained for all subjects enrolled in the study. This record will also include the dates of subject enrollment and completion/termination.

The Site Investigator will keep a record relating the names of the subjects to their enrollment numbers (subject identification log) to permit efficient verification of data subject files, when required. These logs will be reviewed during routine monitoring calls and/or visits.

## 4.2 Inclusion Criteria

To qualify for randomization in this study, the subject must satisfy the following inclusion criteria:

1. Subject’s parent(s) or legal guardian(s) has (have) provided written informed consent and Health Insurance Portability and Accountability Act (HIPAA) authorization, where applicable, prior to any study-related procedures; participants will be asked to give written or verbal assent according to local requirements;
2. Subject has a centrally confirmed (by TRiNDS central genetic counselor[s]) diagnosis of DMD, as defined as:
   - Dystrophin immunofluorescence and/or immunoblot showing complete dystrophin deficiency, and clinical picture consistent with typical DMD, OR
   - Identifiable mutation within the DMD gene (deletion/duplication of one or more exons), where reading frame can be predicted as 'out-of-frame,' and clinical picture consistent with typical DMD, OR
   - Complete dystrophin gene sequencing showing an alteration (point mutation, duplication, other) that is expected to preclude production of the dystrophin protein (i.e., nonsense mutation, deletion/duplication leading to a downstream stop codon), with a clinical picture consistent with typical DMD;
3. Subject is ≥ 4 years and <7 years of age at time of enrollment in the study;
4. Subject weighs >13.0 kg and ≤ 39.9 kg at the Screening Visit;
5. Subject is able to walk independently without assistive devices;
6. Subject is able to complete the Time to Stand Test (TTSTAND) without assistance in <10 seconds, as assessed at the Screening Visit;
7. Clinical laboratory test results are within the normal range at the Screening Visit, or if abnormal, are not clinically significant, in the opinion of the Investigator. [Note: Serum gamma glutamyl transferase (GGT), creatinine, and total bilirubin all must be ≤ upper limit of the normal range at the Screening Visit];

8. Subject has evidence of chicken pox immunity as determined by presence of IgG antibodies to varicella, as documented by a positive test result from the local laboratory at the Screening Visit;
9. Subject is able to swallow tablets, as confirmed by successful test swallowing of placebo tablets during the Screening Period; and
10. Subject and parent(s)/guardian(s) are willing and able to comply with scheduled visits, study drug administration plan, and study procedures.

## 4.3 Exclusion Criteria

A subject will be excluded from randomization in this study if he meets any of the following exclusion criteria:

1. Subject has current or history of major renal or hepatic impairment, diabetes mellitus or immunosuppression;
2. Subject has current or history of chronic systemic fungal or viral infections;
3. Subject has had an acute illness within 4 weeks prior to the first dose of study medication;
4. Subject has used mineralocorticoid receptor agents, such as spironolactone, eplerenone, canrenone (canrenoate potassium), prorenone (prorenoate potassium), mexrenone (mexrenoate potassium) within 4 weeks prior to the first dose of study medication;
5. Subject has a history of primary hyperaldosteronism;
6. Subject has evidence of symptomatic cardiomyopathy [Note: Asymptomatic cardiac abnormality on investigation would not be exclusionary];
7. Subject is currently being treated or has received previous treatment with oral glucocorticoids or other immunosuppressive agents [Notes: Past transient use of oral glucocorticoids or other oral immunosuppressive agents for no longer than 1 month cumulative, with last use at least 3 months prior to first dose of study medication, will be considered for eligibility on a case-by-case basis, unless discontinued for intolerance. Inhaled and/or topical glucocorticoids are permitted

if last use is at least 4 weeks prior to first dose of study medication or if administered at stable dose beginning at least 4 weeks prior to first dose of study medication and anticipated to be used at the stable dose regimen for the duration of the study];

8. Subject has an allergy or hypersensitivity to the study medication or to any of its constituents;
9. Subject has used idebenone within 4 weeks prior to the first dose of study medication;
10. Subject has severe behavioral or cognitive problems that preclude participation in the study, in the opinion of the Investigator;
11. Subject has previous or ongoing medical condition, medical history, physical findings or laboratory abnormalities that could affect safety, make it unlikely that treatment and follow-up will be correctly completed or impair the assessment of study results, in the opinion of the Investigator;
12. Subject is taking (or has taken within 4 weeks prior to the first dose of study medication) herbal remedies and supplements which can impact muscle strength and function (e.g., Co-enzyme Q10, Creatine, etc);
13. Subject is taking (or has taken within 3 months prior to the first dose of study medication) any medication indicated for DMD, including Exondys51 and Translarna;
14. Subject has been administered a live attenuated vaccine within 14 days prior to the first dose of study medication;
15. Subject is currently taking any other investigational drug or has taken any other investigational drug within 3 months prior to the first dose of study medication;
16. Subject has a sibling who is currently enrolled in any vamorolone study or Expanded Access Program, or who intends to enroll in any vamorolone study or Expanded Access Program during the subject's participation in the VBP15-004 study; or
17. Subject has previously been enrolled in the study.

Note: Any parameter/test may be repeated at the Investigator's discretion during Screening to determine reproducibility. In addition, subjects may be rescreened if ineligible due to a transient condition which would prevent the subject from participating, such as an upper respiratory tract infection or injury, or if ineligible due to negative anti-varicella IgG antibody test result.

## 4.4 Withdrawal of Subjects from Study

A subject may withdraw from the study, or may be withdrawn by his parent or guardian at any time without the need to justify the decision.

The Investigator has the right to terminate participation of a subject in the study for any of the following reasons:

- The subject's parent/legal guardian is uncooperative/noncompliant and does not adhere to study responsibilities, including failure to attend study visits;
- Difficulty in obtaining blood samples from the subject for safety monitoring;
- The subject experiences an unmanageable or non-tolerable AE/SAE which is considered to be possibly, probably, or definitely related to study drug, in the opinion of the Investigator, and may jeopardize the subject's health;
- The Sponsor terminates the study;
- Any other reason relating to subject safety or integrity of the study data;
- The subject is unblinded to study treatment.

In the event a subject is withdrawn from the study, the Sponsor or designee (e.g., Coordinating Center) will be informed within one business day. If there is a medical reason for withdrawal, the subject will remain under the supervision of the Investigator until resolution of the event.

All subjects who withdraw from the study prior to the Week 24 Visit should return to the study site for Week 24 assessments and the Week 24 Follow-up Visit ACTH Stimulation Test at the time of early withdrawal and undergo Dose-tapering, where possible (see **Section 6.4.1** and **Section 7.2.7**), subjects who prematurely discontinue

from the study after Week 24 but prior to Week 28 should complete the Week 28 assessments at the time of early withdrawal and undergo Dose-tapering, where possible (see **Section 6.4.2**), and subjects who prematurely discontinue from the study after Week 28 but prior to Week 48 should complete the Week 48 assessments and the Week 48 Follow-up Visit ACTH Stimulation Test at the time of early withdrawal and undergo Dose-tapering, where possible (see **Section 6.3.7** and **Section 7.2.7**), assuming the subject has not withdrawn consent. Subjects will also be asked to come back at the end of the tapering period for a follow up visit. In the event a subject withdraws informed consent, no further study procedures should be performed and no additional data should be collected. Any data collected up to the point of withdrawal of informed consent may be used by the Sponsor.

## 4.5 Termination of Study

This study may be prematurely terminated if, in the opinion of the Sponsor, there is sufficient reasonable cause. An example of a circumstance that may warrant termination is determination of unexpected, significant, or unacceptable risks to participants.

If the study is prematurely terminated or suspended, the Sponsor will promptly inform the site Investigators and the regulatory authority(ies) of the termination or suspension and the reason(s) for the termination or suspension. The Institutional Review Board(s) (IRB[s])/Independent Ethics Committee(s) (IEC[s]) will also be informed promptly by the Investigator/institution or the Sponsor and provided the reason(s) for the termination or suspension.

Subject enrollment at a given site may be terminated by the Sponsor. Possible reasons for termination of the study at a given site include, but are not limited to:

1. Unsatisfactory enrollment with respect to quantity or quality
2. Inaccurate or incomplete data collection
3. Falsification of records
4. Failure to adhere to the protocol.

Subjects who are participating at a given site at the time it is terminated by the Sponsor will be offered the opportunity to continue to participate in the study at an alternative active site. Subjects who decline the offer to participate at an alternative active site will need to undergo dose tapering at the time the original site is terminated according to the dose-tapering schedule (see **Section 6.4**).

# 5 TREATMENT OF STUDY SUBJECTS

## 5.1 Study Medications Administered

### *5.1.1 Study Medications Administered During Treatment Period #1*

Vamorolone 1.33% wt/wt or 4.0% wt/wt oral suspension (investigational medicine), prednisone (active control) or placebo will be administered once daily over the 24-week Treatment Period #1.

There are six treatment groups in this study. The oral suspensions and tablets for Treatment Period #1 are shown in **Table 7** for subjects who will be randomly assigned (2:2:1:1:1:1) to the following six treatment groups.

**Table 7. Study Medications for the Six Treatment Groups During Treatment Period #1**

| Treatment Group | Study Medications | |
|---|---|---|
| | Oral Suspension | Tablets |
| Treatment Group 1 | 2.0 mg/kg vamorolone | placebo |
| Treatment Group 2 | 6.0 mg/kg vamorolone | placebo |
| Treatment Group 3 | placebo | 0.75 mg/kg prednisone |
| Treatment Group 4 | placebo | 0.75 mg/kg prednisone |
| Treatment Group 5 | placebo | placebo |
| Treatment Group 6 | placebo | placebo |

Vamorolone will be administered as a 1.33% wt/wt suspension for oral dosing at the planned dose level of 2.0 mg/kg and as a 4.0% wt/wt suspension for oral dosing at the planned dose level of 6.0 mg/kg. Prednisone will be administered as tablets for oral dosing at a dose of 0.75 mg/kg. Prednisone tablets will be dispensed at a dosage strength

of 5 mg/tablet. To maintain the blind, matched suspension (vamorolone or placebo) and tablets (prednisone or placebo) have been produced. Each subject will receive a dose of suspension (0.15 mL/kg of vamorolone [1.33% oral suspension for the 2.0 mg/kg dose level or 4.0% oral suspension for the 6.0 mg/kg dose level] or placebo) and tablets (prednisone or placebo) each day (see **Section 3.3**). The number of tablets per dose will be determined by body weight (see **Table 10**).

The clinical trials supplies companies will manufacture identical liquid formulation of vamorolone and placebo and identical tablets for prednisone and placebo to maintain the blind. Liquid solution of prednisone could not have been matched for color and flavor with vamorolone to maintain the blind. Therefore, a double-dummy design is being used in this study.

### *5.1.2 Study Medications Administered During Transition Period*

Vamorolone 1.33% wt/wt or 4.0% wt/wt oral suspension (investigational medicine), prednisone (active control) or placebo will be administered once daily over the 4-week Transition Period. Prednisone and placebo tablets will be tapered over the 4-week Transition Period (see **Table 12**).

The oral suspensions and tablets for the Transition Period are shown in **Table 8** for subjects in each of the six treatment groups.

**Table 8. Study Medications for the Six Treatment Groups During the Transition Period**

| Treatment Group | Study Medications | |
|---|---|---|
| | Oral Suspension | Tablets (Tapering Doses) |
| Treatment Group 1 | 2.0 mg/kg vamorolone | placebo |
| Treatment Group 2 | 6.0 mg/kg vamorolone | placebo |
| Treatment Group 3 | placebo | prednisone |
| Treatment Group 4 | placebo | prednisone |
| Treatment Group 5 | placebo | placebo |
| Treatment Group 6 | placebo | placebo |

Vamorolone will continue to be administered as a 1.33% wt/wt suspension for oral dosing at the planned dose level of 2.0 mg/kg and as a 4.0% wt/wt suspension for oral dosing at the planned dose level of 6.0 mg/kg. Prednisone will be administered as tablets for oral dosing with decreasing number of tablets over the 4-week Transition Period (**Table 12**). Prednisone tablets will be dispensed at a dosage strength of 5 mg/tablet. To maintain the blind, each subject will receive a dose of suspension (0.15 mL/kg of vamorolone [1.33% oral suspension for the 2.0 mg/kg dose level or 4.0% oral suspension for the 6.0 mg/kg dose level] or placebo) and tablets (prednisone or placebo) each day (see **Section 3.3**). The number of tablets per dose will be tapered to zero (0) over the 4-week Transition Period (see **Table 12**).

### *5.1.3 Study Medications Administered During Treatment Period #2 and the Dose-tapering Period*

Vamorolone 1.33% wt/wt or 4.0% wt/wt oral suspension (investigational medicine) will be administered once daily over the 20-week Treatment Period #2, and during the 4-week Dose-tapering Period, as applicable. No study drug tablets are administered during Treatment Period #2 or the Dose-tapering Period.

The oral suspensions for Treatment Period #2 are shown in **Table 9** for subjects in each of the six treatment groups.

**Table 9. Study Medications for the Six Treatment Groups During Treatment Period #2 and the Dose-tapering Period**

| Treatment Group | Study Medications |
|---|---|
| | Oral Suspension |
| Treatment Group 1 | 2.0 mg/kg vamorolone |
| Treatment Group 2 | 6.0 mg/kg vamorolone |
| Treatment Group 3 | 2.0 mg/kg vamorolone |
| Treatment Group 4 | 6.0 mg/kg vamorolone |
| Treatment Group 5 | 2.0 mg/kg vamorolone |
| Treatment Group 6 | 6.0 mg/kg vamorolone |

Vamorolone will be administered as a 1.33% wt/wt suspension for oral dosing at the planned dose level of 2.0 mg/kg and as a 4.0% wt/wt suspension for oral dosing at the planned dose level of 6.0 mg/kg. During Treatment Period #2, each subject will receive a dose of suspension (0.15 mL/kg of vamorolone [1.33% oral suspension for the 2.0 mg/kg dose level or 4.0% oral suspension for the 6.0 mg/kg dose level] each day (see **Section 3.3**). The dose of suspension study medication will be tapered according to the schedule outlined in **Section 6.3.7** during the Dose-tapering Period.

## 5.2 Identity of Investigational Product

ReveraGen BioPharma, Inc. will supply the following investigational study medications:

Vamorolone

| | |
|---|---|
| Active Substance: | Vamorolone |
| Strength: | 1.33% wt/wt and 4.0% wt/wt |
| Dosage Form: | Oral suspension |
| Manufacturer: | Velesco Pharmaceutical Services |

Prednisone

| | |
|---|---|
| Active Substance: | Prednisone |
| Strength: | 5 mg |
| Dosage Form: | Tablet |
| Manufacturer: | Piramal Healthcare UK Limited |

Placebo to Match Vamorolone

| | |
|---|---|
| Dosage Form: | Oral suspension |
| Manufacturer: | Velesco Pharmaceutical Services |

Placebo to Match Prednisone

| | |
|---|---|
| Dosage Form: | Tablet |
| Manufacturer: | Piramal Healthcare UK Limited |

## 5.3 Dosage Schedule and Administration of Study Medication

The site pharmacist or designated site study staff will dispense blinded study medication to each subject randomized in the study (see **Section 5.8**). Subjects will receive one of six study medication combinations depending on their treatment group assignment (**Table 7**).

To maintain the study blind, matched suspension (vamorolone or placebo) and tablets (prednisone or placebo) have been produced (see **Section 3.3**). Vamorolone will be administered as a suspension for oral dosing (1.33% wt/wt suspension for the 2.0 mg/kg dose level or as a 4.0% wt/wt suspension for the 6.0 mg/kg dose level) (see Pharmacy Manual for instructions on calculation of suspension dose volume). Prednisone will be administered as 5 mg tablets for oral dosing.

Each subject will receive a dose of suspension (vamorolone or placebo) and tablets (prednisone or placebo) each day during Treatment Period #1, and a dose of suspension (vamorolone or placebo) each day during Treatment Period #2. The number of prednisone or matching placebo tablets per dose will depend upon body weight, as indicated below (**Table 10**). All subjects will receive 0.15 mL/kg per dose of a vamorolone or placebo suspension (**Table 7**).

**Table 10 Weight Bands for Prednisone or Matching Tablet Dosing**

| Band | Weight range in kg | Weight used for calculation of dose per kg | Prednisone dose in mg based on 0.75 mg/kg | Number tablets of prednisone (5 mg) or matching placebo per dose for given weight range |
|---|---|---|---|---|
| A | 13-19.9 | 13.33 kg | 10 mg | 2 |
| B | 20-25.9 | 20.00 kg | 15 mg | 3 |
| C | 26-32.9 | 26.67 kg | 20 mg | 4 |
| D | 33-39.9 | 33.33 kg | 25 mg | 5 |

Subjects will receive study medication, administered orally once daily for 48 weeks, from Study Day 1 to the Week 48 Visit. At the end of the 24-week Treatment Period #1, all

subjects will be tapered off the tablet study medication over a 4-week Transition Period. Those subjects randomized to receive placebo will also undergo tablet dose-tapering to maintain the study blind. At the end of the 20-week Treatment Period #2, subjects who will transition off vamorolone treatment at the end of the study will be tapered off suspension study medication over a 4-week Dose-tapering Period, prior to discharge from the study (see **Section 6.3.7**).

Study medication sufficient for 4, 6, or 8 weeks of dosing (plus overage), depending upon the dispensing interval, will be dispensed by trained study staff at the Day 1 Visit, just prior to dosing, and at Week 6, Week 12, Week 18, Week 24 Follow-up, Week 28, Week 34, Week 40, and Week 48 Follow-up. Each subject's dose (in mL for suspension formulation; in number of tablets for tablet formulation) will be calculated and written on the labels of the bottles and blisters to be dispensed at a given visit by trained site staff based on the weight of the subject (in kg) recorded at the previous visit: weight at Screening will be used to calculate dose of suspension and tablets for drug supply dispensed at Day 1; weight at the Week 2 Visit will be used to calculate dose of suspension and tablets for drug supply dispensed at Week 6; weight at the Week 6 Visit will be used to calculate dose of suspension and tablets for drug supply dispensed at Week 12; weight at the Week 12 Visit will be used to calculate dose of suspension and tablets for drug supply dispensed at Week 18; weight at the Week 18 Visit will be used to calculate dose of suspension and tablets for drug supply dispensed at Week 24 Follow-up for the Transition Period; weight at the Week 24 Visit will be used to calculate dose of suspension for drug supply dispensed at Week 28; weight at the Week 30 Visit will be used to calculate dose of suspension for drug supply dispensed at Week 34; weight at the Week 34 Visit will be used to calculate dose of suspension for drug supply dispensed at Week 40; and weight at the Week 40 Visit will be used to calculate dose of suspension and tablets for drug supply dispensed at the Week 48 Follow-up Visit for the Dose-tapering Period. The dispensed study medication bottle(s) and blister(s) will be returned to the study site at each subsequent scheduled study visit. Study medication suspension and tablets dispensed at the Day 1 Visit should be brought in with the subject to the Week 2 Visit, for Week 2 dosing in-clinic and compliance monitoring; this study

medication will be returned to the subject at the end of the Week 2 Visit for continued dosing through the Week 6 Visit: new study medication will not be dispensed at the Week 2 Visit. In a similar manner, study medication dispensed at the Week 28 Visit should be brought in with the subject to the Week 30 Visit, for Week 30 dosing in-clinic and compliance monitoring; this study medication will be returned to the subject at the end of the Week 30 Visit for continued dosing through the Week 34 Visit: new study medication will not be dispensed at the Week 30 Visit.

Randomized subjects will receive all doses under the supervision of parents or legal guardians or trained study staff. Study drugs will be administered in the study unit at the Day 1, Week 2, Week 12, Week 24, Week 28, Week 30, Week 40, and Week 48 study visits; all other doses will be administered at home. Subjects should receive each dose of study medication in the morning and at approximately the same time of day.

Vamorolone or matching placebo suspension will be administered orally using a volumetric syringe supplied by the site. Following administration of the dose of study drug suspension, the syringe will be filled once with water and the water will be administered by mouth using the volumetric syringe. Prednisone or matching placebo tablets will be taken either immediately before or immediately after the dose of suspension. The subject will then drink approximately 50 mL (approximately 2 ounces) of water to ensure the full dose has been ingested. The dose of study medication should be taken with breakfast, including at least 8 g of fat (approximately 8 ounces [240 mL] of full-fat milk or equivalent high-fat food portion). There are no other food or drink restrictions before or after dosing.

At the Day 1, Week 2, Week 12, Week 24, Week 28, Week 30, Week 40, and Week 48 study visits, subjects will arrive at the study clinic after having fasted for ≥ 6 hours, and will eat breakfast at the study site within 30 minutes prior to administration of the dose of study medication; breakfast at the site will include at least 8 g of fat (8 ounces [240 mL] of full-fat milk or equivalent high-fat food portion).

Any missed or incomplete doses of study medication should be recorded in the Subject Diary and reported immediately to the site Investigator.

## 5.4 Rationale for Dose Selection

Dose levels of the investigational medication were chosen for this study to ensure the safety of subjects participating in the study, and to allow demonstration of efficacy and PD effects. The prednisone (active control) dose was selected based on current standard of care practice in boys with DMD.

All doses of study medication will be administered in the morning with breakfast, including at least 8 g of fat (approximately 8 ounces [240 mL] of full-fat milk or equivalent high-fat food portion).

Based on the comparison between the PK parameters in DMD boys receiving 0.25 mg/kg or 0.75 mg/kg vamorolone with a glass of full-fat milk or equivalent fat-containing food and the PK parameters in healthy adult males receiving similar doses of vamorolone fasted, it appears that the 2.5-fold increase in exposure observed between the fasted and fed conditions in the healthy adult males (**Section 1.3.1**) is not reproduced in the DMD boys (see **Section 1.3.2**). The rationale for the lowest vamorolone dose of 2.0 mg/kg/day, administered with a glass of full-fat milk or equivalent fat-containing food is as follows: A starting dose of 2.0 mg/kg/day with a glass of milk is approximately 10% of the highest safe dose tested in adults (20.0 mg/kg/day fasted).

The highest vamorolone dose to be administered, 6.0 mg/kg/day, will similarly be administered with a glass of full-fat milk or equivalent fat-containing food. As 20.0 mg/kg/day fasted in adult volunteers was shown to be safe in the Phase I adult volunteer study, the proposed highest dose in 4 to 7 year-old children is approximately 30% of the highest safe adult dose. Based on the Phase I PD biomarker safety data presented in **Section 1.3**, safety signals reflective of insulin resistance are not anticipated at either of the planned dose levels. Also based on the Phase I data, vamorolone showed little evidence of either acute (24-hour data) or chronic (Day 15 data) suppression of the adrenal axis at doses of either 1.0 mg/kg/day or 3.0 mg/kg/day. The data suggest that vamorolone induces variable, mild, acute and chronic suppression of the adrenal axis at 9.0 mg/kg/day, and stronger evidence of both acute and chronic adrenal axis suppression at 20.0 mg/kg/day.

Vamorolone at doses of 0.25 mg/kg/day, 0.75 mg/kg/day, 2.0 mg/kg/day and 6.0 mg/kg/day has been demonstrated to be safe and well-tolerated in a 2-week Phase IIa study (VBP15-002) in 4 to <7 years DMD boys. The safety of these four doses continued to be studied in the 24-week Phase II extension study (VBP15-003) in 4 to 7 year-old DMD boys, and continues to be studied in the ongoing VBP15-LTE long-term extension study. There were a total of 4 SAEs in the Phase II VBP15-003 study and three SAEs to date in the VBP15-LTE extension study: two SAEs of pneumonia in two different subjects (both subjects receiving vamorolone 0.75 mg/kg/day), one SAE of bilateral testicular torsion and one SAE of hypoxia in the same subject receiving 6.0 mg/kg/day, one SAE of influenza associated dehydration in a subject receiving 6.0 mg/kg/day, and two SAEs of acute myoglobinuria in the same subject receiving 6.0 mg/kg/day. Each of these SAEs was considered unrelated to study drug, and none of them resulted in discontinuation from the study. One subject receiving vamorolone 6.0 mg/kg in the Phase II extension study (VBP15-003) who had an incidental early morning cortisol drawn following an AE of presyncope had evidence of adrenal suppression. In the VBP15-003 study, after 24 weeks of treatment, 0 of 8 tested participants (0.25 mg/kg/day), 1 of 12 (8.3%) tested participants (0.75 mg/kg/day), 5 of 12 (41.7%) tested participants (2.0 mg/kg/day), and 8 of 9 (88.9%) tested participants (6.0 mg/kg/day) had a depressed morning cortisol (<3.6 µg/dL [100 nM]) consistent with chronic adrenal suppression.[29] Thus, based on the available data in the Phase I and Phase II studies regarding the safety signal of suppression of the adrenal axis, the possibility of adrenal suppression is present in subjects at the 2.0 and 6.0 mg/kg/day dose levels.

The dose of prednisone has been selected according to the Care Recommendation for DMD (daily prednisone 0.75 mg/kg/day). The weight-dose bands (**Table** 10) have been selected to ensure that subjects will not be overdosed in view of the potential side effects.

## 5.5 Treatment Compliance

Subject compliance with the dosing schedule will be assessed by site maintenance of accurate study drug dispensing and return records, and accurate recording of incomplete or missed doses by completion of a diary by the subject's parent or guardian. The

Investigator is responsible for ensuring that dosing is administered in compliance with the protocol. The Investigator or designee will instruct the subject's parent or guardian with regard to proper dosing of study medication and completion of subject diaries, and will reinforce the importance of taking all study medication per protocol instructions. Doses of study drug on the days of the Day 1, Week 2, Week 12, and Week 24 Visits (Treatment Period #1), Week 28 (Transition Period), and Week 30, Week 40, and Week 48 (Treatment Period #2) will be administered at the participating study site by a trained investigational staff member. All incomplete or missed doses are to be documented in the source document and on the appropriate eCRF page. The volume of unused study medication remaining in each bottle returned, as well as the number of unused tablets, will be documented in the source documents and on the appropriate eCRF page.

## 5.6 Study Drug Dose Interruption or Discontinuation

In the event any clinical observation suggests an intolerability of an individual subject to the study medication, in the opinion of the Investigator, the case should be discussed with the Study Chair and the Independent Medical Monitor within 24 hours and study drug discontinuation should be considered. In view of the potential effect of the study drugs on adrenal glands, the study drugs cannot be discontinued suddenly. In case study drug needs to be discontinued, for whatever reason, the dose tapering process described for the end of the treatment period should be followed. If a subject discontinues study drug due to intolerability, the subject will be withdrawn from the study. Study drug discontinuation due to intolerability will not usually require unblinding (see **Section 3.4**). The subject should return to the study site for completion of Week 24 assessments and the Week 24 Follow-up Visit ACTH Stimulation Test at the time of early withdrawal (if withdrawal is prior to the Week 24 Visit), or Week 48 assessments and the Week 48 Follow-up Visit ACTH Stimulation Test (if withdrawal is after Week 24 and prior to the Week 48 Visit), prior to participation in the Dose-tapering Period. Any AE still ongoing at the time of study drug discontinuation will be monitored until it has returned to baseline status, stabilized, or the Investigator, Study Chair, Medical Monitor and Sponsor agree that follow-up is no longer needed.

## 5.7 Prior and Concomitant Medications and Therapies

### *5.7.1 Prior Therapy*

All medications (prescription and over-the-counter [OTC]) taken within 3 months prior to the Screening Visit through Baseline Day -1 (until just prior to administration of the first dose of study medication) will be captured as prior medications (Medication History) in the source document and the eCRF, including the name of the medication (or device or procedure), the dosage and regimen, the indication, and the treatment start and stop dates. All past (lifetime) steroid use will be recorded.

### *5.7.2 Concomitant Therapies*

Any medications that are taken after administration of the first dose of study medication will be recorded as concomitant medications on the appropriate eCRF page. Subject diaries will be provided to subjects to record any concomitant medication changes during the study (see **Section 7.4.6**).

All medications (prescription and OTC) taken during the study must be recorded in the source documents and in the eCRF, including the name of the medication, dosage and regimen, reason for therapy, and treatment start and stop dates. Furthermore, each change in concomitant medication (e.g., new treatment, discontinuation of treatment, or change in dosage/regimen) during the study must be documented in the same manner. Details of any non-pharmacological therapies (e.g., devices, procedures), including name, reason for therapy (i.e., DMD or non-DMD), and dates of therapy will also be recorded. Site personnel will review the information with the subject and/or his parent or guardian, if applicable, for completeness and accuracy at each study visit.

### *5.7.3 Prohibited Therapies*

Subjects must discontinue use of the following medications prior to participation in the study, as indicated, and refrain from using these medications throughout the duration of the study:

- Mineralocorticoid receptor agents, such as spironolactone, eplerenone, canrenone (canrenoate potassium), prorenone (prorenoate potassium), mexrenone

(mexrenoate potassium): use must be discontinued at least 4 weeks prior to the first dose of study medication;

- Oral glucocorticoids or other immunosuppressive agents. Subjects who have received prior treatment with immunosuppressive agents are ineligible for study entry. [Notes: Inhaled and/or topical glucocorticoids are permitted but must be administered at stable dose beginning at least 4 weeks prior to first dose of study medication, and are anticipated to be used at the stable dose regimen for the duration of the study; past transient use of oral or inhaled glucocorticoids or other oral immunosuppressive agents for no longer than 1 month cumulative, with last use at least 3 months (or last use at least one month prior for inhaled glucocorticoids) prior to first dose of study medication, will be considered for eligibility on a case-by-case basis.]
- Idebenone: use must be discontinued at least 4 weeks prior to the first dose of study medication
- Live attenuated vaccines: use must be avoided within 14 days prior to first dose of study medication and for the duration of participation in the study
- Any investigational medications other than vamorolone: use must be discontinued at least 3 months prior to the first dose of study medication
- Other medications indicated for the treatment of DMD, including Exondys 51 and Translarna: use must be discontinued at least 3 months prior to the first dose of study medication.
- Any approved medications or herbal remedies which can impact strength and function (including, but not limited to, Co-enzyme Q10, creatine): use must be discontinued at least 4 weeks prior to the first dose of study medication.

In addition, vamorolone should be used with caution with any drug metabolized by cytochrome P450 3A4 (CYP3A4).

The Investigator should contact the Study Chair and Medical Monitor concerning individual medications or therapies not listed that may be of concern.

### 5.7.4 *Permitted Therapies*

Every effort should be made NOT to start any prescription or OTC medications during the study. Concomitant medications should be maintained on the same dose and regimen throughout the study whenever possible. However, all other medications other than those specifically prohibited above may be taken during the study, if clinically indicated, provided they are recorded in the source documents and in the eCRF.

### 5.7.5 *Hydrocortisone*

All subjects will be given a single dose of hydrocortisone (5 mg or 10 mg) 24 hours after the final dose of Treatment Period #1 study medication at the Week 24 Visit, and 24 hours after the final dose of Treatment Period #2 study medication at the Week 48 Visit. The hydrocortisone dose will be approximately 8 mg/m$^2$, rounded up to either 5 mg or 10 mg; subjects will be provided with either a single 5 mg or 10 mg hydrocortisone tablet which will be dispensed by the site staff at the Week 24 and Week 48 Visits.

In addition, all subjects should be covered with "stress dosing" of hydrocortisone (or prednisone) during times of illness, injury, or surgery (see **Section 7.2.7**).

### 5.7.6 *Vitamin D*

Serum Vitamin D levels will be measured at Screening and at the Weeks 12, 24, 40, and 48 Visits. Vitamin D insufficiency and deficiency (serum 25[OH] D concentration less than 20 ng/mL or less than 50 nmol/L) will be treated with high doses of Vitamin D supplement according to local site guidelines. Vitamin D supplements will be recorded in the source document and in the eCRF.

## 5.8 Study Medication Management

### 5.8.1 *Packaging and Labeling of Study Medication*

When all entry criteria are met, and at least 10 days prior to the Baseline Day -1 Visit, subjects will be randomized to treatment via the IXRS system. Subject-specific suspension and tablet study drug supplies sufficient for the first six weeks of Treatment Period #1 will be packaged, labeled (with MED ID number and other protocol-specific

information) and couriered to the pharmacy at the recruiting site prior to the Baseline Visit for the subject.

Investigational medication suspension and matching placebo suspension will be packaged in sterile 120 mL (4 oz) amber bottles with a 100 mL fill volume with child-resistant cap with a 24 mm bottle press-in adapter. Bottles are filled with 110 mL of suspension in order to guarantee a delivery of 100 mL.

Bottles of investigational medication suspension will contain either 1.33 grams of vamorolone/100 mL (1.33% orange-flavored suspension) or 4.0 grams of vamorolone/100 mL (4.0% orange-flavored suspension). The matching placebo suspension will be identical in appearance and taste to the vamorolone suspensions. The volume per dose to be administered to each subject depends on the subject's weight (in kg) recorded at the visit prior to each study drug dispensing visit. Each subject will receive a volume of 0.15 mL/kg of a vamorolone suspension or matching placebo suspension (**Table 7**). Instructions for the calculation of each dose of liquid formulation are given in the Pharmacy Manual.

Prednisone 5 mg tablets and matching placebo will be dispensed in blister packs, each containing 15 tablets. The number of tablets to be administered per dose to each subject depends on the subject body weight calculated at the visit prior to each study drug dispensing visit (**Table 10**).

Drug supplies will be labeled with the Sponsor name, protocol number, lot number, expiration or retest date, and other appropriate study information. Carton, bottle, and blister pack labels will be written in accordance with all applicable laws, guidance and directives of the jurisdiction where the study is being conducted.

Trained site staff will write the dose in mL (suspension) and number of tablets (tablets) on the bottle and blister pack labels, respectively, prior to bottle and blister pack dispensing to subjects.

At the Screening Visit, a test packet containing placebo tablets will be provided by the site investigator to confirm the subject's ability to swallow tablets. The test of swallowing ability will take place at the Screening Visit. At the beginning of the trial, the

clinical trials supply company will provide each site with sufficient test packets for their anticipated recruitment numbers. These test packets will be stored in a locked cupboard, at ambient room temperature.

Study medication will be dispensed to the subject's parent or legal guardian for Treatment Period #1 dosing at the Day 1 Visit and at the Week 6, Week 12 and Week 18 Visits. At the Week 24 Follow-up Visit, a 4-week supply of study medication for the Week 25 to Week 28 Transition Period will be dispensed. Study medication will be dispensed to the subject's parent or legal guardian for Treatment Period #2 dosing at the Week 28, Week 34, and Week 40 Visits. At the Week 48 Follow-up Visit, study medication for the Dose-tapering Period will be dispensed. Dispensed bottles and unused tablets of study medication will be returned at each subsequent study visit, prior to dispensing bottle(s)/blister pack(s) for the next dispensing interval (see **Section 5.3**). Each study medication bottle and blister pack may be used for a single subject only.

Clinical supplies dispensed by the study site staff and ready for administration to subjects will be labeled with the dispense date, protocol number, MED ID number, and volume (suspension) or number of tablets to be administered per dose.

### *5.8.2 Storage of Study Medication*

All Clinical Trial Materials (CTM) for use in the trial must be stored in a locked container/cabinet free from environmental extremes, under the responsibility of the institutional pharmacist or Principal Investigator. Study medication suspensions should be stored at refrigerated temperature (2ºC – 8ºC; 36ºF – 46ºF). Excursions to ambient temperature are allowed (see Pharmacy Manual for details). Study medication tablets will be stored in a locked cupboard, at ambient room temperature.

Access to study medication stored at the study site must be limited to authorized clinic personnel.

### 5.8.3 *Study Medication Shipping and Handling*

Clinical trial material will be shipped to the study sites only after receipt of required documents in accordance with applicable regulatory requirements and Sponsor procedures.

When all entry criteria are met, at least 10 days prior to the Baseline Visit, subjects will be randomized to treatment via the IXRS system. Subject-specific suspension and tablet drug supplies will be packaged, labeled (with MED ID number and other protocol-specific information) and couriered to the pharmacy at the study site prior to the Baseline Visit for the subject. At the Day 1 Visit, the trained site staff will record the volume in mL per suspension dose (bottles) and number of tablets per dose (blister packs) on the bottle and blister pack labels, respectively. Labeled study drug supplies sufficient to last until the Week 6 Visit will be dispensed to each subject. The first dose of study medication will be administered in clinic on Study Day 1. The initial drug supply will be sufficient to allow for the Week 6 Visit to occur on the latest date permissible within the protocol-specified visit window (6 weeks ± 3 days). No additional study drug supplies will be dispensed at the Week 2 Visit.

Prior to the subsequent study drug dispensing visits (i.e., Week 6, Week 12, Week 18, Week 24 Follow-up, Week 28, Week 34, Week 40, Week 48 Follow-up), additional study drug supplies will be ordered using the IXRS system. Study drug dispensed at the Week 28, Week 34, Week 40, and Week 48 Follow-up Visits for dosing during Treatment Period #2 and the Dose-tapering Period will consist of liquid suspension only (no tablets). The new subject-specific drug supply will be packaged, labeled (as described above) and couriered to the site. Study drug will be ordered at least 10 days prior to the next scheduled study drug dispensing visit to allow time for the new supplies to be shipped to the site in time for the next scheduled dispensing visit.

It is essential to this study that all CTM be accounted for during the study period. All unused (i.e., undispensed; dispensed and returned) study medication will be retained at the study site for reconciliation by the Sponsor's study monitors (or designees) during routine monitoring visits. Final disposition of all unused CTM will be coordinated by the

Sponsor's study monitors (or designees) throughout and at the end of the study (see Section 5.8.4).

Clinical trial material must be dispensed and administered according to the procedures described in this protocol. Only subjects randomized in the study may receive study medication, in accordance with all applicable regulatory requirements. Only authorized study personnel may supply CTM. Authorized study personnel refers to the Investigator (or designee) and hospital pharmacists, in accordance with all applicable regulatory requirements and the Site Signature Log/Delegation of Authority. Only authorized study personnel or the subject's parent or legal guardian may administer CTM.

### *5.8.4 Study Medication Accountability*

The Investigator is responsible for the control of drugs under investigation. Adequate records of the receipt (e.g., Drug Receipt Record) and disposition (e.g., Drug Dispensing Log) of the study drug must be maintained. The Drug Dispensing Log must be kept current and should contain the following information:

- The Subject ID number of the subject to whom the study drug was dispensed
- The MED ID number of the dispensed kit
- The date(s) and quantity of the study drug dispensed to the subject
- The date(s) and quantity of the study drug returned by the subject.

All records and drug supplies must be available for inspection by the Study Monitor at every monitoring visit. Unused medication will be returned to ReveraGen Inc. or its designee or destroyed on site at the end of the study or at a specific time in agreement with the Sponsor, as coordinated between the site and ReveraGen or its designee. The completed Drug Dispensing Log and Drug Return Record(s) will be returned to ReveraGen Inc or its designee. The Investigator's copy of the Drug Return Record(s) must accurately document the return of all study drug supplies to ReveraGen Inc. or its designee.

## 5.9 Procedures for Assigning Subject Study Numbers

All data for all subjects whose parent(s) or guardian(s) sign the Informed Consent Form (ICF) for the study will be identified using the unique subject identification number. Subjects are considered to be enrolled in the study when the parent(s) or guardian(s) signs the study-specific ICF at Screening. The Site Investigator will keep a record relating the names of the subjects to their ID numbers (subject identification log) to permit efficient verification of data subject files, when required. A subject enrollment log will include the dates of subject enrollment and completion/termination.

# 6 STUDY SCHEDULE

## 6.1 Time and Events Schedule

The study procedures to be conducted for each subject are divided into the following study periods:

- **Pretreatment Screening Period:** The up to 32-day interval, from subject's parent or guardian signing of the Informed Consent/HIPAA authorization until completion of all designated screening procedures, 24 hours prior to the first dose of study medication. All screening procedures must be completed by Day -11. Subjects meeting all eligibility criteria will be randomized by Day -11, at least 10 days prior to the Baseline Day -1 Visit.
- **Pretreatment Baseline Period:** The 24-hour period immediately prior to administration of the first dose of study medication (Baseline Day -1).
- **Treatment Period #1:** The 24-week interval starting with administration of the first dose of study medication on Study Day 1 and continuing through the time of the Week 24 Follow-up Visit. Treatment Period #1 includes administration of the final dose of Treatment Period #1 study medication at the Week 24 Visit, and ACTH Stimulation testing at the Week 24 Follow-up Visit, 48 ± 3 hours after the final dose of Treatment Period #1 study medication.

- **Transition Period:** The 4-week interval following the end of the 24-week Treatment Period #1 during which subjects will continue on their suspension study medication at the same dose they received during the Treatment Period #1 and have their tablet study medication dose tapered to zero (0) tablets/day (see **Section 6.3.5**). Once subjects have completed all study assessments for the Transition Period, they will enter Treatment Period #2.
- **Treatment Period #2:** The 20-week interval starting with administration of the first dose of Treatment Period #2 study medication on the day after the Week 28 Visit (Week 28 + 1 day) and continuing through the time of the Week 48 Follow-up Visit. Treatment Period #2 includes administration of the final dose of Treatment Period #2 study medication at the Week 48 Visit, and ACTH Stimulation testing at the Week 48 Follow-up Visit, 48 ± 3 hours after the final dose of Treatment Period #2 study medication. Subjects who will not participate in the Dose-tapering Period will be discharged from the study following completion of the Week 48 assessments and the ACTH Stimulation Test at the Week 48 Follow-up Visit.
- **Dose-tapering Period:** The 4-week interval following the end of the 20-week Treatment Period #2 during which subjects will have their suspension study medication dose tapered to 0 mg/kg/day (see **Section 6.3.7**). Once subjects have completed the Dose-tapering Period, they will be discharged from the study following completion of all final Dose-tapering Period assessments.

The procedures to be completed at each visit during each study period are presented in the Schedule of Study Activities in **Table 11** and in the sections that follow. (Note: In **Table 11**, each visit with the acceptable time window around the planned visit date, where applicable, is provided.) Detailed descriptions of the assessments and the definitions of study endpoints are provided in **Section 7** and **Section 2**, respectively. Any deviation from study procedures should be noted in the source documents and in the Clinical Trial Management Software (CTMS), and significant deviations should be reported immediately to the Sponsor.

Overall, up to approximately 57 weeks are allocated for each subject to complete the study, including a 32-day Pretreatment Screening Period, a one-day Pretreatment Baseline Period, a 24-week Treatment Period #1, a 4-week Transition Period, a 20-week Treatment Period #2, plus a 4-week Dose-Tapering Period, as applicable. Upon the completion of the study, subjects may have the option to enroll in an additional vamorolone study or general access program.

Subjects electing to enroll directly into an additional vamorolone study or general access program to continue vamorolone therapy will be discharged from the VBP15-004 study following completion of all final Week 48 assessments and ACTH Stimulation testing at the Week 48 Follow-up Visit, and will be enrolled in the additional vamorolone study or general access program (separate written protocol and ICF).

Subjects completing the VBP15-004 study and enrolling directly into the additional vamorolone study or general access program to continue vamorolone treatment do not need to dose taper in VBP15-004.

## Table 11 Schedule of Study Activities

| | **Pretreatment Period** | | **Treatment Period #1** | | | | | | | **Transition Period** | | **Treatment Period #2** | | | | | | **Dose-tapering Period** | |
|---|---|---|---|---|---|---|---|---|---|---|---|---|---|---|---|---|---|---|---|
| | **SCR** | **BL** | | | | | | | | | | | | | | | | | |
| | **Day** | | | **Week** | | | | | | | | | | | | | | | |
| **Study Day or Week/Visit** | **-33 to -2**[a] | **-1**[b] | **1**[c] | **2 (±1d)**[d] | **6 (±3d)**[d] | **12 (±1w)**[d] | **18 (±1w)**[d] | **24**[e] **(±1w)**[d] | **24 (F/U)**[e] | **26** | **28**[f] **(±1d)**[d] | **28+1d** | **30 (±1d)**[d] | **34 (±3d)**[d] | **40 (±1w)**[d] | **48**[g] **(±1w)**[d] | **48 F/U**[g] | **50** | **52**[h] **(±1d)**[d] |
| Informed consent | X | | | | | | | | | | | | | | | | | | |
| Enrollment[i] | X | | | | | | | | | | | | | | | | | | |
| Inclusion/exclusion criteria | X | X[j] | | | | | | | | | | | | | | | | | |
| Randomization[k] | X | | | | | | | | | | | | | | | | | | |
| Demographics | X | | | | | | | | | | | | | | | | | | |
| Medical history | X | | | | | | | | | | | | | | | | | | |
| Medication history | X | X | | | | | | | | | | | | | | | | | |
| Physical examination | X | X | | | X | X | X | X | | | X | | | X | X | X | | | X |
| Cushingoid features | | X | | | X | X | X | X | | | X | | | X | X | X | | | X |
| Height | X | | | | | X | | X | | | | | | X | | X | | | |
| Weight | X | X | | X | X | X | X[l] | X | | | X | | X | X | X[l] | X | | | X |
| Vital signs[m] | X | X | X[n] | X | X | X | X | X | | | X | | X | X | X | X | | | X |
| Blood for clinical labs[o] | X | | X[p] | X[p] | X | X[p] | X | X[p] | | | X[p] | | X[p] | X | X[p] | X[p] | | | X[p] |
| Blood for HbA1c[o] | X | | | | | | | X | | | | | | | | X | | | |
| Blood for vitamin D[o] | X | | | | | X | | X | | | | | | | X | X | | | |
| Blood for chicken pox IgG | X | | | | | | | | | | | | | | | | | | |
| Urinalysis[q] | X | | X[p] | X[p] | X | X[p] | X | X[p] | | | X[p] | | X[p] | X | X[p] | X[p] | | | X[p] |
| Blood for serum PD biomarker panel[r,s] | | | X | | | X | | X | | | X | | | | X | X | | | X |
| Fasting blood for insulin, glucose[s] | | | X | | | X | | X | | | X | | | | X | X | | | X |
| Blood for DNA Testing | | | | | | | | X | | | | | | | | | | | |
| ACTH Stimulation Test | X | | | | | | | | X[t] | | | | | | | | X[t] | | |
| Blood for Plasma PK | | | | | | | | | | | | | X[u] | | | | | | |
| 12-lead ECG[v] | X | | | | | X | | X | | | | | | | X | X | | | |
| 2D-echocardiogram | X | | | | | | | X | | | | | | | | X | | | |
| Eye examination | X | | | | | | | X | | | | | | | | X | | | |

| | **Pretreatment Period** | | **Treatment Period #1** | | | | | | | **Transition Period** | | **Treatment Period #2** | | | | | | **Dose-tapering Period** | |
|---|---|---|---|---|---|---|---|---|---|---|---|---|---|---|---|---|---|---|---|
| | **SCR** | **BL** | | | | | | | | | | | | | | | | | |
| | **Day** | | | **Week** | | | | | | | | | | | | | | | |
| **Study Day or Week/Visit** | **-33 to -2[a]** | **-1[b]** | **1[c]** | **2 (±1d)[d]** | **6 (±3d)[d]** | **12 (±1w)[d]** | **18 (±1w)[d]** | **24[e] (±1w)[d]** | **24 (F/U)[e]** | **26** | **28[f] (±1d)[d]** | **28+1d** | **30 (±1d)[d]** | **34 (±3d)[d]** | **40 (±1w)[d]** | **48[g] (±1w)[d]** | **48 F/U[g]** | **50** | **52[h] (±1d)[d]** |
| DXA scan | X | | | | | | | X | | | | | | | | X | | | |
| Spine X-ray | X | | | | | | | X | | | | | | | | | | | |
| Fracture Questionnaire | X | | | | | | | X | | | | | | | | X | | | |
| Dispense study medication | | | X | | X | X | X | | X | | X | | | X | X | | X | | |
| Return study medication/ compliance monitoring | | | | X[w] | X | X | X | X | | | X | | X[w] | X | X | X | | | X |
| Study medication dosing[x] | | | X → | → | → | → | → | X | | | | X → | → | → | → | X | | | |
| Study medication dose tapering | | | | | | | | | X[y] → | → | X | | | | | | X → | → | X |
| Telephone call to subject[z] | | | | | | | | | | X | | | | | | | | X | |
| Time to Stand Test (TTSTAND) | X | X | | | X | X | | X | | | | | | X | X | X | | | |
| Time to Climb Test (TTCLIMB) | X | X | | | | X | | X | | | | | | | X | X | | | |
| Time to Run/Walk Test (TTRW) | X | X | | | | X | | X | | | | | | | X | X | | | |
| NSAA[aa] | X | X | | | | X | | X | | | | | | | X | X | | | |
| Myometry (elbow flexors, knee extensors) | X | X | | | | X | | X | | | | | | | X | X | | | |
| Six-minute Walk Test (6MWT) | X | X | | | | X | | X | | | | | | | X | X | | | |
| Range of Motion (ROM) - ankles | X | X | | | | X | | X | | | | | | | X | X | | | |
| Pediatric Outcomes Data Collection Instrument (PODCI) | X | | | | | | | X | | | | | | | | X | | | |
| Treatment Satisfaction Questionnaire (TSQM) | | | | | | | | X | | | | | | | | X | | | |
| PARS III | X | | | | | X | | X | | | | | | | | X | | | |

| | **Pretreatment Period** | | **Treatment Period #1** | | | | | | | **Transition Period** | **Treatment Period #2** | | | | | | | **Dose-tapering Period** | |
|---|---|---|---|---|---|---|---|---|---|---|---|---|---|---|---|---|---|---|---|
| | **SCR** | **BL** | | | | | | | | | | | | | | | | | |
| | **Day** | | | **Week** | | | | | | | | | | | | | | | |
| **Study Day or Week/Visit** | **-33 to -2[a]** | **-1[b]** | **1[c]** | **2 (±1d)[d]** | **6 (±3d)[d]** | **12 (±1w)[d]** | **18 (±1w)[d]** | **24[e] (±1w)[d]** | **24 (F/U)[e]** | **26** | **28[f] (±1d)[d]** | **28+1d** | **30 (±1d)[d]** | **34 (±3d)[d]** | **40 (±1w)[d]** | **48[g] (±1w)[d]** | **48 F/U[g]** | **50** | **52[h] (±1d)[d]** |
| Ease of Study Medication Administration Assessment[bb] | | | | X | | X | | X | | | | | X | | X | X | | | |
| Blindedness Assessment | | | | | | | | X | | | | | | | | | | | |
| Dispense subject diaries[cc] | | | X | X | X | X | X | X | | | X | | X | X | X | X | | | |
| Return subject diaries | | | | X | X | X | X | X | X[dd] | | X | | X | X | X | X | X[ee] | | X |
| AE/SAE recording[ff] | X | → | → | → | → | → | → | → | → | → | → | → | → | → | → | → | → | → | → X[gg] |
| Concomitant medications | | | X | → | → | → | → | → | → | → | → | → | → | → | → | → | → | → | → X |
| Discharge from study | | | | | | | | | | | | | | | | | X[hh] | | X[ii] |

BL = Baseline; d = day(s); F/U = Follow-up; SCR = Screening; w = week.

a. The Pretreatment Screening Period spans Day -33 through Day -2, but all screening procedures must be completed by Day -11. Subjects meeting all eligibility criteria will be randomized by Day -11, at least 10 days prior to the Baseline Day -1 Visit.

b. Baseline Day -1, within 24 hours prior to administration of the first dose of study drug.

c. Treatment Day 1 begins at the time of administration of the first dose of study medication in the clinic.

d. Time windows around the Week 2, Week 6, Week 12, Week 18, and Week 24 Visits are allowances from date of Day 1 Visit. Time window around the Week 28 Visit is allowance from date of Week 24 F/U Visit. Time windows around the Week 30, Week 34, Week 40, and Week 48 Visits are allowances from date of Week 28+1d Visit. Time window around the Week 52 Visit is allowance from date of Week 48 F/U Visit.

e. Subjects who prematurely discontinue from the study prior to Week 24 should complete the Week 24 assessments and the Week 24 Follow-up Visit ACTH Stimulation Test at the time of early withdrawal and undergo Early Discontinuation Dose-tapering, where possible (see Section 6.4.1 and Section 7.2.7). The Week 24 Follow-up Visit must occur to allow the ACTH Stimulation Test to be performed 48 ± 3 hours after the final dose of Treatment Period #1 study medication.

f. Subjects who prematurely discontinue from the study after Week 24 but prior to Week 28 should complete the Week 28 assessments, and undergo Early Discontinuation Dose-tapering, where possible (see Section 6.4.2).

g. Subjects who prematurely discontinue from the study after Week 28 but prior to Week 48 should complete the Week 48 assessments and the Week 48 Follow-up Visit ACTH Stimulation Test at the time of early withdrawal and undergo Dose-tapering, where possible (see Section 6.4.3 and Section 7.2.7). The Week 48 Follow-up Visit must occur to allow the ACTH Stimulation Test to be performed 48 ± 3 hours after the final dose of Treatment Period #2 study medication.

h. Subjects will have one study site visit during the Dose-tapering Period, at one week after the dose of liquid formulation has been discontinued (Week 52) (see Section 6.3.7).

i. Subjects are considered to be enrolled in the study at the time written informed consent is obtained.

j. Study eligibility should be rechecked and confirmed at Baseline Day -1 Visit.

k. Randomization occurs by Interactive Voice/Web Response System (IXRS) after subjects are confirmed to have met all study entry criteria, at least 10 days prior to the Baseline Day -1 Visit.

l. Weight recorded at the Week 18 Visit and the Week 40 Visit will be used to calculate doses for study drug dispensed at the Week 24 Follow-up and Week 48 Follow-up Visits, respectively.

m. Sitting blood pressure, body temperature, respiratory rate, and heart rate.

n. Vital signs recorded prior to administration of the first dose of study drug at the Day 1 Visit.

o. Blood for hematology, chemistry, and lipids, including HbA1c and Vitamin D where applicable.

p. Blood samples (collected after subjects have fasted for ≥ 6 hours) and urine collected at scheduled visit, and prior to dose of study drug where applicable.

q. Urinalysis by dipstick and microscopic analysis.

r. Blood collected for PD biomarkers includes secondary safety outcomes (morning cortisol, osteocalcin, CTX1, P1NP), and exploratory safety and efficacy PD biomarkers.

s. Blood samples for PD biomarkers and fasting glucose and insulin determination will be collected after subjects have fasted for ≥ 6 hours, prior to the daily dose of study medication where applicable.

t. Subjects will return to the study site for the Week 24 Follow-up Visit for an ACTH Stimulation Test 48 hours ± 3 hours after administration of the final dose of Treatment Period #1 study medication, and for the Week 48 Follow-up Visit for an ACTH Stimulation Test 48 hours ± 3 hours after administration of the final dose of Treatment Period #2 study medication (see **Section 7.2.7**).

u. Blood sample for population PK analysis will be collected 2 hours after administration of the daily dose of study medication.

v. 12-lead ECG recorded after subject has rested quietly in a supine position for at least 5 minutes.

w. Study medication brought by subjects to the Week 2 Visit and Week 30 Visit for dosing and compliance assessment will be redispensed to subjects at the end of the visit.

x. The dose of study medication on the days of the Day 1, Week 2, Week 12, Week 24, Week 28, Week 30, Week 40, and Week 48 Visits will be administered after 1) fasting blood draws; and 2) breakfast provided by the study site. All other doses will be taken at home. See **Section 6.3.3** for other Day 1 pre-dose safety assessments.

y. Doses of tablet study drug will be tapered and suspension study drug will be continued, during Weeks 24-28.

z. Site study staff will contact the parent(s)/guardian(s) by telephone at Weeks 26 and 50 to ensure that the study drug tapering is proceeding according to protocol, to assess potential signs or symptoms indicative of adrenal suppression, and to address any questions the parent(s)/guardian(s) may have.

aa. North Star Ambulatory Assessment; includes the Time to Stand Test (TTSTAND).

bb. Ease of Study Medication Administration assessed at the Weeks 2, 12, and 24, 30, 40, and 48 Visits.

cc. Subject diaries used to record any changes in concomitant medications taken, any AEs experienced during the study, and any incomplete or missed doses of study medication.

dd. Subject diaries dispensed at the Week 24 Visit will be returned and redispensed at the Week 24 F/U Visit; final return will occur at the Week 28 Visit.

ee. Subject diaries dispensed at the Week 48 Visit will be returned at the Week 48 F/U Visit, and will be redispensed to subjects participating in the Dose-tapering Period for final return at the Week 52 Visit.

ff. All AEs and SAEs must be recorded in the source documents and eCRF from the date of the subject's written informed consent until the final Week 52 Visit or the subject's participation in the study is completed (SAEs through 30 days after final study drug dose). Ongoing AEs will be followed to resolution, stabilization, or until such time the Investigator agrees follow-up is not necessary.

gg. For subjects who do not continue to receive vamorolone through an additional vamorolone study or general access program, site staff will make a phone call to the home 31-35 days after the final dose of study medication in VBP15-004 Dose-tapering Period to confirm the final SAE status of the subject.

hh. Subjects who elect to continue vamorolone therapy by enrolling directly into an additional vamorolone study or general access program may be discharged from the study following completion of all final Week 48 assessments, including the Week 48 Follow-up Visit ACTH Stimulation Test.

ii. Subjects who participate in the Dose-tapering Period may be discharged from the study following completion of all final Dose-Tapering Visit assessments (Week 52) (see **Section 6.3.7**).

## 6.2 Informed Consent and Assent Procedures

Subjects are considered to be enrolled in the study at the time written informed consent is obtained.

The parent(s) or guardian(s) of all subjects are to give informed consent in accordance within the Declaration of Helsinki, US 21 Code of Federal Regulations (CFR) Part 50, International Conference on Harmonisation [ICH] guidelines on GCP and all applicable laws, guidances, and directives of the jurisdiction where the study is being conducted.

The parent(s) or guardian(s) of subjects who choose to enroll in this study will give written informed consent at the Screening Visit, Day -33 to Day -11. The Investigator (or designated staff) will obtain the written informed consent from the subject's parent(s) or guardian(s) prior to any study-specific procedures. Each subject's parent(s) or guardian(s) will receive an explanation of the nature and purposes of the study from the Investigator or designee. Time will be given to the parent(s)/guardian(s) to ask questions and make their decision on whether they would like for their child to participate. The Investigator or designee will ensure the study is appropriate for the subject. Reasons for exclusion will be documented for subjects found ineligible during the Pretreatment Period. The subject's parent(s) or guardian(s) will be asked if s/he understands that the study is for research purposes only and that it may not provide any therapeutic benefit to the subject. Each subject's parent(s) or guardian(s) will be asked if s/he understands that the subject is free to withdraw from the study at any time without prejudice. Each subject's parent(s) or guardian(s) will be required to sign a study ICF (and HIPAA authorization, if applicable) before any procedures are performed for the study; both parents or guardians will sign the ICF in jurisdictions where this is required.

If applicable, the assent of the child himself will also be obtained, if possible in writing per individual where a child is intellectually capable of assenting (and in accordance with local regulations), and with the permission of the parent(s)/guardian(s).

The Investigator or designee will obtain written informed consent from each subject's parent(s) or guardian(s) prior to subject's participation in the study using ICFs approved by the appropriate IRB/IEC at each site. Consent must be obtained in accordance with

the principles outlined in the current version of the Declaration of Helsinki. Informed Consent Forms must be dated and signed by the Investigator or designee and the subject's legal representative(s) and the original signed consent form must be kept by the Investigator in the study subject's file. "Legal representative" means an individual whom a judicial or other body authorized under applicable law to consent on behalf of a prospective study subject to the subject's participation in the procedure(s) involved in the research. The Study Monitor will ensure that the ICF has been signed by the subject's legal representative(s). The study subject's legal representative(s) will receive a copy of the signed consent form.

### *6.2.1 HIPAA and Protected Health Information*

In the applicable countries, during the informed consent procedure, the Investigator or designee will review the elements of the HIPAA and Protected Health Information (PHI) with each subject's parent(s) or guardian(s), and each subject's parent or guardian will confirm that s/he understands HIPAA authorization and PHI. The Investigator (or designated staff) will obtain HIPAA authorization from the subject's parent(s) or guardian(s) on the appropriate IRB/IEC-approved form at each site, prior to any study-related procedures.

## 6.3 Visit Schedule and Procedures

During the study, there will be a total of up to 16 study site visits: Pretreatment Screening Visit (screening procedures can be performed on more than one day if necessary); Pretreatment Baseline Day -1 Visit; Treatment Period #1 Day 1 and Weeks 2, 6, 12, 18, and 24 Visits, and Week 24 Follow-up Visit; Transition Period Week 28 Visit; Treatment Period #2 Weeks 30, 34, 40, 48 Visits, and Week 48 Follow-up Visit; and final Dose-tapering Period Week 52 Visit. In addition, to facilitate completion of all required Week 24 and Week 48 assessments, some of the assessments scheduled for the Week 24 and Week 48 Visits may be completed on the day following the Week 24 (Week 48) dose of study medication, the day of the Week 24 (Week 48) Follow-up Visit, or in some cases up to 7 days following the date of the Week 24 (Week 48) dose of study medication, if needed (see **Section 6.3.4** and **Section 6.3.6**).

Each subject will receive the double-blind study medication at stable daily dose for an initial period of 24 weeks (Treatment Period #1). Following completion of the 24-week Treatment Period #1, all subjects will continue to receive the suspension formulation (vamorolone or matching placebo) at the same dose they received during the Treatment Period #1, while tapering the number of tablets (prednisone or matching placebo) during the 4-week double-blind Transition Period, and will return to the study site for study assessments at the end of the Transition Period (Week 28), prior to receiving the first dose of study medication for Treatment Period #2 at home on Week 28 + 1 day.

Each subject will receive double-blind study medication at stable daily dose for a period of 20 weeks (Treatment Period #2). Following completion of the 20-week Treatment Period #2, all subjects who will not be continuing to receive vamorolone in an additional vamorolone study or general access program (separate protocol) will taper their liquid formulation study medication during the 4-week double-blind Dose-Tapering Period, and will return to the study site for study assessments at the end of the Dose-Tapering Period (Week 52). See **Section 7** for a detailed description of the safety, clinical efficacy, PD, and PK assessments to be performed in this study.

### *6.3.1 Screening Period (Day -33 to -2)*

The Investigator or study staff will discuss with each subject and the subject's parent(s) or legal guardian(s) the nature and purpose of the study and the required study procedures. The subject's medical history and medication history will be reviewed to determine initial eligibility for participation in the study and the subject's de-identified dystrophin genetic test report and/or muscle biopsy report will be sent to the Central Genetic Counselor(s) for confirmation that the subject meets the DMD diagnostic inclusion criteria.

Following the signing of the written ICF, subjects will be considered to be enrolled in the study, and will be assigned a unique site-specific 6-digit subject study number that will be comprised of protocol, site, and subject numbers in sequential order of screening into the study. All data will be identified using the unique subject study number. The site Investigator will keep a record relating the names of the subjects to their subject study

numbers (subject identification log) to permit efficient verification of data subject files, when required. This record will also include the dates of subject enrollment and completion/termination. The Coordinating Center will not collect names or other identifiers except dates (diagnosis, study visits), date of birth, and the subject study number.

Subjects will undergo the procedures in the bulleted list below during the Screening Period. The procedures may be completed over the course of several visits, if necessary, but all scheduled Screening procedures must be completed within the timeframe of Day -33 to Day -11, and the actual date each procedure is performed must be recorded in the source document and eCRF. Any parameter/test may be repeated at the Investigator's discretion during Pretreatment Screening to determine reproducibility. In addition, subjects may be rescreened if ineligible due to a transient condition which would prevent the subject from participating, such as an upper respiratory tract infection.

Subjects meeting all Screening eligibility tests will be randomized to treatment during the Screening Period.

- Review of the Inclusion and Exclusion Criteria (see **Sections 4.2** and **4.3**)
- Recording of the medical history, including any toxicities or allergy-related events to prior treatments (see **Section 7.2.1**)
- Recording of prior medications (Medication History) (see **Section 5.7.1)**
- Complete physical examination, including weight (in kilograms) and height (in cm) (see **Section 7.2.2**)
- Recording of vital signs (sitting blood pressure, heart rate, body temperature, respiratory rate) (see **Section 7.2.3**)
- Collection of blood for clinical laboratory testing (hematology, clinical chemistry, lipids, HbA1c, and vitamin D) and collection of urine for urinalysis (see **Section 7.2.4**)
- Testing for chicken pox immunity (see **Section 7.2.5**)

- 12-lead ECG (see **Section 7.2.10**)
- 2D-echocardiogram (see **Section 7.2.11)**
- Time to Stand Test (TTSTAND) (see **Section 7.3.1**)
- Time to Climb Test (TTCLIMB) (see **Section 7.3.2)**
- Time to Run/Walk Test (TTRW) (see **Section 7.3.3**)
- North Star Ambulatory Assessment (NSAA) (see **Section 7.3.4**)
- Six-minute Walk Test (6MWT) (see **Section 7.3.5**)
- Hand-held myometry (elbow flexors and knee extensors) (see **Section 7.3.6**)
- Range of Motion (ROM) in the ankles (see **Section 7.3.7**)
- Eye exam (see **Section 7.2.12**)
- DXA scan (see **Section 7.2.13**)
- Spine X-rays (see **Section 7.2.14**)
- ACTH Stimulation Test (see **Section 7.2.7**)
- Pediatric Outcomes Data Collection Instrument questionnaire (PODCI) (see **Section 7.4.1**)
- PARS III questionnaire (see **Section 7.4.3**)
- Recording of AEs and SAEs beginning at the time written informed consent is obtained (see **Section 7.5)**
- Randomization (see **Section 3.2**)

### *6.3.2 Baseline Period (Day -1) Visit*

Subjects who have met all study eligibility criteria and been randomized to treatment via IXRS during the Screening Period, and for whom subject-specific blinded study medication has been shipped to and received by the study site will return to the study site during the Pretreatment Baseline Period (Day -1, the 24-hour interval immediately

preceding administration of the first dose of study medication) for baseline assessments. Subjects will retain their 6-digit study identification number which was assigned during the Screening Period.

The following procedures will be completed at the Baseline Day -1 Visit:

- Physical examination including weight (in kilograms) and assessment of cushingoid features (see **Section 7.2.2**)
- Recording of vital signs (sitting blood pressure, heart rate, body temperature, respiratory rate) (see **Section 7.2.3**)
- Time to Stand Test (TTSTAND) (see **Section 7.3.1**)
- Time to Climb Test (TTCLIMB) (see **Section 7.3.2**)
- Time to Run/Walk Test (TTRW) (see (see **Section 7.3.3**)
- North Star Ambulatory Assessment (NSAA) (see **Section 7.3.4**)
- Six-minute Walk Test (6MWT) (see **Section 7.3.5**)
- Hand-held myometry (elbow flexors and knee extensors) (see **Section 7.3.6**)
- Range of Motion (ROM) in the ankles (see **Section 7.3.7**)
- Recording of AEs and SAEs; review of all AEs for resolution status and date (see **Section 7.5**)
- Recording of prior medications (Medication History) (see **Section 5.7.1**)

### *6.3.3 Treatment Period #1 Day 1 Visit*

At the Treatment Period #1 Day 1 Visit, certain procedures will be performed prior to administration of the first dose of study drug and are listed in this section. Treatment Period #1 Day 1, for purposes of the study analyses, begins with administration of the first dose of study medication.

Subjects will take the first dose of study medication in clinic on the day after the Pretreatment Baseline Visit. Subjects must have fasted ≥ 6 hours prior to arrival at the

study site for the Day 1 Visit. Breakfast, including at least 8 g of fat (8 ounces [240 mL] of full-fat milk or equivalent high-fat food portion) will be served at the study site after the blood and urine collections for clinical laboratory tests and the blood draw for PD biomarkers, including insulin and glucose, and within 30 minutes prior to administration of the dose of study medication.

Subjects will receive a medical "alert" card stating that participation in the study may increase the subjects' risk of adrenal suppression. The card will include instructions for families and clinicians regarding management of possible adrenal suppression during emergencies, including coverage with "stress doses" of hydrocortisone (or prednisone) during times of illness, injury, or surgery.

The following procedures will be completed at the Treatment Period #1 Day 1 Visit:

- Recording of vital signs (sitting blood pressure, heart rate, body temperature, respiratory rate) prior to administration of first dose of study drug (see **Section 7.2.3**)
- Clinical laboratory evaluation including hematology, clinical chemistry, lipids, and urinalysis tests, prior to administration of first dose of study drug (see **Section 7.2.4**)
- Blood samples for fasted glucose and insulin, prior to administration of first dose of study drug (see **Section 7.2.6**)
- Blood samples for PD biomarkers including osteocalcin, CTX1, serum aminoterminal propeptide of type I collagen (P1NP), and cortisol, prior to administration of first dose of study drug (see **Section 7.2.6**). Blood remaining from collected samples not needed for protocol-specified analyses may be stored for future exploratory biomarker studies.
- Dispensing of study medication and administration of first dose (see **Section 5.8.1** and **Section 5.3**, respectively)
- Dispensing of subject diary (see **Section 7.4.6**)

- Recording of AEs and SAEs; review of all AEs for resolution status and date (see **Section 7.5**)
- Recording of concomitant medications (see **Section 5.7**)

On Day 1, the subject will be discharged from the clinic after completion of all scheduled assessments.

### *6.3.4 Treatment Period #1 (Weeks 1-24)*

Subjects will return to the study site for safety, efficacy, and PD assessments beginning at Week 2 and continuing through the Week 24 Follow-up Visit, according to the schedule of visits in **Table 11.**

Subjects will continue to receive daily oral administration of vamorolone/placebo suspension and prednisone/placebo tablets throughout the 24-week Treatment Period #1. The daily dose of study medication should be taken with breakfast, including at least 8 g of fat (8 ounces [240 mL] of full-fat milk or equivalent high-fat food portion).

Dosing is to occur at home throughout the 24-week Treatment Period #1, except at the Weeks 2, 12 and 24 study visits when dosing will occur at the study site. Subjects must have fasted ≥ 6 hours prior to arrival at the study site for the Weeks 2, 12, and 24 study visits. Breakfast, including at least 8 g of fat (8 ounces [240 mL] of full-fat milk or equivalent high-fat food portion) will be served at the study site after the blood and urine collections for clinical laboratory tests and the blood draws for PD biomarkers, including insulin and glucose (Weeks 12 and 24 only), and within 30 minutes prior to administration of the dose of study medication. Ease of administration of the suspension study medication will be assessed at the Weeks 2, 12, and 24 Visits. Apart from blood and urine sample collections, all other scheduled assessments should be performed after administration of the study medication in clinic.

Study medication will be dispensed at Weeks 6, 12, 18, and at the Week 24 Follow-up Visit, and returned at Weeks 2 (compliance monitoring only; will be redispensed at end of visit), 6, 12, 18, and 24 for all subjects. Subjects will receive subject diaries at each study visit and return the diaries at each subsequent visit. Diaries will be reviewed with

the subject's parent or guardian by the study staff to assess AEs, changes to concomitant medications/therapies, and any missed or incomplete doses of study medication.

Limited safety assessments will be conducted at the Week 2 Visit.

Clinical efficacy assessments (TTSTAND, TTRW, TTCLIMB, NSAA, 6MWT, hand-held myometry, and ROM) and the subject reported outcomes (TSQM, PODCI, Ease of Study Medication Administration Assessment, and PARS III) will be conducted as specified in the schedule of study activities (**Table 11**). Weight will be recorded at every visit and height will be measured at 12-week intervals. Vital signs will be recorded at each study visit. A physical examination including assessment of cushingoid features will be performed every 6 weeks. A 12-lead ECG will be recorded at Weeks 12 and 24. 2D-echocardiography will be performed at Week 24. Blood and urine samples for clinical laboratory tests and blood for the serum PD biomarker panel will be collected at scheduled visits throughout the study (**Table 11**). Blood will be collected at Week 24 for DNA testing for candidate genetic modifiers of DMD. An eye examination to exclude cataracts and glaucoma will be performed at Week 24. A DXA scan and spine X-ray will be performed at Week 24. A Blindedness Assessment will be completed by the parent(s)/guardian(s) at the Week 24 Visit. Adverse events, including SAEs, and concomitant medications will be assessed at each study visit and recorded throughout the study.

There is flexibility in the timing of completion of some of the scheduled Week 24 assessments. The scheduled physical examination, weight, vital signs, clinical laboratory tests, blood draws for PD biomarker analysis and DNA testing, Ease of Study Medication Administration Assessment, PODCI, PARS III, and functional assessments (TTSTAND, TTCLIMB, TTRW, NSAA, 6MWT, hand-held myometry, ROM) must all be performed on the date of the final Week 24 dose of Treatment Period #1 study medication. The 12-lead ECG may be performed on the date of the final Week 24 dose of study medication, the day following the final Week 24 dose of study medication, or the day of the Week 24 Follow-up Visit. Completion of the DXA scan, spine X-ray, Fracture Questionnaire, 2-D echocardiography, eye examination, TSQM, and Blindedness

Assessment may be performed on the date of the final Week 24 dose of study medication or up to 7 days following the date of the final Week 24 dose of study medication to accommodate need for additional scheduling flexibility.

At the Week 24 Visit, all subjects will be dispensed a single dose of oral hydrocortisone (5 mg or 10 mg) to be administered 24 hours after administration of the final dose of study medication at the Week 24 Visit. The hydrocortisone dose will be approximately 8 mg/m$^2$, rounded up to either 5 mg or 10 mg; subjects will be provided with either a single 5 mg or 10 mg hydrocortisone tablet which will be dispensed by the site staff at the Week 24 Visit (see Manual of Operations for details).

Subjects will return to the study site approximately 48 hours after administration of the Week 24 dose of Treatment Period #1 study medication (and approximately 24 hours after the oral hydrocortisone) for ACTH Stimulation testing. The ACTH Stimulation Test will be performed in the morning, before 12 noon local time, 48 ± 3 hours after administration of the final dose of Treatment Period #1 study medication, and prior to administration of the first dose of study drug in the Transition Period (see Manual of Operations for details).

At the end of the 24-week Treatment Period, including the Week 24 Follow-up Visit for the ACTH Stimulation Test, all subjects will begin a 4-week double-blind Transition Period during which the doses of the tablet study medication will be progressively reduced and discontinued (see **Section 6.3.5**). Subjects will take the first doses of study medication in the Transition Period with a high-fat meal on the same day as the Week 24 Follow-up Visit ACTH Stimulation Test, as soon as possible after ACTH Stimulation testing has been completed.

### *6.3.5 Transition Period (Weeks 25-28)*

All subjects will participate in the 4-week double-blind dose Transition Period. During this period, all subjects will continue on the same dose of their liquid formulation (either vamorolone or matching placebo) as they were administered during Treatment Period #1 and will have dose-tapering of their prednisone or matching placebo tablets as outlined in

Table 12. This tapering is to aid in re-establishment of adrenal function if adrenal suppression has occurred in the prednisone-treated patients.

**Table 12. Tablet Dose Tapering**

| Treatment Period Dose (No. tabs) | Week 25 Dose (No. tabs) | Week 26 Dose (No. tabs) | Week 27 Dose (No. tabs) | Week 28 Dose (No. tabs) |
|---|---|---|---|---|
| 2 | 1 | 0 | | |
| 3 | 2 | 1 | 0 | |
| 4 | 3 | 2 | 1 | 0 |
| 5 | 3 | 2 | 1 | 0 |

No. = number; tabs = tablets.

Subjects will take the first doses of study medication in the Transition Period with a high-fat meal on the same day as the Week 24 Follow-up Visit ACTH Stimulation Test, as soon as possible after ACTH Stimulation testing has been completed.

Site study staff will contact the parent(s)/guardian(s) by telephone at Week 26 to ensure that the tablet tapering is proceeding according to protocol, to assess potential signs or symptoms indicative of adrenal suppression, and to address any questions the parent(s)/guardian(s) may have. In addition, subjects will be assessed promptly for adrenal suppression if unwell at any time during the Transition Period. There will be a low threshold for recommending commencement of daily oral prednisone or hydrocortisone, or intravenous hydrocortisone if hospitalization occurs in these circumstances.

Each subject will return to the study site for Week 28 safety study assessments.

Subjects must have fasted ≥ 6 hours prior to arrival at the study site for the Week 28 Visit. Breakfast will be served at the study site after the blood and urine collection for clinical laboratory tests and the blood draw for PD biomarkers, including fasting glucose and insulin. At the Week 28 Visit, subjects will also have a physical examination with weight, assessment of cushingoid features and vital signs recorded. Study medication will be returned for compliance monitoring. Adverse events, including SAEs, and concomitant medications will be assessed. Subject diaries will be returned and reviewed with site staff.

### 6.3.6 *Treatment Period #2 (Week 28 + 1 Day through Week 48)*

Subjects will take the first dose of study medication in Treatment Period #2 at home on the day after the Week 28 Visit (Week 28 + 1 day). There is no scheduled study visit at Week 28 + 1 day.

Subjects will return to the study site for safety, efficacy, and PD assessments beginning at Week 30 and continuing through the Week 48 Follow-up Visit, according to the schedule of visits in **Table 11**. Population PK assessments will be performed on blood collected at the Week 30 Visit only.

Subjects will receive daily oral administration of vamorolone suspension throughout the 20-week Treatment Period #2, from Week 28+ 1 day through the day of the Week 48 Visit. The daily dose of study medication should be taken with breakfast, including at least 8 g of fat (8 ounces [240 mL] of full-fat milk or equivalent high-fat food portion).

Dosing is to occur at home throughout the 20-week Treatment Period #2 except at the Weeks 30, 40, and 48 study visits when dosing will occur at the study site. Subjects must have fasted ≥ 6 hours prior to arrival at the study site for the Weeks 30, 40, and 48 study visits. Breakfast, including at least 8 g of fat (8 ounces [240 mL] of full-fat milk or equivalent high-fat food portion) will be served at the study site after the blood and urine collections for clinical laboratory tests and the blood draws for PD biomarkers, including insulin and glucose (Weeks 40 and 48 only), and within 30 minutes prior to administration of the dose of study medication. Ease of suspension study medication administration will be assessed at the Weeks 30, 40, and 48 Visits. Apart from blood and urine sample collections, all other scheduled assessments should be performed after administration of the study medication in clinic.

Study medication will be dispensed at Weeks 34, 40, and at the Week 48 Follow-up Visit (for subjects participating in the Dose-tapering Period only), and returned at Weeks 30 (compliance monitoring only; will be redispensed at end of visit), 34, 40, and 48 for all subjects. Subjects will receive subject diaries at each study visit and return the diaries at each subsequent visit. Diaries will be reviewed with the subject's parent or guardian by

the study staff to assess AEs, changes to concomitant medications/therapies, and any missed or incomplete doses of study medication.

Limited safety assessments and blood draw for population PK analysis will be conducted at the Week 30 Visit.

Clinical efficacy assessments (TTSTAND, TTRW, TTCLIMB, NSAA, 6MWT, hand-held myometry, and ROM) and the subject reported outcomes (TSQM, PODCI, Ease of Study Medication Administration Assessment, and PARS III) will be conducted as specified in the schedule of study activities (**Table 11**). Weight will be recorded at every visit and height will be measured at Weeks 34 and 48. Vital signs will be recorded at each study visit. A physical examination including assessment of cushingoid features will be performed at Weeks 34, 40, and 48. A 12-lead ECG will be recorded at Weeks 40 and 48. 2D-echocardiography will be performed at Week 48. Blood and urine samples for clinical laboratory tests and blood for the serum PD biomarker panel will be collected at scheduled visits throughout Treatment Period #2 (**Table 11**). A blood sample for plasma PK will be collected at Week 30, two hours following dosing. An eye examination to exclude cataracts and glaucoma will be performed at Week 48. A DXA scan will be performed at Week 48. Adverse events, including SAEs, and concomitant medications will be assessed at each study visit and recorded throughout the study.

There is flexibility in the timing of completion of some of the scheduled Week 48 assessments. The scheduled physical examination, weight, vital signs, clinical laboratory tests, blood draws for PD biomarker analysis, Ease of Study Medication Administration Assessment, PODCI, PARS III, and functional assessments (TTSTAND, TTCLIMB, TTRW, NSAA, 6MWT, hand-held myometry, ROM) must all be performed on the date of the final Week 48 dose of Treatment Period #2 study medication. The 12-lead ECG may be performed on the date of the final Week 48 dose of study medication, the day following the final Week 48 dose of study medication, or the day of the Week 48 Follow-up Visit. Completion of the DXA scan, Fracture Questionnaire, 2-D echocardiography, eye examination, and TSQM, may be performed on the date of the final Week 48 dose of study medication, the day following the Week 48 dose of study medication, or the day of

the Week 48 Follow-up Visit (for subjects receiving vamorolone therapy by enrolling directly into an additional vamorolone study or general access program), or up to 7 days following the date of the final Week 48 dose of study medication (for subjects participating in the Dose-tapering Period) to accommodate need for additional scheduling flexibility.

At the Week 48 Visit, all subjects will be dispensed a single dose of oral hydrocortisone (5 mg or 10 mg) to be administered 24 hours after administration of the final dose of study medication at the Week 48 Visit. The hydrocortisone dose will be approximately 8 mg/m$^2$, rounded up to either 5 mg or 10 mg; subjects will be provided with either a single 5 mg or 10 mg hydrocortisone tablet which will be dispensed by the site staff at the Week 48 Visit (see Manual of Operations for details).

Subjects will return to the study site approximately 48 hours after administration of the final (Week 48) dose of Treatment Period #2 study medication (and approximately 24 hours after the oral hydrocortisone) for ACTH Stimulation testing. The ACTH Stimulation Test will be performed in the morning, before 12 noon local time, 48 ± 3 hours after administration of the final Treatment Period #2 dose of study medication, and prior to administration of the first dose of study drug in the Dose-tapering Period if the subject is to taper, or prior to the first dose of study medication in the additional vamorolone study or general access program for subjects transitioning directly to that protocol (see Manual of Operations for details).

At the end of the 20-week Treatment Period #2, including the Week 48 Follow-up Visit for the ACTH Stimulation Test, subjects may be given the option of continuing vamorolone therapy by enrolling into an additional vamorolone study or general access program. Subjects enrolling directly into an additional vamorolone study or general access program will not need to taper their vamorolone dose prior to enrollment. All other subjects will begin a 4-week double-blind Dose-tapering Period during which the doses of suspension study medication will be progressively reduced and discontinued (see **Section 6.3.7**).

Subjects who will not participate in the Dose-tapering Period (see **Section 6.3.7**) will be discharged from the study following completion of all Week 48 assessments, including the Week 48 Follow-up Visit ACTH Stimulation Test. Subjects who do participate in the Dose-tapering Period will be dispensed vamorolone at the Week 48 Follow-up Visit, as well as instructions for tapering the dose of vamorolone during the Dose-tapering Period. Subjects will take the first dose of study medication in the Dose-tapering Period with a high-fat meal on the same day as the Week 48 Follow-up Visit ACTH Stimulation Test, as soon as possible after ACTH Stimulation testing has been completed.

### *6.3.7 Dose-tapering Period (Weeks 49-52)*

All subjects who complete the study and opt not to continue vamorolone therapy by enrolling into an additional vamorolone study or general access program will participate in a 4-week double-blind Dose-tapering Period during which the doses of suspension study medication will be progressively reduced and discontinued. In addition, subjects who discontinue study medication after Week 28 and prior to Week 48 will also participate in the Dose-tapering Period if possible and if, in the opinion of the Investigator, it is safe to do so. The purpose of dose-tapering is to aid in re-establishment of adrenal function if adrenal suppression has occurred during vamorolone treatment. Dose tapering will be performed in a stepwise manner, according to the subject's most recent calculated liquid formulation dose during the 20-week Treatment Period #2.

Dose tapering for the liquid formulation (vamorolone) will be performed as outlined in **Table 13**. For subjects who have completed Treatment Period #2, the subject's weight recorded at the Week 40 Visit will be used to calculate dose volume for all dose de-escalations during the Dose-tapering Period.

**Table 13. Suspension Dose Tapering**

| Treatment Period #2 Dose Level | Week 49 Dose Level | Week 50 Dose Level | Week 51 Dose Level | Week 52 Dose Level |
|---|---|---|---|---|
| Formulation: 100% | 50% | 25% | 10% | 0% |

Subjects will take the first dose of study medication in the Dose-tapering Period with a high-fat meal on the same day as the Week 48 Follow-up Visit ACTH Stimulation Test, as soon as possible after ACTH Stimulation testing has been completed.

Site study staff will contact the parent(s)/guardian(s) by telephone at Week 50 to ensure that the dose tapering is proceeding according to protocol, to assess potential signs or symptoms indicative of adrenal suppression, and to address any questions the parent(s)/guardian(s) may have. In addition, subjects will be assessed promptly for adrenal suppression if unwell at any time during the Dose-Tapering Period. There will be a low threshold for recommending commencement of daily oral prednisone or hydrocortisone, or intravenous hydrocortisone if hospitalization occurs in these circumstances.

The final end-of-study visit will be scheduled approximately one week after the final dose de-escalation. Each subject will return to the study site for final study assessments when he has received no suspension study medication for one week (Study Week 52).

Subjects must have fasted ≥ 6 hours prior to arrival at the study site for the final Week 52 Dose-tapering Visit. Breakfast will be served at the study site after the blood and urine collection for clinical laboratory tests and the blood draw for PD biomarkers, including fasting glucose and insulin. At the final Week 52 Visit, subjects will also have a physical examination with weight, assessment of cushingoid features and vital signs recorded. Study medication will be returned for compliance monitoring. Adverse events, including SAEs, and concomitant medications will be assessed. Subject diaries will be returned and reviewed with site staff.

Subjects participating in the Dose-tapering Period will be discharged from the study following completion of all Dose-tapering Period assessments.

## 6.4 Subject Discontinuation

In the event that a subject withdraws early from the study prior to the Week 48 Visit, the reason for discontinuation must be fully documented in the source documents and the eCRF.

Any subject who withdraws from the study prior to the Week 24 Visit should return to the study site for Week 24 assessments and the Week 24 Follow-up Visit ACTH Stimulation Test at the time of early withdrawal, whenever possible (see **Section 6.3.4**); any subject who prematurely discontinues from the study after Week 24 but prior to Week 28 should complete the Week 28 assessments at the time of early withdrawal, whenever possible (see **Section 6.3.5**); and any subject who prematurely discontinues from the study after Week 28 but prior to Week 48 should complete the Week 48 assessments and the Week 48 Follow-up Visit ACTH Stimulation Test at the time of early withdrawal, whenever possible (see **Section 6.3.6**), assuming the subject has not withdrawn consent. Site personnel will document all assessments, including any AEs, in the source documents and eCRF.

In the event a subject withdraws informed consent, no further study procedures should be performed and no additional data should be collected. Any data collected up to the point of withdrawal of informed consent may be used by the Sponsor. Every effort will be made to ensure that subjects who withdraw consent undergo dose-tapering, as appropriate, prior to the date of withdrawal of consent. Subjects who withdraw early from the study may be replaced, at the discretion of the Sponsor.

Subjects who discontinue study medication should follow the procedures for the applicable Early Discontinuation Dose-tapering Period described below and detailed in the Manual of Operations, whenever possible. Dose tapering for subjects who discontinue the study early is to aid in re-establishment of adrenal function if adrenal suppression has occurred in the prednisone and/or vamorolone treated patients.

Subjects will be assessed promptly if unwell during the tapering phase or in the weeks following study medication cessation due to the risk of adrenal suppression. There will be a low threshold for recommending commencement of daily oral prednisone or hydrocortisone, or intravenous hydrocortisone if hospitalization occurs in these circumstances.

The final end-of-study visit will be scheduled approximately one week after the final dose de-escalation. Each subject will return to the study site for final study assessments when he has received no suspension and/or tablet study medication for one week.

Subjects must have fasted ≥ 6 hours prior to arrival at the study site for the final Early Discontinuation Dose-tapering Visit. Breakfast will be served at the study site after the blood and urine collection for clinical laboratory tests and the blood draw for PD biomarkers, including fasting glucose and insulin. At the final Study Visit, subjects will also have a physical examination with weight, assessment of cushingoid features and vital signs recorded. Study medication will be returned for compliance monitoring. Adverse events, including SAEs, and concomitant medications will be assessed. Subject diaries will be returned and reviewed with site staff.

### 6.4.1 Early Discontinuation Prior to Week 24

Any subject who discontinues the study after Day 1 and prior to the Week 24 Visit should return to the study site for Week 24 assessments and the Week 24 Follow-up ACTH Stimulation Test at the time of early withdrawal, and will participate in a 4-week Early Discontinuation Dose Tapering Period, whenever possible. Dose tapering will be performed in a stepwise manner, according to the subject's most recently calculated liquid and tablet formulation doses during the 24-week Treatment Period #1.

Dose tapering for tablets (prednisone and matching placebo) will be performed as outlined in **Table 14**.

**Table 14. Tablet Dose Tapering for Subjects Discontinuing Prior to Week 24**

| Treatment Period Dose (No. tabs) | Week 25 Dose (No. tabs) | Week 26 Dose (No. tabs) | Week 27 Dose (No. tabs) | Week 28 Dose (No. tabs) |
|---|---|---|---|---|
| 2 | 1 | 0 | | |
| 3 | 2 | 1 | 0 | |
| 4 | 3 | 2 | 1 | 0 |
| 5 | 3 | 2 | 1 | 0 |

No. = number; tabs = tablets.

Dose tapering for the liquid formulation (vamorolone and matching placebo) will be performed as outlined in **Table 15**.

**Table 15. Suspension Dose Tapering for Subjects Discontinuing Prior to Week 24**

| Treatment Period Dose Level | Week 25 Dose Level | Week 26 Dose Level | Week 27 Dose Level | Week 28 Dose Level |
|---|---|---|---|---|
| Formulation: 100% | 50% | 25% | 10% | 0% |

As soon as possible after ACTH Stimulation testing has been completed at the Week 24 Follow-up/Early Termination Visit, subjects will take the first doses of study medication in the Early Discontinuation Dose Tapering Period with a high-fat meal on the same day.

### *6.4.2 Early Discontinuation After Week 24 and Prior to Week 28*

Subjects who discontinue from the study after Week 24 and prior to Week 28 will continue on the same schedule for dose tapering of tablets they are already following for Transition Period dosing, and will begin the 4-week suspension dose tapering schedule shown in **Table 16**.

**Table 16. Suspension Dose-Tapering for Subjects Discontinuing After Week 24 and Prior to Week 28**

| | **Formulation Dose Level (% of Volume Administered During Treatment Period #1) at Each Week Following Discontinuation** | | | | |
|---|---|---|---|---|---|
| **Time of Early Discontinuation** | **100%** | **50%** | **25%** | **10%** | **0%** |
| | | | | | |
| **Week 25** | **Week 25** | **Week 26** | **Week 27** | **Week 28** | **Week 29** |
| | | | | | |
| **Week 26** | **Week 26** | **Week 27** | **Week 28** | **Week 29** | **Week 30** |
| | | | | | |
| **Week 27** | **Week 27** | **Week 28** | **Week 29** | **Week 30** | **Week 31** |
| | | | | | |

Each subject will return to the study site for final study assessments when he has received no suspension or tablet study medication for one week (Study Week 29, Week 30, or Week 31, depending on when the subject discontinued [see **Table 16**]).

### *6.4.3 Early Discontinuation After Week 28 and Prior to Week 48*

Subjects who discontinue from the study after Week 28 and before the Week 48 Visit will follow the same schedule for dose tapering of suspension as described in **Section 6.3.7.**

## 6.5 Subject and Study Completion

A completed subject is defined as a subject who has completed Treatment Period #1 and Treatment Period #2, through the Week 48 and Week 48 Follow-up Visit assessments, and Dose-tapering Period, if applicable, and has not prematurely withdrawn from the study for any reason. The study will be completed when the final subject has completed his final study visit ("last subject, last visit").

# 7 STUDY ASSESSMENTS AND MEASUREMENTS

## 7.1 Demographic Assessments

Demographic information (birth date, race, and ethnicity) will be collected during the Pretreatment Screening Period and will be recorded on the appropriate eCRF page.

### *7.1.1 Genetic Modifiers of DMD*

Approximately 6 mL of blood will be collected at the Week 24 Visit for DNA testing to determine if candidate genetic modifiers of DMD (gene polymorphisms associated with disease severity or response to glucocorticoid treatment) are similarly associated with vamorolone-treated DMD patients (baseline disease severity or response to vamorolone or prednisone treatment).

DNA testing will be performed by a certified central laboratory.

The procedures for the collection, handling, and shipping of blood samples for DNA testing will be specified in the Laboratory Manual(s) provided to the clinical center.

## 7.2 Safety and Tolerability Assessments

### *7.2.1 Medical History*

The medical history will be recorded at the Screening Visit and will include significant past medical or surgical procedures as well as previous and current co-existent diseases. It should include the date (month/year) the subject was diagnosed with DMD, initial symptoms of DMD and the age at which they were first identified, and any toxicities or allergies to prior treatments. It should include relevant medical history for the

following body systems: head, eyes, ears, nose and throat (HEENT), respiratory, cardiovascular, gastrointestinal, endocrine, hematological, dermatological, genital-urinary, neurological, musculoskeletal, psychological/psychiatric, and any other history of medical significance. The medical history will be recorded on the appropriate eCRF page.

### *7.2.2 Physical Examination, Cushingoid Features, Weight, and Height*

A complete physical examination will be performed at Screening, Baseline Day -1 and every 6 weeks thereafter through Treatment Period #1, at the Week 28 Transition Period Visit, at Weeks 34, 40, and 48 of Treatment Period #2, and at the final Week 52 Dose-tapering Period Visit, and will include examination of the following: head, eyes, ears, nose, and throat, neck *(including an examination of the thyroid),* heart, lungs, abdomen *(including an examination of the liver and spleen),* lymph nodes, extremities, nervous system, and skin. Clinically significant changes from baseline should be recorded as AEs. Particular attention will be paid in identifying any sign of cushingoid features, which should also be recorded as AEs if they first appear or worsen during the study.

Additional unscheduled symptom-directed physical examinations may be conducted at any time at the Investigator's discretion.

Height (in cm) will be recorded at Screening, and Weeks 12, 24, 34, and 48. Weight (in kg) will be recorded at Screening, Baseline Day -1, Week 2, Week 6, Week 12, Week 18, Week 24, Week 28, Week 30, Week 34, Week 40, and Week 48, and at the final Week 52 Dose-tapering Visit (**Table 11**). Weight recorded at the previous visit will be used to calculate the study medication (suspension and tablets) dose for the subsequent dispensing interval (see **Section 5.3**).

Results will be recorded in the source documents and on the appropriate eCRF page.

### *7.2.3 Vital Signs*

Vital signs (sitting blood pressure, heart rate, respiration rate, and body temperature) will be recorded at Screening, Baseline Day -1, Day 1, Week 2, Week 6, Week 12, Week 18,

Week 24, Week 28, Week 30, Week 34, Week 40, and Week 48, and at the final Week 52 Dose-tapering Visit. Vital signs should be recorded after the subject has been resting for at least 5 minutes. Body temperature may be measured using oral, tympanic, or temporal recording devices; however, the same methodology must be used for all assessments of a given subject.

Results will be recorded in the source documents and on the appropriate eCRF page.

If vital signs are recorded at the same study visit as blood sampling and ECG recording, at least 15 minutes should elapse after collection of blood samples and before performing ECG and recording vital signs.

### *7.2.4 Clinical Laboratory Tests*

Each subject will have blood drawn and urine collected for the hematology, chemistry, lipids, and urinalysis clinical laboratory tests listed in **Table 17** and **Table 18**, below, during the Screening Period, at the Day 1 Visit, and at each of the subsequent study visits specified in **Table 11**. Blood for vitamin D and HbA1c are collected at specific visits only (**Table 17**). Fasted blood and urine samples for clinical laboratory tests will be collected pre-dose at the Day 1 and Weeks 2, 12, 24, 28, 30, 40, and 48 Visits, and fasted samples will be collected at the final Week 52 Dose-tapering Period Visit. Non-fasted blood and urine samples for clinical laboratory tests will be collected at the Screening, and Weeks 6, 18, and 34 Visits. Details of blood draws can be found in the Laboratory Manual.

All blood and urine samples will be sent to the designated central laboratory for testing.

For the hematology, chemistry, and lipids laboratory tests, blood will be collected by direct venipuncture of peripheral veins. A total of approximately 140 mL of blood will be collected over the course of this study for clinical safety laboratory evaluation (see **Section 7.2.9** for details of blood volumes to be collected).

If blood sampling is performed at the same study visit as vital signs assessment and ECG recording, at least 15 minutes should elapse after collection of blood samples and before performing ECG and recording vital signs.

Any abnormal hematology, chemistry, lipid, or urinalysis test result deemed clinically significant by the Investigator or medically qualified sub-investigator may be repeated, including test results obtained on the final study day.

Any treatment-emergent abnormal laboratory test result that is clinically significant, i.e., meeting one or more of the following conditions, should be recorded as a single diagnosis on the AE section of the eCRF:

- Accompanied by clinical symptoms
- Requiring a change in concomitant therapy (e.g., addition of, interruption of, discontinuation of, or any other change in a concomitant medication, therapy, or treatment)
- Is otherwise considered clinically significant by the Investigator

Any clinically significant test abnormality as defined above should be recorded as an AE (unless it was considered spurious), and repeat analysis performed until resolution or until the Investigator or medically qualified sub-investigator determines that resolution of the abnormality is not expected.

**Table 17. Hematology, Chemistry, and Lipids Clinical Laboratory Tests**

| | |
|---|---|
| **Hematology** | |
| Red Blood Cells (RBC) | Numerical platelet count (estimate not acceptable) |
| Hemoglobin | White Blood Cells (WBC) with differential (percent) |
| Hematocrit | |
| | |
| **Chemistry** | |
| Sodium | Total Bilirubin[a] |
| Potassium | Uric Acid |
| Chloride | Glucose |
| Calcium | Glutamate dehydrogenase (GLDH) |
| Inorganic Phosphorus | Alkaline phosphatase (ALP) |
| Blood Urea Nitrogen (BUN) | Gamma Glutamyl Transferase (GGT) |
| Creatinine | Aspartate aminotransferase (AST) |
| Total Protein | Alanine aminotransferase (ALT) |
| Albumin | Creatine kinase (CK) |
| Bicarbonate | Lipase |
| Lactate Dehydrogenase (LDH) | Amylase |
| Cystatin C | Vitamin D[b] |
| HbA1c[c] | |
| | |
| **Lipids** | |
| Triglycerides | Low Density Lipoprotein (LDL) |
| Total cholesterol | High density Lipoprotein (HDL) |

a. If outside normal range, direct bilirubin will be measured and reported.
b. Vitamin D levels measured at Screening, Weeks 12, 24, 40, and 48 only.
c. HbA1c levels measured at Screening, Week 24, and Week 48 only.

Urine will be collected for routine analysis, by dipstick and microscopic analysis, for the tests described in **Table 18**.

**Table 18. Urinalysis Clinical Laboratory Tests**

| Urinalysis (including microscopic examination) | |
|---|---|
| **Dipstick[a]** | **Microscopic Analysis** |
| Protein | WBC/hpf |
| Glucose | RBC/hpf |
| Ketones | Casts |
| pH | Bacteria |
| Leukocyte esterase | |
| Blood | |
| a. A midstream clean-catch urine specimen will be collected for dipstick analysis. | |

Blood for hemoglobin A1c (HbA1c) determination is collected at the scheduled Screening, Week 24 and Week 48 Visits, and should also be collected if urine glucose is positive and/or fasted glucose levels are above normal limits (see **Section 7.2.6** and Laboratory Manual).

Clinical laboratory tests will be performed by a central laboratory; results will be reported to the study site and transferred electronically into the clinical study database.

The procedures for the collection, handling, and shipping of laboratory samples will be specified in the Laboratory Manual provided to the clinical center.

***Follow-up of Abnormal Laboratory Test Results***

In the event of a medically significant, unexplained, or abnormal clinical laboratory test value, the test(s) may be repeated, evaluated by the Investigator for sustainability and reproducibility to determine if the abnormality represents an AE, and followed-up until the results have returned to the normal range, stabilized, and/or an adequate explanation for the abnormality is found. If a clear explanation is established, it should be recorded in the source documents and eCRF. The clinical laboratory will clearly mark all laboratory test values that are outside the normal range and the Investigator will indicate which of these deviations are clinically significant. These clinically significant deviating laboratory results will then be further described as AEs, and the relationship to the treatment, in the Investigator's opinion, will be indicated (see **Section 7.5**).

### *7.2.5 Chicken Pox Immunity*

At Screening, a 2 mL blood sample will be collected for antibodies (IgG) to Varicella Zoster virus to confirm immunity. The blood sample will be sent to the local laboratory for testing.

If antibodies are not detected in the blood sample sent to the local laboratory, immunization before starting the trial will be advised and the immunization status must be re-checked prior to randomization (see Manual of Operations for details). Lack of willingness to immunize a child who is not already immune to chicken pox will be a reason for exclusion of the child from the trial.

### *7.2.6 Pharmacodynamic Biomarker Panel*

Blood samples will be collected to explore the effect of vamorolone on biomarkers associated with glucocorticoid safety concerns (secondary outcomes for adrenal suppression, insulin resistance, bone turnover and immune suppression), as listed in **Table 19**.

Blood samples will be collected pre-dose at the Day 1 and Weeks 12, 24, 28, 40, and 48 Visits, and at the final Week 52 Dose-tapering Period Visit for analysis of biomarkers for secondary outcome measures of adrenal suppression, bone turnover, and insulin resistance. Approximately 2 mL of blood will be collected for the PD biomarker panel (osteocalcin, P1NP, and CTX bone turnover markers) at each scheduled collection time point; blood samples for analysis of morning cortisol levels (adrenal suppression biomarker) and fasting glucose and insulin (insulin resistance biomarkers) will be collected as part of the clinical laboratory tests and require no additional blood volume. All samples will be collected after the subject has fasted for ≥ 6 hours and prior to administration of the daily dose of study medication at dosing visits. Blood samples for analysis of immune suppression (differential lymphocyte percentage) will be collected as part of the clinical laboratory tests (see **Section 7.2.4**). Blood remaining from collected samples not needed for protocol-specified analyses at each of these time points may be stored for future exploratory biomarker studies for aspects of safety and efficacy. These remaining blood samples may be released to scientists worldwide for research purposes,

including research on biomarkers in DMD. Any released samples will have no identifying subject information.

Blood for HbA1c determination should be collected if urine glucose is positive and/or fasted glucose levels are above normal limits at any of the scheduled assessment time points (see **Section 7.2.4** and Laboratory Manual).

A total of approximately 14 mL of blood will be collected for the PD biomarker panel (osteocalcin, P1NP, and CTX bone turnover markers) over the course of the 57-week study (see **Section 7.2.9**).

**Table 19. Pharmacodynamic Biomarkers – Secondary Safety Outcomes**

| **Adrenal Suppression** |
|---|
| Cortisol - morning |
| **Insulin Resistance** |
| Glucose – fasting |
| Insulin - fasting |
| **Bone Turnover** |
| Osteocalcin |
| CTX1 |
| P1NP |
| **Immune Suppression** |
| Differential lymphocyte percentage |

### *7.2.7 ACTH Stimulation Test*

The ACTH Stimulation Test will be performed in the morning during the Screening Visit, in the morning of the Week 24 Follow-up Visit (48 ± 3 hours after the final dose of Treatment Period #1 study medication), and in the morning of the Week 48 Follow-up Visit (48 ± 3 hours after the final dose of Treatment Period #2 study medication) to assess the adrenal gland stress response.

All subjects will be given a single dose of hydrocortisone (5 mg or 10 mg) 24 hours after the dose of study medication at the Week 24 Visit and 24 hours after the dose of study medication at the Week 48 Visit. The hydrocortisone dose will be approximately 8 $mg/m^2$, rounded up to either 5 mg or 10 mg; subjects will be provided with either a single 5 mg or 10 mg hydrocortisone tablet which will be dispensed by the site staff at the Week 24 or Week 48 Visit (see Manual of Operations for details).

Subjects will return to the study site approximately 48 hours after administration of the final (Week 24) dose of Treatment Period #1 study medication (and approximately 24 hours after the dose of oral hydrocortisone), and approximately 48 hours after administration of the final (Week 48) dose of Treatment Period #2 study medication (and approximately 24 hours after the dose of oral hydrocortisone) for ACTH Stimulation testing. The ACTH Stimulation Test will be initiated as close to 8 AM local time as possible, but in any case completed in the morning, before 12 noon local time, 48 ± 3 hours after administration of the final Treatment Period #1 or Treatment Period #2 dose of study medication, and prior to administration of the first dose of study drug in the Transition Period (Week 24) or Dose-Tapering Period (Week 48) (see Manual of Operations for details).

The ACTH Stimulation Test involves insertion of a saline lock and then administration of 250 µg of Cosyntropin at time zero. Blood samples for cortisol measurement are collected at time 0 immediately prior to Cosyntropin administration, and at 30 ± 5 minutes and 60 ± 5 minutes after Cosyntropin administration.

The potential side effects of ACTH Stimulation testing (nausea, sweating, dizziness, palpitations, facial flushing) will be discussed with the subject and the family before starting the test. Cortisol levels below 18 µg/dL (equivalent to 500 nM) 30 or 60 minutes after stimulation with Cosyntropin will be considered indicative of adrenal suppression.

Approximately 2 mL of blood will be collected at each time point for cortisol measurement; a total of approximately 6 mL of blood will be collected during each of the Screening Visit, the Week 24 Follow-up Visit (48 ± 3 hours following the final dose of Treatment Period #1 study medication), and the Week 48 Follow-up Visit (48 ± 3 hours following the final dose of Treatment Period #2 study medication) for the ACTH Stimulation Test (see **Section 7.2.9**).

Blood samples will be sent to a central laboratory, and results centrally interpreted (Children's Hospital of Eastern Ontario). Results of the Week 24 analysis will not be reported to the study sites.

### *7.2.8 Population PK Assessment*

At the Week 30 Visit, all subjects will have blood collected for PK assessments at 2 hours post-dose. Approximately 2 mL of blood will be collected into $K_2$-EDTA tubes at the single assessment time point.

Plasma concentrations of vamorolone will be measured using a specific and validated liquid chromatography tandem mass spectrometry assay. PK assessments will be performed by a central laboratory. The procedures for the collection, handling, and shipping of laboratory samples will be specified in the Laboratory Manual(s) provided to the study sites.

The exact time of blood sampling will be recorded in the source document and eCRF.

If PK and PD or clinical laboratory blood samples are to be collected at the same time point, the PK blood sample should be collected prior to the PD blood sample(s), which in turn should be collected prior to the clinical laboratory blood samples.

### *7.2.9 Total Blood Volume Required*

The number and volume of blood samples and total volume of blood to be collected from each subject throughout the duration of the 57-week study are summarized in **Table 20**. A total of 182 mL of blood will be collected from each subject over the course of the up-to-57-week study.

## Table 20. Blood Sample Number and Volume by Study Visit

| Test | Total mL of Blood | | | | | | | | | | | | | | | |
|---|---|---|---|---|---|---|---|---|---|---|---|---|---|---|---|---|
| | SCR | Day 1 | Week 2 | Week 6 | Week 12 | Week 18 | Week 24 | Week 24 F/U | Week 28 | Week 30 | Week 34 | Week 40 | Week 48 | Week 48 F/U | Week 52 | Total Volume |
| Clinical Safety Labs[a] | 12[b] | 10[c] | 10[c] | 10 | 12[c] | 10 | 12[b,c] | | 10[c] | 10[c] | 10 | 12[c] | 12[b,c] | | 10[c] | 140[b] |
| Varicella Zoster IgG | 2 | | | | | | | | | | | | | | | 2 |
| PD Biomarker Panel[d] | | 2 | | | 2 | | 2 | | 2 | | | 2 | 2 | | 2 | 14 |
| PD Insulin/Glucose[c] | | From Clinical Safety Lab Sample | | | From Clinical Safety Lab Sample | | From Clinical Safety Lab Sample | | From Clinical Safety Lab Sample | | | From Clinical Safety Lab Sample | From Clinical Safety Lab Sample | | From Clinical Safety Lab Sample | |
| DNA Testing | | | | | | | 6 | | | | | | | | | 6 |
| ACTH Stimulation Test[e] | 6 | | | | | | | 6 | | | | | | 6 | | 18 |
| PK[f] | | | | | | | | | | 2 | | | | | | 2 |
| Total Volume by Visit (mL) | 20 | 12 | 10 | 10 | 14 | 10 | 20 | 6 | 12 | 12 | 10 | 14 | 14 | 6 | 12 | 182 |
| **Total Volume: 182 mL** | | | | | | | | | | | | | | | | |

F/U = Follow-up; SCR = Screening
[a] Hematology, Chemistry, Lipids; GLDH; volume includes blood for vitamin D testing, fasting insulin and glucose, cortisol, and exploratory PD biomarkers, where applicable.
[b] Includes blood for HbA1c testing.
[c] Subjects must have fasted ≥ 6 hours prior to blood draws.
[d] Osteocalcin, CTX1, P1NP, pre-dose on dosing days; subjects must have fasted ≥ 6 hours prior to pre-dose and Week 52 blood draws.
[e] ACTH Stimulation Test performed at Screening, Week 24 Follow-up Visit, 48 ± 3 hours after the final dose of Treatment Period #1 study medication and prior to first dose of study medication in the Transition Period, and Week 48 Follow-up Visit, 48 ± 3 hours after the final dose of Treatment Period #2 study medication and prior to first dose of study medication in the Dose-Tapering Period.
[f] Blood drawn for population PK at 2 hours post-dose at the Week 30 Visit.

### 7.2.10 12-Lead ECG

12-lead ECGs will be recorded at the Screening, Week 12, Week 24, Week 40, and Week 48 Visits. All ECG recordings must be performed using a standard high-quality, high-fidelity machine equipped with computer-based interval measurements. Digital ECG recording is recommended. Automated ECG intervals (QRS duration, PR [PQ] interval, RR interval [interbeat interval], QT interval, QTc, and heart rate) will be captured or calculated.

12-lead ECGs will be obtained over a 3- to 5-minute period after the subject has been resting quietly in a supine position for at least 5 minutes.

If blood sampling, vital signs assessment, and ECG recordings are scheduled at the same study visits, at least 15 minutes should elapse between collection of blood samples and before performing ECG and recording vital signs.

ECG results will be read locally. Results must be interpreted and recorded on the appropriate eCRF page.

### 7.2.11 2D-echocardiography

Standard trans-thoracic echocardiogram will be performed at Screening, Week 24, and Week 48 to assess cardiac status. The echocardiograms must be manually reviewed and interpreted locally by medically qualified personnel. No central reading of echocardiography results is planned. The findings will be categorized as: normal; abnormal but not clinically significant; abnormal and clinically significant. An echocardiogram result that is abnormal and clinically significant will be recorded in medical history if detected during the Screening Period and will be considered as an AE if detected after the first dose of study medication. Adequate management should be initiated if any abnormalities of clinical significance are detected.

Echocardiographic parameters to be recorded will be described in the Manual of Operations. Results must be interpreted and recorded on the appropriate eCRF page.

### *7.2.12 Eye Examination*

An eye examination will be performed by a certified and appropriately trained optometrist or ophthalmologist at Screening, Week 24, and Week 48 to assess for presence and degree of cataracts and glaucoma. Number and severity of cataracts, if present, will be recorded. Ocular pressure and presence/absence of glaucoma will be recorded.

Results must be interpreted and recorded in the source document and on the appropriate eCRF page.

### *7.2.13 Bone Health and DXA Scan (total body and spine)*

Data on bone mass, density, and total body composition (fat mass, fat-free mass, lean mass, Lean Mass Index, and Fat Mass Index) will be collected by DXA during the Screening Period (depending on local facilities, this may require an additional visit but should be completed prior to starting study medication), and at Week 24 and Week 48.

Antero-posterior spine (L1-L4) and total body (without head) bone mineral density (BMD) by DXA scan will be collected. DXA quality control will be performed as described in the Manual of Operations. DXA scans will be analyzed centrally by a certified medical radiation technologist at Children's Hospital of Ottawa in Ottawa, Canada, and then age-specific z-scores will be generated in order to chart the differences in the change in BMD z-scores among the different groups participating in this trial. The Screening result will represent a baseline assessment for long-term follow up. Additional DXA scanning may be arranged if clinically indicated throughout the study.

Vitamin D deficiency and insufficiency will be treated with Vitamin D supplements (see **Section 5.7.6**).

Vertebral and non-vertebral fractures will be assessed and recorded at Screening, Week 24, and Week 48 using a Fracture Questionnaire. Fractures will be recorded as medical history if reported during the Screening Period and as AEs if detected following the first dose of study medication and confirmed by radiologic investigation. The Fracture Questionnaires completed at the Weeks 24 and 48 Visits will document all

radiographically confirmed fractures which occurred during the course of the study, following the first dose of study medication.

### *7.2.14 Spine X-rays*

Data on bone health will also be collected by lateral spine X-ray (T4-L5) during the Screening Period (depending on local facilities, this may require an additional visit but should be completed prior to starting study medication) and at the Week 24 Visit. Lateral spine X-ray will be analyzed centrally by two certified pediatric radiologists at Children's Hospital of Ottawa in Ottawa, Canada, who are blinded to the results of one another; a third radiologist will resolve any discrepancies arising from the first two readings. Quantification of any vertebral fractures detected will be performed. The Screening result will represent a baseline assessment for long-term follow up. Additional spine X-rays may be arranged if clinically indicated throughout the study.

Fractures will be recorded as medical history if detected during the Screening Period and as AEs if detected following the first dose of study medication.

## 7.3 Assessment of Muscle Strength and Function

Muscle strength and function assessments should be performed in the morning and at approximately the same time of day, whenever possible.

### *7.3.1 Time to Stand Test (TTSTAND)*

The Time to Stand Test (TTSTAND) will be assessed at the Screening, Baseline Day -1, Week 6, Week 12, Week 24, Week 34, Week 40 and Week 48 Visits.

The TTSTAND measures the time (in seconds) required for the subject to stand to an erect position from a supine position (floor), and is assessed as part of the NSAA (see **Section 7.3.4**). Complete instructions for administering and scoring the TTSTAND are given in the Clinical Evaluator Manual to be supplied to the sites prior to study start.

Results will be recorded in the source documents and in the eCRF.

### 7.3.2 Time to Climb Test (TTCLIMB)

The Time to Climb Test (TTCLIMB) will be assessed at Screening, Baseline Day -1, Week 12, Week 24, Week 40, and Week 48 Visits.

The TTCLIMB measures the time (in seconds) required for the subject to climb 4 standard stairs, beginning and ending in a standing position with arms at the sides.[35] Complete instructions for administering the TTCLIMB are given in the Clinical Evaluator Manual to be supplied to the sites prior to study start.

Results will be recorded in the source documents and in the eCRF.

### 7.3.3 Time to Run/Walk Test (TTRW)

The Time to Run/Walk Test (TTRW) will be assessed at Screening, Baseline Day -1, Week 12, Week 24, Week 40, and Week 48 Visits.

The TTRW measures the time (in seconds) that it takes a subject to run or walk 10 meters and is assessed as part of the NSAA (see **Section 7.3.4**). Complete instructions for administering and scoring the TTRW are given in the Clinical Evaluator Manual to be supplied to sites prior to study start.

Results will be recorded in the source documents and in the eCRF.

### 7.3.4 North Star Ambulatory Assessment (NSAA)

The North Star Ambulatory Assessment (NSAA) is a clinical assessment scale specifically designed to measure functional ability in ambulant boys with DMD.[36] The NSAA consists of 17 scored items and 2 timed tests, including the TTRW and the TTSTAND (see **Section 7.3.1**). The NSAA will be conducted at Screening, Baseline Day -1, Week 12, Week 24, Week 40, and Week 48 Visits.

Subjects should be barefoot and wear comfortable clothing. Complete instructions for administering and scoring the NSAA are given in the Clinical Evaluator Manual to be supplied to the sites prior to study start.

The NSAA should be assessed BEFORE the 6MWT at study visits where both tests are performed.

Results will be recorded in the source documents and in the eCRF.

### *7.3.5 Six-minute Walk Test (6MWT)*

Functional exercise capacity and mobility will be assessed in all subjects by means of the Six-minute Walk Test (6MWT) at Screening, Baseline Day -1, Week 12, Week 24, Week 40, and Week 48 Visits. This evaluation is a modified version of the 6MWT, adapted for use in DMD patients.[37]

The total distance traveled, in meters, should be recorded along with the validity of the test as assessed by the test administrator in the source documents and in the eCRF. If a subject cannot complete 6 minutes of walking, the total meters and the time until discontinuation of the test should be recorded. Subjects should wear comfortable shoes (trainers) and clothing. Complete instructions for administering the 6MWT are given in the Clinical Evaluator Manual to be supplied to the sites prior to study start.

The 6MWT should be assessed AFTER the NSAA at study visits where both tests are performed.

Results will be recorded in the source documents and in the eCRF.

### *7.3.6 Hand-Held Myometry (elbow flexors and knee extensors)*

Muscle strength will be measured with hand-held myometry. Elbow flexor muscles will be measured in the upper limbs and quadriceps muscle will be used for the lower limbs. Measurements will be performed unilaterally on the elbow and knee muscles, on the same side as the dominant hand (see Clinical Evaluator Manual for details). Muscle strength will be measured at Screening, Baseline Day -1, Week 12, Week 24, Week 40, and Week 48 Visits.

Results will be recorded in the source documents and eCRF.

### *7.3.7 Range of Motion (ROM)*

Range of motion (ROM) at the ankle joint will be measured using a standard goniometer at the Screening, Baseline Day -1, Week 12, Week 24, Week 40, and Week 48 Visits. Measurements will be performed on both the right and left ankle joints.

Training will be given to study staff and specific detailed instructions are included in the Clinical Evaluator Manual.

Results will be recorded in the source documents and eCRF.

## 7.4 Patient-Reported Outcome Measures

### *7.4.1 Pediatric Outcomes Data Collection Instrument (PODCI)*

Physical functioning will be assessed by completion of the Pediatric Outcomes Data Collection Instrument (PODCI). The subject parent/legal guardians will be asked to complete this instrument at the Screening, Week 24, and Week 48 Visits.

The completed Instrument is considered the source documentation for this assessment. Results will be recorded in the eCRF.

### *7.4.2 Treatment Satisfaction Questionnaire (TSQM)*

Satisfaction with treatment will be measured at the Week 24 and Week 48 Visits using the Treatment Satisfaction Questionnaire for Medication (TSQM). The TSQM consists of 14 Likert-scale items that yield four subscale scores: Effectiveness, Side Effects, Convenience, and Global Satisfaction (the latter being a component of the primary outcome variable for the proposed trial). A child-report version of the TSQM is not available. Therefore, the parent (s)/guardian(s) will be asked to report from their perspective of the boy’s treatment. TSQM is available in all primary languages spoken at sites for this study.

The completed Questionnaire is considered the source documentation for this assessment. Results will be recorded in the eCRF.

### *7.4.3 Behavioral Assessment*

One instrument, for completion by the parent(s)/guardian(s), will be used for behavior assessment screening and evaluation of behavior change. This is the PARS III, a scale designed to measure psychosocial adjustment of children with chronic physical illnesses. The instrument will be completed by the parent(s)/guardian(s) at the Screening Visit and

at the Weeks 12, 24, and 48 Visits. The PARS III is available in all primary languages spoken at sites for this study.

The completed assessment is considered the source documentation. Results will be recorded in the eCRF.

#### *7.4.4 Ease of Study Medication Administration Assessment*

Ease of administration of the suspension study medication will be assessed by the parent(s)/guardian(s) at the Weeks 2, 12, 24, 30, 40, and 48 Visits. Results will be recorded in the source documents and eCRF.

#### *7.4.5 Blindedness Assessment*

The subject's parent(s)/guardian(s), the site Principal Investigator, and the Clinical Evaluator will each complete a Blindedness Assessment at the Week 24 Visit. This is a brief questionnaire which asks each evaluator to predict the identity of the study medication (vamorolone, prednisone, or placebo) the subject was taking during Treatment Period #1, and to rate on a 4-point scale his/her level of certainty and the reason for the chosen level of certainty.

Results will be recorded in the source documents and in the eCRF.

#### *7.4.6 Subject Diary*

The parent or legal guardian of each subject will be given a subject diary at the Day 1 Visit in which to record any new concomitant medications and any changes to existing concomitant medications taken during the study, any AEs experienced by the subject during the study, and any missed or incomplete doses of study medication. Parents/legal guardians will be instructed in how to record information in the diary and will be instructed to bring the diary with them to each study visit for review by study staff for completeness and accuracy. A new diary will be dispensed at each visit for use through the time of the next scheduled visit. Collection of final diaries will occur at the Week 52 Visit, at the end of the Dose-tapering Period.

## 7.5 Adverse Events and Serious Adverse Events

The condition of the subjects will be monitored throughout the duration of the study by the clinical site study team and by recording of AEs in subject diaries. An AE is any untoward medical occurrence in a subject which does not necessarily have to have a causal relationship with the intervention. An AE can therefore be any unfavorable and unintended sign (including an abnormal laboratory finding, for example), symptom, or disease temporally associated with the use of an investigational product, whether or not considered related to the drug. Pre-existing conditions that worsen during a study are to be reported as AEs. Signs and symptoms of DMD should not be recorded as AEs, unless their nature or severity is unexpected for the course of the disease.

The Investigator is responsible for reporting AEs and SAEs to the Sponsor or designee. For reported death of a participant, the Investigator shall supply the Sponsor and the IRB/IEC with any additional information requested.

Adverse events will be recorded from the date of informed consent and through the time of the subject's last study visit (study completion or early discontinuation). Serious adverse events will be recorded from the date of informed consent, throughout the clinical trial, and for up to 30 days after the final administration of study drug. In addition, subjects (and their parent or legal guardian) will be questioned by study staff at each study visit for any new signs or symptoms or changes in existing signs or symptoms.

All AEs and SAEs that are spontaneously reported, identified during questioning, or are apparent from a participant's physical appearance, will be recorded in the source documents and in the subject's eCRF. The date of onset will be recorded. Any laboratory abnormality that is outside the normal range and is considered an AE (see **Section 7.2.4**) should be recorded as an AE on the appropriate eCRF page. The details recorded shall include the nature, date of onset, final outcome and its date, intensity assessment (Common Terminology Criteria for Adverse Events [CTCAE] grade), and a determination of the relationship of the event to administration of the study drug (i.e., causality). All AEs will be graded by CTCAE, Version 4.03. Details of any medications given to the subject to abate the AE should be recorded on the appropriate eCRF page.

### 7.5.1 Intensity

All clinical AEs encountered during the clinical study will be recorded in the eCRF. Intensity of AEs will be graded using the most current version of the CTCAE, version 4.03, 5-point scale, and reported in detail as indicated in the eCRF. A description of the intensity scales can be found below:

Mild (Grade 1): Asymptomatic or mild symptoms: clinical or diagnostic observations only; intervention not indicated.

Moderate (Grade 2): Minimal, local, or noninvasive intervention indicated; limiting age-appropriate instrumental activities of daily living (ADL).

Severe (Grade 3): Severe or medically significant but not immediately life-threatening: hospitalization or prolongation of hospitalization indicated; disabling; limiting self-care ADL; incapacitating with inability to work or perform normal daily activity.

Life-Threatening (Grade 4): Urgent intervention indicated.

Death (Grade 5): Death related to AE.

### 7.5.2 Relationship

Relationship to study drug will be graded on a 5-point scale (definite, probable, possible, remote, or unrelated). A description of the relationship scale can be found below:

Definite: This category applies to an AE that meets at least criteria 1, 2, and 4 of the "Probable" category.

Probable: This category applies to those AEs that are considered, with a high degree of certainty, to be related to the study drug. An AE may be considered probable, if (must include first 3):

1. It follows a reasonable temporal sequence from administration of the study drug.

2. It cannot be reasonably explained by the known characteristics of the subject's clinical state, environmental or toxic factors, or other modes of therapy administered to the subject.
3. It disappears or decreases after dosing is complete. (There are important exceptions when an AE does not disappear upon discontinuation of study drug, yet drug relatedness clearly exists, e.g., [1] bone marrow depression and [2] tardive dyskinesia.)
4. It follows a known pattern of response to the suspected study drug.

Possible: This category applies to those AEs for which the connection with study drug administration appears unlikely but cannot be ruled out with certainty. An AE may be considered possibly related to study drug if or when (must include first 2):

1. It follows a reasonable temporal sequence from administration of the study drug.
2. It may have been produced by the subject's clinical state, environmental or toxic factors, or other modes of therapy administered to the subject.
3. It follows a known pattern of response to the suspected study drug.

Remote: In general, this category is applicable to an AE that meets the following criteria (must include the first 2):

1. It does not follow a reasonable temporal sequence from administration of the study drug.
2. It may readily have been produced by the subject's clinical state, environmental or toxic factors, or other modes of therapy administered to the subject.
3. It does not follow a known pattern of response to the suspected study drug.

Unrelated: This category is applicable to those AEs which are judged to be clearly and incontrovertibly due only to extraneous causes (disease, environment, etc.) and

do not meet the criteria for study drug relationship listed under remote, possible, or probable.

### *7.5.3 Clinical Laboratory Test Abnormalities*

Clinical laboratory test results will be recorded on the designated eCRF page. The intensity of abnormal clinical laboratory test results that are AEs will also be graded using the most current version of the CTCAE, version 4.03, 5-point scale and reported in detail as indicated in the eCRF. A description of the intensity scale can be found above.

Any treatment-emergent abnormal clinical laboratory test result that is clinically significant, i.e., meeting one or more of the following conditions, should be recorded as a single diagnosis on the AE section of the eCRF:

- Accompanied by clinical symptoms
- Requiring a change in concomitant therapy (e.g., addition of, interruption of, discontinuation of, or any other change in a concomitant medication, therapy, or treatment)
- Is otherwise considered clinically significant by the Investigator

**This applies to** any protocol and non-protocol-specified safety laboratory result from tests performed after the first dose of study drug, which falls outside the laboratory reference range and meets the clinical significance criteria per Investigator standard operating procedures (SOPs).

**This does not apply to** any abnormal laboratory result that falls outside the laboratory reference range, but does not meet the clinical significance criteria (which will be analyzed and reported as laboratory abnormalities); those that are considered AEs of the type explicitly exempted by the protocol; or those that are the result of an AE which has already been reported.

**Please Note**: any clinical laboratory abnormality fulfilling the criteria for an SAE should be reported as such, in addition to being recorded as an AE in the eCRF.

### 7.5.4 Follow-Up of Adverse Events

Adverse events will be followed until they have returned to baseline status, stabilized, or the Investigator, Study Chair, Medical Monitor and Sponsor agree that follow-up is no longer needed. If a clear explanation of cause is established, it should be recorded in the source documents and eCRF. In the event of unexplained abnormal laboratory test values, the tests may be repeated as soon as possible and followed up until they have returned to the normal range or baseline value and/or an adequate explanation of the abnormality is found. In case of ongoing AEs at the time of database closure, the data obtained at the time of database closure will be used in the statistical analysis. The further follow-up of AEs will be documented in the source documents and will be described in the final report only if considered relevant by the Investigator, the Study Chair, the Medical Monitor and/or the Sponsor.

In addition, the Medical Monitor may request additional blood tests, diagnostic imaging studies, or specialist physician consultations in order to further evaluate any AE or test abnormality considered to be clinically significant by the Study Sponsor.

### 7.5.5 Dosing Error

For the purposes of this study, a dosing error is defined as a dose exceeding or less than the scheduled dose of liquid formulation, tablet formulation, or both. Such occurrences will be reported and recorded in the dosing page of the eCRF and as follows:

- Use of study medication in doses in excess of that specified in the protocol will not be recorded as an AE unless there are associated signs or symptoms.
- A dosing error with associated non-serious AEs will be recorded as AEs on the relevant AE forms in the eCRF.
- A dosing error with an associated SAE will be recorded as an SAE.
- Details of all dosing errors, including actual dose administered, will be documented in the source documents and recorded in the appropriate documentation.

### 7.5.6 *Serious Adverse Events*

Serious adverse events will be collected and reported during the study from the time informed consent is obtained through 30 days after the final dose of study medication, according to the protocol and applicable regulations. For subjects who do not continue into an additional vamorolone study or general access program, site staff will make a phone call to the home 31-35 days after the final dose of study medication in the VBP15-004 Dose-tapering Period to confirm the final SAE status of the subject.

All SAEs, including those that continue beyond the normal AE collection period (i.e., are ongoing at the subject's last study visit), will be followed until resolution or until stabilized without sequelae. All SAEs, both related and unrelated, that begin within 30 days after the subject's final dose of study medication will be reported to the Sponsor within 24 hours of discovery by the Investigator.

During the SAE collection period, the Investigator or clinical site personnel should notify the Coordinating Center of all SAEs, regardless of relationship to the investigational drug, within 24 hours of clinical staff becoming aware of the event; notification to the Coordinating Center will trigger alerts to the Study Chair, the Sponsor, and the Medical Monitor. The Investigator will provide the initial notification by completing the SAE Report Form in the electronic data capture (EDC) system, which must include the Investigator's assessment of the relationship of the event to investigational drug, and must be signed by the Investigator.

In addition, notification is sent by the Investigator to the IRB/IEC and the subject's Primary Care Physician.

Follow-up information, or new information regarding an ongoing SAE, must be provided promptly to the Coordinating Center within 24 hours of knowledge of the new or follow-up information, which will forward the information to the Study Chair, the Sponsor, and the Medical Monitor.

All SAE reports should be completed within the EDC.

An AE or suspected adverse reaction is considered serious if, in the view of either the Investigator or Sponsor, it results in any of the following outcomes:

- Is fatal (results in the outcome of death)
- Is life-threatening
- Requires in-patient hospitalization or prolongation of existing hospitalization
- Results in persistent or significant incapacity or substantial disruption of the ability to conduct normal life functions
- Is a congenital anomaly or birth defect
- Is an important medical event that may jeopardize the subject and may require medical or surgical intervention to prevent one of the outcomes listed above.

The terms death and sudden death are clearly distinct and must not be used interchangeably.

Any AE or clinically significant abnormal laboratory test value, as determined by the Investigator, that is serious and which occurs during the course of the study (as defined above) must be reported to the Coordinating Center, who will notify the Study Chair, the Sponsor and the Medical Monitor within 24 hours of the Investigator becoming aware of the event. Additional information that becomes available for an SAE after the initial report is submitted will be reported to the Coordinating Center, who will notify the Study Chair, the Sponsor and the Medical Monitor within 24 hours of the Investigator becoming aware of the new information.

All SAEs must be collected and reported during the study from the time of informed consent through 30 days after the final dose of study medication. All SAEs, related and unrelated, must be reported to the Sponsor within 24 hours of first awareness.

If, at any time during the study, a subject experiences an SAE, appropriate care should be instituted.

**In the event of a serious adverse event (SAE), the Investigator will complete the SAE electronic case report form within 24 hours of first awareness of the event. In the**

**unlikely event that the electronic study database is inaccessible and the Investigator is unable to complete the SAE electronic case report form within 24 hours, the SAE Notification Form (pdf) should be completed and emailed or printed/faxed to the PRA safety management team within 24 hours, using the contact information below:**

**In United States and Canada:**

**Email: CHOSafety@prahs.com**
**Drug Safety Fax: 1 888 772 6919 or 1 434 951 3482**
**SAE Questions: Drug Safety Helpline: 1 800 772 2215**

**In Europe, Asia, Pacific, Africa and Australia:**

**Email: MHGSafety@prahs.com**
**Drug Safety Fax: +44 1792 525720**
**SAE Questions: Drug Safety Helpline: +49 621 878 2154**

Serious Adverse Events will be recorded from the time the subject's written informed consent is obtained. Serious adverse events that occur within 30 days of study drug dosing must continue to be recorded and reported to the Study Sponsor or its designee. Should there be an SAE that occurs that suggests an increased risk to the participants, the following steps will be considered, depending on the number and severity of the SAE(s): modification of the protocol, investigation of the relationship of the SAE(s) to study drug, suspension of the study, and/or discontinuation of the study.

Suspected Unexpected Serious Adverse Reaction (SUSAR) Identification and Reporting

A Suspected Unexpected Serious Adverse Reaction (SUSAR) is a suspected adverse reaction that is both serious and unexpected (not identified in the Investigator's Brochure[29]). Sponsor will inform Investigators of SUSARs in a manner and timeframe consistent with applicable national regulatory requirements.

The study will comply with all local regulatory requirements. This study adheres to the definition and reporting requirements of ICH Guideline for Clinical Safety Data Management, Definitions and Standards for Expedited Reporting.

# 8 STUDY COMMITTEES

## 8.1 Study Steering Committee

A study steering committee (SSC – VISION-DMD) will be responsible for protocol development, review of any study amendments, and coordination of study conduct and interpretation of study results. The SSC comprises the Sponsor, study chairs and medical monitors for the VBP15-003, VBP15-LTE, VBP15-004, and other studies, as applicable, the project managers, and the patient representatives.

## 8.2 Data and Safety Monitoring Board

An unblinded Data and Safety Monitoring Board (DSMB), operating autonomously from the SSC and the site investigators, will be responsible for providing independent recommendations to the SSC about risk-benefit of the study and for any modification affecting safety or data integrity required during the course of the study. The DSMB members must not be actively involved in study design, conduct or daily management of this study and must not have financial, propriety, professional, or other interests that may affect impartial, independent decision-making.

Specialists may be invited to participate as non-voting members at any time if additional expertise is desired. The DSMB will formally interact with the SSC through the sharing of blinded DSMB meeting minutes.

The DSMB will be responsible for:

- Examining accumulating safety and other relevant data at pre-specified points during the course of the study in order to make recommendations concerning continuation, termination, or modification of the study;
- Reviewing important protocol deviations;
- Providing expert advice to the SSC on an ad hoc basis regarding matters such as safety concerns or diagnostic evaluations in individual subjects;

- Based on the results of its deliberations, the DSMB can recommend continuation of the studies unchanged, study interruption, study termination, modification of the studies, or alteration in the DSMB monitoring plan.

# 9 DATA COLLECTION

## 9.1 Source Documents

Source documents are defined as original documents, data, and records. These documents may include hospital records, clinical and office charts, laboratory data/information, subjects' diaries or evaluation checklists, pharmacy dispensing and other records, recorded data from automated instruments, microfilm or magnetic media, and/or x-rays. Data collected during this study must be recorded in the appropriate source documents.

Investigators will keep a record relating the names of the subjects to their enrollment numbers (subject identification log) to permit efficient verification of data subject files, when required.

A subject enrollment log is to be completed at each study site. Data recorded on the enrollment log are to include a subject identifier, the dates of enrollment and completion/termination, and the reason the subject was not entered (if applicable).

The Investigator(s)/institution(s) will permit study-related monitoring, audits, IRB/IEC review, and regulatory inspection(s), providing direct access to source data documents.

## 9.2 Electronic Case Report Form Completion

Subject data will be collected in this study using an EDC system. The EDC and database system will be OpenClinica by Akaza Research, LLC. OpenClinica is a web-based (https://www.openclinica.com) data entry system utilizing a high security environment. The underlying storage facility will be PostgreSQL, whose structure permits the linking of subject information across all tables in relational databases. OpenClinica uses secure socket layers (SSL) and in its Enterprise version used in this study is 21 CFR Part 11 compliant. Once an eCRF is created in the database, a data dictionary exists and the data team creates compatible paper source documentation.

The Coordinating Center will design an electronic database in OpenClinica for this study. Access rights to the EDC system for the study site team members will need to be requested. Every user of the system will be made aware of the fact that user name and password should never be shared and their electronic signature constitutes the legally binding equivalent of a handwritten signature. Only trained personnel certified by the Coordinating Center will receive a user name and password.

All data will be directly entered or collected on a source document and then entered into OpenClinica or transferred electronically to the study database (e.g., clinical laboratory results).

The Coordinating Center data management team will monitor the eCRFs for completeness and acceptability throughout the course of the study. ReveraGen personnel (or their representatives) will be allowed read-only access to all source documents in order to verify eCRF entries.

## 9.3 Data Processing

A clinical study database will be constructed from the eCRFs and any data merged electronically, and the data will be validated both manually and electronically. Clarification of data will be requested from the study site as required. The database will be quality assured in accordance to the data management plan and will be available for statistical analysis according to the methods outlined in **Section 10.9** and the Statistical Analysis Plan (SAP).

## 9.4 Subject Diaries

The information recorded in the diary will be considered source documentation, and any relevant requested information recorded in the diary should be transcribed by study staff to the appropriate eCRF page.

# 10 STATISTICAL METHODS AND PLANNED ANALYSES

## 10.1 Sample Size Determination

This is a randomized, double-blind, parallel group, placebo- and active-controlled study. Study medication is administered daily in this Phase IIb trial. Data for untreated subjects

from the Cooperative International Neuromuscular Research Group (CINRG) Duchenne Natural History Study[38,39,40,41] and data for prednisone treated subjects from the CINRG Prednisone study[42] were used to help estimate sample sizes for this study.

The primary efficacy outcome is TTSTAND (velocity) change from baseline to Week 24. A sample size of 30 subjects per treatment group will detect a 0.0674 point difference in mean change from baseline to Week 24 in TTSTAND (velocity) between a vamorolone dose level and placebo, assuming a common standard deviation of 0.08, a two-sided t test, and a Type-I error of 0.025 with approximately 83% power. The Bonferroni adjustment method will be used to control the Type-I error rate at 0.05 due to the multiple comparisons (2 vamorolone dose levels will be tested against placebo).

Based on this calculation, a total of approximately 120 subjects will be randomized to treatment with vamorolone 2.0 mg/kg/day (n=30), vamorolone 6.0 mg/kg/day (n=30), prednisone 0.75 mg/kg/day (n=30), or placebo (n=30).

Note that subjects in the prednisone and placebo groups will actually be randomized into two groups each:

- Prednisone 0.75 mg/kg/day → Vamorolone 2.0 mg/kg/day (n=15);
- Prednisone 0.75 mg/kg/day → Vamorolone 6.0 mg/kg/day (n=15);
- Placebo → Vamorolone 2.0 mg/kg/day (n=15); or
- Placebo → Vamorolone 6.0 mg/kg/day (n=15).

These groups will be pooled by initial treatment (prednisone or placebo) for the Treatment Period #1 analyses.

If the number of subjects who withdraw early from the study is high, additional subjects may be enrolled to achieve approximately 120 subjects completing the Week 24 Visit assessments.

## 10.2 Statistical and Analytical Plan (SAP)

The sections below summarize the intended statistical methods and analyses for this study. A more detailed SAP will be written and finalized prior to any lock of the study database (final or interim, if applicable) and any analysis performed. The SAP will give

a detailed description of the summaries and analyses that will be performed and will clearly describe when these analyses will take place.

### *10.2.1 Deviations from the Statistical Analysis Plan*

Any deviation(s) from the original SAP will be described and justified in the clinical study report.

## 10.3 Analysis Populations

Three populations will be defined for data analysis: the Safety Population, the modified Intent-to-Treat Population, and the Pharmacokinetic Population.

### *10.3.1 Safety Population*

All subjects who receive at least one dose of study medication will be included in the Safety Population. The Safety Population is the primary analysis population for safety and PD assessments. Results will be presented “as treated.”

### *10.3.2 Modified Intent-to-Treat (mITT) Population*

All subjects who receive at least one dose of study medication and have at least one post-baseline assessment will be included in the mITT Population. The mITT Population is the primary analysis population for clinical efficacy. Subjects who receive at least one dose but never have post-baseline assessments will be excluded. Results will be presented “as randomized.”

### *10.3.3 Pharmacokinetic (PK) Population*

All subjects who receive at least one dose of vamorolone study medication and have sufficient data for PK analysis will be included in the PK Population.

## 10.4 Measures Taken to Avoid/Minimize Bias

Not applicable.

## 10.5 Interim Analysis

No formal interim statistical analyses are planned; however, interim safety data and overviews will be supplied to the DSMB. Since this is a blinded study, an unblinded and

independent statistician will be involved in preparing the overviews for the DSMB. These overviews will not be shared outside the unblinded and independent statistician and the unblinded DSMB members.

## 10.6 Week 24 Analysis

The primary analyses for this study are the analyses which will be performed after all subjects complete Week 24 of Treatment Period #1. The results from these analyses will be provided to regulatory authorities. Investigators, study subjects, study staff, and monitors will remain blinded throughout the duration of the 52-week study. The Sponsor, including data management and statistical personnel, will be unblinded after the Week 24 analyses.

## 10.7 Week 48 Analysis

The Week 48 analyses will be performed after all subjects complete Week 48 of Treatment Period #2. The results from these analyses will be provided to regulatory authorities. All study staff may be unblinded after database lock of Treatment Period #2 data.

## 10.8 Missing, Unused, and Spurious Data

The primary analyses for the primary and secondary efficacy variables will be conducted on observed data only; the MMRMs will employ appropriate covariance structures to accommodate missing data, as described in **Section 10.9.4**. Missing efficacy data will be imputed for supportive sensitivity analyses on the primary and secondary efficacy endpoints using the following methods:

- Multiple Imputations using Markov Chain Monte Carlo (MCMC); and
- Multiple Imputations using a Control-based Pattern Mixture Model.

Full details will be provided in the SAP.

## 10.9 Statistical Analysis

### *10.9.1 General Considerations*

Statistical analyses will be performed using SAS® version 9.4 or later.

All measurements will be analyzed based upon the type of distribution, and descriptive statistics will be presented by treatment group and assessment time point, as appropriate. Descriptive statistics for continuous variables (number [N], mean, median, standard deviation [SD], minimum, and maximum), descriptive statistics for categorical variables (N and percentage), and individual subject profiles will be presented, as appropriate.

No formal interim statistical analyses are planned, apart from the interim data views and presentations to be created for the DSMB. Missing values for safety and exploratory outcomes will be treated as missing, unless stated otherwise.

Baseline measurement is defined as the last non-missing value prior to the first dose of study drug in the study.

Treatment Period #1 analyses will be summarized by four treatment groups:

- Vamorolone 2.0 mg/kg/day (n=30);
- Vamorolone 6.0 mg/kg/day (n=30);
- Prednisone 0.75 mg/kg/day (n=30); and
- Placebo (n=30).

Treatment Period #2 analyses (besides historical control comparison data) will be summarized by six treatment groups:

- Vamorolone 2.0 mg/kg/day → Vamorolone 2.0 mg/kg/day (n=30);
- Vamorolone 6.0 mg/kg/day → Vamorolone 6.0 mg/kg/day (n=30);
- Prednisone 0.75 mg/kg/day → Vamorolone 2.0 mg/kg/day (n=15);
- Prednisone 0.75 mg/kg/day → Vamorolone 6.0 mg/kg/day (n=15);
- Placebo → Vamorolone 2.0 mg/kg/day (n=15); and
- Placebo → Vamorolone 6.0 mg/kg/day (n=15).

TTSTAND, TTCLIMB, and TTRW will be analyzed using raw scores and velocity. Velocity will be calculated as follows:

- TTSTAND velocity = 1 / TTSTAND and is expressed as rises/sec
- TTCLIMB velocity = 1 / TTCLIMB and is expressed as tasks/sec
- TTRW velocity = 10 / TTRW and is expressed as meters/sec

Sensitivity analyses will be performed and will be described in the SAP.

### *10.9.2 Adjustment for Multiple Comparisons*

The primary efficacy endpoint tests each dose of vamorolone vs. placebo using TTSTAND velocity at Week 24 and occurs during Treatment Period #1 of the study. The primary efficacy endpoint supports the primary objective of this study. The study is thus powered for the efficacy comparisons.

A multi-branched gatekeeping procedure will be utilized for the primary efficacy endpoint. The primary efficacy endpoint (TTSTAND velocity at Week 24) will be tested first using a Bonferroni adjustment. Any dose that is significant for the primary efficacy endpoint will then have the secondary efficacy endpoints tested sequentially. Further details are provided below.

For primary efficacy (TTSTAND velocity), the two vamorolone dose levels will be compared with placebo. To account for these comparisons (two vamorolone dose levels vs. placebo), Bonferroni multiple comparison adjustments will be utilized. Each comparison will be conducted at the 0.025 (0.05/2) alpha level.

Secondary efficacy endpoints will be tested sequentially on change from baseline to Week 24 values (each dose of vamorolone vs. placebo or prednisone [for 6MWT only]). Only the doses that are significant for the primary efficacy endpoint (change in TTSTAND velocity at Week 24) will have the secondary endpoints tested. A 0.025 alpha level will be used for the sequential testing. Testing will stop once a p-value is >0.025 for one of the secondary efficacy endpoints. The Week 24 change from baseline values will be tested using this sequential testing procedure. The order of the secondary efficacy endpoints is as follows.

1. Six-minute Walk Test (6MWT) vs. placebo
2. Time to Run/Walk 10 meters Test (TTRW) velocity vs placebo
3. 6MWT vs. prednisone
4. North Star Ambulatory Assessment (NSAA) vs. placebo
5. Hand-held Myometry (knee extensors) vs. placebo

6. Hand-held Myometry (elbow flexors) vs. placebo
7. Time to Climb 4 Steps (TTCLIMB) velocity vs. placebo
8. Range of Motion (ROM) in the ankles vs. placebo

All other analyses will not be corrected for multiple comparisons (tests will be performed at the 0.05 alpha level), as they will be viewed and handled in the perspective of not testing a formal hypothesis.

### *10.9.3 Subject Disposition, Demographics, and Baseline Characteristics*

Subject disposition will be summarized by analysis population. The number of subjects enrolled, the number in each population, and the reason for discontinuation from the study will be summarized and listed.

Subject demographics (e.g., age, race, and ethnicity) and baseline characteristics (e.g., height, weight, and months/years since DMD diagnosis) will be summarized descriptively by treatment group and overall, per analysis population. In addition, tables will be presented according to age stratification. Baseline characteristics between groups presented in these summary tables will be reviewed for any clinically relevant differences among the treatment groups or age stratification groups, and may be accounted for in the statistical models for the endpoints.

### *10.9.4 Efficacy Analyses*

The evaluations of clinical efficacy will be performed using the mITT Population. Analyses will be done as per randomized treatment.

All efficacy will be summarized and listed. Where considered relevant, plots will be created.

The primary efficacy outcome is the Time to Stand (TTSTAND) from supine (velocity). Secondary efficacy outcomes are the NSAA assessment, Time to Climb four stairs (TTCLIMB), Time to Run/Walk 10 meters (TTRW), the distance walked in 6 minutes (6MWT), hand-held myometry (elbow flexors and knee extensors), and ROM. TTSTAND, TTCLIMB, and TTRW will be analyzed using raw scores and velocity.

The primary efficacy outcome TTSTAND (velocity) change from baseline to Week 24 will be compared between each of the two different vamorolone dose groups and the placebo group using a restricted maximum likelihood (REML)-based mixed model for repeated measures (MMRM). This model includes fixed effects for treatment (vamorolone 2.0 mg/kg/day, vamorolone 6.0 mg/kg/day, prednisone 0.75 mg/kg/day, and placebo), week, baseline TTSTAND, age group (per stratification), and the treatment-by-week interaction. Study week will be included in the model as a categorical variable (Weeks 6, 12, and 24) along with the treatment-by-week interaction. Within this model, pairwise comparisons (using least squares [LS] mean contrasts) will be made to compare TTSTAND at 24 weeks for each vamorolone dose level with placebo separately (primary efficacy outcome), for each vamorolone dose level with prednisone separately (secondary analysis), and for the high vamorolone dose level with the low vamorolone dose level (secondary analysis). Treatments will also be compared at other weeks as secondary analyses. An unstructured covariance matrix will be used, and underlying modelling assumptions will be checked. If differences between baseline characteristics exist between the three treatment groups in this comparison, it will be investigated if adjustment for these characteristics is clinically relevant and necessary. The secondary outcome measures will be compared using similar models. Full details will be provided in the SAP.

The following sensitivity or supportive analyses will be performed (see SAP for full details) for the primary efficacy and secondary efficacy endpoints:

- Multiple imputations using MCMC methods for missing data;
- Multiple imputations using a Control-based Pattern-Mixture Model for missing data.

Subjects who are randomized to receive vamorolone 2.0 mg/kg/day or vamorolone 6.0 mg/kg/day for both treatment periods will have TTSTAND velocity change from baseline data captured over 48 weeks compared with untreated DMD historical control data. Full details will be provided in the SAP.

### 10.9.5 Safety Analyses

The Safety Population will be used for presentations and analyses of the safety parameters. Analyses will be done as per actual treatment received.

In general, descriptive statistics for each safety endpoint will be presented by time point and treatment group. In addition, individual subject listings of all safety data will be created and sorted by treatment group and time point, and will be reviewed for any evidence of dose-related differences or trends in the safety profile of vamorolone. Where considered relevant, plots will be created.

Safety data will include BMI, weight, vital signs, 2D-echocardiogram, DXA scan, spine X-ray, eye examination results, and ACTH Stimulation Test results, and ECG results, and these will be presented using descriptive statistics. Safety laboratory data will be summarized using descriptive statistics, and out-of-range values will be listed. For safety analyses, the vamorolone doses will be compared to prednisone.

In Treatment Period #1, BMI z-score change from baseline data captured over 24 weeks will be compared for subjects who are randomized to receive vamorolone 2.0 mg/kg/day or vamorolone 6.0 mg/kg/day vs. subjects randomized to receive prednisone. The same MMRM that is used for the primary efficacy analysis will be used for BMI z-score. The test for statistical significance will be performed at the 0.05 level. Subjects who are randomized to receive vamorolone 2.0 mg/kg/day or vamorolone 6.0 mg/kg/day for both treatment periods will have their BMI z-score change from baseline data captured over 48 weeks compared with prednisone-treated DMD historical control data. Full details will be provided in the SAP.

Adverse events will be coded using the Medical Dictionary for Regulatory Activities (MedDRA), version 20. The incidence of AEs will be summarized overall and by treatment group, SOC and preferred term; treatment group, SOC, preferred term, and intensity (CTCAE v 4.03 grade); and treatment group, SOC, preferred term, and relationship to study drug. Additional AE analyses will be at the subject level: the number of subjects who had any AE, the distribution of number of AEs per subject within

a treatment group, worst intensity in a subject within a treatment group, highest level of relationship to study treatment for each subject within a treatment group.

Physical examination results will be listed only.

### *10.9.6 Pharmacodynamic Analyses*

The evaluations of PD biomarkers will be performed using the Safety Population. Analyses will be done as per actually received treatment.

All PD biomarker results will be summarized and listed.

Serum PD biomarkers of adrenal axis suppression, insulin resistance, bone turnover, and immune suppression will be assessed. PD biomarkers will be analyzed using MMRMs similar to the primary efficacy model. Plots will be created. Additional exploratory PD biomarkers of both safety and efficacy may be assessed. Vamorolone-treated groups will be compared to both prednisone-treated and placebo groups.

### *10.9.7 Patient-Reported Outcome Exploratory Analyses*

Patient Reported Outcomes including the TSQM, PODCI, PARS III, Ease of Study Medication Administration Assessment, and the Blindedness Assessment will be listed and presented using descriptive statistics by treatment and time point. In addition, comparison will be made for each vamorolone dose group with placebo for the PODCI and for each vamorolone dose group with prednisone for the PARS III, with p values computed for the comparisons. Full details will be provided in the SAP.

### *10.9.8 Pharmacokinetic Analyses*

The 2-hr post-dose plasma concentration measurements of vamorolone at Week 30 will be used for comparison of drug exposures between the two dosing groups. They will be added to PK data from previous studies in DMD boys for comparison with measurements obtained in healthy adult male subjects. All PK data will be combined in a population assessment of plasma concentrations in relation to dose and age of subjects. The PK population will be used for these analyses. A separate PK Analysis Plan will be created to further discuss these analyses.

### *10.9.9 Concurrent Medications*

A summary of all concomitant medications taken during the course of the study will be presented in tabular form by therapeutic drug class and generic drug name using the World Health Organization (WHO) Drug classification (Version 4.3). All concomitant medications will be detailed in the subject data listings.

# 11 STUDY MANAGEMENT AND ETHICAL AND REGULATORY REQUIREMENTS

## 11.1 Regulatory Approval and Good Clinical Practice

This study will be conducted in accordance with the principles of the 18$^{th}$ World Medical Assembly (Helsinki, June 1964), and amendments of the 29$^{th}$ (Tokyo, 1975), 35$^{th}$ (Venice, 1983), 41$^{st}$ (Hong Kong, 1989), 48$^{th}$ (Somerset West, 1996), 52$^{nd}$ (Edinburgh, 2000), 53$^{rd}$ (Washington, 2002), 55$^{th}$ (Tokyo, 2004), 59$^{th}$ (Seoul, 2008), and 64$^{th}$ (Fortaleza, 2013) World Medical Assemblies and ICH E6 Guideline for Good Clinical Practice (GCP).

Further, the trial will be conducted in accordance with all applicable laws, guidances and directives of the jurisdiction where the study is being conducted

## 11.2 Investigator Responsibilities

### *11.2.1 Subject Information and Informed Consent*

It is the Investigator’s responsibility to ensure that parent(s)/guardian(s) give(s) informed consent before the subject is admitted to the study, in accordance with ICH guidelines on GCP and all applicable laws, guidances and directives of the jurisdiction where the study is being conducted.

If applicable, written or verbal assent will also be obtained from each subject as required per regulations.

An approved ICF will be given to each parent/guardian written in a language they understand.

The Investigator or designee will review the study with the parent(s)/guardian(s) of each subject. The review will include the nature, scope, procedures, and possible consequences of the subject's participation in the study. The consent, assent, and review must be in a form understandable to the parent(s)/guardian(s) of the subject. The Investigator or designee and the parent(s)/guardian(s) of the subject must both sign and date the ICF after review and before the subject can participate in the study. The parent(s)/guardian(s) of the subject will receive a copy of the signed and dated form, and the original will be retained in the site study files. The Investigator or designee must emphasize to the parent(s)/guardian(s) of the subject that study participation is entirely voluntary and that consent regarding study participation may be withdrawn at any time without penalty or loss of benefits to which the subject is otherwise entitled.

If the ICF is amended during the study, the Investigator must follow all applicable regulatory requirements pertaining to all new subjects and repeat the consent process with the amended ICF for any ongoing subjects.

### *11.2.2 Institutional Review Board/Independent Ethics Committee Approval and Other Institutional Requirements*

Before the start of the study, the study protocol, ICF, and any other appropriate documents will be submitted to the IRB/IEC for review and approval. Per institutional requirements, the study protocol and any other appropriate documents will be submitted to relevant committees for approval.

The Investigator will forward to the Sponsor, or designee (Coordinating Center), a copy of the IRB/IEC's approval of this protocol, amendments, ICF and any changes to the ICF, as per ICH guidelines on GCP and all applicable laws, guidances and directives of the jurisdiction where the study is being conducted. The Investigator will also keep documentation of study approval by internal committees per institutional requirements.

It is the responsibility of the Sponsor to notify the competent authority of the Member State concerned and/or the IEC of any substantial amendment(s) to the protocol.

Study medication can only be supplied to the Investigator after documentation of all ethical and legal requirements for starting the study has been received by the Sponsor or

designee (Coordinating Center). This documentation must also include an IRB/IEC membership list that contains members' occupations and qualifications. If the IRB/IEC will not disclose the names of the committee members, it should be asked to issue a statement confirming that the composition of the committee is in accordance with GCP.

The Investigator will keep the IRB/IEC informed regarding the progress of the study, per institutional requirements. No changes will be made in the study without IRB/IEC approval, except when required to eliminate apparent immediate hazards to the subjects. In cases where any implemented deviation from, or a change of, the protocol to eliminate an immediate hazard(s) to trial participants is implemented without prior IRB/IEC approval, the implemented deviation should be notified as soon as possible not only to the IRB/IEC for review and approval/favorable opinion but also to the regulatory authority(ies).

While the study is ongoing and at study completion/discontinuation, the Investigator must submit to the IRB/IEC the following information in accordance with applicable regulatory requirements where the study is being conducted:

1. Information on serious or unexpected AEs, showing due diligence in providing this information as soon as possible
2. Periodic reports on the progress of the study
3. Final Study Summary upon study completion or closure.

Notification of the end of the trial will be sent to the IRB/IEC within 30 days after completion of the study close-out visit. In case the study is ended prematurely, the IRB/IEC will be notified within 15 days, including the reasons for the premature termination. The end of the trial is defined as the date of final analysis of the study data according to the SAP.

### *11.2.3 Study Documentation*

#### *Before the Start of the Study*

The following study documentation will be in place at the study site prior to the first administration of study drug:

- Fully signed protocol and protocol-supporting manuals
- Investigator's Brochure[29]
- Investigator Protocol Agreement form signed by the Investigator
- IRB/IEC-approved copy of the ICF
- Curriculum vitae of the Investigator and all sub-investigators listed on the FDA Form 1572
- A letter of IRB/IEC approval for protocol
- A list of members of the IRB/IEC and their affiliations
- A copy of the Investigator-signed FDA 1572 form
- An Investigator-signed financial disclosure form
- Investigator/site study contract.

***During the Study***

The following documentation should be added to the site study file during study conduct:

- Any paper source forms completed and subsequently entered into the study database. An explanation should be given for all missing data and any protocol deviations documented in the site study file
- Any changes to the documentation identified above (see ***Before the Start of the Study***)
- Shipping documents relating to shipment of medication (drug accountability) and bioanalytical samples
- Copies of relevant correspondence such as letters, emails, meeting notes, and telephone calls.

***After the Study***

After completion or premature termination of the trial, all of the documents identified should be in the file, together with the following:

- Study drug accountability documents
- Audit certificates (if applicable)
- Investigator delegation of responsibilities log
- Site signature log
- Subject enrollment log
- Subject identification log
- Substantive correspondence with the Sponsor and IRB/IEC
- Notification of the end of the trial to the IRB/IEC.

#### *11.2.4 Delegation of Investigator Responsibilities*

The Investigator must (a) ensure that any individual to whom a task is delegated is qualified by education, training, and experience (and licensure, if relevant) to perform the task; and (b) provide adequate supervision. The Investigator should maintain a list of sub-investigators and other appropriately qualified persons to whom he or she has delegated significant study-related duties.

## 11.3 Protocol Deviations

### *11.3.1 Protocol Deviation Definitions*

Protocol deviations should be documented in accordance with the Manual of Operations.

***Protocol Deviation***

A protocol deviation is any change, divergence, or departure from the study design or procedures of a research protocol that has not been approved by the IRB/IEC.

Changes or alterations in the conduct of the trial which do not have a major impact on the subject's rights, safety or well-being, or the completeness, accuracy and reliability of the study data are considered minor protocol deviations.

***Important Protocol Deviation***

An important protocol deviation is a deviation from the IRB/IEC-approved protocol that may significantly affect the subject's rights, safety, or well-being and/or the completeness, accuracy and reliability of the study data. This includes examples such as inappropriate consent, errors in drug dosing, or lack of reporting of safety data.

### *11.3.2 Reporting Important Protocol Deviations*

Upon discovery of an important protocol deviation, the Investigator is responsible for reporting the important protocol deviation to the IRB/IEC and Sponsor or designee (Coordinating Center) within 24 hours of discovery, or according to local site requirements.

All deviations must be recorded in the CTMS.

## 11.4 Study Records Retention and Direct Access to Source Documents

Following completion of the clinical study, the medical files of trial subjects as well as other essential documents shall be retained by the Sponsor and the Investigator for at least 10 years after completion of the clinical trial, or for a period of time as required by the applicable regulatory authority.

The Investigator must maintain a copy of all data collected for each subject treated (including eCRFs and source data). In order to assure the accuracy of data collected in the eCRF, it is mandatory that representatives of the Sponsor, or designee, as well as representatives of health authorities have direct access to original source documents (e.g., subject records, subject charts, and laboratory reports). During the review of these documents, the anonymity of the subject will be respected with strict adherence to professional standards of confidentiality.

The Sponsor reserves the right to terminate the study for refusal of the Investigator to supply source documentation of work performed in this clinical study.

The following includes, but is not limited to, the records that must be retained by the Investigator:

1. Signed informed consent documents for all subjects
2. Subject identification log
3. Subject enrollment log
4. Record of all relevant communications between the Investigator and the IRB/IEC
5. Composition of the IRB/IEC
6. Record of all relevant communications between the Investigator and the Sponsor (or designee)
7. List of sub-investigators and other appropriately qualified persons to whom the Investigator has delegated significant study-related duties, together with their roles in the study and their signatures
8. Drug accountability records (See **Section 5.8.4**)
9. Record of any body fluids or tissue samples retained
10. All other source documents (subject records, hospital records, laboratory records, etc.)
11. All other documents as listed in Section 8 of the ICH consolidated guideline on GCP (Essential Documents for the Conduct of a Clinical Trial).

## 11.5 Study Monitoring

In accordance with applicable regulations, GCP, and the procedures of the Sponsor or its designees, the Study Monitor will periodically contact the site and conduct on-site visits. The extent, nature, and frequency of on-site visits will be based on enrollment rate and data quality at the site. Through frequent communications (e.g., letter, e-mail, and telephone), the Study Monitor will ensure that the investigation is conducted according to protocol and regulatory requirements.

During these contacts, the monitoring activities will include:

1. Checking and assessing the progress of the study
2. Reviewing study data collected to date for completeness and accuracy

3. Reviewing compliance with protocol assessments
4. Conducting source document verification by reviewing eCRF database data against source documents when available (e.g., medical records, subject diaries, ICF [and assent, if applicable], laboratory result reports, raw data collection forms)
5. Identifying any issues and addressing resolutions.

These activities will be done in order to verify that the:

1. Data are authentic, accurate, and complete
2. Safety and rights of the subjects are being protected
3. Study is conducted in accordance with the currently approved protocol (and any amendments), GCP, and all applicable regulatory requirements.

The Investigator will allow the Study Monitor direct access to all relevant documents, and allocate his/her time and the time of his/her staff to the Study Monitor to discuss findings and any relevant issues.

In addition to contacts during the study, the Study Monitor will contact the site prior to the start of the study to discuss the protocol and data collection procedures with site personnel.

At study closure, Study Monitors will conduct all activities as indicated in **Section 11.7.**

## 11.6 Quality Assurance

At its discretion, the Sponsor or its designee may conduct a quality assurance audit of this study. Auditing procedures of the Sponsor and/or its designee will be followed in order to comply with GCP guidelines and ensure acceptability of the study data for registration purposes. If such an audit occurs, the Investigator will give the auditor direct access to all relevant documents, and will allocate his/her time and the time of his/her staff to the auditor as may be required to discuss findings and any relevant issues.

In addition, regulatory authorities and/or the IRB/IEC may conduct an inspection of this study. If such an inspection occurs, the Investigator will allow the inspector direct access to all source documents, eCRFs, and other study documentation for source data check

and/or on-site audit inspection. The Investigator must allocate his/her time and the time of his/her staff to the inspector to discuss findings of any relevant issues.

An explanation will be given for all missing, unused, and spurious data in the relevant section of the study report.

## 11.7 Study Termination and Site Closure

Upon completion of the study, the following activities, when applicable, must be conducted by the Study Monitor in conjunction with the Investigator, as appropriate:

1. Provision of all study data to the Sponsor
2. Data clarifications and/or resolutions
3. Accounting, reconciliation, and final disposition of used and unused study medication
4. Review of site study records for completeness.

In addition, the Sponsor reserves the right to temporarily suspend or prematurely terminate this study for any reason.

If the study is suspended or terminated for safety reason(s), the Sponsor will promptly inform the Investigator, and will also inform the regulatory authorities of the suspension or termination of the study and the reason(s) for the action. The Investigator is responsible for promptly informing the IRB/IEC, and providing the reason(s) for the suspension or termination of the study.

If the study is prematurely terminated, all study data must be returned to the Sponsor. In addition, the site must conduct final disposition of all unused study medications in accordance with the Sponsor procedures for the study.

## 11.8 Site Termination

The Sponsor may at any time, at its sole discretion, terminate the study site for various reasons, including, without limitation, the following:

1. Failure of the Investigator to enroll subjects into the study

2. Failure of the Investigator to comply with applicable laws and/or pertinent regulations
3. Submission of knowingly false information from the research facility to the Sponsor, Study Monitor, or regulatory authorities
4. Insufficient adherence to protocol requirements.

If participation of a study site is terminated, the Sponsor and Study Chair will issue a written notice to the Investigator. The written notice will contain the reasons for taking such action. If the study site is terminated for noncompliance, appropriate regulatory authorities will also be notified by the Sponsor.

Study termination and follow-up will be performed in compliance with relevant regulations where the study is being conducted.

### 11.9 Discontinuation of Study

The Sponsor reserves the right to discontinue the study for any reason at any time. In addition, the study may be stopped at any time if, in the opinion of the Sponsor and Medical Monitor, safety data suggest that the medical safety of subjects is being or may become compromised.

## 12 DISCLOSURE OF DATA

### 12.1 Confidentiality

The rights and privacy of participants in this study will be protected at all times. All personal details of subjects will be treated as confidential by the Investigator. All applicable data protection laws in the relevant countries will be adhered to at all times.

Subject names will remain confidential and will not be included in the database. Only enrollment number, and birth date will be recorded on the eCRF. If the subject's name appears on any other document collected (e.g., hospital discharge summary), the name must be obliterated before the document is transmitted to the Sponsor or its designee. All study findings will be stored in electronic databases. The subjects' parents or guardians will give explicit permission for representatives of the Sponsor, regulatory authorities, and the IRB/IEC to inspect the subjects' medical records to verify the information

collected. The subjects' parents or guardians will be informed that all personal information made available for inspection will be handled in the strictest confidence and in accordance with all applicable data protection / privacy laws in the relevant countries.

The parents or guardians of all participants in the United States will provide written authorization to disclose private health information either as a part of the written ICF or as a separate authorization form. The authorization will contain all required elements specified by 21 CFR 50, and will contain a waiver of subject access to study-related private health information until the conclusion of the clinical study. The authorization will remain valid and in full force and effect until the first to occur of (1) the expiration of 2 years after the study medication is approved for the indication being studied, or (2) the expiration of 2 years after the research program is discontinued. Individual subject medical information obtained during this study is confidential, and its disclosure to third parties (other than those mentioned in this section) is strictly prohibited. In addition, medical information obtained during this study may be provided to the subject's personal physician or to other appropriate medical personnel when required in connection with the subject's continued health and welfare.

The study Investigator will maintain a subject identification log (enrollment numbers and corresponding subject names) to enable records to be identified.

## 12.2 Publication

ReveraGen BioPharma, Inc. retains the ownership of all data and results collected during this study. Therefore, the Sponsor reserves the right to use the data from this present study, either in the form of eCRFs (or copies of these), or in the form of a report, with or without comments and analysis in order to submit them to Health Authorities of any country.

Furthermore, in the event that the clinical research leads to patentable results, the Investigator (or entity acting on his/her behalf according to local requirements) shall refrain from filing patent application(s). Patent applications will be filed by ReveraGen BioPharma, Inc. or another entity delegated by ReveraGen BioPharma, Inc.

All information concerning the product as well as any information such as clinical indications for the drug, its formula, methods of manufacture and other scientific data relating to it, that have been provided by the Sponsor or designee, and are unpublished, are confidential and must remain the sole property of the Sponsor. The Investigator will agree to use the information only for the purposes of carrying out this study and for no other purpose unless prior written permission from the Sponsor is obtained. The Sponsor has full ownership of the eCRFs completed as part of the study.

By signing the study protocol, the Investigator agrees that the results of the study may be used for the purposes of national and international registration, publication, and information for medical and pharmaceutical professionals by the Sponsor. If necessary, the authorities will be notified of the Investigator's name, address, qualifications, and extent of involvement.

The Sponsor or designee will prepare a final report on the study. The Investigator may not publish or present any information on this study without the express written approval of the Sponsor. Additionally, the Sponsor, may, for any reason, withhold approval for publication or presentation.

# 13 INVESTIGATOR PROTOCOL AGREEMENT

The Investigator Protocol Agreement at the front of this document must be signed by the study site Principal Investigator. The Investigator must retain the original and an electronic signed copy must be kept on file by the Sponsor. The completed Protocol Agreement signifies review and acceptance of the protocol amendment by the Principal Investigator prior to initiation of the study.

# 14 REFERENCES


1. Mah JK, Korngut L, Dykeman J, Day L, Pringsheim T, Jette N. A systemic review and meta-analysis on the epidemiology of Duchenne and Becker muscular dystrophy. *Neuromuscul Disord*. 2014; 24(6): 482-91.
2. Hoffman EP, Brown RH and Kunkel LM. Dystrophin: the protein product of the Duchenne muscular dystrophy locus. *Cell*. 1987; 51: 919-28.
3. Evans NP, Misyak SA, Robertson JL, Bassaganya-Riera J, Grange RW. Immune mediated mechanisms potentially regulate the disease time course of Duchenne muscular dystrophy and provide targets for therapeutic intervention. *PM R*. 2009; 1(8): 755-68.
4. Kumar A and Boriek AM. Mechanical stress activates the nuclear factor-kappa B pathway in skeletal muscle fibers: a possible role in Duchenne muscular dystrophy. *FASEB J*. 2003; 17(3): 386-96.
5. Acharyya S, Villalta SA, Bakkar N, et al. Interplay of IKK/NF-kappa B signaling in macrophages and myofibers promotes muscle cell degeneration in Duchenne muscular dystrophy. *J Clin Invest*. 2007; 117(4): 889-901.
6. Dogra C, Changotra H, Wergedal JE, Kumar A. Regulation of phosphatidylinositol-3-kinase (PI3K)/Akt and nuclear factor-kappa B signaling pathways in dystrophin-deficient skeletal muscle in response to mechanical stretch. *J Cell Physiol*. 2006; 208(3): 575-85.
7. Pahl HL. Activators and target genes of Rel/NF-kappa B transcription factors. *Oncogene*. 1999; 18(49): 6853-66.
8. Spencer MJ, Montecino-Rodriguez E, Dorshkind K, Tidball JG. Helper (CD4(+)) and cytotoxic (CD8(+)) T cells promote the pathology of dystrophin-deficient muscle. *Clin Immunol*. 2001; 98(2): 235-43.
9. Spencer MJ and Tidball JG. Do immune cells promote the pathology of dystrophin-deficient myopathies? *Neuromuscul Disord*. 2001; 11(6-7): 556-64.

10. Malik V, Rodino-Klapac L and Mendell JR. Emerging drug for Duchenne muscular dystrophy. *Expert Opin Emerg Drugs*. 2012; 17(2): 261-77.

11. Reeves EK, Hoffman EP, Nagaraju K, Damsker JM, McCall JM. VBP15: preclinical characterization of a novel anti-inflammatory delta 9,11 steroid. *Bioorg Med Chem*. 2013; 21(8): 2241-9.

12. Bushby K, Finkel R, Birnkrant DJ, et al. Diagnosis and management of Duchenne muscular dystrophy, part 1: diagnosis, and pharmacological and psychosocial management. *Lancet Neurol*. 2010; 9(1): 77-93.

13. Pane M, Mazzone ES, Sivo S, et al. Long term natural history data in ambulant Boys with Duchenne muscular dystrophy: 36-month changes. *PlosOne*. 2014; 10: 1371/journal.pone.0108205.

14. Manzur AY, Kuntzer T, Pike M, Swan AV. Glucocorticoid corticosteroids for Duchenne muscular dystrophy. *Cochrane Database Syst Rev.* 2008; (1): CD003725.

15. Heier CR, Damsker JM, Yu Q, et al. VBP15, a novel anti-inflammatory and membrane-stabilizer improves muscular dystrophy without side effects. *EMBO Mol Med*. 2013; 5(10): 1569-85.

16. Damsker JM, Dillingham BC, Rose MC, et al. VBP15, a glucocorticoid analogue, is effective at reducing allergic lung inflammation in mice. *Plos One*. 2013; 8(5): e63871.

17. Dillingham BC, Knoblach SM, Many GM, et al. VBP15, a novel anti-inflammatory, is effective at reducing the severity of murine experimental autoimmune encephalomyelitis. *Cell Mol Neurobiol*. 2015; 35(3): 377-87.

18. Dadgar S, Wang Z, Johnston H, et al. Asynchronous remodeling is a driver of failed regeneration in Duchenne muscular dystrophy. *J Cell Biol*. 2014; 207(1): 139-58.

19. Raman SV, Hor KN, Mazur W, Halnon NJ, Kissel JT, He X, Tran T, Smart S, McCarthy B, Taylor MD, Jefferies JL, Rafael-Fortney JA, Lowe J, Roble SL,

Cripe LH. Eplerenone for early cardiomyopathy in Duchenne muscular dystrophy: a randomised, double-blind, placebo-controlled trial. *Lancet Neurol.* 2015; 14(2): 153-61.

20. Avioli LV. Glucocorticoid effects on statural growth. *Br J Rheumatol*. 1993; 32 Suppl 2: 27-30.
21. Wolthers OD and Pedersen S. Short term linear growth in asthmatic children during treatment with prednisolone. *BMJ*. 1990; 301(6744): 145-8.
22. Bircan Z, Soran M, Yildirim I, et al. The effect of alternate-day low dose prednisolone on bone age in children with steroid dependent nephrotic syndrome. *Int Urol Nephrol*. 1997; 29(3): 357-61.
23. Manolagas SC, Weinstein RS. New developments in the pathogenesis and treatment of steroid-induced osteoporosis. *J Bone Miner Res*. 1999; 14(7): 1061-6.
24. Sali A, Guerron AD, Gordish-Dressman H, et al. Glucocorticoid-treated mice are an inappropriate positive control for long-term preclinical studies in the mdx mouse. *Plos One*. 2012; 7(4): e34204.8
25. Kauh E, Mixson L, Malice M-P, Mesens S, Ramael S, Burke J, Reynders T, Van Dyck K, Beals C, Rosenberg E, and Ruddy M. Prednisone affects inflammation, glucose tolerance, and bone turnover within hours of treatment in healthy individuals. *European J Endocrinol.* 2012; 166: 459–67.
26. Wehling-Henricks M, Oltmann M, Rinaldi C, Myung KH, and Tidball JG. Loss of positive allosteric interactions between neuronal nitric oxide synthase and phosphofructokinase contributes to defects in glycolysis and increased fatigability in muscular dystrophy. *Hum Mol Genet*. 2009; 18(18): 3439-51.
27. Younes M, Neffati F, Touzi M, Hassen-Zrour S, Fendri Y, Bejia I, Ben Amor A, Bergaoui N, and Najjar MF. Systemic effects of epidural and intraarticular glucocorticoid injections in diabetic and non-diabetic patients. *Joint Bone Spine*. 2007; 74(5): 472-6.

28. Moon HJ, Choi KH, Lee SI, Lee OJ, Shin JW, and Kim TW. Changes in blood glucose and cortisol levels after epidural or shoulder intra-articular glucocorticoid injections in diabetic or nondiabetic patients. *Am J Phys Med Rehabil*. 2014; 93(5): 372-8.

29. Investigator's Brochure, Version 8, Vamorolone (VBP15) (17α, 21-dihydroxy-16α-methyl-pregna-1,4,9(11)-triene-3,20-dione) 1.33% and 4% Oral Suspension, ReveraGen BioPharma, Inc., September 24, 2018.

30. Fleishaker DL, Mukherjee A, Whaley FS, Daniel S, Zeiher BG. Safety and pharmacodynamic dose response of short-term prednisone in healthy adult subjects: a dose ranging, randomized, placebo-controlled, crossover study. BMC Musculoskelet Disord. 2016 Jul 16;17:293.

31. Hathout Y, Conklin LS, Seol H, Gordish-Dressman H, Brown KJ, Morgenroth LP, Nagaraju K, Heier CR, Damsker JM, van den Anker JN, Henricson E, Clemens PR, Mah JK, McDonald C, Hoffman EP. Serum pharmacodynamic biomarkers for chronic corticosteroid treatment of children. *Sci Rep*. 2016; Aug 17;6:31727. doi:10.1038/srep31727.

32. Agwu, J.C., et al., *Tests of adrenal insufficiency*. Arch Dis Child, 1999. **80**(4): p. 330-3.

33. van Staa TP, Leufkens HG, Abenhaim L, Zhang B, Cooper C. Oral corticosteroids and fracture risk: relationship to daily and cumulative doses. *Rheumatology (Oxford)*. 2000 Dec; 39(12): 1383-9.

34. FDA Draft Guidance for Industry: Duchenne muscular dystrophy and related dystrophinopathies: developing drugs for treatment. June 2015.

35. Brooke MH, Griggs RC, Mendell JR, et al. Clinical trial in Duchenne dystrophy. I. The design of the protocol. *Muscle & Nerve*. 1981; 4(3): 186-97.

36. Mazzone ES, Messina S, Vasco G, et al. Reliability of the North Star Ambulatory Assessment in a multicentric setting. *Neuromuscul Disord*. 2009; 19(7): 458-61.

37. McDonald CM, Henricson EK, Han JJ, et al. The 6-minute Walk Test as a new outcome measure in Duchenne muscular dystrophy. *Muscle Nerve.* 2010; 41(4): 500-10.

38. Bello L, Kesari A, Gordish-Dressman H, et al. Genetic modifiers of ambulation in the Cooperative International Neuromuscular Research Group Duchenne natural history study. *Ann Neurol*. 2015; 77(4):684-696.

39. Spurney C, Shimizu R, Morgenroth LP, et al. Cooperative International Neuromuscular Research Group Duchenne Natural History Study demonstrates insufficient diagnosis and treatment of cardiomyopathy in Duchenne muscular dystrophy. *Muscle Nerve*. 2014; Aug; 50(2):250-256.

40. McDonald CM, Henricson EK, Abresch RT, et al. The Cooperative International Neuromuscular Research Group Duchenne natural history study – a longitudinal investigation in the era of glucocorticoid therapy: design of protocol and the methods used. *Muscle Nerve.* 2013; 48(1):32-54.

41. Henricson EK, Abresch RT, Cnaan A, et al. The Cooperative International Neuromuscular Research Group Duchenne natural history study: glucocorticoid treatment preserves clinically meaningful function milestones and reduces rate of disease progression as measured by manual muscle testing and other commonly used clinical trial outcome measures. *Muscle Nerve.* 2013; 48(1):55-67.

42. Escolar DM, Hache LP, Clemens PR, et al. Randomized, blinded trial of weekend vs. daily prednisone in Duchenne muscular dystrophy. *Neurology.* 2011; 77(5):444-452.

# 15 APPENDICES

## Appendix 15.1 Protocol Amendment #2 Complete List of Changes

The following changes have been incorporated into the protocol under this protocol amendment, as summarized in Protocol Amendment Tracking, Reasons for Protocol Amendment #2. Protocol sections that have been changed are itemized below with the original and revised text. Changes that were strictly editorial (e.g. grammar, punctuation, correction of spelling errors) are not included.

**Section Changed:**

**Title Page:**

***Original Text:***

| | |
|---|---|
| **North America** | Benjamin Schwartz, M.D., Ph.D.<br>Phone: (314) 220 2067<br>Email: bdschw@camdengroup.com |

***Revised Text:***

| | |
|---|---|
| **North America** | Benjamin Schwartz, M.D., Ph.D.<br>Phone: (314) 220 7067<br>Email: bdschw@camdengroup.com |

**Sections Changed:**

**Synopsis: Exploratory Objectives**
**Section 2.1.3: Exploratory Objectives**

***Original Text:***

1. To evaluate the satisfaction with treatment of vamorolone administered orally at daily doses of 2.0 mg/kg and 6.0 mg/kg over a 24-week treatment period vs. daily prednisone 0.75 mg/kg in ambulant boys ages 4 to <7 years with DMD;
2. To evaluate the effect of vamorolone administered orally at daily doses of 2.0 mg/kg and 6.0 mg/kg over a 24-week treatment period vs. daily prednisone 0.75 mg/kg on Quality of Life and neuropsychology;
3. To assess the ease of administration of the study medication suspension to ambulant boys ages 4 to <7 years with DMD;
4. To compare the effects of vamorolone administered orally at daily doses of 2.0 mg/kg and 6.0 mg/kg over a 24-week treatment period vs. placebo on potential serum pharmacodynamics (PD) biomarkers of safety and efficacy in ambulant boys ages 4 to <7 years with DMD; and
5. To compare the effects of vamorolone administered orally at daily doses of 2.0 mg/kg and 6.0 mg/kg over a 24-week treatment period vs. daily prednisone 0.75 mg/kg on potential serum PD biomarkers of safety and efficacy in ambulant boys ages 4 to <7 years with DMD.

***Revised Text:***

1. To evaluate the satisfaction with treatment of vamorolone administered orally at daily doses of 2.0 mg/kg and 6.0 mg/kg over a 24-week treatment period vs. daily prednisone 0.75 mg/kg in ambulant boys ages 4 to <7 years with DMD;
2. To evaluate the effect of vamorolone administered orally at daily doses of 2.0 mg/kg and 6.0 mg/kg over a 24-week treatment period vs. daily prednisone 0.75 mg/kg on behavior and neuropsychology;
3. To evaluate the effect of vamorolone administered orally at daily doses of 2.0 mg/kg and 6.0 mg/kg over a 24-week treatment period vs. placebo on physical functioning;
4. To assess the ease of administration of the study medication suspension to ambulant boys ages 4 to <7 years with DMD;
5. To compare the effects of vamorolone administered orally at daily doses of 2.0 mg/kg and 6.0 mg/kg over a 24-week treatment period vs. placebo on potential serum pharmacodynamics (PD) biomarkers of safety and efficacy in ambulant boys ages 4 to <7 years with DMD;
6. To compare the effects of vamorolone administered orally at daily doses of 2.0 mg/kg and 6.0 mg/kg over a 24-week treatment period vs. daily prednisone 0.75 mg/kg on potential serum PD biomarkers of safety and efficacy in ambulant boys ages 4 to <7 years with DMD; and
7. To determine if candidate genetic modifiers of DMD (gene polymorphisms associated with disease severity, or response to glucocorticoid treatment) are similarly associated with vamorolone-treated DMD patients (baseline disease severity, or response to vamorolone or prednisone treatment).

## Section Changed:

## Synopsis: Study Design, paragraph 6

### *Original Text*

At the end of the Treatment Period #2, subjects will be given the option of enrolling into a long-term extension study or to transition to standard of care treatment for DMD (may include glucocorticoids). Subjects completing VBP15-004 and enrolling directly into the long-term extension study will not need to taper their vamorolone dose prior to enrollment into the study. All other subjects will begin a 4-week double-blind Dose-tapering Period during which the dose of study medication will be progressively reduced and discontinued.

### *Revised Text*

At the end of the Treatment Period #2, subjects may be given access to vamorolone through an additional study or general access program, or given the option to transition to standard of care treatment for DMD (may include glucocorticoids). Subjects completing VBP15-004 and enrolling directly into an additional vamorolone study or general access program to receive vamorolone will not need to taper their vamorolone dose prior to enrollment. All other subjects will begin a 4-week double-blind Dose-tapering Period during which the dose of study medication will be progressively reduced and discontinued.

## Sections Changed:

## Throughout Text

### *Original Text:*

long-term extension study

### *Revised Text:*

additional vamorolone study or general access program

## Sections Changed:

## Synopsis: Exclusion Criteria, #7-15
## Section 4.3: Exclusion Criteria, #7-15

### *Original Text:*

7. Subject is currently being treated or has received previous treatment with oral glucocorticoids or other immunosuppressive agents [Notes: Past transient use of oral or inhaled glucocorticoids or other oral immunosuppressive agents for indication other than DMD for no longer than 3 months cumulative, with last use at least 3 months (or last use at least one month prior for inhaled glucocorticoids) prior to first dose of study medication, will be considered for eligibility on a case-by-case basis. Inhaled and/or topical glucocorticoids prescribed for an indication other than DMD are permitted if last use is at least 4 weeks prior to first dose of study medication or are administered at stable dose beginning at least 4 weeks prior to first dose of study medication, and are anticipated to be used at the stable dose regimen for the duration of the study];
8. Subject has an allergy or hypersensitivity to the study medication or to any of its constituents;
9. Subject has used idebenone within 4 weeks prior to the first dose of study medication;
10. Subject has severe behavioral or cognitive problems that preclude participation in the study, in the opinion of the Investigator;
11. Subject has previous or ongoing medical condition, medical history, physical findings or laboratory abnormalities that could affect safety, make it unlikely that treatment and follow-up will be correctly completed or impair the assessment of study results, in the opinion of the Investigator;
12. Subject is taking (or has taken within 4 weeks prior to the first dose of study medication) herbal remedies and supplements which can impact muscle strength and function (e.g., Co-enzyme Q10, creatine, etc);
13. Subject is taking (or has taken within 3 months prior to the first dose of study medication) any medication indicated for DMD, including Exondys51 and Translarna;
14. Subject is currently taking any other investigational drug or has taken any other investigational drug within 3 months prior to the first dose of study medication; or
15. Subject has previously been enrolled in the study.

### *Revised Text:*

7. Subject is currently being treated or has received previous treatment with oral glucocorticoids or other immunosuppressive agents [Notes: Past transient use of oral or inhaled glucocorticoids or other oral immunosuppressive agents for no longer than 1 month cumulative, with last use at least 3 months prior to first dose of study medication, will be considered for eligibility on a case-by-case basis, unless discontinued for intolerance. Inhaled and/or topical glucocorticoids are permitted if last use is at least 4 weeks prior to first dose of study medication or if administered at stable dose beginning at least 4 weeks prior to first dose of study medication and anticipated to be used at the stable dose regimen for the duration of the study];
8. Subject has an allergy or hypersensitivity to the study medication or to any of its constituents;
9. Subject has used idebenone within 4 weeks prior to the first dose of study medication;
10. Subject has severe behavioral or cognitive problems that preclude participation in the study, in the opinion of the Investigator;
11. Subject has previous or ongoing medical condition, medical history, physical findings or laboratory abnormalities that could affect safety, make it unlikely that treatment and follow-up will be correctly completed or impair the assessment of study results, in the opinion of the Investigator;

12. Subject is taking (or has taken within 4 weeks prior to the first dose of study medication) herbal remedies and supplements which can impact muscle strength and function (e.g., Co-enzyme Q10, creatine, etc);
13. Subject is taking (or has taken within 3 months prior to the first dose of study medication) any medication indicated for DMD, including Exondys51 and Translarna;
14. Subject has been administered a live attenuated vaccine within 14 days prior to the first dose of study medication;
15. Subject is currently taking any other investigational drug or has taken any other investigational drug within 3 months prior to the first dose of study medication;
16. Subject has a sibling who is currently enrolled in any vamorolone study or Expanded Access Program, or who intends to enroll in any vamorolone study or Expanded Access Program during the subject's participation in the VBP15-004 study; or
17. Subject has previously been enrolled in the study.

## Section Changed:

## Synopsis: Individual Subject Study Duration, paragraph 2

### *Original Text*

Subjects who complete the Treatment Period Week 48 assessments will be given the option of continuing vamorolone treatment in a long-term extension study under separate protocol. Subjects who continue directly with vamorolone treatment in the long-term extension study will be discharged from the VBP15-004 study following completion of all Week 48 assessments and the Week 48 Follow-up Visit ACTH Stimulation Test, and will not participate in the Dose-tapering Period.

### *Revised Text*

Subjects who complete the Treatment Period Week 48 assessments may be given the option of continuing vamorolone treatment in an additional vamorolone study or general access program under separate protocol. Subjects who continue directly with vamorolone treatment in the additional vamorolone study or general access program will be discharged from the VBP15-004 study following completion of all Week 48 assessments and the Week 48 Follow-up Visit ACTH Stimulation Test, and will not participate in the Dose-tapering Period.

## Section Changed:

## Synopsis: Study Drug Formulation, Dosage, and Administration, Treatment Period #1, paragraph 2

### *Original Text*

Study drugs will be administered in the study unit on Day 1, and at the Week 2, Week 12 and Week 24 study visits; all other doses will be administered at home. Study drug oral suspensions will be administered by mouth using a volumetric syringe. Following administration of the dose of study drug, the syringe will be filled once with water and the water will be administered by mouth using the volumetric syringe. Prednisone or matching placebo tablets will be taken right after the dose of suspension. The subject will then drink approximately 50 mL (approximately 2 ounces) of water to ensure the full dose has been ingested.

### *Revised Text*

Study drugs will be administered in the study unit on Day 1, and at the Week 2, Week 12 and Week 24 study visits; all other doses will be administered at home. Study drug oral suspensions will be administered by mouth using a volumetric syringe. Following administration of the dose of study drug suspension, the syringe will be filled once with water and the water will be administered by mouth using the volumetric syringe. Prednisone or matching placebo tablets will be taken either immediately before or immediately after the dose of suspension. The subject will then drink approximately 50 mL (approximately 2 ounces) of water to ensure the full dose has been ingested.

## Sections Changed:

## Synopsis: Study Summary, paragraph 5
## Section 3.1: Overall Study Design, paragraph 7

### *Original Text:*

There is flexibility in the timing of completion of some of the scheduled Week 24 and Week 48 assessments. The scheduled physical examination, weight, vital signs, clinical laboratory tests, blood draws for PD biomarker analysis, Ease of Study Medication Administration Assessment, and functional assessments (TTSTAND, TTCLIMB, TTRW, NSAA, 6MWT, hand-held myometry, ROM) should all be performed on the date of the Week 24 or Week 48 dose of study medication. However, subjects may return to the study site on the day following the Week 24 or Week 48 dose of study medication and/or the day of the Week 24 or Week 48 Follow-up Visit for completion of the DXA scan, spine X-rays (Week 24 only), Fracture Questionnaire, 12-lead ECG, 2-D echocardiography, eye examination, PODCI, TSQM, Child Behavior Checklist, PARS III, and Blindedness Assessment (Week 24 only).

### *Revised Text:*

There is flexibility in the timing of completion of some of the scheduled Week 24 and Week 48 assessments. The scheduled physical examination, weight, vital signs, clinical laboratory tests, blood draws for PD biomarker analysis, blood draw for DNA testing (Week 24 only), Ease of Study Medication Administration Assessment, PODCI, PARS III, and functional assessments (TTSTAND, TTCLIMB, TTRW, NSAA, 6MWT, hand-held myometry, ROM) must all be performed on the date of the Week 24 or Week 48 dose of study medication. The 12-lead ECG may be performed on the date of the Week 24 or Week 48 dose of study medication, the day following the Week 24 or Week 48 dose of study medication, or the day of the Week 24 or Week 48 Follow-up Visit. For the Week 24 assessments, completion of the DXA scan, spine X-rays, Fracture Questionnaire, 2-D echocardiography, eye examination, TSQM, and Blindedness Assessment may be performed up to 7 days following the date of the Week 24 dose of study medication to accommodate need for additional scheduling flexibility. For the Week 48 assessments, completion of the DXA scan, Fracture Questionnaire, 2-D echocardiography, eye examination, and TSQM may be performed on the date of the final Week 48 dose of study medication, the day following the Week 48 dose of study medication, or the day of the Week 48 Follow-up Visit for subjects who will receive additional vamorolone therapy by enrolling directly into an additional vamorolone study or general access program, or up to 7 days following the date of the final Week 48 dose of study medication for subjects participating in the Dose-tapering Period.

## Sections Changed:

## Synopsis: Study Summary, paragraph 7
## Section 3.1: Overall Study Design, paragraph 9

### *Original Text*

Subjects who complete the VBP15-004 study assessments through the Week 48 Visit and Week 48 Follow-up Visit will be given the opportunity to enroll into a Phase IIb long-term extension study, or alternatively transition to standard of care treatment (including glucocorticoids) for DMD. Standard of care treatment for DMD may be offered to the subject following completion of the Phase IIb VBP15-004 study, if the

subject's parent or guardian does not wish to enroll the subject in the long-term extension study and/or the Investigator feels it to be in the best interest of the subject.

### *Revised Text*

Subjects who complete the VBP15-004 study assessments through the Week 48 Visit and Week 48 Follow-up Visit may be given access to vamorolone through an additional study or general access program, or given the option to transition to standard of care treatment (including glucocorticoids) for DMD. Standard of care treatment for DMD may be offered to the subject following completion of the Phase IIb VBP15-004 study, if the subject's parent or guardian does not wish to enroll the subject in the additional vamorolone study or general access program and/or the Investigator feels it to be in the best interest of the subject.

## Section Changed:

## Synopsis: Exploratory Measures

### *Original Text*

- Treatment satisfaction questionnaire (TSQM)
- Pediatric Outcome Data Collection Instrument (PODCI)
- Child Behavior Checklist
- PARS III questionnaire
- Ease of Study Medication Administration Assessment
- Blindedness Assessment

### *Revised Text*

- Treatment satisfaction questionnaire (TSQM)
- Pediatric Outcome Data Collection Instrument (PODCI)
- PARS III questionnaire
- Ease of Study Medication Administration Assessment
- Blindedness Assessment
- DNA testing for candidate genetic modifiers of DMD

## Sections Changed:

## Synopsis: Statistical Methods, Sample Size, paragraph 5
## Section 10.1: Sample Size Determination, paragraph 5

### *Original Text*

A drop-out rate is not considered; subjects who withdraw early from the study may be replaced, at the discretion of the Sponsor.

### *Revised Text*

If the number of subjects who withdraw early from the study is high, additional subjects may be enrolled to achieve approximately 120 subjects completing the Week 24 Visit assessments.

## Sections Changed:

## Synopsis: Statistical Methods, Adjustment for Multiple Comparisons, paragraphs 4 and 5
## Section 10.9.2: Adjustment for Multiple Comparisons, paragraphs 4 and 5

### *Original Text*

Secondary efficacy endpoints will be tested sequentially on change from baseline to Week 24 values (each dose of vamorolone vs. placebo). Only the doses that are significant for the primary efficacy endpoint (change in TTSTAND velocity at Week 24) will have the secondary endpoints tested. A 0.025 alpha level will be used for the sequential testing. Testing will stop once a p-value is >0.025 for one of the secondary efficacy endpoints vs. placebo. The Week 24 change from baseline values will be tested using this sequential testing procedure. The order of the secondary efficacy endpoints is as follows.

1. Six-minute Walk Test (6MWT)
2. Time to Run/Walk 10 meters Test (TTRW) velocity
3. North Star Ambulatory Assessment (NSAA)
4. Hand-held Myometry (knee extensors)
5. Hand-held Myometry (elbow flexors)
6. Time to Climb 4 Steps (TTCLIMB) velocity
7. Range of Motion (ROM) in the ankles

There is also one key secondary efficacy endpoint: each dose of vamorolone vs. prednisone using 6MWT at Week 24. This efficacy endpoint will also occur during Treatment Period #1 of the study. This secondary efficacy endpoint of vamorolone vs. prednisone is independent of the secondary efficacy endpoints of vamorolone vs. placebo, and has an adjustment for multiplicity applied. The secondary efficacy endpoint (6MWT) will separately utilize the same Bonferroni adjustment and will be conducted at the 0.025 (0.05/2) alpha level.

### *Revised Text*

Secondary efficacy endpoints will be tested sequentially on change from baseline to Week 24 values (each dose of vamorolone vs. placebo or prednisone [6MWT only]). Only the doses that are significant for the primary efficacy endpoint (change in TTSTAND velocity at Week 24) will have the secondary endpoints tested. A 0.025 alpha level will be used for the sequential testing. Testing will stop once a p-value is >0.025 for one of the secondary efficacy endpoints. The Week 24 change from baseline values will be tested using this sequential testing procedure. The order of the secondary efficacy endpoints is as follows.

1. Six-minute Walk Test (6MWT) vs. placebo
2. Time to Run/Walk 10 meters Test (TTRW) velocity vs. placebo
3. 6MWT vs. prednisone
4. North Star Ambulatory Assessment (NSAA) vs. placebo
5. Hand-held Myometry (knee extensors) vs. placebo
6. Hand-held Myometry (elbow flexors) vs. placebo
7. Time to Climb 4 Steps (TTCLIMB) velocity vs. placebo
8. Range of Motion (ROM) in the ankles vs. placebo

## Sections Changed:

## Synopsis: Patient Reported Outcome Analyses
## Section 10.9.7: Patient-Reported Outcome Exploratory Analyses

### *Original Text*

Patient Reported Outcomes including the TSQM, PODCI, Child Behavior Checklist, PARS III, Ease of Study Medication Administration Assessment, and the Blindedness Assessment will be listed and presented using descriptive statistics by treatment and time point.

***Revised Text***

Patient Reported Outcomes including the TSQM, PODCI, PARS III, Ease of Study Medication Administration Assessment, and the Blindedness Assessment will be listed and presented using descriptive statistics by treatment and time point. In addition, comparison will be made for each vamorolone dose group with placebo for the PODCI and for each vamorolone dose group with prednisone for the PARS III, with p values computed for the comparisons. Full details will be provided in the SAP.

**Section Changed:**

**List of Abbreviations**

***Original Text***

| | |
|---|---|
| DMD | Duchenne muscular dystrophy |
| DSMB | Data and Safety Monitoring Board |
| | |
| MAD | multiple ascending dose (study) |
| MD | Medical Doctor (physician) |

***Revised Text***

| | |
|---|---|
| DMD | Duchenne muscular dystrophy |
| DNA | deoxyribonucleic acid |
| DSMB | Data and Safety Monitoring Board |
| | |
| MAD | multiple ascending dose (study) |
| MCMC | Markov Chain Monte Carlo |
| MD | Medical Doctor (physician) |

**Section Changed:**

**Section 1.3: Clinical Experience**

***Original Text:***

Clinical experience is limited to a single completed Phase I clinical trial of vamorolone in healthy adult volunteers (VBP15-001), and to ongoing Phase IIa (VBP15-002), Phase IIa extension (VBP15-003), and long-term extension (VBP15-LTE) trials in DMD boys.

***Revised Text:***

Clinical experience with vamorolone is comprised of a completed Phase I clinical trial of vamorolone in healthy adult volunteers (VBP15-001), completed Phase IIa (VBP15-002) and Phase II extension (VBP15-003) trials in DMD boys, and an ongoing long-term extension (VBP15-LTE) trial in DMD boys.

**Section Changed:**

**Section 1.3.2: Phase IIa Study in 4 to 7 years Duchenne Muscular Dystrophy Boys (VBP15-002)**

***Original Text***

### *1.3.2 Phase IIa Study in 4 to 7 years Duchenne Muscular Dystrophy Boys (VBP15-002)*

***Preliminary Assessment of Vamorolone Pharmacokinetics in Boys with DMD***

In the first three cohorts of VBP15-002, 36 boys with DMD received oral doses of vamorolone once daily for 14 days: 12 boys received 0.25 mg/kg, 12 boys received 0.75 mg/kg, and 12 boys received 2.0 mg/kg. Based on the fasted-fed observations in adult men (**Section 1.3.1.2**), the drug was administered to the DMD boys with 8 ounces of full-fat milk containing 8 g of fat, or an equivalent high-fat food to maximize vamorolone exposure. The PK of vamorolone in all 12 DMD boys in each of the first three cohorts (0.25 mg/kg/day, 0.75 mg/kg/day, and 2.0 mg/kg/day) has been analyzed. The final dose group (6.0 mg/kg/day) has completed treatment (n = 12), but PK data have not yet been generated or interpreted.

Six blood samples were collected from each boy at 0, 1, 2, 4, 6, and 8 hours post-dose and the plasma from each sample was analyzed for vamorolone. The time-course of plasma drug concentrations were assessed using non-compartmental analytical (NCA) methods employing WinNonlin software (Certera). Selected exposure parameters from this relatively limited sampling schedule were compared with those from the healthy adult men from Study VBP15-001, who received a range of single and multiple oral doses of vamorolone.

The graphs displayed in **Figure 8** depict the plasma concentration versus time profiles on Days 1 and 14 for DMD boys receiving 0.25 mg/kg/day (**Figure 8a**), 0.75 mg/kg/day (**Figure 8b**), and 2.0 mg/kg/day (**Figure 8c**). The sampling schedule reasonably captured the absorption and disposition profiles. On Day 1, the mean $C_{max}$ (CV%) was 23.0 (13), 79.8 (34), and 199.0 (56) ng/mL, respectively, for subjects dosed at 0.25 mg/kg/day, 0.75 mg/kg/day and 2.0 mg/kg/day. On Day 14, mean $C_{max}$ (CV%) was 32.2 (47), 124.7 (33), and 252.5 (38) ng/mL, respectively, for the corresponding dose groups. On Day 1, the mean $AUC_{inf}$ (CV%) was 117.9 (40), 379.2 (31), and 761.1 (46) hr*ng/mL, respectively, for subjects dosed at 0.25 mg/kg/day, 0.75 mg/kg/day, and 2.0 mg/kg/day. On Day 14, the mean $AUC_{inf}$ (CV%) was 163.7 (30), 543.6 (27) and 1137.5 (41) hr*ng/mL, respectively, for the corresponding dose groups. The CV% values ranging from 13 to 56 indicate modest to moderate variability in these parameters among the subjects in each dosing group. The $C_{max}$ and $AUC_{inf}$ values tended to be higher on Day 14 compared to Day 1.

**Figure 8. Pharmacokinetic Data on Days 1 and 14 from 4-7 Year-Old DMD Boys Treated with 0.25 mg/kg/day (Figure 8a), 0.75 mg/kg/day (Figure 8b), and 2.0 mg/kg/day (Figure 8c) Vamorolone**

**Figure 8a. 0.25 mg/kg/day**

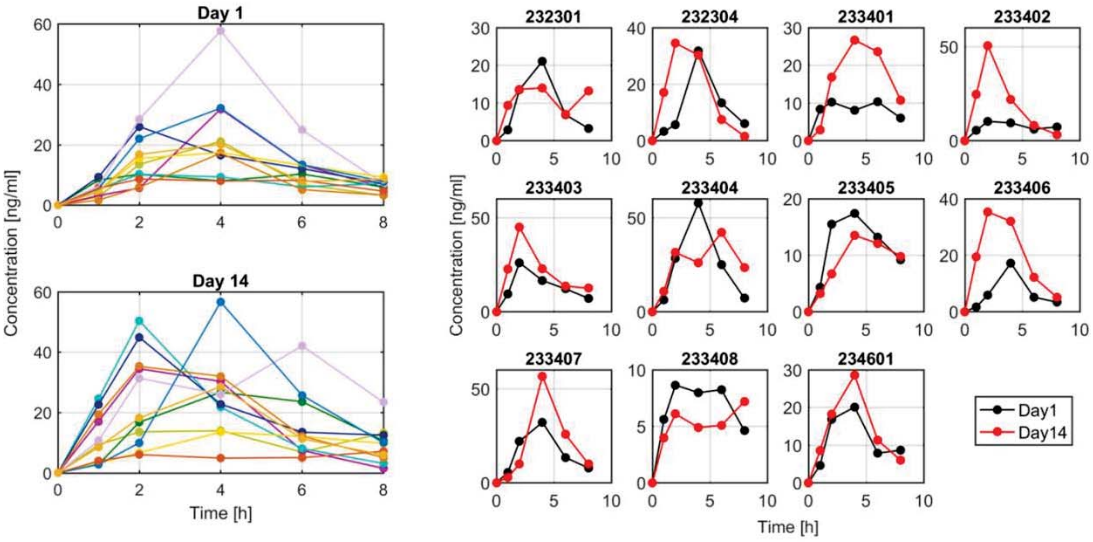


**Figure 8b. 0.75 mg/kg/day**

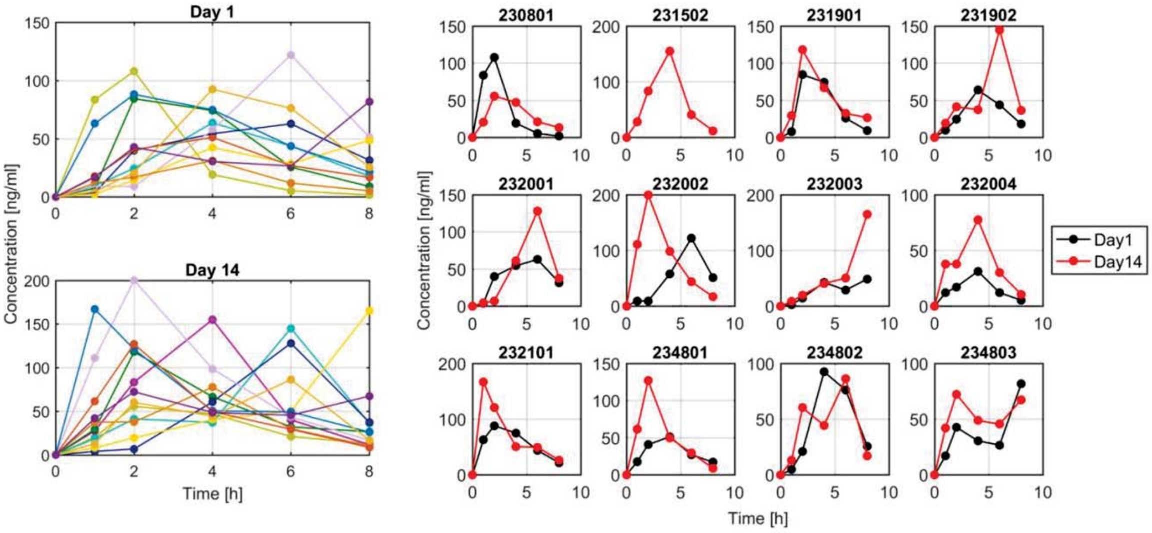


**Figure 8c. 2.0 mg/kg/day**

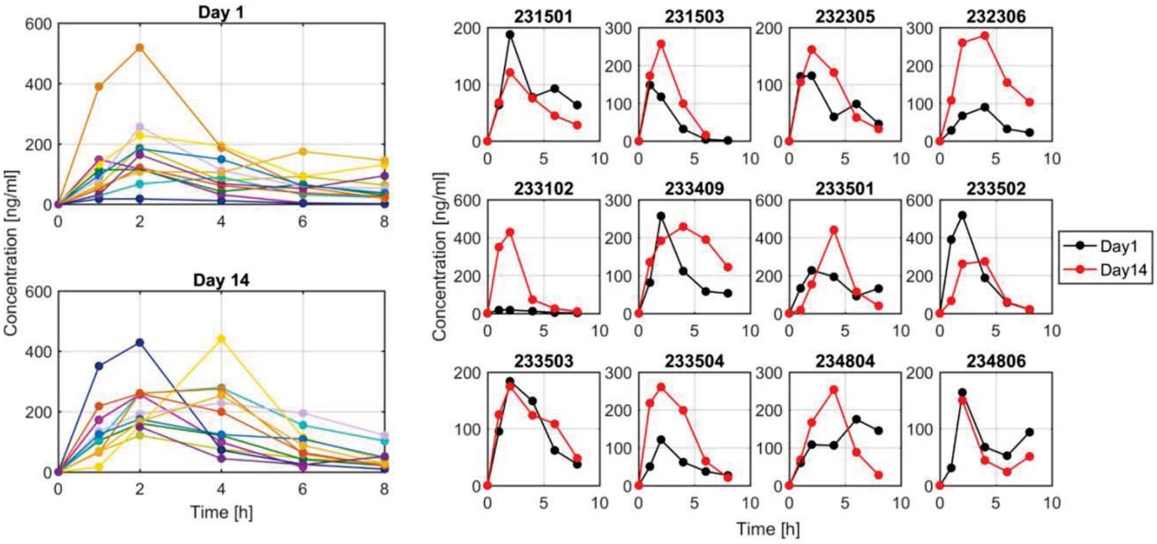


A comparison of the NCA PK parameters for the data from Days 1 and 14 (DMD boys) and a single dose in six healthy adult men is provided in **Table 5**. As noted in **Section 1.3.1.2**, the PK parameters of vamorolone in healthy men did not change from Day 1 to Day 14. It should be noted that the clearance values (CL) in **Table 5** are apparent since the bioavailability of the drug in these participants is uncertain.

**Table 5. Comparison of PK Parameters in DMD Boys Receiving Vamorolone 0.25 mg/kg/day, 0.75 mg/kg/day, or 2.0 mg/kg/day on Days 1 and 14, and in Six Healthy Adult Men (Single Dose) Receiving Vamorolone 0.3 mg/kg/day, 1 mg/kg/day or 3 mg/kg/day**

| Dose [mg/kg] | Cmax [ng/ml] | Tmax [hr] | $AUC_{inf}$ [hr*ng/ml] | $t_{1/2}$ [hr] | CL [ml/hr/kg] |
|---|---|---|---|---|---|
| **Day-1- Mean (SD)** | | | | | |
| 0.25 | 23.0 (13.4) | 3.64 (1.15) | 117.9 (47.7) | 2.13 (0.85) | 2459 (897) |
| 0.75 | 75.8 (25.9) | 4.55 (2.10) | 379.2 (117.2) | 1.81 (0.39) | 2282 (1102) |
| 2 | 199.0 (111.4) | 2.45 (1.30) | 761.1 (351.9) | 1.95 (0.79) | 2696 (1282) |
| **Day-14- Mean (SD)** | | | | | |
| 0.25 | 32.2 (15.2) | 3.82 (1.80) | 163.7 (49.8) | 1.97 (0.96) | 1828 (919) |
| 0.75 | 124.7 (40.8) | 3.75 (2.08) | 543.6 (148.6) | 2.11 (0.80) | 1509 (464) |
| 2 | 252.5 (95.9) | 2.83 (0..99) | 1137.5 (466.7) | 1.93 (1.02) | 2047 (771) |
| **Comparison with Adults (Single-Dose)** | | | | | |
| 0.3 | 50.8 (16.5%) | 1.5 | 170 (16.5%) | 1.60 | 1760 (15.5%) |
| 1 | 153 (15.9%) | 3.0 | 695 (22.1%) | 1.80 | 1440 (22.1%) |
| 3 | 281 (36.9%) | 2.0 | 1488 (23.7%) | 2.41 | 2020 (23.7%) |

In DMD children, the $C_{max}$ and $AUC_{inf}$ values increase with increasing doses with moderate variability. Since fitted curves of $AUC_{inf}$ or $C_{max} = a(Dose)^b$ result in b values that are not significantly different than 1.0 (95% CI includes 1.0), the kinetics appear linear with dose. The higher dose (2 mg/kg/day) does not appear to produce saturation of metabolic pathways controlling drug elimination.

Subject 233102 in the 2.0 mg/kg/day group (**Figure 8c**) had a low $AUC_{inf}$ on Day 1 that led to a high calculated clearance (clearance = Dose/AUC). This low AUC may have been a result of either poor bioavailability or incomplete swallowing on Day 1, as the Day 14 data were reasonable. The PK values for subject 233102 on Day 1 were removed from the calculations of the means.

Comparison of the Day 14 PK parameters following multiple-dosing to Day 1 PK parameters showed somewhat larger $C_{max}$ and $AUC_{inf}$ values and lower clearances in the DMD boys on Day 14. This observation was not due to drug accumulation, as nearly all children showed return to baseline within the PK study period, consistent with the short half-life (2-3 hr) in both DMD boys and adult men. It remains to be determined if the

greater $C_{max}$ and $AUC_{inf}$ values and lower clearances in the DMD boys at Day 14 is clinically meaningful.

Comparison of the single dose PK parameters in the fasted adult men and the PK parameters in the DMD boys taking their doses with a glass of milk showed the following:

Overall, the adult men attain higher $C_{max}$ and lower $T_{max}$ values than the DMD boys, indicating greater and faster absorption in the men. Food (milk) consumption in the boys as well as differences in physiology owing to either age or DMD may be possible explanations for this observation.

The boys have clearances (mL/hr/kg) that are similar to or greater than those of adults. This observation may in part be due to the DMD boys having somewhat greater clearances than would be expected from allometry, i.e., a higher body weight is usually associated with a greater clearance. In addition, vamorolone is highly metabolized and the enzyme concentration relative to body weight is greater in boys compared to adults.

Different doses impact the PK parameters similarly between boys and adults. The pharmacokinetics are essentially linear with dose, and changes during multiple-dosing are modest.

***Revised Text***

### *1.3.2 Pharmacokinetics in Phase IIa Study in 4 to 7 years Duchenne Muscular Dystrophy Boys (VBP15-002)*

Forty-eight boys with DMD received oral doses of vamorolone once daily for 14 days at doses of 0.25 mg/kg/day, 0.75 mg/kg/day, 2.0 mg/kg/day, and 6.0 mg/kg/day, 12 subjects receiving vamorolone at each dose level. The drug was administered with a glass of whole milk or other high-fat food. Six blood samples were collected on Day 1 and Day 14 at 0, 1, 2, 4, 6, and 8 hours post-dose and plasma was analyzed for vamorolone. The time-course of plasma drug concentrations was assessed using noncompartmental analytical (NCA) methods using the WinNonlin software (Certara).

Of the 48 DMD subjects enrolled in the study (12 at each dosing level), 47 had evaluable PK data. The average Day 1 and Day 14 vamorolone concentration versus time profiles are shown in **Figure 8** for the four dose levels. Overall, as expected, higher doses resulted in higher plasma concentrations of vamorolone. In particular, plasma vamorolone concentrations versus time showed a rapid to moderate rate of absorption, a maximum concentration ($C_{max}$) at a $T_{max}$ of 2 to 4 hours, and a decline phase with a typical half-life ($t_{1/2}$) of 2 hours. For all 4 doses, the mean PK profiles on Day 1 and Day14 appear similar. Many individual subjects had closely matching Day 1 and Day 14 profiles, but several did not. There was no accumulation of drug owing to the relatively short half-life and once-daily (morning) dosing.

**Figure 8 Mean Plasma Concentrations of Vamorolone after Once Daily Oral Administration of 0.25, 0.75, 2.0, and 6.0 mg/kg Doses**

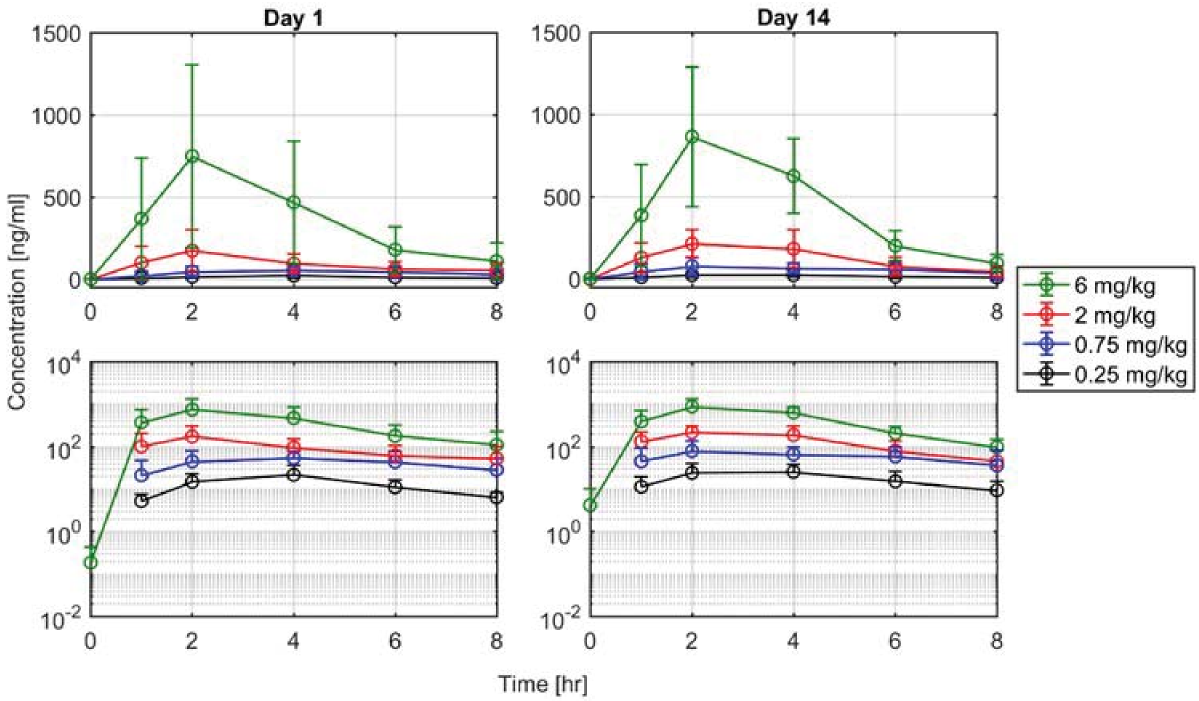


Vamorolone concentrations shown as mean ± standard deviation on linear (top panel) and semi-logarithmic (bottom panel) graphs.

A summary of the observed ($C_{max}$ and $T_{max}$) and calculated (AUC, $t_{1/2}$, and CL) PK parameters for all doses on Day 1 and Day 14 is provided in **Table 5**. Even with only 6 blood samples collected from each subject, the PK profiles adequately capture the overall exposures, but must be considered approximate. This particularly applies to the peak

values and the half-lives. Nevertheless, there is good consistency in the data with moderate variability as SD values are reasonable and the coefficient of variation values are typically less than 50%.

The drug is absorbed at a moderate rate with peak concentrations typically occurring at 2 to 4 hours on both Days 1 and 14. The median $T_{max}$ across all doses is typically 2 or 4 hours. The half-lives were similar on both days, averaging about 2 hours, although some subjects had irregular profiles without a clear decline phase. The apparent clearance (CL and CL/F) values were consistent across the dose levels for Day 1 as well as for Day 14, but the latter tended to average slightly lower (about 2400 mL/hr/kg on Day 1 and 1900 mL/hr/kg on Day 14).

**Table 5. Summary of Pharmacokinetic Parameters for Vamorolone after Once Daily Oral Administration of 0.25, 0.75, 2.0 and 6.0 mg/kg Doses to DMD Boys**

| | Day 1 | | | | Day 14 | | | |
|---|---|---|---|---|---|---|---|---|
| | Dose, mg/kg/day | | | | Dose, mg/kg/day | | | |
| Parameters | **0.25** | **0.75** | **2.0** | **6.0** | **0.25** | **0.75** | **2.0** | **6.0** |
| Cmax [ng/ml] | 22.9 (13.4) | 75.9 (25.9) | 199 (111) | 855.6 (471) | 32.2 (15.2) | 124.7 (42.5) | 252.5 (96) | 970 (270) |
| $T_{max}$ [h] | 3.6 [4] (1.2) | 4.6 [4] (2.1) | 2.5 [2] (1.3) | 2.7 [2] (1.3) | 3.8 [4] (1.8) | 3.8 [4] (2.2) | 2.8 [2] (1) | 2.3 [2] (0.86) |
| $AUC_{inf}$ [hr·ng/ml] | 118 (48) | 379 (117) | 761 (352) | 3279 (1693) | 164 (61) | 544 (155) | 1138 (467) | 3606 (897) |
| $t_{1/2}$ [h] | 2.1 (0.85) | 1.8 (0.43) | 1.9 (0.79) | 1.9 (0.95) | 1.9 (0.96) | 2.1 (0.8) | 1.9 (1.02) | 1.4 (0.35) |
| CL [ml/hr/kg] | 2459 (897) | 2285 (1103) | 2697 (1285) | 2320 (1375) | 1828 (919) | 1509 (482) | 2047 (771) | 1777 (476) |

Values are mean (standard deviation) and for $T_{max}$, which are shown as mean [median] (standard deviation).

An assessment of the linearity of the PKwith dose using $C_{max}$ and AUC is shown in **Figure 9**. Data for all individual subjects were used which also depict the variability for each dose level. While larger doses appear to present greater variability, this is a visual size distortion as the standard deviation is similar at each dose level (**Table 5**). Fitting the power equation of the form ($C_{max}$ , $AUC_{inf}$ = a x·$Dose^b$) resulted in 95% confidence intervals for *b* values that include 1.0 for $C_{max}$ and $AUC_{inf}$ for both Days 1 and 14 indicative of linear relationships across this range of dose levels.

**Figure 9 Regression Analysis of $C_{max}$ and $AUC_{inf}$ versus Dose**

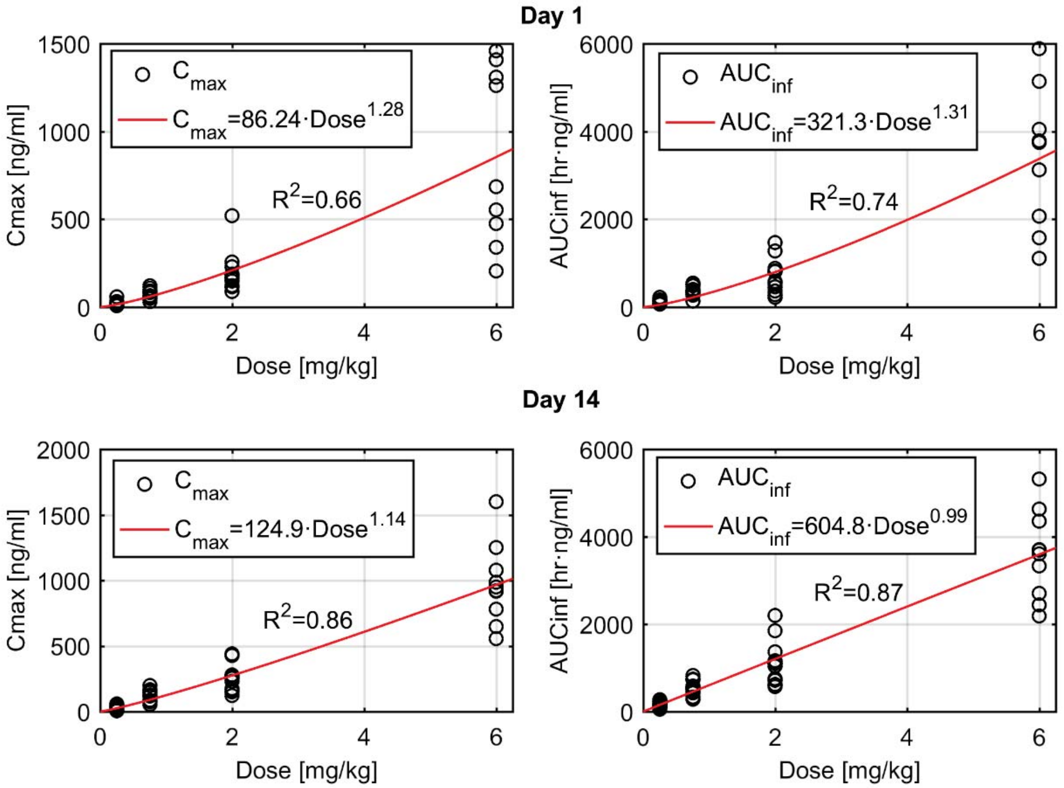


Regression was performed by fitting the indicated power equations to the data for Day 1 and Day 14. The fitted parameters are shown in the figure boxes.

Compared with fasting healthy adult volunteers (**Section 1.3.1**), the boys with DMD showed lower $C_{max}$ values and more variation in the time to reach the maximal concentration of vamorolone for any given dose. This observation might be due to the fact that the boys with DMD received vamorolone after 8 ounces of whole milk or other high fat food and a so-called food effect might have played a role. The apparent clearance of vamorolone seems higher in the boys with DMD but this finding is not significant.

**Section Changed:**

**Section 1.3.3: Safety in Phase II Studies in 4 to7 years Duchenne Muscular Dystrophy Boys (VBP15-002 and VBP15-003)**

***Original Text***

None

*Revised Text*

### *1.3.3 Safety in Phase II Studies in 4 to7 years Duchenne Muscular Dystrophy Boys (VBP15-002 and VBP15-003)*

*Adverse events:* There were no serious adverse events (SAEs) reported over the 14-day treatment in the Phase I clinical trial in healthy adult volunteers, nor in the four cohorts (0.25 mg/kg, 0.75 mg/kg, 2.0 mg/kg, and 6.0 mg/kg) of the Phase IIa study (VBP15-002; 14-day treatment) in boys ages 4 to <7 years with DMD. There were a total of 4 SAEs in the Phase II VBP15-003 study and three SAEs to date in the VBP15-LTE extension study: two SAEs of pneumonia in two different subjects (both subjects receiving vamorolone 0.75 mg/kg/day), one SAE of bilateral testicular torsion and one SAE of hypoxia in the same subject receiving 6.0 mg/kg/day, one SAE of influenza associated dehydration in a subject receiving 6.0 mg/kg/day, and two SAEs of acute myoglobinuria in the same subject receiving 6.0 mg/kg/day. Each of these SAEs was considered unrelated to study drug, and none of them resulted in discontinuation from the study. In the VBP15-003 study, there were a total of 218 TEAEs among 42 of the 48 subjects (87.5%). In VBP15-003 study, the TEAEs with the highest incidence were viral upper respiratory tract infection (41.7%); pyrexia (35.4%); cough (18.8%); vomiting (14.6%); and diarrhea (10.4%).

*Body Mass Index (BMI):* Body Mass Index was measured throughout the VBP15-003 study. The mean change from baseline to Week 24 for BMI was 0.03, 0.20, 0.23, and 1.15 for the 0.25, 0.75, 2.0, and 6.0 mg/kg/day dose level groups, respectively. Body Mass Index increases generally reflect an increase in weight. Body Mass Index z-score was monitored in the VBP15-003 study. The mean change from baseline to Week 24 for BMI z-score for the 6.0 mg/kg/day group showed a statistically significant increase compared to the mean change from baseline to Week 24 for the 0.25 mg/kg/day and 0.75 mg/kg/day dose level groups. In contrast, the mean change from baseline to Week 24 for the 2.0 mg/kg/day dose level group in BMI z-score was minimal and comparisons with the other vamorolone dose level groups lacked statistical significance. The mean increase from baseline to Week 24 for BMI z-score was similar for the 6.0 mg/kg/day group and a daily prednisone-treated historical control group.

*Potential liver toxicity:* In the Phase I clinical trial in adult volunteers, vamorolone showed mild elevations of liver enzymes in one subject receiving 20.0 mg/kg in the fasted state, and dosing was halted. All DMD subjects have elevated serum ALT and AST enzymes because of the muscle condition. For that reason, two enzymes, glutamate dehydrogenase (GLDH) and gamma glutamyl transferase (GTT), that are preferentially expressed in liver were evaluated in the VBP15-003 study. None of the mean changes in GLDH from baseline to any of the VBP15-003 on-treatment assessment time points were statistically significant for any dose level group. Although the mean changes from baseline to Week 8, Week 16, and Week 24 in GLDH levels did not show a dose response, shift analysis of GLDH levels did suggest a possible dose-related shift to higher GLDH levels at 2.0 mg/kg/day and 6.0 mg/kg/day at Weeks 16 and 24. Mean GGT levels and individual subject values at each VBP15-003 assessment time point across the four dose level groups remained at or below the normal range. On the basis of these mean GLDH and GGT data, vamorolone at dose levels up to 6.0 mg/kg/day does not appear to induce liver toxicity over a 24-week treatment period.

*Adrenal suppression:* In the VBP15-003 study, after 24 weeks of treatment, 0 of 8 tested participants (0.25 mg/kg/day), 1 of 12 (8.3%) tested participants (0.75 mg/kg/day), 5 of 12 (41.7%) tested participants (2.0 mg/kg/day), and 8 of 9 (88.9%) tested participants (6.0 mg/kg/day) had a depressed morning cortisol (<3.6 µg/dL [100 nM]) consistent with chronic adrenal suppression.[29]

*Insulin resistance:* In the VBP15-003 study, mean changes from baseline for fasting insulin showed dose-and time-related changes for all dose level groups at Week 12 and Week 24. Statistical significance was observed for mean increase from baseline for the 6.0 mg/kg/day dose level group at Week 12 and Week 24.

*Bone Turnover:* In the VBP15-003 study, pharmacodynamic biomarker testing for bone turnover markers suggested that vamorolone does not have the detrimental bone effects observed with prednisone and deflazacort.

**Section Changed:**

**Section 1.4: Rationale for Study Design, paragraph 5, sentence 4**

***Original Text***

Moreover, quality of life, neuropsychology, and satisfaction with treatment will be measured as exploratory efficacy outcomes using the parent proxy-report of Pediatric Outcomes Data Collection Instrument (PODCI), Child Behavior Checklist, PARS III, Treatment Satisfaction Questionnaire (TSQM), and Ease of Study Medication Administration Assessment for the study medication suspension, respectively.

***Revised Text***

Moreover, physical functioning, behavior, neuropsychology, and satisfaction with treatment will be measured as exploratory efficacy outcomes using the parent proxy-report of Pediatric Outcomes Data Collection Instrument (PODCI), PARS III, Treatment Satisfaction Questionnaire (TSQM), and Ease of Study Medication Administration Assessment for the study medication suspension, respectively.

**Section Changed:**

**Section 1.5: Overall Benefit/Risk**

***Original Text***

It is anticipated that the adverse effect profile of the investigational product will be more favorable than standard of care glucocorticoids in the long term. There were no serious adverse events (SAEs) reported over the 14-day treatment in the Phase I clinical trial in healthy adult volunteers, nor in the four cohorts (0.25 mg/kg, 0.75 mg/kg, 2.0 mg/kg, and 6.0 mg/kg) of the Phase IIa study (VBP15-002; 14-day treatment) in boys ages 4 to <7 years with DMD. There have been a total of 5 SAEs in the Phase IIa VBP15-003 and VBP15-LTE extension studies in DMD boys: two SAEs of pneumonia in two different subjects (both subjects receiving vamorolone 0.75 mg/kg/day), one SAE of bilateral testicular torsion and one SAE of hypoxia in the same subject receiving 6.0 mg/kg/day, and one SAE of influenza associated dehydration in a subject receiving 6.0 mg/kg/day. Each of these SAEs was considered unrelated to study drug, and none of them resulted in discontinuation from the study. In the Phase I clinical trial in adult volunteers, vamorolone showed mild elevations of liver enzymes in one subject receiving 20.0 mg/kg

in the fasted state. In the VBP15-002 study, after 2 weeks of treatment, 0 of 11 tested participants who received vamorolone 0.25 mg/kg/day, 0 of 11 tested participants who received vamorolone 0.75 mg/kg/day, 2 of 11 (18.2%) tested participants who received vamorolone 2 mg/kg/day, and 6 of 10 (60.0%) tested participants who received vamorolone 6 mg/kg/day had a depressed morning cortisol (<3.6 μg/dL [100 nmol/L]) consistent with chronic adrenal suppression. The long-term (24 weeks) safety profile of the same dose levels in boys ages 4 to 7 years with DMD is being explored in the Phase IIa extension study VBP15-003. Instructions for detecting adrenal crisis and the circumstances in which stress dose steroids should be provided will be included in the Informed Consent Form (ICF) and Manual of Operations, and Investigators should monitor clinical study participants closely to identify elevations in liver-specific enzymes.

Subjects may or may not receive direct health benefit from participating in the study. Some subjects will be randomly assigned to vamorolone at one of two planned dose levels (2.0 mg/kg/day and 6.0 mg/kg/day) over the course of the 48-week trial. While it is anticipated from nonclinical studies that these dose levels may be efficacious in the treatment of DMD, there are no clinical efficacy data yet available to validate this hypothesis. In view of the initial clinical evidence of safety and the nature of key nonclinical toxicological findings that can be monitored, the data support an acceptable risk profile for vamorolone.

***Revised Text***

It is anticipated that the adverse effect profile of the investigational product will be more favorable than standard of care glucocorticoids in the long term. There were no serious adverse events (SAEs) reported over the 14-day treatment in the Phase I clinical trial in healthy adult volunteers, nor in the four cohorts (0.25 mg/kg, 0.75 mg/kg, 2.0 mg/kg, and 6.0 mg/kg) of the Phase IIa study (VBP15-002; 14-day treatment) in boys ages 4 to <7 years with DMD. There have been a total of 7 SAEs in the Phase II VBP15-003 and VBP15-LTE extension studies in DMD boys: two SAEs of pneumonia in two different subjects (both subjects receiving vamorolone 0.75 mg/kg/day), one SAE of bilateral

testicular torsion and one SAE of hypoxia in the same subject receiving 6.0 mg/kg/day, one SAE of influenza associated dehydration in a subject receiving 6.0 mg/kg/day, and two SAEs of acute myoglobinuria in the same subject receiving 6.0 mg/kg/day. Each of these SAEs was considered unrelated to study drug, and none of them resulted in discontinuation from the study. In the Phase I clinical trial in adult volunteers, vamorolone showed mild elevations of liver enzymes in one subject receiving 20.0 mg/kg in the fasted state. On the basis of mean GLDH and GGT data, vamorolone at dose levels up to 6.0 mg/kg/day did not appear to induce liver toxicity over a 24-week treatment period in the VBP15-003 study. In the VBP15-002 study, after 2 weeks of treatment, 0 of 11 tested participants who received vamorolone 0.25 mg/kg/day, 0 of 11 tested participants who received vamorolone 0.75 mg/kg/day, 2 of 11 (18.2%) tested participants who received vamorolone 2 mg/kg/day, and 6 of 10 (60.0%) tested participants who received vamorolone 6 mg/kg/day had a depressed morning cortisol (<3.6 μg/dL [100 nmol/L]) consistent with chronic adrenal suppression. In the VBP15-003 study, after 24 weeks of treatment, 0 of 8 tested participants (0.25 mg/kg/day), 1 of 12 (8.3%) tested participants (0.75 mg/kg/day), 5 of 12 (41.7%) tested participants (2.0 mg/kg/day), and 8 of 9 (88.9%) tested participants (6.0 mg/kg/day) had a depressed morning cortisol (<3.6 μg/dL [100 nM]) consistent with chronic adrenal suppression. Instructions for detecting adrenal crisis and the circumstances in which stress dose steroids should be provided will be included in the Informed Consent Form (ICF) and Manual of Operations, and Investigators should monitor clinical study participants closely to identify elevations in liver-specific enzymes.

*Potential Health Benefits:* Subjects may or may not receive direct health benefit from participating in the study. Some subjects will be randomly assigned to vamorolone at one of two planned dose levels (2.0 mg/kg/day and 6.0 mg/kg/day) over the course of the 48-week trial. In the VBP15-003 study, clinical efficacy was assessed by Timed Function Tests. Improvement in Time to Stand, Time to Climb, Time to Run/Walk 10 Meters, and 6-Minute Walk Test were seen predominantly for the 2.0 and 6.0 mg/kg/day dose level groups, with many of the improvements showing statistical

significance compared to an untreated Duchenne Natural History Group. In view of the initial clinical evidence of safety, the improvements observed in the assessments of efficacy, and the nature of potential adverse effects that can be monitored, the data support an acceptable benefit/risk profile for vamorolone.

**Section Changed:**

**Section 2.2.1: Safety Endpoints**

***Original Text***

9. Dual-energy x-ray absorptiometry (DXA) scan: Change from baseline to Week 24 and Week 48 in BMD z-score;

***Revised Text***

9. Dual-energy x-ray absorptiometry (DXA) scan: Change from baseline to Week 24 and Week 48 in spine BMD, spine BMD z-score, total body BMD, spine and total body bone mass, and total body composition (lean mass, fat mass, fat-free mass, Lean Mass Index, and Fat Mass Index);

**Section Changed:**

**Section 2.2.3: Exploratory Endpoints**

***Original Text***

1. Treatment satisfaction questionnaire (TSQM): Comparison of each vamorolone dose level group to the prednisone group at the Week 24 visit; comparison of each treatment group at the Week 48 visit;
2. Pediatric Outcomes Data Collection Instrument (PODCI): Comparison of each vamorolone dose level group to the prednisone group for change from baseline to the Week 24 assessment; comparison of each treatment group for change from baseline to the Week 48 assessment;
3. Behavioral changes (Child Behavior Checklist and PARS III): Comparison of each vamorolone dose level group to the prednisone group and to the placebo group for change from baseline to each of the scheduled study assessment time

points up to the Week 24 assessment; comparison of each treatment group for change from baseline to the Week 48 assessment;

4. Ease of study medication administration assessed at each of the scheduled study assessment time points; and
5. Blindedness Assessment at each of the scheduled study assessment time points

***Revised Text***

1. Treatment satisfaction questionnaire (TSQM): Comparison of each vamorolone dose level group to the prednisone group at the Week 24 visit; comparison of each treatment group at the Week 48 visit;
2. Pediatric Outcomes Data Collection Instrument (PODCI): Comparison of each vamorolone dose level group to the placebo group for change from baseline to the Week 24 assessment; comparison of each treatment group for change from baseline to the Week 48 assessment;
3. Behavioral changes (PARS III): Comparison of each vamorolone dose level group to the prednisone group for change from baseline to each of the scheduled study assessment time points up to the Week 24 assessment; comparison of each treatment group for change from baseline to the Week 48 assessment;
4. Ease of study medication administration assessed at each of the scheduled study assessment time points;
5. Blindedness Assessment at each of the scheduled study assessment time points; and
6. DNA testing for candidate genetic modifiers of DMD.

**Section Changed:**

**Section 3.2: Randomization, paragraph 2**

***Original Text***

When the site investigator/designee completes the randomization procedures via the IXRS system, an e-mail report with the randomization number and age stratification

group will be generated and sent to the site investigator and to the clinical trials supply company confirming randomization into the trial.

***Revised Text***

When the site investigator/designee completes the randomization procedures via the IXRS system, an e-mail report with the randomization number and age stratification group will be generated and sent to the clinical trials supply company confirming randomization into the trial.

**Section Changed:**

**Section 3.4: Unblinding, paragraph 6**

***Original Text***

After breaking the blind, the site staff should record details regarding the reasons for breaking the blind, including any AEs leading to the unblinding, in the source documents and electronic case report form (eCRF). Once the blind is broken for a given subject, study drug will be discontinued and the subject will be withdrawn from the study. Furthermore, as the subject might have received glucocorticoids (prednisone) as part of the study and as the effect of vamorolone on the adrenal axis remains unknown, prednisone and vamorolone cannot be discontinued without a proper dose-tapering period.

***Revised Text***

After breaking the blind, the site staff should record details regarding the reasons for breaking the blind, including any AEs leading to the unblinding, in the source documents and electronic case report form (eCRF). Once the blind is broken for a given subject, study drug will be discontinued and the subject will be withdrawn from the study. Furthermore, as the subject might have received glucocorticoids (prednisone) as part of the study and as vamorolone appears to affect the adrenal axis, prednisone and vamorolone cannot be discontinued without a proper dose-tapering period.

**Section Changed:**

**Section 5.3: Dosage Schedule and Administration of Study Medication, paragraph 7**

***Original Text***

Vamorolone or matching placebo suspension will be administered orally using a volumetric syringe supplied by the site. Following administration of the dose of study drug, the syringe will be filled once with water and the water will be administered by mouth using the volumetric syringe. Prednisone or matching placebo tablets will be taken right after the dose of suspension. The subject will then drink approximately 50 mL (approximately 2 ounces) of water to ensure the full dose has been ingested. The dose of study medication should be taken with breakfast, including at least 8 g of fat (approximately 8 ounces [240 mL] of full-fat milk or equivalent high-fat food portion). There are no other food or drink restrictions before or after dosing.

***Revised Text***

Vamorolone or matching placebo suspension will be administered orally using a volumetric syringe supplied by the site. Following administration of the dose of study drug suspension, the syringe will be filled once with water and the water will be administered by mouth using the volumetric syringe. Prednisone or matching placebo tablets will be taken either immediately before or immediately after the dose of suspension. The subject will then drink approximately 50 mL (approximately 2 ounces) of water to ensure the full dose has been ingested. The dose of study medication should be taken with breakfast, including at least 8 g of fat (approximately 8 ounces [240 mL] of full-fat milk or equivalent high-fat food portion). There are no other food or drink restrictions before or after dosing.

**Section Changed:**

**Section 5.4: Rationale for Dose Selection, paragraph 5**

***Original Text***

Vamorolone at doses of 0.25 mg/kg/day, 0.75 mg/kg/day, 2.0 mg/kg/day and 6.0 mg/kg/day has been demonstrated to be safe and well-tolerated in a 2-week Phase IIa study (VBP15-002) in 4 to <7 years DMD boys. The safety of these four doses continues to be studied in the 24-week Phase IIa extension study (VBP15-003) in 4 to 7 year-old

DMD boys. One SAE of pneumonia was reported for one subject taking 0.75 mg/kg in the VBP15-003 study, and was assessed by both the Investigator and Medical Monitor to be at most remotely related to study drug, and resolved without interruption of study drug administration. One subject receiving vamorolone 6 mg/kg in the Phase IIa extension study (VBP15-003) who had an incidental early morning cortisol drawn following an AE of presyncope had evidence of adrenal suppression. Thus, based on the available data in the Phase I and Phase IIa studies regarding the safety signal of suppression of the adrenal axis, no adrenal suppression is anticipated at the planned 2.0 mg/kg/day dose, but the possibility of adrenal suppression is present in subjects at the 6.0 mg/kg/day dose level.

***Revised Text***

Vamorolone at doses of 0.25 mg/kg/day, 0.75 mg/kg/day, 2.0 mg/kg/day and 6.0 mg/kg/day has been demonstrated to be safe and well-tolerated in a 2-week Phase IIa study (VBP15-002) in 4 to <7 years DMD boys. The safety of these four doses continued to be studied in the 24-week Phase II extension study (VBP15-003) in 4 to 7 year-old DMD boys, and continues to be studied in the ongoing VBP15-LTE long-term extension study. There were a total of 4 SAEs in the Phase II VBP15-003 study and three SAEs to date in the VBP15-LTE extension study: two SAEs of pneumonia in two different subjects (both subjects receiving vamorolone 0.75 mg/kg/day), one SAE of bilateral testicular torsion and one SAE of hypoxia in the same subject receiving 6.0 mg/kg/day, one SAE of influenza associated dehydration in a subject receiving 6.0 mg/kg/day, and two SAEs of acute myoglobinuria in the same subject receiving 6.0 mg/kg/day. Each of these SAEs was considered unrelated to study drug, and none of them resulted in discontinuation from the study. One subject receiving vamorolone 6.0 mg/kg in the Phase II extension study (VBP15-003) who had an incidental early morning cortisol drawn following an AE of presyncope had evidence of adrenal suppression. In the VBP15-003 study, after 24 weeks of treatment, 0 of 8 tested participants (0.25 mg/kg/day), 1 of 12 (8.3%) tested participants (0.75 mg/kg/day), 5 of 12 (41.7%) tested participants (2.0 mg/kg/day), and 8 of 9 (88.9%) tested participants (6.0 mg/kg/day) had a depressed morning cortisol (<3.6 μg/dL [100 nM]) consistent with chronic adrenal suppression.[29] Thus, based on the available data in the Phase I and Phase II studies

regarding the safety signal of suppression of the adrenal axis, the possibility of adrenal suppression is present in subjects at the 2.0 and 6.0 mg/kg/day dose levels.

**Section Changed:**

**Section 5.7.3: Prohibited Therapies, paragraph 1, bullets #2 and 4**

***Original Text***

- Oral glucocorticoids or other immunosuppressive agents. Subjects who have received any prior treatment with immunosuppressive agents are ineligible for study entry. [Notes: Inhaled and/or topical glucocorticoids prescribed for an indication other than DMD are permitted but must be administered at stable dose beginning at least 4 weeks prior to first dose of study medication, and are anticipated to be used at the stable dose regimen for the duration of the study; past transient use of oral or inhaled glucocorticoids or other oral immunosuppressive agents for indication other than DMD for no longer than 3 months cumulative, with last use at least 3 months (or last use at least one month prior for inhaled glucocorticoids) prior to first dose of study medication, will be considered for eligibility on a case-by-case basis.]
- Live attenuated vaccines (use must be avoided for the duration of participation in the study)

***Revised Text***

- Oral glucocorticoids or other immunosuppressive agents. Subjects who have received prior treatment with immunosuppressive agents are ineligible for study entry. [Notes: Inhaled and/or topical glucocorticoids are permitted but must be administered at stable dose beginning at least 4 weeks prior to first dose of study medication, and are anticipated to be used at the stable dose regimen for the duration of the study; past transient use of oral or inhaled glucocorticoids or other oral immunosuppressive agents for no longer than 1 month cumulative, with last use at least 3 months (or last use at least one month prior for inhaled

glucocorticoids) prior to first dose of study medication, will be considered for eligibility on a case-by-case basis.]

- Live attenuated vaccines: use must be avoided within 14 days prior to first dose of study medication and for the duration of participation in the study

**Section Changed:**

**Section 5.7.5: Hydrocortisone**

***Original Text***

All subjects will be given a single dose of hydrocortisone (5 mg or 10 mg) at home, 24 hours after the final dose of Treatment Period #1 study medication at the Week 24 Visit, and 24 hours after the final dose of Treatment Period #2 study medication at the Week 48 Visit. The hydrocortisone dose will be approximately 8 mg/m$^2$, rounded up to either 5 mg or 10 mg; subjects will be provided with either a single 5 mg or 10 mg hydrocortisone tablet which will be dispensed by the site staff at the Week 24 and Week 48 Visits.

In addition, subjects with evidence of adrenal suppression (cortisol levels <18 µg/dL [or 500 nM]) 30 or 60 minutes after stimulation with Cosyntropin [250 µg]) or with suspected adrenal suppression should be covered with "stress dosing" of hydrocortisone (or prednisone) during times of illness, injury, or surgery (see **Section 7.2.7**).

***Revised Text***

All subjects will be given a single dose of hydrocortisone (5 mg or 10 mg) 24 hours after the final dose of Treatment Period #1 study medication at the Week 24 Visit, and 24 hours after the final dose of Treatment Period #2 study medication at the Week 48 Visit. The hydrocortisone dose will be approximately 8 mg/m$^2$, rounded up to either 5 mg or 10 mg; subjects will be provided with either a single 5 mg or 10 mg hydrocortisone tablet which will be dispensed by the site staff at the Week 24 and Week 48 Visits.

In addition, all subjects should be covered with "stress dosing" of hydrocortisone (or prednisone) during times of illness, injury, or surgery (see **Section 7.2.7**).

**Section Changed:**

**Section 5.8.3: Study Medication Shipping and Handling, paragraph 4**

***Original Text***

It is essential to this study that all CTM be accounted for during the study period. All unused (i.e., undispensed; dispensed and returned) study medication will be retained at the study site for reconciliation by the Sponsor's study monitors (or designees) during routine monitoring visits. Final disposition of all unused CTM will be coordinated by the Sponsor's study monitors (or designees) at the end of the study (see **Section 5.8.4**).

***Revised Text***

It is essential to this study that all CTM be accounted for during the study period. All unused (i.e., undispensed; dispensed and returned) study medication will be retained at the study site for reconciliation by the Sponsor's study monitors (or designees) during routine monitoring visits. Final disposition of all unused CTM will be coordinated by the Sponsor's study monitors (or designees) throughout and at the end of the study (see **Section 5.8.4**).

**Section Changed:**

**Section 6.1: Time and Events Schedule, paragraphs 3, 4, and 5**

***Original Text***

Overall, up to approximately 57 weeks are allocated for each subject to complete the study, including a 32-day Pretreatment Screening Period, a one-day Pretreatment Baseline Period, a 24-week Treatment Period #1, a 4-week Transition Period, a 20-week Treatment Period #2, plus a 4-week Dose-Tapering Period, as applicable. Upon the completion of the study, subjects will have the option to enroll in the Phase IIb long-term extension study.

**Optional 24-month long-term extension study:** Subjects electing to enroll directly into the subsequent extension protocol to continue vamorolone therapy will be discharged from the VBP15-004 study following completion of all final Week 48 assessments and ACTH Stimulation testing at the Week 48 Follow-up Visit, and will be enrolled in the long-term extension study (separate written protocol and ICF).

Subjects completing the VBP15-004 study and enrolling directly into the long-term extension study do not need to dose taper in VBP15-004.

***Revised Text***

Overall, up to approximately 57 weeks are allocated for each subject to complete the study, including a 32-day Pretreatment Screening Period, a one-day Pretreatment Baseline Period, a 24-week Treatment Period #1, a 4-week Transition Period, a 20-week Treatment Period #2, plus a 4-week Dose-Tapering Period, as applicable. Upon the completion of the study, subjects may have the option to enroll in an additional vamorolone study or general access program.

Subjects electing to enroll directly into an additional vamorolone study or general access program to continue vamorolone therapy will be discharged from the VBP15-004 study following completion of all final Week 48 assessments and ACTH Stimulation testing at the Week 48 Follow-up Visit, and will be enrolled in the additional vamorolone study or general access program (separate written protocol and ICF).

Subjects completing the VBP15-004 study and enrolling directly into the additional vamorolone study or general access program to continue vamorolone treatment do not need to dose taper in VBP15-004

**<u>Section Changed:</u>**

**<u>Section 6.1: Time and Events Schedule, Table 11, Schedule of Study Activities</u>**

***Original Text:***

**Table 11 Schedule of Study Activities**

| | Pretreatment Period | | Treatment Period #1 | | | | | | | Transition Period | | Treatment Period #2 | | | | | | Dose-tapering Period | |
|---|---|---|---|---|---|---|---|---|---|---|---|---|---|---|---|---|---|---|---|
| | SCR | BL | | | | | | | | | | | | | | | | | |
| | Day | | | Week | | | | | | | | | | | | | | | |
| **Study Day or Week/Visit** | **-33 to -2[a]** | **-1[b]** | **1[c]** | **2 (±1d)** | **6 (±3d)** | **12 (±1w)** | **18 (±1w)** | **24[d] (±1w)** | **24 (F/U)[d]** | **26** | **28[e] (±1d)** | **28+1d** | **30 (±1d)** | **34 (±3d)** | **40 (±1w)** | **48[f] (±1w)** | **48 F/U[f]** | **50** | **52[g] (±1d)** |
| Informed consent | X | | | | | | | | | | | | | | | | | | |
| Enrollment[h] | X | | | | | | | | | | | | | | | | | | |
| Inclusion/exclusion criteria | X | | | | | | | | | | | | | | | | | | |
| Randomization[i] | X | | | | | | | | | | | | | | | | | | |
| Demographics | X | | | | | | | | | | | | | | | | | | |
| Medical history | X | | | | | | | | | | | | | | | | | | |
| Medication history | X | X | | | | | | | | | | | | | | | | | |
| Physical examination | X | X | | | X | X | X | X | | | X | | | X | X | X | | | X |
| Cushingoid features | | X | | | X | X | X | X | | | X | | | X | X | X | | | X |
| Height | X | | | | | X | | X | | | | | | X | | X | | | |
| Weight | X | X | | X | X | X | X[j] | X | | | X | | X | X | X[j] | X | | | X |
| Vital signs[k] | X | X | X[l] | X | X | X | X | X | | | X | | X | X | X | X | | | X |
| Blood for clinical labs[m] | X | | X[n] | X[n] | X | X[n] | X | X[n] | | | X[n] | | X[n] | X | X[n] | X[n] | | | X[n] |
| Blood for HbA1c[m] | X | | | | | | | X | | | | | | | | X | | | |
| Blood for vitamin D[m] | X | | | | | X | | X | | | | | | | X | X | | | |
| Blood for chicken pox IgG | X | | | | | | | | | | | | | | | | | | |
| Urinalysis[o] | X | | X[n] | X[n] | X | X[n] | X | X[n] | | | X[n] | | X[n] | X | X[n] | X[n] | | | X[n] |
| Blood for serum PD biomarker panel[p,q] | | | X | | | X | | X | | | X | | | | X | X | | | X |
| Fasting blood for insulin, glucose[q] | | | X | | | X | | X | | | X | | | | X | X | | | X |
| ACTH Stimulation Test | X | | | | | | | | X[r] | | | | | | | | X[r] | | |
| Blood for Plasma PK | | | | | | | | | | | | | X[s] | | | | | | |
| 12-lead ECG[t] | X | | | | | X | | X | | | | | | | X | X | | | |
| 2D-echocardiogram | X | | | | | | | X | | | | | | | | X | | | |

| | **Pretreatment Period** | | **Treatment Period #1** | | | | | | | **Transition Period** | | **Treatment Period #2** | | | | | | **Dose-tapering Period** | |
|---|---|---|---|---|---|---|---|---|---|---|---|---|---|---|---|---|---|---|---|
| | **SCR** | **BL** | | | | | | | | | | | | | | | | | |
| | **Day** | | | **Week** | | | | | | | | | | | | | | | |
| **Study Day or Week/Visit** | **-33 to -2[a]** | **-1[b]** | **1[c]** | **2 (±1d)** | **6 (±3d)** | **12 (±1w)** | **18 (±1w)** | **24[d] (±1w)** | **24 (F/U)[d]** | **26** | **28[e] (±1d)** | **28+1d** | **30 (±1d)** | **34 (±3d)** | **40 (±1w)** | **48[f] (±1w)** | **48 F/U[f]** | **50** | **52[g] (±1d)** |
| Eye examination | X | | | | | | | X | | | | | | | | X | | | |
| DXA scan | X | | | | | | | X | | | | | | | | X | | | |
| Spine X-ray | X | | | | | | | X | | | | | | | | | | | |
| Fracture Questionnaire | X | | | | | | | X | | | | | | | | X | | | |
| Dispense study medication | | | X | | X | X | X | | X | | X | | | X | X | | X | | |
| Return study medication/ compliance monitoring | | | | X[u] | X | X | X | X | | | X | | X[u] | X | X | X | | | X |
| Study medication dosing[v] | | | X → | | | | | X | | | | X → | | | | X | | | |
| Study medication dose tapering | | | | | | | | | X[w] → | | X | | | | | | X → | | X |
| Telephone call to subject[x] | | | | | | | | | | X | | | | | | | | X | |
| Time to Stand Test (TTSTAND) | X | X | | | X | X | | X | | | | | | X | X | X | | | |
| Time to Climb Test (TTCLIMB) | X | X | | | | X | | X | | | | | | | X | X | | | |
| Time to Run/Walk Test (TTRW) | X | X | | | | X | | X | | | | | | | X | X | | | |
| NSAA[y] | X | X | | | | X | | X | | | | | | | X | X | | | |
| Myometry (elbow flexors, knee extensors) | X | X | | | | X | | X | | | | | | | X | X | | | |
| Six-minute Walk Test (6MWT) | X | X | | | | X | | X | | | | | | | X | X | | | |
| Range of Motion (ROM) - ankles | X | X | | | | X | | X | | | | | | | X | X | | | |
| Pediatric Outcomes Data Collection Instrument (PODCI) | X | | | | | | | X | | | | | | | | X | | | |
| Treatment Satisfaction Questionnaire (TSQM) | | | | | | | | X | | | | | | | | X | | | |

| | Pretreatment Period | | Treatment Period #1 | | | | | | | Transition Period | | Treatment Period #2 | | | | | | Dose-tapering Period | |
|---|---|---|---|---|---|---|---|---|---|---|---|---|---|---|---|---|---|---|---|
| | SCR | BL | | | | | | | | | | | | | | | | | |
| | Day | | | Week | | | | | | | | | | | | | | | |
| **Study Day or Week/Visit** | **-33 to -2[a]** | **-1[b]** | **1[c]** | **2 (±1d)** | **6 (±3d)** | **12 (±1w)** | **18 (±1w)** | **24[d] (±1w)** | **24 (F/U)[d]** | **26** | **28[e] (±1d)** | **28+1d** | **30 (±1d)** | **34 (±3d)** | **40 (±1w)** | **48[f] (±1w)** | **48 F/U[f]** | **50** | **52[g] (±1d)** |
| Child Behavior Checklist | X | | | | | X | | X | | | | | | | | X | | | |
| PARS III | X | | | | | X | | X | | | | | | | | X | | | |
| Ease of Study Medication Administration Assessment[z] | | | | X | | X | | X | | | | | X | | X | X | | | |
| Blindedness Assessment | | | | | | | | X | | | | | | | | | | | |
| Dispense subject diaries[aa] | | | X | X | X | X | X | X | | | X | | X | X | X | X | | | |
| Return subject diaries | | | | X | X | X | X | X | | | X | | X | X | X | X | | | X |
| AE/SAE recording[bb] | X → | | | | | | | | | | | | | | | | | | → X[cc] |
| Concomitant medications | | | X → | | | | | | | | | | | | | | | | → X |
| Discharge from study | | | | | | | | | | | | | | | | | X[dd] | | X[ee] |

BL = Baseline; d = day(s); F/U = Follow-up; SCR = Screening; w = week.

a. The Pretreatment Screening Period spans Day -33 through Day -2, but all screening procedures must be completed by Day -11. Subjects meeting all eligibility criteria will be randomized by Day -11, at least 10 days prior to the Baseline Day -1 Visit.

b. Baseline Day -1, within 24 hours prior to administration of the first dose of study drug.

c. Treatment Day 1 begins at the time of administration of the first dose of study medication in the clinic.

d. Subjects who prematurely discontinue from the study prior to Week 24 should complete the Week 24 assessments and the Week 24 Follow-up Visit ACTH Stimulation Test at the time of early withdrawal and undergo Early Discontinuation Dose-tapering, where possible (see **Section 6.4.1** and **Section 7.2.7**). The Week 24 Follow-up Visit must occur to allow the ACTH Stimulation Test to be performed 48 ± 3 hours after the final dose of Treatment Period #1 study medication.

e. Subjects who prematurely discontinue from the study after Week 24 but prior to Week 28 should complete the Week 28 assessments, and undergo Early Discontinuation Dose-tapering, where possible (see **Section 6.4.2**).

f. Subjects who prematurely discontinue from the study after Week 28 but prior to Week 48 should complete the Week 48 assessments and the Week 48 Follow-up Visit ACTH Stimulation Test at the time of early withdrawal and undergo Dose-tapering, where possible (see **Section 6.4.3** and **Section 7.2.7**). The Week 48 Follow-up Visit must occur to allow the ACTH Stimulation Test to be performed 48 ± 3 hours after the final dose of Treatment Period #2 study medication.

g. Subjects will have one study site visit during the Dose-tapering Period, at one week after the dose of liquid formulation has been discontinued (Week 52) (see **Section 6.3.7**).

h. Subjects are considered to be enrolled in the study at the time written informed consent is obtained.

i. Randomization occurs by Interactive Voice/Web Response System (IXRS) after subjects are confirmed to have met all study entry criteria, at least 10 days prior to the Baseline Day -1 Visit.

j. Weight recorded at the Week 18 Visit and the Week 40 Visit will be used to calculate doses for study drug dispensed at the Week 24 Follow-up and Week 48 Follow-up Visits, respectively.
k. Sitting blood pressure, body temperature, respiratory rate, and heart rate.
l. Vital signs recorded prior to administration of the first dose of study drug at the Day 1 Visit.
m. Blood for hematology, chemistry, and lipids, including HbA1c and Vitamin D where applicable.
n. Blood samples (collected after subjects have fasted for ≥ 6 hours) and urine collected at scheduled visit, and prior to dose of study drug where applicable.
o. Urinalysis by dipstick and microscopic analysis.
p. Blood collected for PD biomarkers includes secondary safety outcomes (morning cortisol, osteocalcin, CTX1, P1NP), and exploratory safety and efficacy PD biomarkers.
q. Blood samples for PD biomarkers and fasting glucose and insulin determination will be collected after subjects have fasted for ≥ 6 hours, prior to the daily dose of study medication where applicable.
r. Subjects will return to the study site for the Week 24 Follow-up Visit for an ACTH Stimulation Test 48 hours ± 3 hours after administration of the final dose of Treatment Period #1 study medication, and for the Week 48 Follow-up Visit for an ACTH Stimulation Test 48 hours ± 3 hours after administration of the final dose of Treatment Period #2 study medication (see **Section 7.2.7**).
s. Blood sample for population PK analysis will be collected 2 hours after administration of the daily dose of study medication.
t. 12-lead ECG recorded after subject has rested quietly in a supine position for at least 5 minutes.
u. Study medication brought by subjects to the Week 2 Visit and Week 30 Visit for dosing and compliance assessment will be redispensed to subjects at the end of the visit.
v. The dose of study medication on the days of the Day 1, Week 2, Week 12, Week 24, Week 28, Week 30, Week 40, and Week 48 Visits will be administered after 1) a fasting blood draw for insulin and glucose; and 2) breakfast provided by the study site. All other doses will be taken at home. See **Section 6.3.3** for other Day 1 pre-dose safety assessments.
w. Doses of tablet study drug will be tapered and suspension study drug will be continued, during Weeks 24-28.
x. Site study staff will contact the parent(s)/guardian(s) by telephone at Weeks 26 and 50 to ensure that the study drug tapering is proceeding according to protocol, to assess potential signs or symptoms indicative of adrenal suppression, and to address any questions the parent(s)/guardian(s) may have.
y. North Star Ambulatory Assessment; includes the Time to Stand Test (TTSTAND).
z. Ease of Study Medication Administration assessed at the Weeks 2, 12, and 24, 30, 40, and 48 Visits.
aa. Subject diaries used to record any changes in concomitant medications taken, any AEs experienced during the study, and any incomplete or missed doses of study medication.
bb. All AEs and SAEs must be recorded in the source documents and eCRF from the date of the subject's written informed consent until the final Week 52 Visit or the subject's participation in the study is completed (SAEs through 30 days after final study drug dose). Ongoing AEs will be followed to resolution, stabilization, or until such time the Investigator agrees follow-up is not necessary.
cc. For subjects who do not continue into the long-term extension study, site staff will make a phone call to the home 31-35 days after the final dose of study medication in VBP15-004 Dose-tapering Period to confirm the final SAE status of the subject.
dd. Subjects who elect to continue vamorolone therapy by enrolling directly into the long-term extension study may be discharged from the study following completion of all final Week 48 assessments, including the Week 48 Follow-up Visit ACTH Stimulation Test.
ee. Subjects who participate in the Dose-tapering Period may be discharged from the study following completion of all final Dose-Tapering Visits assessments (Week 52) (see **Section 6.3.7**).

***Revised Text***

**Table 11 Schedule of Study Activities**

| | **Pretreatment Period** | | **Treatment Period #1** | | | | | | | **Transition Period** | **Treatment Period #2** | | | | | | | **Dose-tapering Period** | |
|---|---|---|---|---|---|---|---|---|---|---|---|---|---|---|---|---|---|---|---|
| | **SCR** | **BL** | | | | | | | | | | | | | | | | | |
| | **Day** | | | **Week** | | | | | | | | | | | | | | | |
| **Study Day or Week/Visit** | **-33 to -2[a]** | **-1[b]** | **1[c]** | **2 (±1d)[d]** | **6 (±3d)[d]** | **12 (±1w)[d]** | **18 (±1w)[d]** | **24[e] (±1w)[d]** | **24 (F/U)[e]** | **26** | **28[f] (±1d)[d]** | **28+1d** | **30 (±1d)[d]** | **34 (±3d)[d]** | **40 (±1w)[d]** | **48[g] (±1w)[d]** | **48 F/U[g]** | **50** | **52[h] (±1d)[d]** |
| Informed consent | X | | | | | | | | | | | | | | | | | | |
| Enrollment[i] | X | | | | | | | | | | | | | | | | | | |
| Inclusion/exclusion criteria | X | X[j] | | | | | | | | | | | | | | | | | |
| Randomization[k] | X | | | | | | | | | | | | | | | | | | |
| Demographics | X | | | | | | | | | | | | | | | | | | |
| Medical history | X | | | | | | | | | | | | | | | | | | |
| Medication history | X | X | | | | | | | | | | | | | | | | | |
| Physical examination | X | X | | | X | X | X | X | | | X | | | X | X | X | | | X |
| Cushingoid features | | X | | | X | X | X | X | | | X | | | X | X | X | | | X |
| Height | X | | | | | X | | X | | | | | | X | | X | | | |
| Weight | X | X | | X | X | X | X[l] | X | | | X | | X | X | X[l] | X | | | X |
| Vital signs[m] | X | X | X[n] | X | X | X | X | X | | | X | | X | X | X | X | | | X |
| Blood for clinical labs[o] | X | | X[p] | X[p] | X | X[p] | X | X[p] | | | X[p] | | X[p] | X | X[p] | X[p] | | | X[p] |
| Blood for HbA1c[o] | X | | | | | | | X | | | | | | | | X | | | |
| Blood for vitamin D[o] | X | | | | | X | | X | | | | | | | X | X | | | |
| Blood for chicken pox IgG | X | | | | | | | | | | | | | | | | | | |
| Urinalysis[q] | X | | X[p] | X[p] | X | X[p] | X | X[p] | | | X[p] | | X[p] | X | X[p] | X[p] | | | X[p] |
| Blood for serum PD biomarker panel[r,s] | | | X | | | X | | X | | | X | | | | X | X | | | X |
| Fasting blood for insulin, glucose[s] | | | X | | | X | | X | | | X | | | | X | X | | | X |
| Blood for DNA Testing | | | | | | | | X | | | | | | | | | | | |
| ACTH Stimulation Test | X | | | | | | | | X[t] | | | | | | | | X[t] | | |
| Blood for Plasma PK | | | | | | | | | | | | | X[u] | | | | | | |
| 12-lead ECG[v] | X | | | | | X | | X | | | | | | | X | X | | | |
| 2D-echocardiogram | X | | | | | | | X | | | | | | | | X | | | |

| | **Pretreatment Period** | | **Treatment Period #1** | | | | | | | **Transition Period** | | **Treatment Period #2** | | | | | | **Dose-tapering Period** | |
|---|---|---|---|---|---|---|---|---|---|---|---|---|---|---|---|---|---|---|---|
| | **SCR** | **BL** | | | | | | | | | | | | | | | | | |
| | **Day** | | | **Week** | | | | | | | | | | | | | | | |
| **Study Day or Week/Visit** | **-33 to -2[a]** | **-1[b]** | **1[c]** | **2 (±1d)[d]** | **6 (±3d)[d]** | **12 (±1w)[d]** | **18 (±1w)[d]** | **24[e] (±1w)[d]** | **24 (F/U)[e]** | **26** | **28[f] (±1d)[d]** | **28+1d** | **30 (±1d)[d]** | **34 (±3d)[d]** | **40 (±1w)[d]** | **48[g] (±1w)[d]** | **48 F/U[g]** | **50** | **52[h] (±1d)[d]** |
| Eye examination | X | | | | | | | X | | | | | | | | X | | | |
| DXA scan | X | | | | | | | X | | | | | | | | X | | | |
| Spine X-ray | X | | | | | | | X | | | | | | | | | | | |
| Fracture Questionnaire | X | | | | | | | X | | | | | | | | X | | | |
| Dispense study medication | | | X | | X | X | X | | X | | X | | | X | X | | X | | |
| Return study medication/ compliance monitoring | | | | X[w] | X | X | X | X | | | X | | X[w] | X | X | X | | | X |
| Study medication dosing[x] | | | X | → | → | → | → | X | | | | X | → | → | → | X | | | |
| Study medication dose tapering | | | | | | | | | X[y] | → | X | | | | | | X | → | X |
| Telephone call to subject[z] | | | | | | | | | | X | | | | | | | | X | |
| Time to Stand Test (TTSTAND) | X | X | | | X | X | | X | | | | | | X | X | X | | | |
| Time to Climb Test (TTCLIMB) | X | X | | | | X | | X | | | | | | | X | X | | | |
| Time to Run/Walk Test (TTRW) | X | X | | | | X | | X | | | | | | | X | X | | | |
| NSAA[aa] | X | X | | | | X | | X | | | | | | | X | X | | | |
| Myometry (elbow flexors, knee extensors) | X | X | | | | X | | X | | | | | | | X | X | | | |
| Six-minute Walk Test (6MWT) | X | X | | | | X | | X | | | | | | | X | X | | | |
| Range of Motion (ROM) - ankles | X | X | | | | X | | X | | | | | | | X | X | | | |
| Pediatric Outcomes Data Collection Instrument (PODCI) | X | | | | | | | X | | | | | | | | X | | | |
| Treatment Satisfaction Questionnaire (TSQM) | | | | | | | | X | | | | | | | | X | | | |

| | Pretreatment Period | | Treatment Period #1 | | | | | | | Transition Period | Treatment Period #2 | | | | | | | Dose-tapering Period | |
|---|---|---|---|---|---|---|---|---|---|---|---|---|---|---|---|---|---|---|---|
| | SCR | BL | | | | | | | | | | | | | | | | | |
| | Day | | | Week | | | | | | | | | | | | | | | |
| **Study Day or Week/Visit** | **-33 to -2[a]** | **-1[b]** | **1[c]** | **2 (±1d)[d]** | **6 (±3d)[d]** | **12 (±1w)[d]** | **18 (±1w)[d]** | **24[e] (±1w)[d]** | **24 (F/U)[e]** | **26** | **28[f] (±1d)[d]** | **28+1d** | **30 (±1d)[d]** | **34 (±3d)[d]** | **40 (±1w)[d]** | **48[g] (±1w)[d]** | **48 F/U[g]** | **50** | **52[h] (±1d)[d]** |
| PARS III | X | | | | | X | | X | | | | | | | | X | | | |
| Ease of Study Medication Administration Assessment[bb] | | | | X | | X | | X | | | | | X | | X | X | | | |
| Blindedness Assessment | | | | | | | | X | | | | | | | | | | | |
| Dispense subject diaries[cc] | | | X | X | X | X | X | X | | | X | | X | X | X | X | | | |
| Return subject diaries | | | | X | X | X | X | X | X[dd] | | X | | X | X | X | X | X[ee] | | X |
| AE/SAE recording[ff] | X → | | | | | | | | | | | | | | | | | → | X[gg] |
| Concomitant medications | | | X → | | | | | | | | | | | | | | | → | X |
| Discharge from study | | | | | | | | | | | | | | | | | X[hh] | | X[ii] |

BL = Baseline; d = day(s); F/U = Follow-up; SCR = Screening; w = week.

a. The Pretreatment Screening Period spans Day -33 through Day -2, but all screening procedures must be completed by Day -11. Subjects meeting all eligibility criteria will be randomized by Day -11, at least 10 days prior to the Baseline Day -1 Visit.

b. Baseline Day -1, within 24 hours prior to administration of the first dose of study drug.

c. Treatment Day 1 begins at the time of administration of the first dose of study medication in the clinic.

d. Time windows around the Week 2, Week 6, Week 12, Week 18, and Week 24 Visits are allowances from date of Day 1 Visit. Time window around the Week 28 Visit is allowance from date of Week 24 F/U Visit. Time windows around the Week 30, Week 34, Week 40, and Week 48 Visits are allowances from date of Week 28+1d Visit. Time window around the Week 52 Visit is allowance from date of Week 48 F/U Visit.

e. Subjects who prematurely discontinue from the study prior to Week 24 should complete the Week 24 assessments and the Week 24 Follow-up Visit ACTH Stimulation Test at the time of early withdrawal and undergo Early Discontinuation Dose-tapering, where possible (see **Section 6.4.1** and **Section 7.2.7**). The Week 24 Follow-up Visit must occur to allow the ACTH Stimulation Test to be performed 48 ± 3 hours after the final dose of Treatment Period #1 study medication.

f. Subjects who prematurely discontinue from the study after Week 24 but prior to Week 28 should complete the Week 28 assessments, and undergo Early Discontinuation Dose-tapering, where possible (see **Section 6.4.2**).

g. Subjects who prematurely discontinue from the study after Week 28 but prior to Week 48 should complete the Week 48 assessments and the Week 48 Follow-up Visit ACTH Stimulation Test at the time of early withdrawal and undergo Dose-tapering, where possible (see **Section 6.4.3** and **Section 7.2.7**). The Week 48 Follow-up Visit must occur to allow the ACTH Stimulation Test to be performed 48 ± 3 hours after the final dose of Treatment Period #2 study medication.

h. Subjects will have one study site visit during the Dose-tapering Period, at one week after the dose of liquid formulation has been discontinued (Week 52) (see **Section 6.3.7**).

i. Subjects are considered to be enrolled in the study at the time written informed consent is obtained.
j. Study eligibility should be rechecked and confirmed at Baseline Day -1 Visit.
k. Randomization occurs by Interactive Voice/Web Response System (IXRS) after subjects are confirmed to have met all study entry criteria, at least 10 days prior to the Baseline Day -1 Visit.
l. Weight recorded at the Week 18 Visit and the Week 40 Visit will be used to calculate doses for study drug dispensed at the Week 24 Follow-up and Week 48 Follow-up Visits, respectively.
m. Sitting blood pressure, body temperature, respiratory rate, and heart rate.
n. Vital signs recorded prior to administration of the first dose of study drug at the Day 1 Visit.
o. Blood for hematology, chemistry, and lipids, including HbA1c and Vitamin D where applicable.
p. Blood samples (collected after subjects have fasted for ≥ 6 hours) and urine collected at scheduled visit, and prior to dose of study drug where applicable.
q. Urinalysis by dipstick and microscopic analysis.
r. Blood collected for PD biomarkers includes secondary safety outcomes (morning cortisol, osteocalcin, CTX1, P1NP), and exploratory safety and efficacy PD biomarkers.
s. Blood samples for PD biomarkers and fasting glucose and insulin determination will be collected after subjects have fasted for ≥ 6 hours, prior to the daily dose of study medication where applicable.
t. Subjects will return to the study site for the Week 24 Follow-up Visit for an ACTH Stimulation Test 48 hours ± 3 hours after administration of the final dose of Treatment Period #1 study medication, and for the Week 48 Follow-up Visit for an ACTH Stimulation Test 48 hours ± 3 hours after administration of the final dose of Treatment Period #2 study medication (see **Section 7.2.7**).
u. Blood sample for population PK analysis will be collected 2 hours after administration of the daily dose of study medication.
v. 12-lead ECG recorded after subject has rested quietly in a supine position for at least 5 minutes.
w. Study medication brought by subjects to the Week 2 Visit and Week 30 Visit for dosing and compliance assessment will be redispensed to subjects at the end of the visit.
x. The dose of study medication on the days of the Day 1, Week 2, Week 12, Week 24, Week 28, Week 30, Week 40, and Week 48 Visits will be administered after 1) fasting blood draws; and 2) breakfast provided by the study site. All other doses will be taken at home. See **Section 6.3.3** for other Day 1 pre-dose safety assessments.
y. Doses of tablet study drug will be tapered and suspension study drug will be continued, during Weeks 24-28.
z. Site study staff will contact the parent(s)/guardian(s) by telephone at Weeks 26 and 50 to ensure that the study drug tapering is proceeding according to protocol, to assess potential signs or symptoms indicative of adrenal suppression, and to address any questions the parent(s)/guardian(s) may have.
aa. North Star Ambulatory Assessment; includes the Time to Stand Test (TTSTAND).
bb. Ease of Study Medication Administration assessed at the Weeks 2, 12, and 24, 30, 40, and 48 Visits.
cc. Subject diaries used to record any changes in concomitant medications taken, any AEs experienced during the study, and any incomplete or missed doses of study medication.
dd. Subject diaries dispensed at the Week 24 Visit will be returned and redispensed at the Week 24 F/U Visit; final return will occur at the Week 28 Visit.
ee. Subject diaries dispensed at the Week 48 Visit will be returned at the Week 48 F/U Visit, and will be redispensed to subjects participating in the Dose-tapering Period for final return at the Week 52 Visit.
ff. All AEs and SAEs must be recorded in the source documents and eCRF from the date of the subject's written informed consent until the final Week 52 Visit or the subject's participation in the study is completed (SAEs through 30 days after final study drug dose). Ongoing AEs will be followed to resolution, stabilization, or until such time the Investigator agrees follow-up is not necessary.

gg. For subjects who do not continue to receive vamorolone through an additional vamorolone study or general access program, site staff will make a phone call to the home 31-35 days after the final dose of study medication in VBP15-004 Dose-tapering Period to confirm the final SAE status of the subject.

hh. Subjects who elect to continue vamorolone therapy by enrolling directly into an additional vamorolone study or general access program may be discharged from the study following completion of all final Week 48 assessments, including the Week 48 Follow-up Visit ACTH Stimulation Test.

ii. Subjects who participate in the Dose-tapering Period may be discharged from the study following completion of all final Dose-Tapering Visit assessments (Week 52) (see **Section 6.3.7**).

**Section Changed:**

**Section 6.3: Visit Schedule and Procedures, paragraph 1**

***Original Text***

During the study, there will be a total of up to 16 study site visits: Pretreatment Screening Visit (screening procedures can be performed on more than one day if necessary); Pretreatment Baseline Day -1 Visit; Treatment Period #1 Day 1 and Weeks 2, 6, 12, 18, and 24 Visits, and Week 24 Follow-up Visit; Transition Period Week 28 Visit; Treatment Period #2 Weeks 30, 34, 40, 48 Visits, and Week 48 Follow-up Visit; and final Dose-tapering Period Week 52 Visit. In addition, to facilitate completion of all required Week 24 and Week 48 assessments, some of the assessments scheduled for the Week 24 and Week 48 Visits may be completed on the day following the Week 24 (Week 48) dose of study medication and/or the day of the Week 24 (Week 48) Follow-up Visit, if needed (see **Section 6.3.4** and **Section 6.3.6**).

***Revised Text***

During the study, there will be a total of up to 16 study site visits: Pretreatment Screening Visit (screening procedures can be performed on more than one day if necessary); Pretreatment Baseline Day -1 Visit; Treatment Period #1 Day 1 and Weeks 2, 6, 12, 18, and 24 Visits, and Week 24 Follow-up Visit; Transition Period Week 28 Visit; Treatment Period #2 Weeks 30, 34, 40, 48 Visits, and Week 48 Follow-up Visit; and final Dose-tapering Period Week 52 Visit. In addition, to facilitate completion of all required Week 24 and Week 48 assessments, some of the assessments scheduled for the Week 24 and Week 48 Visits may be completed on the day following the Week 24 (Week 48) dose of study medication, the day of the Week 24 (Week 48) Follow-up Visit, or in some cases up to 7 days following the date of the Week 24 (Week 48) dose of study medication, if needed (see **Section 6.3.4** and **Section 6.3.6**).

**Section 6.3: Visit Schedule and Procedures, paragraph 3**

***Original Text***

Each subject will receive double-blind study medication at stable daily dose for a period of 20 weeks (Treatment Period #2). Following completion of the 20-week Treatment Period #2, all subjects who will not be continuing into the long-term extension study (separate protocol) will taper their liquid formulation study medication during the 4-week double-blind Dose-Tapering Period, and will return to the study site for study assessments at the end of the Dose-Tapering Period (Week 52). See **Section 7** for a detailed description of the safety, clinical efficacy, PD, and PK assessments to be performed in this study.

***Revised Text***

Each subject will receive double-blind study medication at stable daily dose for a period of 20 weeks (Treatment Period #2). Following completion of the 20-week Treatment Period #2, all subjects who will not be continuing to receive vamorolone in an additional vamorolone study or general access program (separate protocol) will taper their liquid formulation study medication during the 4-week double-blind Dose-Tapering Period, and will return to the study site for study assessments at the end of the Dose-Tapering Period (Week 52). See **Section 7** for a detailed description of the safety, clinical efficacy, PD, and PK assessments to be performed in this study.

**Section Changed:**

**Section 6.3.4: Treatment Period #1 (Weeks 1-24), paragraphs 6 and 7**

***Original Text***

Clinical efficacy assessments (TTSTAND, TTRW, TTCLIMB, NSAA, 6MWT, hand-held myometry, and ROM) and the subject reported outcomes (TSQM, PODCI, Ease of Study Medication Administration Assessment, Child Behavior Checklist, and PARS III) will be conducted as specified in the schedule of study activities (**Table 11**). Weight will be recorded at every visit and height will be measured at 12-week intervals. Vital signs will be recorded at each study visit. A physical examination including

assessment of cushingoid features will be performed every 6 weeks. A 12-lead ECG will be recorded at Weeks 12 and 24. 2D-echocardiography will be performed at Week 24. Blood and urine samples for clinical laboratory tests and blood for the serum PD biomarker panel will be collected at scheduled visits throughout the study (**Table 11**). An eye examination to exclude cataracts and glaucoma will be performed at Week 24. A DXA scan and spine X-ray will be performed at Week 24. A Blindedness Assessment will be completed by the parent(s)/guardian(s) at the Week 24 Visit. Adverse events, including SAEs, and concomitant medications will be assessed at each study visit and recorded throughout the study.

There is flexibility in the timing of completion of some of the scheduled Week 24 assessments. The scheduled physical examination, weight, vital signs, clinical laboratory tests, blood draws for PD biomarker analysis, Ease of Study Medication Administration Assessment, and functional assessments (TTSTAND, TTCLIMB, TTRW, NSAA, 6MWT, hand-held myometry, ROM) should all be performed on the date of the final Week 24 dose of Treatment Period study medication. However, subjects may return to the study site on the day following the final Week 24 dose of study medication and/or the day of the Week 24 Follow-up Visit for completion of the DXA scan, spine X-ray, Fracture Questionnaire, 12-lead ECG, 2-D echocardiography, eye examination, PODCI, TSQM, Child Behavior Checklist, PARS III, and Blindedness Assessment.

***Revised Text***

Clinical efficacy assessments (TTSTAND, TTRW, TTCLIMB, NSAA, 6MWT, hand-held myometry, and ROM) and the subject reported outcomes (TSQM, PODCI, Ease of Study Medication Administration Assessment, and PARS III) will be conducted as specified in the schedule of study activities (**Table 11**). Weight will be recorded at every visit and height will be measured at 12-week intervals. Vital signs will be recorded at each study visit. A physical examination including assessment of cushingoid features will be performed every 6 weeks. A 12-lead ECG will be recorded at Weeks 12 and 24. 2D-echocardiography will be performed at Week 24. Blood and urine samples for clinical laboratory tests and blood for the serum PD biomarker panel will be collected at

scheduled visits throughout the study (**Table 11**). Blood will be collected at Week 24 for DNA testing for candidate genetic modifiers of DMD. An eye examination to exclude cataracts and glaucoma will be performed at Week 24. A DXA scan and spine X-ray will be performed at Week 24. A Blindedness Assessment will be completed by the parent(s)/guardian(s) at the Week 24 Visit. Adverse events, including SAEs, and concomitant medications will be assessed at each study visit and recorded throughout the study.

There is flexibility in the timing of completion of some of the scheduled Week 24 assessments. The scheduled physical examination, weight, vital signs, clinical laboratory tests, blood draws for PD biomarker analysis and DNA testing, Ease of Study Medication Administration Assessment, PODCI, PARS III, and functional assessments (TTSTAND, TTCLIMB, TTRW, NSAA, 6MWT, hand-held myometry, ROM) must all be performed on the date of the final Week 24 dose of Treatment Period #1 study medication. The 12-lead ECG may be performed on the date of the final Week 24 dose of study medication, the day following the final Week 24 dose of study medication or the day of the Week 24 Follow-up Visit. Completion of the DXA scan, spine X-ray, Fracture Questionnaire, 2-D echocardiography, eye examination, TSQM, and Blindedness Assessment may be performed on the date of the final Week 24 dose of study medication or up to 7 days following the date of the final Week 24 dose of study medication to accommodate need for additional scheduling flexibility.

**Section 6.3.4: Treatment Period #1 (Weeks 1-24), paragraph 8, sentence 1**

***Original Text***

At the Week 24 Visit, all subjects will be dispensed a single dose of oral hydrocortisone (5 mg or 10 mg) to be administered at home, 24 hours after administration of the final dose of study medication at the Week 24 Visit.

***Revised Text***

At the Week 24 Visit, all subjects will be dispensed a single dose of oral hydrocortisone (5 mg or 10 mg) to be administered 24 hours after administration of the final dose of study medication at the Week 24 Visit.

**Section Changed:**

**Section 6.3.6: Treatment Period #2 (Week 28+1 Day through Week 48), paragraph 8**

***Original Text***

There is flexibility in the timing of completion of some of the scheduled Week 48 assessments. The scheduled physical examination, weight, vital signs, clinical laboratory tests, blood draws for PD biomarker analysis, Ease of Study Medication Administration Assessment, and functional assessments (TTSTAND, TTCLIMB, TTRW, NSAA, 6MWT, hand-held myometry, ROM) should all be performed on the date of the final Week 48 dose of Treatment Period study medication. However, subjects may return to the study site on the day following the final Week 48 dose of study medication and/or the day of the Week 48 Follow-up Visit for completion of the DXA scan, Fracture Questionnaire, 12-lead ECG, 2-D echocardiography, eye examination, PODCI, TSQM, Child Behavior Checklist, and PARS III (see below).

***Revised Text***

There is flexibility in the timing of completion of some of the scheduled Week 48 assessments. The scheduled physical examination, weight, vital signs, clinical laboratory tests, blood draws for PD biomarker analysis, Ease of Study Medication Administration Assessment, PODCI, PARS III, and functional assessments (TTSTAND, TTCLIMB, TTRW, NSAA, 6MWT, hand-held myometry, ROM) must all be performed on the date of the final Week 48 dose of Treatment Period #2 study medication. The 12-lead ECG may be performed on the date of the final Week 48 dose of study medication, the day following the final Week 48 dose of study medication, or the day of the Week 48 Follow-up Visit. Completion of the DXA scan, Fracture Questionnaire, 2-D echocardiography, eye examination, and TSQM may be performed on the date of the final Week 48 dose of study medication, the day following the Week 48 dose of study medication, or the day of the Week 48 Follow-up Visit (for subjects receiving vamorolone therapy by enrolling directly into an additional vamorolone study or general access program), or up to 7 days following the date of the final Week 48 dose of study medication (for subjects

participating in the Dose-tapering Period) to accommodate need for additional scheduling flexibility.

**Section 6.3.6: Treatment Period #2 (Week 28+1 Day through Week 48), paragraph 9, sentence 1**

***Original Text***

At the Week 48 Visit, all subjects will be dispensed a single dose of oral hydrocortisone (5 mg or 10 mg) to be administered at home, 24 hours after administration of the final dose of study medication at the Week 48 Visit. Revised

***Revised Text***

At the Week 48 Visit, all subjects will be dispensed a single dose of oral hydrocortisone (5 mg or 10 mg) to be administered 24 hours after administration of the final dose of study medication at the Week 48 Visit.

**Section 6.3.6: Treatment Period #2 (Week 28+1 Day through Week 48), paragraph 11**

***Original Text***

At the end of the 20-week Treatment Period #2, including the Week 48 Follow-up Visit for the ACTH Stimulation Test, subjects will be given the option of enrolling into a Phase IIb long-term extension study. Subjects enrolling directly into the long-term extension study will not need to taper their vamorolone dose prior to enrollment into the study. All other subjects will begin a 4-week double-blind Dose-tapering Period during which the doses of suspension study medication will be progressively reduced and discontinued (see **Section 6.3.7**).

***Revised Text***

At the end of the 20-week Treatment Period #2, including the Week 48 Follow-up Visit for the ACTH Stimulation Test, subjects may be given the option of continuing vamorolone therapy by enrolling into an additional vamorolone study or general access program. Subjects enrolling directly into an additional vamorolone study or general access program will not need to taper their vamorolone dose prior to enrollment. All

other subjects will begin a 4-week double-blind Dose-tapering Period during which the doses of suspension study medication will be progressively reduced and discontinued (see **Section 6.3.7**).

**Section Changed:**

**Section 6.3.7: Dose-tapering Period (Weeks 49-52), paragraph 1, sentence 1**

***Original Text***

All subjects who complete the study and opt not to enroll into the long-term extension study will participate in a 4-week double-blind Dose-tapering Period during which the doses of suspension study medication will be progressively reduced and discontinued.

***Revised Text***

All subjects who complete the study and opt not to continue vamorolone therapy by enrolling into an additional vamorolone study or general access program will participate in a 4-week double-blind Dose-tapering Period during which the doses of suspension study medication will be progressively reduced and discontinued.

**Section Changed:**

**Section 7.1: Demographic Assessments**

***Original Text***

Demographic information (birth date, race, and ethnicity) will be collected during the Pretreatment Screening Period and will be recorded on the appropriate eCRF page.

***Revised Text***

Demographic information (birth date, race, and ethnicity) will be collected during the Pretreatment Screening Period and will be recorded on the appropriate eCRF page.

### *7.1.1 Genetic Modifiers of DMD*

Approximately 6 mL of blood will be collected at the Week 24 Visit for DNA testing to determine if candidate genetic modifiers of DMD (gene polymorphisms associated with disease severity or response to glucocorticoid treatment) are similarly associated with

vamorolone-treated DMD patients (baseline disease severity or response to vamorolone or prednisone treatment).

DNA testing will be performed by a certified central laboratory.

The procedures for the collection, handling, and shipping of blood samples for DNA testing will be specified in the Laboratory Manual(s) provided to the clinical center.

**Section Changed:**

**Section 7.2.7: paragraph 2, sentence 1**

***Original Text***

All subjects will be given a single dose of hydrocortisone (5 mg or 10 mg) at home 24 hours after the dose of study medication at the Week 24 Visit and 24 hours after the dose of study medication at the Week 48 Visit.

***Revised Text***

All subjects will be given a single dose of hydrocortisone (5 mg or 10 mg) 24 hours after the dose of study medication at the Week 24 Visit and 24 hours after the dose of study medication at the Week 48 Visit.

**Section 7.2.7: paragraph 4**

***Original Text***

The ACTH Stimulation Test involves insertion of a saline lock and then administration of 250 µg of Cosyntropin at time zero. Blood samples for cortisol measurement are collected at time 0 immediately prior to Cosyntropin administration, and at 30 and 60 minutes after Cosyntropin administration.

***Revised Text***

The ACTH Stimulation Test involves insertion of a saline lock and then administration of 250 µg of Cosyntropin at time zero. Blood samples for cortisol measurement are collected at time 0 immediately prior to Cosyntropin administration, and at 30 ± 5 minutes and 60 ± 5 minutes after Cosyntropin administration.

**Section 7.2.7: paragraph 7**

***Original Text***

Blood samples will be sent to a central laboratory, and results centrally interpreted (Children's Hospital of Eastern Ontario).

***Revised Text***

Blood samples will be sent to a central laboratory, and results centrally interpreted (Children's Hospital of Eastern Ontario). Results of the Week 24 analysis will not be reported to the study sites.

**Section Changed:**

**Section 7.2.9: Total Blood Volume Required, paragraph 1**

***Original Text***

The number and volume of blood samples and total volume of blood to be collected from each subject throughout the duration of the 57-week study are summarized in **Table 20**. A total of 176 mL of blood will be collected from each subject over the course of the up-to-57-week study.

***Revised Text***

The number and volume of blood samples and total volume of blood to be collected from each subject throughout the duration of the 57-week study are summarized in **Table 20**. A total of 182 mL of blood will be collected from each subject over the course of the up-to-57-week study.

## Section 7.2.9: Total Blood Volume Required, Table 20

***Original Text:***

### Table 20. Blood Sample Number and Volume by Study Visit

| Test | Total mL of Blood | | | | | | | | | | | | | | | |
|---|---|---|---|---|---|---|---|---|---|---|---|---|---|---|---|---|
| | **SCR** | **Day 1** | **Week 2** | **Week 6** | **Week 12** | **Week 18** | **Week 24** | **Week 24 F/U** | **Week 28** | **Week 30** | **Week 34** | **Week 40** | **Week 48** | **Week 48 F/U** | **Week 52** | **Total Volume** |
| Clinical Safety Labs[a] | 12[b] | 10[c] | 10[c] | 10 | 12[c] | 10 | 12[b, c] | | 10[c] | 10[c] | 10 | 12[c] | 12[b,c] | | 10[c] | 140[b] |
| Varicella Zoster IgG | 2 | | | | | | | | | | | | | | | 2 |
| PD Biomarker Panel[d] | | 2 | | | 2 | | 2 | | 2 | | | 2 | 2 | | 2 | 14 |
| PD Insulin/Glucose[c] | | From Clinical Safety Lab Sample | | | From Clinical Safety Lab Sample | | From Clinical Safety Lab Sample | | From Clinical Safety Lab Sample | | | From Clinical Safety Lab Sample | From Clinical Safety Lab Sample | | From Clinical Safety Lab Sample | |
| ACTH Stimulation Test[e] | 6 | | | | | | | 6 | | | | | | 6 | | 18 |
| PK[f] | | | | | | | | | | 2 | | | | | | 2 |
| Total Volume by Visit (mL) | 20 | 12 | 10 | 10 | 14 | 10 | 14 | 6 | 12 | 12 | 10 | 14 | 14 | 6 | 12 | 176 |
| **Total Volume: 176 mL** | | | | | | | | | | | | | | | | |

F/U = Follow-up; SCR = Screening

[a] Hematology, Chemistry, Lipids; GLDH; volume includes blood for vitamin D testing, fasting insulin and glucose, cortisol, and exploratory PD biomarkers, where applicable.

[b] Includes blood for HbA1c testing.

[c] Subjects must have fasted ≥ 6 hours prior to blood draws.

[d] Osteocalcin, CTX1, P1NP, pre-dose on dosing days; subjects must have fasted ≥ 6 hours prior to pre-dose and Week 52 blood draws.

[e] ACTH Stimulation Test performed at Screening, Week 24 Follow-up Visit, 48 ± 3 hours after the final dose of Treatment Period #1 study medication and prior to first dose of study medication in the Transition Period, and Week 48 Follow-up Visit, 48 ± 3 hours after the final dose of Treatment Period #2 study medication and prior to first dose of study medication in the Dose-Tapering Period.

[f] Blood drawn for population PK at 2 hours post-dose at the Week 30 Visit.

***Revised Text***

## Table 20. Blood Sample Number and Volume by Study Visit

| Test | Total mL of Blood | | | | | | | | | | | | | | | |
|---|---|---|---|---|---|---|---|---|---|---|---|---|---|---|---|---|
| | **SCR** | **Day 1** | **Week 2** | **Week 6** | **Week 12** | **Week 18** | **Week 24** | **Week 24 F/U** | **Week 28** | **Week 30** | **Week 34** | **Week 40** | **Week 48** | **Week 48 F/U** | **Week 52** | **Total Volume** |
| Clinical Safety Labs[a] | 12[b] | 10[c] | 10[c] | 10 | 12[c] | 10 | 12[b,c] | | 10[c] | 10[c] | 10 | 12[c] | 12[b,c] | | 10[c] | 140[b] |
| Varicella Zoster IgG | 2 | | | | | | | | | | | | | | | 2 |
| PD Biomarker Panel[d] | | 2 | | | 2 | | 2 | | 2 | | | 2 | 2 | | 2 | 14 |
| PD Insulin/Glucose[c] | | From Clinical Safety Lab Sample | | | From Clinical Safety Lab Sample | | From Clinical Safety Lab Sample | | From Clinical Safety Lab Sample | | | From Clinical Safety Lab Sample | From Clinical Safety Lab Sample | | From Clinical Safety Lab Sample | |
| DNA Testing | | | | | | | 6 | | | | | | | | | 6 |
| ACTH Stimulation Test[e] | 6 | | | | | | | 6 | | | | | | 6 | | 18 |
| PK[f] | | | | | | | | | | 2 | | | | | | 2 |
| Total Volume by Visit (mL) | 20 | 12 | 10 | 10 | 14 | 10 | 20 | 6 | 12 | 12 | 10 | 14 | 14 | 6 | 12 | 182 |
| **Total Volume: 182 mL** | | | | | | | | | | | | | | | | |

F/U = Follow-up; SCR = Screening

[a] Hematology, Chemistry, Lipids; GLDH; volume includes blood for vitamin D testing, fasting insulin and glucose, cortisol, and exploratory PD biomarkers, where applicable.

[b] Includes blood for HbA1c testing.

[c] Subjects must have fasted ≥ 6 hours prior to blood draws.

[d] Osteocalcin, CTX1, P1NP, pre-dose on dosing days; subjects must have fasted ≥ 6 hours prior to pre-dose and Week 52 blood draws.

[e] ACTH Stimulation Test performed at Screening, Week 24 Follow-up Visit, 48 ± 3 hours after the final dose of Treatment Period #1 study medication and prior to first dose of study medication in the Transition Period, and Week 48 Follow-up Visit, 48 ± 3 hours after the final dose of Treatment Period #2 study medication and prior to first dose of study medication in the Dose-Tapering Period.

[f] Blood drawn for population PK at 2 hours post-dose at the Week 30 Visit.

**Section Changed:**

**Section 7.2.13: Bone Health and DXA Scan (total body and spine), paragraphs 1 and 2**

***Original Text***

Data on bone mass, density and body composition (fat and fat-free mass) will be collected by DXA during the Screening Period (depending on local facilities, this may require an additional visit but should be completed prior to starting study medication), and at Week 24 and Week 48.

Antero-posterior spine (L1-L4) and total body (without head) bone mineral density (BMD) by DXA scan will be collected. DXA quality control will be performed as described in the Manual of Operations. DXA scans will be analyzed centrally by a certified medical radiation technologist at Children's Hospital of Ottawa in Ottawa, Canada, and then age- and gender-specific Z-scores will be generated in order to chart the differences in the change in BMD Z-scores among the different groups participating in this trial. The Screening result will represent a baseline assessment for long-term follow up. Additional DXA scanning may be arranged if clinically indicated throughout the study.

***Revised Text***

Data on bone mass, density, and total body composition (fat mass, fat-free mass, lean mass, Lean Mass Index, and Fat Mass Index) will be collected by DXA during the Screening Period (depending on local facilities, this may require an additional visit but should be completed prior to starting study medication), and at Week 24 and Week 48.

Antero-posterior spine (L1-L4) and total body (without head) bone mineral density (BMD) by DXA scan will be collected. DXA quality control will be performed as described in the Manual of Operations. DXA scans will be analyzed centrally by a certified medical radiation technologist at Children's Hospital of Ottawa in Ottawa, Canada, and then age-specific Z-scores will be generated in order to chart the differences in the change in BMD Z-scores among the different groups participating in this trial. The

Screening result will represent a baseline assessment for long-term follow up. Additional DXA scanning may be arranged if clinically indicated throughout the study.

**Section Changed:**

**Section 7.4.1: Pediatric Outcomes Data Collection Instrument (PODCI), paragraph 1**

***Original Text***

Quality of life will be assessed by completion of the Pediatric Outcomes Data Collection Instrument (PODCI). The subject parent/legal guardians will be asked to complete this instrument at the Screening, Week 24, and Week 48 Visits.

***Revised text***

Physical functioning will be assessed by completion of the Pediatric Outcomes Data Collection Instrument (PODCI). The subject parent/legal guardians will be asked to complete this instrument at the Screening, Week 24, and Week 48 Visits.

**Section Changed:**

**Section 7.4.3: Behavioral Assessments, paragraph 1**

***Original Text***

***7.4.3 Behavioral Assessments***

Two instruments, for completion by the parent(s)/guardian(s), will be used for behavior assessment screening and evaluation of behavior change. These are (a) the Child Behavior Checklist and (b) the PARS III, a scale designed to measure psychosocial adjustment of children with chronic physical illnesses. Each instrument will be completed by the parent(s)/guardian(s) at the Screening Visit and at the Weeks 12, 24, and 48 Visits. The Child Behavior Checklist and the PARS III are available in all primary languages spoken at sites for this study.

***Revised Text***

***7.4.3 Behavioral Assessment***

One instrument, for completion by the parent(s)/guardian(s), will be used for behavior assessment screening and evaluation of behavior change. This is the PARS III, a scale designed to measure psychosocial adjustment of children with chronic physical illnesses. The instrument will be completed by the parent(s)/guardian(s) at the Screening Visit and at the Weeks 12, 24, and 48 Visits. The PARS III is available in all primary languages spoken at sites for this study.

**Sections Changed:**

**Throughout Text:**

***Original Text***

Child Behavior Checklist

***Revised Text***

[text deleted]

**Section Changed:**

**Section 8.1: Study Steering Committee**

***Original Text***

A study steering committee (SSC – VISION-DMD) will be responsible for protocol development, review of any study amendments, and coordination of study conduct and interpretation of study results. The SSC comprises the Sponsor, study chairs and medical monitors for the VBP15-003, VBP15-LTE, VBP15-004, and long-term extension studies, the project managers, and the patient representatives.

***Revised Text***

A study steering committee (SSC – VISION-DMD) will be responsible for protocol development, review of any study amendments, and coordination of study conduct and interpretation of study results. The SSC comprises the Sponsor, study chairs and medical monitors for the VBP15-003, VBP15-LTE, VBP15-004, and other studies, as applicable, the project managers, and the patient representatives.

**Section Changed:**

**Section 8.2: Data and Safety Monitoring Board, paragraph 3**

***Original Text***

The DSMB will be responsible for:

- Examining accumulating safety and other relevant data at pre-specified points during the course of the study in order to make recommendations concerning continuation, termination, or modification of the study;
- Examining accumulating clinical efficacy data in order to make recommendations concerning continuation, termination, or modification of the study;
- Reviewing important protocol deviations;
- Providing expert advice to the SSC on an ad hoc basis regarding matters such as safety concerns or diagnostic evaluations in individual subjects;

Based on the results of its deliberations, the DSMB can recommend continuation of the studies unchanged, study interruption, study termination, modification of the studies, or alteration in the DSMB monitoring plan.

***Revised Text***

The DSMB will be responsible for:

- Examining accumulating safety and other relevant data at pre-specified points during the course of the study in order to make recommendations concerning continuation, termination, or modification of the study;
- Reviewing important protocol deviations;
- Providing expert advice to the SSC on an ad hoc basis regarding matters such as safety concerns or diagnostic evaluations in individual subjects;

Based on the results of its deliberations, the DSMB can recommend continuation of the studies unchanged, study interruption, study termination, modification of the studies, or alteration in the DSMB monitoring plan.

**Section Changed:**

**Section 10.6: Week 24 Analysis**

***Original Text***

The primary analyses for this study are the analyses which will be performed after all subjects complete Week 24 of Treatment Period #1. The results from these analyses will be provided to regulatory authorities. Investigators, study staff, and monitors will remain blinded.

***Revised Text***

The primary analyses for this study are the analyses which will be performed after all subjects complete Week 24 of Treatment Period #1. The results from these analyses will be provided to regulatory authorities. Investigators, study subjects, study staff, and monitors will remain blinded throughout the duration of the 52-week study. The Sponsor, including data management and statistical personnel, will be unblinded after the Week 24 analyses.

**Section Changed:**

**Section 10.8: Missing, Unused, and Spurious Data**

***Original Text***

For presentations using descriptive statistics as well as listings, missing values for efficacy, safety and exploratory outcomes will be treated as missing. For the statistical analysis models used for the efficacy, safety and exploratory endpoints, missing data will not need to be imputed, as the mixed model has the capacity to handle unbalanced data when these are missing at random.

***Revised Text***

The primary analyses for the primary and secondary efficacy variables will be conducted on observed data only; the MMRMs will employ appropriate covariance structures to accommodate missing data, as described in **Section 10.9.4**. Missing efficacy data will be

imputed for supportive sensitivity analyses on the primary and secondary efficacy endpoints using the following methods:

- Multiple Imputations using Markov Chain Monte Carlo (MCMC); and
- Multiple Imputations using a Control-based Pattern Mixture Model.

Full details will be provided in the SAP.

**Section Changed:**

**Section 10.9.1: General Considerations, paragraph 1**

***Original Text***

Statistical analyses will be performed using SAS® version 9.2 or later.

***Revised Text***

Statistical analyses will be performed using SAS® version 9.4 or later.

**Section Changed:**

**Section 10.9.4: Efficacy Analyses, paragraph 5**

***Original Text***

Subjects who are randomized to receive vamorolone 2.0 mg/kg/day or vamorolone 6.0 mg/kg/day for both treatment periods will have TTSTAND velocity change from baseline data captured over 48 weeks compared with untreated DMD historical control data. Full details will be provided in the SAP.

***Revised Text***

The following sensitivity or supportive analyses will be performed (see SAP for full details) for the primary efficacy and secondary efficacy endpoints:

- Multiple imputations using MCMC methods for missing data;
- Multiple imputations using a Control-based Pattern-Mixture Model for missing data.

Subjects who are randomized to receive vamorolone 2.0 mg/kg/day or vamorolone 6.0 mg/kg/day for both treatment periods will have TTSTAND velocity change from baseline data captured over 48 weeks compared with untreated DMD historical control data. Full details will be provided in the SAP.

**Section Changed:**

**Section 11.2.3: Study Documentation, During the Study, bullet 2**

***Original Text***

- Any changes to the documentation identified above in **Section 11.2.3.1**

***Revised Text***

- Any changes to the documentation identified above (see ***Before the Start of the Study***)

**Section Changed:**

**Section 14: References**

***Original Text***

29. Investigator's Brochure, Version 7, Vamorolone (VBP15) (17α, 21-dihydroxy-16α-methyl-pregna-1,4,9(11)-triene-3,20-dione) 1.33% and 4% Oral Suspension, ReveraGen BioPharma, Inc., March 26, 2018.

***Revised Text***

29. Investigator's Brochure, Version 8, Vamorolone (VBP15) (17α, 21-dihydroxy-16α-methyl-pregna-1,4,9(11)-triene-3,20-dione) 1.33% and 4% Oral Suspension, ReveraGen BioPharma, Inc., September 24, 2018.
