# Supplementary material for: Efficacy and Safety of Vamorolone vs Placebo and Prednisone Among Boys With Duchenne Muscular Dystrophy: A Randomized Clinical Trial
Source: JAMA Neurol. 2022 Aug 29;79(10):1005–14. doi: 10.1001/jamaneurol.2022.2480 (PMC9425287; doi:10.1001/jamaneurol.2022.2480)
Supplement: Supplement 6. — Protocol Clarification Letter [file jamaneurol-e222480-s006.pdf]

Protocol Clarification Letter #2 for

VBP15-004

A Phase IIb Randomized, Double-blind, Parallel Group, Placebo- and Active-controlled Study with Double-Blind Extension to Assess the Efficacy and Safety of Vamorolone in Ambulant Boys with Duchenne Muscular Dystrophy (DMD)

Clarification Letter Date: 23 March 2020

CLARIFICATION LETTER

DATE: 23 March 2020

TO: VBP15-004 Investigators

FROM: VBP15-004 Protocol Team

SUBJECT: Clarification Letter #2 to the VBP15-004 Protocol (VBP15-004-A3, Version 1.3, 21 May 2019 and VBP15-004-A3.1UK, Version 1.1.3, 21 May 2019) entitled "A Phase IIb Randomized, Double-blind, Parallel Group, Placebo- and Active-controlled Study with Double-Blind Extension to Assess the Efficacy and Safety of Vamorolone in Ambulant Boys with Duchenne Muscular Dystrophy (DMD):"

Study Modifications During the Coronavirus (COVID-19) Pandemic

The following clarification to protocol VBP15-004 (VBP15-004-A3, Version 1.3, 21 May 2019 and VBP15-004-A3.1UK, Version 1.1.3, 21 May 2019) is intended to detail the modifications to study conduct and site monitoring that will be implemented during the global COVID-19 pandemic. In light of disruptions to subject site access and study staff availability during the COVID-19 pandemic, certain subject- and site-specific modifications will be made to the study assessments and procedures as described in the study protocol to ensure the safety of all participants, continued access to investigational product, and retention of study data integrity.

Vamorolone is a first-in-class steroid anti-inflammatory drug being evaluated for ability to ameliorate Duchenne muscular dystrophy (DMD), and prednisone, a glucocorticoid, is the active comparator in this study. Glucocorticoid treatment is a standard of care for DMD, and glucocorticoid dosing interruption during periods of (impending) illness or interruption of the trial, even if only temporary, could potentially compromise the overall well-being and best interest of trial participants. It is therefore imperative that subjects who have initiated treatment with investigational product in this study remain on treatment during the COVID-19 pandemic, with the safeguard of protocol-specified "stress dosing" of supplemental hydrocortisone as coverage during times of illness. The measures outlined in this Protocol Clarification Letter aim

to avoid study interruption. In addition, since the standard of care for DMD recommends steroid treatment be initiated prior to functional decline at an average age of 4-8 years in order to avoid greater risk of rapid deterioration, it would be detrimental to new subjects who have not yet been screened but wish to enroll in the study, (in compliance with site policy and Investigator discretion) to deny them entry into the study at sites that are currently allowing site access. For both subjects who have initiated treatment and new subjects who may be screened for entry into the study, the availability of off-site (remote) study visits (following an on-site Screening Visit), minimizes exposure to COVID-19, and further increases the benefit:risk ratio for subjects participating in this study.

This study will continue to be implemented per protocol at all sites and for all subjects, except in cases where the impact of COVID-19 prevents the performance of scheduled assessments or procedures as described in the current study protocol. In these cases, scheduled visits should still be conducted and scheduled assessments should still be performed per protocol, to the extent possible for each subject at each scheduled visit. At a minimum, the assessments described below and highlighted in **Table 1** must be performed, though they may be performed remotely, with the exception of assessments scheduled for the Screening Visit. Furthermore, it is expected that the study will again be implemented according to the current protocol on a per subject and per site basis as soon as possible upon alleviation of COVID-19 risks, at the discretion of the Investigator and study site. In all cases, deviations to the VBP15-004 protocol will be recorded in the clinical database and described in the Clinical Study Report. The following modifications will be made on a visit-, subject- or site-specific basis, as needed:

1. Informed Consent for Modifications to Study Procedures

The Investigator or designee will obtain informed re-consent from each subject's parent(s)/guardian(s) for the study modifications outlined in this Protocol Clarification Letter and for willingness to continue participation in the study. To enable the expeditious implementation of the modifications outlined, parent(s)/guardian(s) will be contacted by telephone or videoconference by site study staff who will present the modifications as detailed in this Letter, and any questions will be addressed; the telephone script will be submitted to the IRB/IEC at each site. The site staff will send by email (or mail, if necessary) to the parent(s)/guardian(s) at the time of the telephone contact a signature page to be signed by the parent(s)/guardian(s) indicating they understand the modifications as discussed on the telephone call and are willing to continue in the study. After the call, the site staff will document in the subject's source documents that the information was presented, the name of the staff member who contacted the parent(s)/guardian(s), and the date and time of contact. The parent(s)/guardian(s) will print out (if sent by email) and sign the signature page, and take a picture of the signed page on a smart phone and send the picture to the study staff, if possible. Alternatively, the parent(s)/guardian(s) may scan and email, or mail, the signed document back to the study site.

If applicable, the re-assent of the child will be obtained, if possible in writing by signing the signature page per individual where a child is intellectually capable of assenting (and in accordance with local regulations), and with the permission of the parent(s)/guardian(s), following the procedure above.

The signed signature page must be dated and signed by the Investigator or designee and the signed signature page must be kept by the Investigator in the study subject's file.

2. Visit Schedule and On-site Study Visits:

Subjects should attempt to complete as many of the scheduled study visits as possible at the study site. For all subjects, the Screening Visit must be performed on-site, and all scheduled Screening assessments must be completed per protocol, prior to randomization.

The study team has determined that the critical study assessments scheduled for Weeks 12, 24, 34, 40, 48 (and 52, if applicable) are necessary to maintain the safety and validity of the study. These assessments, highlighted in **Table 1,** may be performed remotely, with the modifications specified below. Every attempt should be made to conduct the assessments highlighted in **Table 1** at the specified assessment time point.

In all cases, for critical safety assessments, intervals between assessments should be no longer than 12 weeks (see #3 and #7, below). Subjects will be monitored for adverse events and concomitant medications at each remote visit. Scheduled safety assessments other than clinical laboratories, adverse events, concomitant medications, subject weight, and vital signs that cannot be performed on-site will not be performed remotely. The remote study visits will be conducted by telephone call, or by video call during which subjects may be observed by study staff if the parent(s)/guardian(s) give consent for a video interaction with the subject. All data collected remotely will be recorded in the eCRF.

Subject diaries will be dispensed according to the schedule in **Table 1**.

Additional remote interactions may be scheduled to assess efficacy (see #6, below).

The PODCI, TSQM, PARS III, Ease of Study Medication Administration, and the Blindedness Assessment will not be administered remotely.

3. Clinical Laboratory Assessments

Blood and urine may be collected and processed for clinical laboratory assessment (i.e., hematology, chemistry, urinalysis) either at a local physician's office, local laboratory, or by a visiting phlebotomist at each of the scheduled visits. Clinical laboratory assessments should be performed at each scheduled assessment time point, and in all cases at no longer than 12-week intervals. (This timing is based upon experience in the vamorolone VBP15-LTE study showing that subject safety is protected when clinical laboratory assessments are performed at 6-month intervals.) Results will be recorded in the eCRF. Subjects who are unable to obtain clinical laboratory assessments will be withdrawn from the study.

Blood for pharmacodynamic assessments may be collected if a visiting phlebotomist is used.

Blood for pharmacokinetic and ACTH stimulation assessments will not be collected if a local physician's office, local laboratory, or visiting phlebotomist is used.

At a minimum, the following clinical laboratory analytes should be assessed at each critical assessment time point (analytes not required are shown in strike-through font):

**Hematology**	
Red Blood Cells (RBC)	Numerical platelet count (estimate not acceptable)
Hemoglobin	White Blood Cells (WBC) with differential (percent)
Hematocrit	
**Chemistry**	
Sodium	Total Bilirubin[a]
Potassium	~~Uric Acid~~
Chloride	Glucose
Calcium	~~Glutamate dehydrogenase (GLDH)~~
Inorganic Phosphorus	Alkaline phosphatase (ALP)
Blood Urea Nitrogen (BUN)	Gamma Glutamyl Transferase (GGT)
Creatinine	Aspartate aminotransferase (AST)
Total Protein	Alanine aminotransferase (ALT)
Albumin	~~Creatine kinase (CK)~~
Bicarbonate	~~Lipase~~
Lactate Dehydrogenase (LDH)	~~Amylase~~
~~Cystatin C~~	~~Vitamin D~~[b]
HbA1c (collect if possible)	
**Lipids (collect if possible)**	
Triglycerides	Low Density Lipoprotein (LDL)
Total cholesterol	High density Lipoprotein (HDL)
a. If outside normal range, direct bilirubin will be measured and reported.	
**Urinalysis (including microscopic examination)**	
**Dipstick**[a]	**Microscopic Analysis**
Protein	~~WBC/hpf~~
Glucose	~~RBC/hpf~~
Ketones	~~Casts~~
pH	~~Bacteria~~
Leukocyte esterase	
Blood	
a. A midstream clean-catch urine specimen will be collected for dipstick analysis.	

4. Subject Weight

In the event subjects are unable to access the site for on-site study visits, subject weight should be recorded by a local physician's office, local laboratory, or visiting healthcare professional, optimally at no longer than 12-week intervals. Data will be recorded in the

eCRF with notation that the weight was recorded at a remote visit. Investigational product dose calculation will be based upon the weight (in kg) recorded at the most recent on-site or remote visit; it is understood that this may be the Screening Visit in all calculations for some subjects.

5. Vital Signs

In the event subjects are unable to access the site for on-site study visits, vital signs may be recorded by a local physician's office, or visiting healthcare professional, optimally at no longer than 12-week intervals. Data will be recorded in the eCRF with notation that the vital signs were recorded at a remote visit.

6. Efficacy Assessments

In cases where on-site study visits are not possible for the completion of scheduled efficacy assessments, the study staff will arrange with the parent(s)/guardian(s) to have the Time to Stand Test (TTSTAND) conducted remotely by a trained clinical evaluator (preferably, the site clinical evaluator, whenever possible) by videoconferencing interface (see **Appendix 1** for methodology). Remote assessment of TTSTAND must be completed at the Baseline, Week 24, and Week 48 assessment time points. If possible, remote assessment of TTSTAND at Week 12 may also be performed. Results will be recorded in the eCRF.

TTSTAND must be performed per protocol at the on-site Screening Visit (see #2, above).

All other scheduled efficacy assessments (i.e, TTRW, TTCLIMB, NSAA, 6MWT, ROM, Hand-held myometry) will not be collected remotely.

7. Collection of Adverse Events and Concomitant Medications

In the event subjects are unable to access the site for on-site study visits, adverse events and changes to concomitant medications will be reviewed by remote telephone or video call contact (see #2, above) between the Investigator or trained study staff and the subject's parent(s)/guardian(s). Data should be collected by site staff interview and review of diary entries at each remote visit, but in all cases at no longer than 12-week intervals. Data will be recorded in the eCRF.

8. Subject Diaries

In the event subjects cannot access the study site for the study visits at which subject diaries are scheduled to be dispensed and returned, site staff will email or mail new diaries to the parent(s)/guardian(s), and the parent(s)/guardian(s) will return completed diaries to the site by email or mail, at the protocol-specified intervals. Diary entries will be reviewed with the parents(s)/guardian(s) remotely by telephone or video call at no longer than 12-week intervals (see #7, above).

9. Dispensing of Investigational Product (IP)

Investigational product kits including bottles of vamorolone/placebo liquid suspension and blister packs of prednisone/placebo tablets will be shipped by the central pharmacy (Almac Clinical Services) to the clinical site and subsequently assigned using the IXRS system as currently detailed in the VBP15-004 protocol. In the case(s) where subjects are unable to come to the site to collect the IP kits, the assigned kit numbers will be couriered

from the site directly to the subjects' homes using a courier service, with confirmation of receipt. These couriered kits will be shipped under temperature conditions that are supported by the available stability data of the drug product.

Investigational Product sufficient for up to 12 weeks of dosing may be dispensed in a single shipment. Investigational Product will be dispensed at the time of the scheduled Week 24 Visit for the Transition Period, and Week 48 Visit for subjects entering the Dose-tapering Period, in lieu of dispensing at the time of the protocol-specified Week 24 Follow-up (F/U) and Week 48 F/U Visits, respectively.

10. Administration of IP

Investigational Product will be administered daily from Study Day 1 through Week 48 or Week 52 (for subjects participating in the Dose-tapering Period), as specified in the study protocol. For subjects who are unable to access the site for Week 24 F/U or Week 48 F/U Visits, IP will be dispensed at the Week 24 and Week 48 Visits (see #9, above). Transition Period dose tapering will begin the day after the remote Week 24 Visit, and IP tapering during the Dose-tapering Period will begin the day after the Week 48 Visit.

Tapering of IP during the Week 25-28 Transition Period and during the Week 49-52 Dose-tapering Period will continue to occur per protocol specifications.

11. IP Compliance Measurement

In cases where study site access is restricted due to COVID-19, subjects will be requested to retain used bottles and blister packs in a secure location at home, and bring them back to the site for compliance monitoring when they are able to return to the site for scheduled visit(s). In the event site access is restricted for the duration of the study, subjects will be requested to return used IP by mail, as directed by the Sponsor.

No interim compliance measurements will be performed at Week 2 and Week 30 for subjects who are unable to access the study sites.

12. Protocol Deviations

Protocol deviations will continue to be recorded as specified per study protocol, with important protocol deviations reported by the Investigator to the IRB/IEC and Sponsor or designee (Coordinating Center) within 24 hours of discovery, or according to local site requirements (VBP15-004 Protocol, Section 11.3). In addition, all deviations resulting from the COVID-19 pandemic will be recorded with documented reasons pertaining to COVID-19.

13. Transition to Vamorolone General Access Program

Subjects who complete the VBP15-004 Week 48 study assessments, as modified in this Protocol Clarification Letter, may be given access to vamorolone through a general access program. Subjects must have, at a minimum, clinical laboratory assessments, TTSTAND, and review of adverse events and concomitant medications at the scheduled Week 48 assessment time point to be eligible for continued vamorolone treatment in a general access program. These Week 48 assessments may be performed remotely if they cannot be performed on-site.

Subjects who complete the VBP15-004 study and will enroll directly into a general access program to continue vamorolone treatment may be discharged from the VBP15-004 study following completion of the modified Week 48 assessments.

14. Site Monitoring

In accordance with applicable regulations, GCP, and the procedures of the Sponsor or its designees, the Study Monitor will periodically contact the site to conduct monitoring reviews. During the COVID-19 pandemic, the extent, nature, and frequency of monitoring reviews will be largely based on the site's ability to accommodate on-site monitoring visits, site policies and local regulations regarding remote review of study data, and COVID-19 related travel restrictions. Additional factors that will be considered are enrollment rate and data quality at the site. It is expected that most monitoring during this period will be conducted remotely. Every attempt will be made to closely mirror the comprehensive nature of an on-site monitoring visit. Due to strict privacy laws in some countries, the only possible monitoring may be review of data in the electronic database. Through frequent communications (e.g., letter, e-mail, and telephone), the Study Monitor will ensure that the investigation continues to be conducted according to protocol and regulatory requirements.

During these contacts, the monitoring activities will include as many of the protocol-specified activities as possible (VBP15-004 Protocol, Section 11.5).

The Investigator will allow the Study Monitor direct access to all relevant documents as allowable by site policy and local regulations, and allocate his/her time and the time of his/her staff to the Study Monitor to discuss findings and any relevant issues.

226 **Table 1. Schedule of Study Activities**

227 **Assessments highlighted in the Table below are the critical safety and efficacy assessments which must be performed at the**
228 **specified assessment time points.**

	**Pretreatment Period**		**Treatment Period #1**							**Transition Period**		**Treatment Period #2**						**Dose-tapering Period**	
	**SCR**	**BL**																	
	**Day**			**Week**															
**Study Day or Week/Visit**	**-33 to -2**	**-1**	**1**	**2 (±1d)**	**6 (±3d)**	**12 (±1w)**	**18 (±1w)**	**24 (±1w)**	**24 (F/U)**	**26**	**28 (±1d)**	**28+1d**	**30 (±1d)**	**34 (±3d)**	**40 (±1w)**	**48 (±1w)**	**48 F/U**	**50**	**52 (±1d)**
Informed consent	X*																		
Enrollment	X*																		
Inclusion/exclusion criteria	X*	X*																	
Randomization	X*																		
Demographics	X*																		
Medical history	X*																		
Medication history	X*	X*																	
Physical examination	X*	X			X	X	X	X			X			X	X	X			X
Cushingoid features		X			X	X	X	X			X			X	X	X			X
Height	X*					X		X						X		X			
Weight	X*	X		X	X	X	X	X			X		X	X	X	X			X
Vital signs	X*	X	X	X	X	X	X	X			X		X	X	X	X			X
Blood for clinical labs	X*		X	X	X	X*	X	X*			X			X*	X*	X*			X*
Blood for HbA1c	X*							X								X			
Blood for vitamin D	X*					X		X					X		X	X			
Confirmation of varicella immunity	X*																		
Urinalysis	X*		X	X	X	X*	X	X*			X		X	X*	X*	X*			X*
Blood for serum PD biomarker panel			X			X		X			X				X	X			X
Fasting blood for insulin, glucose			X			X		X			X				X	X			X
Blood for DNA Testing								X											
ACTH Stimulation Test	X*								X								X		
Blood for Plasma PK													X						

	**Pretreatment Period**		**Treatment Period #1**							**Transition Period**		**Treatment Period #2**						**Dose-tapering Period**	
	**SCR**	**BL**																	
	**Day**			**Week**															
**Study Day or Week/Visit**	**-33 to -2**	**-1**	**1**	**2 (±1d)**	**6 (±3d)**	**12 (±1w)**	**18 (±1w)**	**24 (±1w)**	**24 (F/U)**	**26**	**28 (±1d)**	**28+1d**	**30 (±1d)**	**34 (±3d)**	**40 (±1w)**	**48 (±1w)**	**48 F/U**	**50**	**52 (±1d)**
12-lead ECG	**X***					X		X							X	X			
2D-echocardiogram	**X***							X								X			
Eye examination	**X***							X								X			
DXA scan	**X***							X								X			
Spine X-ray	**X***							X											
Fracture Questionnaire	**X***							X								X			
Dispense study medication			**X***		**X***	**X***	**X***		**X***		**X***			**X***	**X***		**X***		
Return study medication/ compliance monitoring				**X***	**X***	**X***	**X***	**X***			**X***		**X***	**X***	**X***	**X***			**X***
Study medication dosing			**X** →	→	→	→	→	**X**				**X** →	→	→	→	**X**			
Study medication dose tapering									**X** →	→	**X**						**X** →	→	**X**
Telephone call to subject										**X***								**X***	
Time to Stand Test (TTSTAND)	**X***	**X***			X	X		**X***						X	X	**X***			
Time to Climb Test (TTCLIMB)	**X***	X				X		X							X	X			
Time to Run/Walk Test (TTRW)	**X***	X				X		X							X	X			
NSAA	**X***	X				X		X							X	X			
Myometry (elbow flexors, knee extensors)	**X***	X				X		X							X	X			
Six-minute Walk Test (6MWT)	**X***	X				X		X							X	X			
Range of Motion (ROM) - ankles	**X***	X				X		X							X	X			
Pediatric Outcomes Data Collection Instrument (PODCI)	**X***							X								X			

	**Pretreatment Period**		**Treatment Period #1**							**Transition Period**		**Treatment Period #2**						**Dose-tapering Period**	
	**SCR**	**BL**																	
	**Day**			**Week**															
**Study Day or Week/Visit**	**-33 to -2**	**-1**	**1**	**2 (±1d)**	**6 (±3d)**	**12 (±1w)**	**18 (±1w)**	**24 (±1w)**	**24 (F/U)**	**26**	**28 (±1d)**	**28+1d**	**30 (±1d)**	**34 (±3d)**	**40 (±1w)**	**48 (±1w)**	**48 F/U**	**50**	**52 (±1d)**
Treatment Satisfaction Questionnaire (TSQM)								X								X			
PARS III	**X***					X		X								X			
Ease of Study Medication Administration Assessment				X		X		X					X		X	X			
Blindedness Assessment								X											
Dispense subject diaries			**X***	**X***	**X***	**X***	**X***	**X***			**X***		**X***	**X***	**X***	**X***			
Return subject diaries				**X***	**X***	**X***	**X***	**X***	**X***		**X***		**X***	**X***	**X***	**X***	**X***		X
AE/SAE recording	**X*** →																		→ **X***
Concomitant medications			**X*** →																→ **X***
Discharge from study																	**X***		**X***

229

Appendix 1. Remote Assessment of TTSTAND

REMOTE STAND FROM SUPINE (TTSTAND) MANUAL

VERSION 1.0 MARCH 20, 2020

Vamorolone Phase IIb Study

VBP15-004: A Phase IIb Randomized, Double-blind, Parallel Group, Placebo- and Active-controlled Study with Double-Blind Extension to Assess the Efficacy and Safety of Vamorolone in Ambulant Boys with Duchenne Muscular Dystrophy (DMD)

Contact Information:

Clinical Evaluator Questions:

Linda Johnson

ljohnson@trinds.com

01-916-479-4062

Michelle Eagle

Michelle.eagle@atominternational.org

44-754-005-1001

Note: This manual is to be used to perform the TTSTAND assessment remotely during COVID-19 pandemic.

Remote TTSTAND Manual Version 1.0 dated 23-Mar-2020

Reviewed and Approved By:

Eric Hoffman, Ph.D.<br>Vice President, Research<br>ReveraGen BioPharma, Inc.	23 March 2020<br>DATE
Linda Johnson<br>Clinical Evaluator Manager<br>TRiNDS, LLC	23 MAR 2020<br>DATE
Michelle Eagle<br>Clinical Evaluator Manager<br>ATOM International Limited	24 MAR 2020<br>DATE

General Testing Guidelines

- Only Time to Stand is to be performed remotely.
- TTSTAND testing should be performed at approximately the same time of day and after similar pre-test activities and routines.
    1. Site will arrange a testing time that corresponds to normal testing time during research appointments.
    2. The Site Coordinator or other site staff will arrange an appointment for the remote assessment of TTSTAND between the parents and Site CE using Zoom videoconferencing interface.
    3. During the Zoom session, the Site CE will explain the TTSTAND test to the child using the Zoom interface, and then carry out the assessment using video via Zoom, with remote timing of the test by the Site CE.
        - Parents, and site CE will confirm testing surface used during regular research visit, and confirm similar surface in participants home
        - Parents, and site CE will identify one family member/caregiver who will be present and participating in remote home visit. Other family members should not be present per protocol
    4. If Site CE is not available due to institutional employment restrictions during Covid19 limitation, central CE manager /trainer will perform testing.

- The same clinical evaluator (CE) should perform the strength and function testing at each visit if possible.
- If the TTSTAND must be repeated, a 60-second rest period is allowed, unless otherwise stated.
- To avoid bias, the CE should not review any previous strength and function results.
- The strength and function testing environment should be standardized. Since this is a remote visit, it is recognized that the environment may be different. Every effort should be made to use similar surface as in clinic, minimize distractions and limit family members present to essential personnel to assist with testing.
- The participant should wear loose clothing. It is preferable for the participant to wear a short sleeved shirt and shorts. This test is performed without shoes and socks.
- U.S. site only: If the participant has agreed and consented to participate in the companion community-based outcome measure (i.e. Actigraph) protocol, make sure the participant is wearing the actigraph during the strength and function testing.
- If the participant is unable to perform any of the assessments, the test should be skipped and re-introduced at the next visit. Proper documentation should be provided for why the assessment was not completed. Do not enter a score of '0' if test was not completed.
- Make note of start/stop times.
- Complete the timed tests using a manual stopwatch. For all timed tests, the CE will record time to complete the assessment and grade the quality of the movement.
- CEs may provide verbal instruction to improve quality of movement.
- CEs should try to obtain both time and quality assessment in one observation but may have the participant perform test twice, as necessary, to achieve best score in time and grading. For example, if the participant achieves a better time in trial 1 and grading from trial 2, the time for trial 1 and grading from trial 2 are the data points that should be recorded.
- The CE should explain the test item to the participant prior to having him perform the task (examiner is able to demonstrate the task first)
    1. CE will provide verbal instructions for test remotely to both participant and parent/caregiver.
    2. Parent/caregiver will demonstrate test as determined by CE

During the Session

1. At start of videochat session, the CE and parents make sure there is enough room to roll over and stand up.
2. CE will provide verbal instructions for test remotely to both participant and parent/caregiver prior to testing
3. Parent/caregiver will demonstrate test if determined necessary by CE to ensure entire test procedure is in view of camera
4. CE will give instructions for participant to obtain starting position via Zoom interface
5. CE will start stopwatch on the word 'go' and stop when participant is standing straight with arms at side.
6. Test will be repeated as necessary per protocol guidelines to obtain best score in time and grading
7. CE will complete source worksheets and add note on source to indicate that remote testing was done. A similar comment will be added to the eCRF.

The participant lays supine on the floor with their hands at their sides. They are asked to stand up as quickly as they can and return to a standing straight position with their hands at their sides. Make sure there is enough room to roll over and stand up. Furniture or external support is only used if needed. Do not use a mat unless absolutely necessary. Standard floor surface preferred.

**Preparation**	Participant lays supine on the floor with arms at sides
**Starting position**	Supine on floor with arms at sides
**Instructions**	When I say "GO" stand up from the floor as quickly and safely as you can, and stop by standing up straight with your arms by your side
**Timing**	Start the watch on the word "go" and stop it when the participant is standing straight with arms by his side
**Grading**	1. **Unable** to stand from supine, even with use of chair*<br>2. Assisted Gowers: requires **furniture or external support** for assist in arising from supine to full upright posture*<br>3. Rolls over, stands up with **TWO hands** "climbing up" the legs to achieve full upright posture.<br>4. Rolls over, stands up with **ONE hand** support on leg<br>5. Rolls to the side and stands up with one or both hands on the floor to start to rise but **does not touch legs**.<br>6. Stands up **without** rolling over or using hands on legs<br><br>*A time will not be recorded if the functional grade is 1 or 2.

Remote TTSTAND Manual Version 1.0 dated 23-Mar-2020
