# Supplementary material for: Efficacy and Safety of Vamorolone vs Placebo and Prednisone Among Boys With Duchenne Muscular Dystrophy: A Randomized Clinical Trial
Source: JAMA Neurol. 2022 Aug 29;79(10):1005–14. doi: 10.1001/jamaneurol.2022.2480 (PMC9425287; doi:10.1001/jamaneurol.2022.2480)
Supplement: Supplement 7. — Statistical Analysis Plan [file jamaneurol-e222480-s007.pdf]

ReveraGen BioPharma, Inc.

STATISTICAL ANALYSIS PLAN
for Protocol VBP15-004 Treatment Period #1

**Protocol Title:**	A Phase IIb Randomized, Double-blind, Parallel Group, Placebo- and Active-controlled Study with Double-Blind Extension to Assess the Efficacy and Safety of Vamorolone in Ambulant Boys with Duchenne Muscular Dystrophy (DMD)
**Protocol Number**	VBP15-004
**Phase:**	Phase IIb
**Sponsor:**	ReveraGen BioPharma, Inc.<br>155 Gibbs Street<br>Suite 433<br>Rockville, MD 20850
**Author:**	Summit Analytical, LLC<br>8354 Northfield Blvd., Bldg. G Suite 3700<br>Denver, CO 80238
**SAP Date:**	17 May 2021
**Status:**	Final Version 3.0

12

Statistical Analysis Plan Approval Form

Sponsor: ReveraGen BioPharma, Inc.
Protocol: VBP15-004
Protocol Title: A Phase IIb Randomized, Double-blind, Parallel Group, Placebo- and Active-controlled Study with Double-Blind Extension to Assess the Efficacy and Safety of Vamorolone in Ambulant Boys with Duchenne Muscular Dystrophy (DMD)
SAP Version: Final v3.0
SAP Date: 17 May 2021

The statistical analysis plan has been reviewed and approved.

Sponsor: *ReveraGen BioPharma, Inc.*
Eric Hoffman, PhD
CEO

Signature Date

Author: *Summit Analytical LLC*
Philip Shale, PhD
Biostatistics

Signature Date

Reviewer: *Santhera Pharmaceuticals*
Mika Leinonen
Head Biostatistics

Signature Date

Reviewer: *The Camden Group LLC*
Benjamin D. Schwartz, MD, PhD
Consulting Medical Monitor

Signature Date

1. TABLE OF CONTENTS

1. TABLE OF CONTENTS 3
2. LIST OF ABBREVIATIONS AND DEFINITION OF TERMS 6
3. INTRODUCTION 8
3.1. Preface 8
3.2. Purpose of Analyses 8
3.3. Summary of Statistical Analysis Changes to the Protocol 9
4. STUDY OBJECTIVES AND ENDPOINTS 11
4.1. Study Objectives 11
4.1.1. Primary Objectives 11
4.1.2. Secondary Objectives 11
4.1.3. Exploratory Objectives 11
4.2. Study Endpoints 12
4.2.1. Safety Endpoints 12
4.2.2. Tolerability Endpoint 13
4.2.3. Efficacy Endpoints 13
4.2.4. Exploratory Patient-reported Outcomes Endpoints 14
4.2.5. Pharmacodynamic Endpoints 15
5. STUDY METHODS 16
5.1. General Study Design and Plan 16
5.2. Inclusion – Exclusion Criteria and General Study Population 22
5.3. Randomization and Blinding 22
5.4. Analysis Variables 22
6. SAMPLE SIZE 23
7. GENERAL CONSIDERATIONS 25
7.1. Analysis Populations 25
7.1.1. Safety Population 25
7.1.2. Modified Intent-to-Treat (mITT) Population 25
7.1.3. Per Protocol Population 25
7.2. Covariates and Subgroups 26
7.2.1. Planned Covariates 26
7.2.2. Subgroups 26
7.3. Management of Analysis Data 26
7.3.1. Data Handling 26
7.3.2. Missing Data 26
7.3.3. Handling of Early Termination Visit Information 32
7.3.4. Pooling of Investigational Sites 32
7.3.5. Coding Conventions for Events and Medications 32
7.3.6. Baseline Visits 32
7.3.7. Calculating Derived Variables 32
7.3.8. Analysis Software 37
7.3.9. Study Data 37
7.3.10. Period #1 Data Management Study Data Cutoff 38
7.4. General Statistical Methodology 39
7.4.1. Statistical Summaries: Descriptive and Inferential 39

7.4.2. Interim Analyses and Data Monitoring 39
7.4.3. Week 24 Analysis 40
7.4.4. Week 48 Analysis 40
7.4.5. Multiple Testing Procedures 40
7.5. Summary of Study Data 41
7.5.1. Subject Summary Grouping 41
7.5.2. Study Disposition 41
7.5.3. Protocol Deviations 41
7.5.4. Demographics and Baseline Characteristics 41
7.5.5. Medical History 42
7.5.6. Prior and Concomitant Medications 42
7.5.7. Treatment Compliance and Study Drug Exposure 43
7.6. Efficacy Analyses 43
7.7. Safety Analyses 48
7.7.1. Adverse Events 48
7.7.2. Vital Signs, 12-Lead ECG, and Laboratory Outcomes 50
7.7.3. Physical Examination 51
7.7.4. Other Safety Measures 51
7.8. Exploratory Analyses 52
7.9. Pharmacodynamic (PD) Serum and Other Biomarkers 52
8. REPORTING CONVENTIONS 54
8.1. General Reporting Conventions 54
8.2. Population Summary Conventions 55
9. REFERENCES 57
10. APPENDICES 59
10.1. List of Tables, Listings, and Figures 59
10.2. SAP Amendment Summary of Important Changes 59

LIST OF TABLES

Table 1 Study Randomization Schedule 16
Table 2 Schedule of Study Activities 17

LIST OF FIGURES

Figure 1 SDTM, ADaM, and TFL Development and Validation 38

2. LIST OF ABBREVIATIONS AND DEFINITION OF TERMS

Only abbreviations and terms relevant to the SAP are repeated herein. The reader is referred to the protocol for the complete and comprehensive list of abbreviations and definitions of terms for the study.

ACTH	adrenocorticotropic hormone
AE	adverse event
ANCOVA	analysis of covariance
BL	baseline
BMC	bone mineral content
BMI	body mass index
CINRG	Cooperative International Neuromuscular Research Group
CK	creatine kinase
cm	centimeter
CTCAE	Common Terminology Criteria for Adverse Events
CTX	carboxy-terminal telopeptide
CV	coefficient of variation
dL	deciliter
DMD	Duchenne muscular dystrophy
DSMB	data and safety monitoring board
ECG	electrocardiogram
eCRF	electronic case report form
EDC	electronic data capture
F/U	follow-up
g	gram
GLDH	glutamate dehydrogenase
HbA1c	hemoglobin A1c
HDL	high density lipoprotein
ICH	International Conference on Harmonisation
IND	investigational new drug
L	Liter
LLC	limited liability company
LDL	low density lipoprotein
LS	least squares
m	meter

MAR	missing at random
MCMC	Markov Chain Monte Carlo
MedDRA	Medical Dictionary for Regulatory Activities
mg	milligram
MI	multiple imputation
mL	milliliter
MMRM	mixed model for repeated measures
MNAR	missing not at random
No., n	number
NSAA	North Star Ambulatory Assessment
%CV	percentage coefficient of variation
PD	pharmacodynamic(s)
P1NP	serum aminoterminal propeptide of type I collagen
PK	pharmacokinetic(s)
PMM	pattern mixture model
PODCI	Pediatric Outcomes Data Collection Instrument
6MWT	six-minute walk test
REML	restricted maximum likelihood
ROM	range of motion (test)
SAE	serious adverse event
SAP	statistical analysis plan
SCR	screening
SD	standard deviation
SDTM	Study Data Tabulation Model
SOC	system organ class
TEAE	treatment-emergent adverse event
TSQM	Treatment Satisfaction Questionnaire
TTCLIMB	time to climb (test)
TTSTAND	time to stand (test)
TTRW	time to run/walk (test)
vs.	versus
WHO	World Health Organization

102

3. INTRODUCTION

3.1. Preface

This document presents a statistical analysis plan (SAP) for Treatment Period #1 of ReveraGen BioPharma, Inc. Protocol VBP15-004, (*A Phase IIb Randomized, Double-blind, Parallel Group, Placebo- and Active-controlled Study with Double-Blind Extension to Assess the Efficacy and Safety of Vamorolone in Ambulant Boys with Duchenne Muscular Dystrophy (DMD)*). This SAP will provide the details and methods for analysis and reporting of the subject characteristics, safety, and efficacy information for data captured during Period #1 of this study, i.e. through the Week 24 Follow-up (F/U) Visit. A separate SAP will be developed for the data captured during the entire study (Period #1, Transition Period, and Period #2) (see Section 3.2 of this SAP).

Reference materials for this SAP include the protocol VBP15-004 (Amendment #4 Dated: 28 August 2020).

The SAP described herein is an *a priori* plan. The SAP will be finalized and approved prior to database lock and unblinding. Statistical programming may occur as study data accumulate in order to have analysis programs ready at the time the study finishes.

As divergent recommendations on the analysis plans for the VBP15-004 study were received by FDA and EMA, this version of the SAP is specifically designed for data analysis in line with FDA recommendation for analysis of Period #1 data. The separate SAP, as mentioned above, will be developed to accommodate recommendations specified by EMA, in addition to describing the data analysis of the entire study.

For publication and other commercial purposes of the Period #1 VBP15-004 study data, the primary analysis as specified in this SAP will be considered the primary analysis of the study.

3.2. Purpose of Analyses

The purposes of the planned analyses described in this SAP are to assess the safety, tolerability, pharmacodynamics, and efficacy of two oral doses of vamorolone (2.0 mg/kg/day and 6.0 mg/kg/day) in ambulant boys ages 4-< 7 years with DMD. Safety and efficacy endpoints (clinical and biomarker) will be compared to a placebo group, and to a group treated with oral prednisone (0.75 mg/kg/day).

The study has four periods: Treatment Period #1, a Transition Period, Treatment Period #2, and a Dose-tapering Period applicable to a subset of subjects.

- Period #1 is a double-blind, placebo-controlled, prednisone-controlled 24-week treatment period;

- The Transition Period is a 4-week double-blind period during which all subjects taper off their placebo or prednisone tablets; and
- Period #2 is a double-blind 20-week treatment period during which all subjects are treated with vamorolone 2.0 mg/kg/day or 6.0 mg/kg/day; and
- The Dose-tapering Period is a 4-week double-blind period during which subjects who choose not to receive further vamorolone treatment by enrolling directly into a subsequent vamorolone general access program will have their dose of vamorolone suspension tapered and discontinued.

This SAP is relevant to Period #1, the double-blind, placebo-controlled, prednisone-controlled part of the study, with the clinical study report corresponding to the initial 24-week Period #1. Results from the analyses completed will be included in the Period #1 24-week clinical study report for VBP15-004, and may also be utilized for regulatory submissions, manuscripts, additional endpoint specific reports, or other clinical development activities.

A separate SAP and clinical study report will be issued for the entire VBP15-004 study, including the initial 24-week Period #1, the Transition Period (Weeks 25-28), and the second 20-week Period #2 (when all subjects are treated with vamorolone). The separate SAP for the entire VBP15-004 study will be finalized before unblinding of data from Period #1. Furthermore, the separate SAP defines the analyses as recommended by the EMA. The two SAPs of this study as consistent with each other regarding the analyses specific to Period #1, including the analysis of the primary and secondary efficacy endpoints (with the exception of which of the analyses is considered as primary by FDA and EMA, respectively).

Post-hoc exploratory analyses not identified in this SAP may be performed to further examine the study data and provide context for study results. These analyses will be clearly identified, where appropriate, in the final clinical study report.

Additional analyses not prospectively identified in this SAP may also be completed for publications, additional endpoint specific reports, regulatory, or funding inquiries. These analyses, if performed, may not be reported in the clinical study report, but will be fully documented in the document containing the additional analyses.

3.3. Summary of Statistical Analysis Changes to the Protocol

The analyses described in this analysis plan are consistent with the analyses described in the study protocol with the exception of those items noted in this section.

Spine x-ray analysis is discussed in the protocol. It will be presented in a separate SAP and is not included in this SAP.

Analysis of DNA testing (genetic modifiers) is described as an additional exploratory endpoint in the protocol. However, the DNA samples will be stored for later testing after completion of the 48-week trial and are not analyzed in this SAP.

Exploratory biomarkers mentioned in the protocol will be tested and described in a later report, with samples stored and studied later and are not included in this SAP.

4. STUDY OBJECTIVES AND ENDPOINTS

Study objectives and endpoints defined in the protocol include safety, tolerability, pharmacokinetics, pharmacodynamics, and efficacy endpoints. Objectives and pre-specified endpoints for Treatment Period #1 are as follows:

4.1. Study Objectives

4.1.1. Primary Objectives

The primary objectives of this study are:

1. To compare the efficacy of vamorolone administered orally at daily doses of 6.0 mg/kg over a 24-week treatment period vs. placebo in ambulant boys ages 4 to <7 years with DMD; and
2. To evaluate the safety and tolerability of vamorolone administered orally at daily doses of 2.0 mg/kg and 6.0 mg/kg in ambulant boys ages 4 to <7 years with DMD.

4.1.2. Secondary Objectives

The secondary objectives of this study are:

1. To compare the efficacy of vamorolone administered orally at daily doses of 2.0 mg/kg over a 24-week treatment period vs. placebo in ambulant boys ages 4 to <7 years with DMD;
2. To compare the safety of vamorolone administered orally at daily doses of 2.0 mg/kg and 6.0 mg/kg over a 24-week treatment period vs. daily prednisone 0.75 mg/kg in ambulant boys ages 4 to <7 years with DMD; and
3. To compare the efficacy of vamorolone administered orally at daily doses of 2.0 mg/kg and 6.0 mg/kg over a 24-week treatment period vs. daily prednisone 0.75 mg/kg in ambulant boys ages 4 to <7 years with DMD.

4.1.3. Exploratory Objectives

The exploratory objectives of this study are:

1. To evaluate the satisfaction with treatment of vamorolone administered orally at daily doses of 2.0 mg/kg and 6.0 mg/kg over a 24-week treatment period vs. daily prednisone 0.75 mg/kg in ambulant boys ages 4 to <7 years with DMD;
2. To evaluate the effect of vamorolone administered orally at daily doses of 2.0 mg/kg and 6.0 mg/kg over a 24-week treatment period vs. daily prednisone 0.75 mg/kg on behaviour and neuropsychology;
3. To evaluate the effect of vamorolone administered orally at daily doses of 2.0 mg/kg and 6.0 mg/kg over a 24-week treatment period vs. placebo on physical functioning.
4. To assess the ease of administration of the study medication suspension to ambulant boys ages 4 to <7 years with DMD;
5. To compare the effects of vamorolone administered orally at daily doses of 2.0 mg/kg and 6.0 mg/kg over a 24-week treatment period vs. placebo on potential serum

pharmacodynamics (PD) biomarkers of safety and efficacy in ambulant boys ages 4 to <7 years with DMD;

6. To compare the effects of vamorolone administered orally at daily doses of 2.0 mg/kg and 6.0 mg/kg over a 24-week treatment period vs. daily prednisone 0.75 mg/kg on potential serum PD biomarkers of safety and efficacy in ambulant boys ages 4 to <7 years with DMD; and
7. To determine if candidate genetic modifiers of DMD (gene polymorphisms associated with disease severity, or response to glucocorticoid treatment) are similarly associated with vamorolone-treated DMD subjects (baseline disease severity, or response to vamorolone or prednisone treatment).

4.2. Study Endpoints

4.2.1. Safety Endpoints

1. Linear growth velocity: Comparison of each vamorolone dose level group with the prednisone group in change in height percentile for age from baseline;
2. Adrenal suppression: Comparison of each vamorolone dose level group with the prednisone group and of vamorolone 2.0 mg/kg/day vs. vamorolone 6.0 mg.kg/day in % of subjects with peak cortisol below 18 µg/dL (500 nM) in the ACTH stimulation test at Week 24 at 30 or 60 minutes after stimulation with Cosyntropin;
3. Cushingoid features: Comparison of each vamorolone dose level group with the prednisone group in change from baseline to each of the scheduled on-treatment and post-treatment assessment time points (changes from baseline will be recorded as AEs);
4. Dual-energy x-ray absorptiometry (DXA) scan: Comparison of both vamorolone dose level groups with the prednisone group:
   - Percent change from baseline to Week 24 in spine BMD (g/cm2)
   - Percent change from baseline to Week 24 in total body BMD (g/cm2)
   - Percent change from baseline to Week 24 spine and total body bone mass (bone mineral content, g)
   - Percent change from baseline to Week 24 in total body composition (lean mass (g), fat mass (g), fat-free mass (g), Lean Mass Index (kg/m2), and Fat Mass Index (kg/m2));
5. BMI Z-score: Comparison of each vamorolone dose level group with the prednisone group in change from baseline;
6. Treatment-emergent adverse events (TEAEs) and serious adverse events (SAEs) by system organ class (SOC): Overall by treatment, by treatment and relationship, and by treatment and intensity (see protocol Section **Error! Reference source not found.**);
7. Vital signs (sitting blood pressure, heart rate, respiratory rate, and body temperature): Change from baseline to each of the scheduled on-treatment and post-treatment assessment time points;

8. Body weight, height, and BMI (kg/m^2): Change from baseline to each of the scheduled on-treatment and post-treatment assessment time points;
9. Clinical laboratory values: Change from baseline to each of the scheduled on-treatment and post-treatment assessment time points in:
    - Hematology and clinical chemistry
    - Lipid profile (triglycerides, total cholesterol, low density lipoprotein [LDL], high density lipoprotein [HDL])
    - Vitamin D level
    - Urinalysis;
10. 12-lead electrocardiogram (ECG): Change from baseline to each of the scheduled on-treatment and post-treatment assessment time points;
11. 2D-echocardiogram: Change from baseline to Week 24;
12. Eye examination for detection of clinically significant abnormalities (cataracts and/or glaucoma) at the Week 24 assessment compared to baseline.

Data for the following additional safety outcomes will be listed only:

1. Physical examination findings at each of the pretreatment, on-treatment, and post-treatment assessment time points;
2. Fracture Questionnaire results at pretreatment and Week 24.

4.2.2. Tolerability Endpoint

1. Premature discontinuations of study treatment due to adverse events.

4.2.3. Efficacy Endpoints

4.2.3.1. Primary Efficacy Endpoint

TTSTAND velocity (rise/second): Comparison of vamorolone 6.0 mg/kg/day dose level group versus the placebo group in change from baseline to the Week 24 assessment. The velocity will be calculated as defined in Section 7.3.7.

4.2.3.2. Secondary Efficacy Endpoints (by fixed sequential testing following successful statistical testing of the primary efficacy endpoint)

Change from baseline to Week 24 for the following comparisons:

- TTSTAND velocity, vamorolone 2.0 mg/kg/day vs. placebo
- 6 Minute Walk Test (6MWT) meters walked, vamorolone 6.0 mg/kg/day vs. placebo
- 6 Minute Walk Test (6MWT) meters walked, vamorolone 2.0 mg/kg/day vs. placebo
- Time to Run/Walk (TTRW) 10 meters velocity, vamorolone 6.0 mg/kg/day vs. placebo
- Time to Run/Walk (TTRW) 10 meters velocity, vamorolone 2.0 mg/kg/day vs. placebo

- 6MWT meters walked, vamorolone 6.0 mg/kg/day vs. prednisone
- 6MWT meters walked, vamorolone 2.0 mg/kg/day vs. prednisone

4.2.3.3. Exploratory Efficacy Endpoints

Change from baseline to each of the scheduled study assessment time points up to and including Week 24 for the following comparisons:

- TTSTAND velocity, vamorolone 6.0 mg/kg/day vs. placebo (Week 6 and 12 only)
- TTSTAND velocity, vamorolone 2.0 mg/kg/day vs. placebo (Week 6 and 12 only)
- 6 Minute Walk Test (6MWT) meters walked, vamorolone 6.0 mg/kg/day vs. placebo (Week 12 only)
- 6 Minute Walk Test (6MWT) meters walked, vamorolone 2.0 mg/kg/day vs. placebo (Week 12 only)
- TTRW 10 meters velocity, vamorolone 6.0 mg/kg/day vs. placebo (Week12 only)
- TTRW 10 meters velocity, vamorolone 2.0 mg/kg/day vs. placebo (Week12 only)
- 6MWT meters walked, vamorolone 6.0 mg/kg/day vs. prednisone (Week 12 only)
- 6MWT meters walked, vamorolone 2.0 mg/kg/day vs. prednisone (Week 12 only)
- NSAA total score, vamorolone 6.0 mg/kg/day vs. placebo
- NSAA total score, vamorolone 2.0 mg/kg/day vs. placebo
- Hand-held Myometry knee extensors, vamorolone 6.0 mg/kg/day vs. placebo
- Hand-held Myometry knee extensors, vamorolone 2.0 mg/kg/day vs. placebo
- Hand-held Myometry elbow flexors, vamorolone 6.0 mg/kg/day vs. placebo
- Hand-held Myometry elbow flexors, vamorolone 2.0 mg/kg/day vs. placebo
- Time to Climb (TTCLIMB) velocity, vamorolone 6.0 mg/kg/day vs. placebo
- Time to Climb (TTCLIMB) velocity, vamorolone 2.0 mg/kg/day vs. placebo
- Range of Motion (ROM) in the ankles, vamorolone 6.0 mg/kg/day vs. placebo
- Range of Motion (ROM) in the ankles, vamorolone 2.0 mg/kg/day vs. placebo

4.2.4. Exploratory Patient-reported Outcomes Endpoints

1. Treatment satisfaction questionnaire (TSQM): Comparison of each vamorolone dose level group to the prednisone group at the Week 24 visit;
2. Pediatric Outcomes Data Collection Instrument (PODCI): Comparison of each vamorolone dose level group to the placebo group for change from baseline to the Week 24 assessment;
3. Behavioral changes (PARS III): Comparison of each vamorolone dose level group to the prednisone group and to the placebo group for change from baseline to each of the scheduled study assessment time points up to the Week 24 assessment for four PARS III subscores for peer relations, dependency, anxiety and depression, and withdrawal;
4. Ease of study medication administration (Question 1 only; tablet vs. liquid) assessed at each of the scheduled study assessment time points; and

5. Blindedness Assessment at each of the scheduled study assessment time points.

4.2.5. Pharmacodynamic Endpoints

1. The following pharmacodynamic biomarkers are considered secondary outcome measures focusing on safety outcomes. In each case, the biomarkers studied reflect safety concerns of glucocorticoids:
    a. Adrenal suppression. First-in-morning serum cortisol levels (fasting and non-fasting) will be measured. Cortisol measures falling below 3.6 µg/dL (or 100 nM) will be considered to be indicative of the development of adrenal suppression. ACTH Stimulation Test will be performed at the Screening Visit and at the Week 24 Follow-up Visit (48 ± 3 hours after the final dose of Treatment Period #1 study medication): peak cortisol levels <18 µg/dL (or 500 nM) 30 or 60 minutes after stimulation with Cosyntropin (250 µg) will be considered to be indicative of adrenal suppression, where peak is the higher of the 30- and 60-minute assessments.
    b. Bone turnover. Measures of serum osteocalcin are reflective of bone formation, and measures of serum CTX1 are reflective of bone resorption. Levels of osteocalcin and CTX1 predict later clinical safety concerns of osteopenia and bone fragility.
    c. Insulin resistance. Glucocorticoids cause both acute and chronic insulin resistance, with serum elevations of both insulin and glucose. Measures of hyperinsulinemia and hyperglycemia are accepted measures of insulin resistance.
2. Exploratory biomarkers for aspects of safety and efficacy will be studied and reported at a later date.

5. STUDY METHODS

5.1. General Study Design and Plan

As background for the statistical methods presented below, this section provides an overview of the study design and plan of study execution. The protocol is the definitive reference for all matters discussed in what follows.

This Phase IIb study is a randomized, double-blind, parallel group, placebo- and active-controlled study with double-blind extension to evaluate the long-term efficacy, safety, tolerability, PD, and population PK of vamorolone (the investigational medicine) compared to prednisone (active control) and placebo over a Treatment Period of 24 weeks in boys ages 4 to <7 years with DMD (Period #1), and determine the persistence of effect over a total Treatment Period of 48 weeks. Six treatment groups will receive either vamorolone at one of two doses (2.0 mg/kg or 6.0 mg/kg), prednisone (0.75 mg/kg), or placebo once daily for 24 weeks, and will receive vamorolone at one of two doses (2.0 mg/kg or 6.0 mg/kg) daily for an additional 20 weeks (Period #2). A total of approximately 120 subjects will be randomized into the study as shown in Table 11.

Table 1 Study Randomization Schedule

Group	Planned Number of Subjects	Treatment Period #1 (24 Weeks)	Treatment Period #2 (20 Weeks)
1	30	Vamorolone, 2.0 mg/kg/day →	Vamorolone, 2.0 mg/kg/day
2	30	Vamorolone, 6.0 mg/kg/day →	Vamorolone, 6.0 mg/kg/day
3	15	Prednisone, 0.75 mg/kg/day →	Vamorolone, 2.0 mg/kg/day
4	15	Prednisone, 0.75 mg/kg/day →	Vamorolone, 6.0 mg/kg/day
5	15	Placebo →	Vamorolone, 2.0 mg/kg/day
6	15	Placebo →	Vamorolone, 6.0 mg/kg/day

Error! Reference source not found. presents the schedule of study procedures.

Table 2 **Schedule of Study Activities**

	Pretreatment Period		Treatment Period #1							Transition Period		Treatment Period #2						Dose-tapering Period	
	SCR	BL																	
	Day			Week															
**Study Day or Week/Visit**	**-33 to -2[a]**	**-1[b]**	**1[c]**	**2 (±1d)[d]**	**6 (±3d)[d]**	**12 (±1w)[d]**	**18 (±1w)[d]**	**24[e] (±1w)[d]**	**24 (F/U)[e]**	**26**	**28[f] (±1d)[d]**	**28+1d**	**30 (±1d)[d]**	**34 (±3d)[d]**	**40 (±1w)[d]**	**48[g] (±1w)[d]**	**48 F/U[g]**	**50**	**52[h] (±1d)[d]**
Informed consent	X																		
Enrollment[i]	X																		
Inclusion/exclusion criteria	X	X[j]																	
Randomization[k]	X																		
Demographics	X																		
Medical history	X																		
Medication history	X	X																	
Physical examination	X	X			X	X	X	X			X			X	X	X			X
Cushingoid features		X			X	X	X	X			X			X	X	X			X
Height	X					X		X						X		X			
Weight	X	X		X	X	X	X[l]	X			X		X	X	X[l]	X			X
Vital signs[m]	X	X	X[n]	X	X	X	X	X			X		X	X	X	X			X
Blood for clinical labs[o]	X		X[p]	X[p]	X	X[p]	X	X[p]			X[p]		X[p]	X	X[p]	X[p]			X[p]
Blood for HbA1c[o]	X							X								X			
Blood for vitamin D[o]	X					X		X							X	X			
Confirmation of varicella immunity	X																		
Urinalysis[q]	X		X[p]	X[p]	X	X[p]	X	X[p]			X[p]		X[p]	X	X[p]	X[p]			X[p]
Blood for serum PD biomarker panel[r,s]			X			X		X			X				X	X			X
Fasting blood for insulin, glucose[s]			X			X		X			X				X	X			X
Blood for DNA Testing								X											
ACTH Stimulation Test	X								X[t]								X[t]		
Blood for Plasma PK													X[u]						
12-lead ECG[v]	X					X		X							X	X			
2D-echocardiogram	X							X								X			

	Pretreatment Period		Treatment Period #1							Transition Period		Treatment Period #2						Dose-tapering Period	
	SCR	BL																	
	Day			Week															
**Study Day or Week/Visit**	**-33 to -2[a]**	**-1[b]**	**1[c]**	**2 (±1d)[d]**	**6 (±3d)[d]**	**12 (±1w)[d]**	**18 (±1w)[d]**	**24[e] (±1w)[d]**	**24 (F/U)[e]**	**26**	**28[f] (±1d)[d]**	**28+1d**	**30 (±1d)[d]**	**34 (±3d)[d]**	**40 (±1w)[d]**	**48[g] (±1w)[d]**	**48 F/U[g]**	**50**	**52[h] (±1d)[d]**
Eye examination	X							X								X			
DXA scan	X							X								X			
Spine X-ray	X							X											
Fracture Questionnaire	X							X								X			
Dispense study medication			X		X	X	X		X		X			X	X		X		
Return study medication/ compliance monitoring				X[w]	X	X	X	X			X		X[w]	X	X	X			X
Study medication dosing[x]			X →	→	→	→	→	X				X →	→	→	→	X			
Study medication dose tapering									X[y] →	→	X						X →	→	X
Telephone call to subject[z]										X								X	
Time to Stand Test (TTSTAND)	X	X			X	X		X						X	X	X			
Time to Climb Test (TTCLIMB)	X	X				X		X							X	X			
Time to Run/Walk Test (TTRW)	X	X				X		X							X	X			
NSAA[aa]	X	X				X		X							X	X			
Myometry (elbow flexors, knee extensors)	X	X				X		X							X	X			
Six-minute Walk Test (6MWT)	X	X				X		X							X	X			
Range of Motion (ROM) – ankles	X	X				X		X							X	X			
Pediatric Outcomes Data Collection Instrument (PODCI)	X							X								X			
Treatment Satisfaction Questionnaire (TSQM)								X								X			

	Pretreatment Period		Treatment Period #1							Transition Period		Treatment Period #2						Dose-tapering Period	
	SCR	BL																	
	Day			Week															
**Study Day or Week/Visit**	**-33 to -2[a]**	**-1[b]**	**1[c]**	**2 (±1d)[d]**	**6 (±3d)[d]**	**12 (±1w)[d]**	**18 (±1w)[d]**	**24[e] (±1w)[d]**	**24 (F/U)[e]**	**26**	**28[f] (±1d)[d]**	**28+1d**	**30 (±1d)[d]**	**34 (±3d)[d]**	**40 (±1w)[d]**	**48[g] (±1w)[d]**	**48 F/U[g]**	**50**	**52[h] (±1d)[d]**
PARS III	X					X		X								X			
Ease of Study Medication Administration Assessment[bb]				X		X		X					X		X	X			
Blindedness Assessment								X											
Dispense subject diaries[cc]			X	X	X	X	X	X			X		X	X	X	X			
Return subject diaries				X	X	X	X	X	X[dd]		X		X	X	X	X	X[ee]		X
AE/SAE recording[ff]	X	→	→	→	→	→	→	→	→	→	→	→	→	→	→	→	→	→	X[gg]
Concomitant medications			X	→	→	→	→	→	→	→	→	→	→	→	→	→	→	→	X
Discharge from study																	X[hh]		X[ii]

BL = Baseline; d = day(s); F/U = Follow-up; SCR = Screening; w = week.

a. The Pretreatment Screening Period spans Day -33 through Day -2, but all screening procedures must be completed by Day -11. Subjects meeting all eligibility criteria will be randomized by Day -11, at least 10 days prior to the Baseline Day -1 Visit.

b. Baseline Day -1, within 24 hours prior to administration of the first dose of study drug.

c. Treatment Day 1 begins at the time of administration of the first dose of study medication in the clinic.

d. Time windows around the Week 2, Week 6, Week 12, Week 18, and Week 24 Visits are allowances from date of Day 1 Visit. Time window around the Week 28 Visit is allowance from date of Week 24 F/U Visit. Time windows around the Week 30, Week 34, Week 40, and Week 48 Visits are allowances from date of Week 28+1d Visit. Time window around the Week 52 Visit is allowance from date of Week 48 F/U Visit.

e. Subjects who prematurely discontinue from the study prior to Week 24 should complete the Week 24 assessments and the Week 24 Follow-up Visit ACTH Stimulation Test at the time of early withdrawal and undergo Early Discontinuation Dose-tapering, where possible (see protocol Section **Error! Reference source not found.** and Section **Error! Reference source not found.**). The Week 24 Follow-up Visit must occur to allow the ACTH Stimulation Test to be performed 48 ± 3 hours after the final dose of Treatment Period #1 study medication.

f. Subjects who prematurely discontinue from the study after Week 24 but prior to Week 28 should complete the Week 28 assessments, and undergo Early Discontinuation Dose-tapering, where possible (see protocol Section **Error! Reference source not found.**).

g. Subjects who prematurely discontinue from the study after Week 28 but prior to Week 48 should complete the Week 48 assessments and the Week 48 Follow-up Visit ACTH Stimulation Test at the time of early withdrawal and undergo Dose-tapering, where possible (see protocol Section **Error! Reference source not found.** and Section **Error! Reference source not found.**). The Week 48 Follow-up Visit must occur to allow the ACTH Stimulation Test to be performed 48 ± 3 hours after the final dose of Treatment Period #2 study medication.

h. Subjects will have one study site visit during the Dose-tapering Period, at one week after the dose of liquid formulation has been discontinued (Week 52) (see protocol Section **Error! Reference source not found.**).

i. Subjects are considered to be enrolled in the study at the time written informed consent is obtained.

j. Study eligibility should be rechecked and confirmed at Baseline Day -1 Visit.

k. Randomization occurs by Interactive Voice/Web Response System (IXRS) after subjects are confirmed to have met all study entry criteria, at least 10 days prior to the Baseline Day -1 Visit.

l. Weight recorded at the Week 18 Visit and the Week 40 Visit will be used to calculate doses for study drug dispensed at the Week 24 Follow-up and Week 48 Follow-up Visits, respectively.

m. Sitting blood pressure, body temperature, respiratory rate, and heart rate.

n. Vital signs recorded prior to administration of the first dose of study drug at the Day 1 Visit.

o. Blood for hematology, chemistry, and lipids, including HbA1c and Vitamin D where applicable.

p. Blood samples (collected after subjects have fasted for ≥ 6 hours) and urine collected at scheduled visit, and prior to dose of study drug where applicable.

q. Urinalysis by dipstick and microscopic analysis.

r. Blood collected for PD biomarkers includes secondary safety outcomes (morning cortisol, osteocalcin, CTX1, P1NP), and exploratory safety and efficacy PD biomarkers.

s. Blood samples for PD biomarkers and fasting glucose and insulin determination will be collected after subjects have fasted for ≥ 6 hours, prior to the daily dose of study medication where applicable.

t. Subjects will return to the study site for the Week 24 Follow-up Visit for an ACTH Stimulation Test 48 hours ± 3 hours after administration of the final dose of Treatment Period #1 study medication, and for the Week 48 Follow-up Visit for an ACTH Stimulation Test 48 hours ± 3 hours after administration of the final dose of Treatment Period #2 study medication (see protocol Section **Error! Reference source not found.**).

u. Blood sample for population PK analysis will be collected 2 hours after administration of the daily dose of study medication.

v. 12-lead ECG recorded after subject has rested quietly in a supine position for at least 5 minutes.

w. Study medication brought by subjects to the Week 2 Visit and Week 30 Visit for dosing and compliance assessment will be redispensed to subjects at the end of the visit.

x. The dose of study medication on the days of the Day 1, Week 2, Week 12, Week 24, Week 28, Week 30, Week 40, and Week 48 Visits will be administered after 1) fasting blood draws; and 2) breakfast provided by the study site. All other doses will be taken at home. See protocol Section **Error! Reference source not found.** for other Day 1 pre-dose safety assessments.

y. Doses of tablet study drug will be tapered and suspension study drug will be continued, during Weeks 24-28.

z. Site study staff will contact the parent(s)/guardian(s) by telephone at Weeks 26 and 50 to ensure that the study drug tapering is proceeding according to protocol, to assess potential signs or symptoms indicative of adrenal suppression, and to address any questions the parent(s)/guardian(s) may have.

aa. North Star Ambulatory Assessment; includes the Time to Stand Test (TTSTAND).

bb. Ease of Study Medication Administration assessed at the Weeks 2, 12, and 24, 30, 40, and 48 Visits.

cc. Subject diaries used to record any changes in concomitant medications taken, any AEs experienced during the study, and any incomplete or missed doses of study medication.

dd. Subject diaries dispensed at the Week 24 Visit will be returned and redispensed at the Week 24 F/U Visit; final return will occur at the Week 28 Visit.

ee. Subject diaries dispensed at the Week 48 Visit will be returned at the Week 48 F/U Visit, and will be redispensed to subjects participating in the Dose-tapering Period for final return at the Week 52 Visit.

ff. All AEs and SAEs must be recorded in the source documents and eCRF from the date of the subject's written informed consent until the final Week 52 Visit or the subject's participation in the study is completed (SAEs through 30 days after final study drug dose). Ongoing AEs will be followed to resolution, stabilization, or until such time the Investigator agrees follow-up is not necessary.

gg. For subjects who do not continue to receive vamorolone through an additional vamorolone study or general access program, site staff will make a phone call to the home 31-35 days after the final dose of study medication in VBP15-004 Dose-tapering Period to confirm the final SAE status of the subject.

hh. Subjects who elect to continue vamorolone therapy by enrolling directly into an additional vamorolone study or general access program may be discharged from the study following completion of all final Week 48 assessments, including the Week 48 Follow-up Visit ACTH Stimulation Test.

ii. Subjects who participate in the Dose-tapering Period may be discharged from the study following completion of all final Dose-Tapering Visit assessments (Week 52) (see protocol Section 6.3.7).

5.2. Inclusion – Exclusion Criteria and General Study Population

Approximately 15 or 30 subjects with confirmed diagnosis of DMD (4-<7 years of age) will be randomized into each of the six groups (120 subjects total) (Table 1). The inclusion and exclusion criteria defined in the protocol apply to all subjects and are not repeated herein the SAP.

5.3. Randomization and Blinding

Following consent and review of study entry criteria to confirm subject eligibility for the study, the subject can be randomized to treatment. Randomization should be performed at least 10 days prior to the baseline visit and will be achieved via the Interactive Voice/Web Response System (IXRS) system with username and password access. Randomization will be stratified by participant's age at study entry (<6 vs. ≥ 6 years). Randomization will be stratified only by age; randomization will not be stratified by investigational site. Randomization will require the site investigator, or designee, to verify that the subject meets the inclusion/exclusion criteria of the study, and to verify that the child has not previously been randomized. Randomization for both Period #1 and Period #2 will be performed prior to study start.

5.4. Analysis Variables

Variables to be analyzed include demographics and baseline characteristics, safety variables, and efficacy variables. Derived variables from study endpoints are described with the sections describing the analyses for these endpoints.

Unless otherwise noted, baseline is defined as the last measurement taken prior to first exposure to study drug, including Day 1 measurements taken pre-dosing.

6. SAMPLE SIZE

This is a randomized, double-blind, parallel group, placebo- and active-controlled study. Study medication is administered daily in this Phase IIb trial. Data for untreated subjects from the Cooperative International Neuromuscular Research Group (CINRG) Duchenne Natural History Study (Bellow et al., 2015; Spurney et al., 2014; McDonald et al., 2013; and Henricson et al., 2013) and data for prednisone treated subjects from the CINRG Prednisone study (Escolar et al., 2011) were used to help estimate sample sizes for this study.

Note that subjects in the prednisone and placebo groups will actually be randomized into two groups each:

- Prednisone 0.75 mg/kg/day → Vamorolone 2.0 mg/kg/day (n=15);
- Prednisone 0.75 mg/kg/day → Vamorolone 6.0 mg/kg/day (n=15);
- Placebo → Vamorolone 2.0 mg/kg/day (n=15); or
- Placebo → Vamorolone 6.0 mg/kg/day (n=15).

These groups will be pooled by initial treatment (prednisone or placebo) for the Treatment Period #1 analyses.

If subjects withdraw early from the study in Period #1, additional subjects may be enrolled to achieve approximately 120 subjects completing the Week 24 Visit assessments. Subjects withdrawing during Period 2 will not be replaced.

<u>Analysis method:</u>
In the 24-week vamorolone dose-ranging study (VBP15-003) (Hoffman et al., 2019), doses of 0.25 and 0.75 mg/kg/day showed little evidence of clinical benefit, whereas doses of 2.0 and 6.0 mg/kg/day both showed clear evidence of clinical benefit. The effect size of clinical efficacy depended on the motor outcomes measure (TTSTAND, TTRW, TTCLIMB, 6MWT and NSAA).

More recently acquired data on 18-month treatment with vamorolone at 2.0 or 6.0 mg/kg/day (data from subjects who were in the 24-week VBP15-003 study and continued into VBP15-LTE, with dose escalations to either 2.0 mg/kg/day or 6.0 mg/kg/day [dose escalation and de-escalation were permitted in VBP15-LTE] and have a 12-month data assessment in VBP15-LTE) have suggested that both the 2.0 and 6.0 mg/kg/day doses show long-term benefit that is comparable to that seen with glucocorticoids (prednisone and deflazacort) (Smith et al., 2020).

In consideration of having the primary efficacy endpoint be the comparison of 6.0 mg/kg/day vamorolone versus placebo in VBP15-004, sample size calculations were performed using

data from vamorolone-treated subjects from the VBP15-002/VBP15-003 24-week treatment period. The analysis compared TTSTAND velocity from the combined 2.0 and 6.0 mg/kg/day groups (drug treated, Group A) to the combined 0.25 and 0.75 mg/kg/day (pseudo-placebo; Group B).

LS means from the MMRM modeling of VBP15-002/VBP15-003 24-week data were used as parameter estimates of the population means in the sample size calculations for the comparison of the Treatment Group (2.0 + 6.0 mg/kg/day) vs. the Pseudo-Placebo Group (0.25 + 0.75 mg/kg/day) on the mean change from baseline at Week 24. For the estimates of standard deviations within each comparison group, simple descriptive statistics from the VBP15-002/VBP15-003 24-week data were used. We then estimated power using two-sided t-tests assuming unequal variance, with alpha =0.05.

The following table shows the resultant estimated power for various sample sizes per comparison group for comparing the Treatment Group (2.0 + 6.0 mg/kg/day) vs. the Pseudo-Placebo Group (0.25 + 0.75 mg/kg/day) on the mean change from baseline at Week 24 for the alternative hypothesis that H1: $\mu1 \neq \mu2$ assuming an alpha level of 0.05 and parameter estimates from the analyses described above for TTSTAND velocity:

Sample Size per Comparison Group	μ1 (Pseudo-Placebo)	μ2 (Treatment Group)	σ1 (Pseudo-Placebo)	σ2 (Treatment Group)	Estimated Power
25	-0.0052	0.0450	0.0628	0.0530	84.89%
28	-0.0052	0.0450	0.0628	0.0530	88.76%
30	-0.0052	0.0450	0.0628	0.0530	90.81%

The sample size of 25 per treatment group for 2.0 mg/kg/day, 6.0 mg/kg/day, prednisone, and placebo will result in a total enrollment of 100 subjects which will provide approximately 85% power at alpha level 0.05 to detect a statistically significant difference between 6.0 mg/kg/day and placebo on TTSTAND velocity at Week 24. Similarly, a total enrollment of 120 subjects (30 per treatment group) will provide approximately 91% power at alpha level 0.05 to detect a statistically significant difference between 6.0 mg/kg/day and placebo on TTSTAND velocity at Week 24.

7. GENERAL CONSIDERATIONS

7.1. Analysis Populations

Three populations will be defined for data analysis: the Safety Population, the modified Intent-to-Treat Population, and the Per Protocol Population.

7.1.1. Safety Population

All subjects who receive at least one dose of study medication will be included in the Safety Population. The Safety Population is the primary analysis population for safety and PD assessments. Results will be presented "as treated."

7.1.2. Modified Intent-to-Treat (mITT) Population

All subjects who receive at least one dose of study medication and have at least one TTSTAND, TTRW, TTCLIMB, 6MWT, NSAA, myometry, or ROM post-baseline efficacy assessment will be included in the mITT Population. The mITT Population is the primary analysis population for clinical efficacy. Subjects who receive at least one dose but never have any of the aforementioned post-baseline efficacy assessments will be excluded. Results will be presented "as randomized."

7.1.3. Per Protocol Population

The Per Protocol (PP) Population will be those subjects in the mITT Population who had no major protocol deviations and will be the secondary analysis population for analysis of the efficacy data. Exclusion of subjects from the PP Population will be made on a subject-by-subject basis prior to database soft lock at the end of the 24-week treatment period.

The patients with major violations impacting the evaluation of the primary endpoint (TTSTAND velocity) will be excluded from the PP Population. The major violations include the following:

- Patient discontinues the study without completing the Week 24 assessment of TTSTAND velocity
- Patient has missing TTSTAND velocity data at Week 24 due to the COVID-19 pandemic or due to other reasons.

7.2. Covariates and Subgroups

7.2.1. Planned Covariates

Covariates will include Baseline response and age group.

7.2.2. Subgroups

Descriptive summary statistics for TTSTAND velocity will be presented by 3 subgroups: baseline age <=5.0 years vs. >5.0 years; baseline age <=6.0 years vs. >6.0 years; and TTSTAND seconds at baseline <=5.0 seconds vs. >5.0 seconds.

7.3. Management of Analysis Data

7.3.1. Data Handling

For the summary of continuous values, and laboratory shift tables and ECG and 2D-echo interpretation and Eye examination, unscheduled tests will be included with the time of the nearest regularly scheduled test. If there is a scheduled test and one or more unscheduled tests assigned to the same time point, the most conservative test (i.e., a test with low or high results or a test with abnormal results) will be used, unless on review, the medical monitor determines that the most conservative test result was spurious. Repeated tests will be included only if they reflect abnormal (low or high) results and the corresponding original results are normal.

All laboratory values and ECG results, for all visits, will be provided in by-subject listings.

7.3.2. Missing Data

Every effort will be made to collect all data. However, despite best efforts, missing or incomplete data may be reported. All missing or partial data will be presented in the subject data listing, as they are recorded on the eCRF.

The COVID-19 pandemic necessitated changes in the protocol to avoid unnecessary potential exposure of subjects to coronavirus and resulted in particular safety and efficacy assessments being made optional. As a result, there are multiple occurrences of missing data. The data that are missing due to COVID-19 will primarily be considered to be missing at random in the respective sensitivity analyses. Subjects that are determined to have an appreciable amount of missing data due to the COVID-19 pandemic may be excluded from the Per Protocol Population. See Section 7.3.2.3 for additional sensitivity analyses that will be performed for the primary efficacy endpoint.

For subjects with missing or incomplete data, all available data will be included in statistical presentations. Unless otherwise specified (e.g., primary endpoint sensitivity analysis), in general no imputation of values for missing data will be performed.

7.3.2.1. Handling of Missing Date Values

Partial or Missing Dates

The following conventions will be used to impute missing portions of dates for adverse events and concomitant medications, if warranted. Note that the imputed values outlined here may not always provide the most conservative date. In those circumstances, the imputed value may be replaced by a date that will lead to a more conservative analysis.

A. Start Dates

1) If the year is unknown, then the date will not be imputed and will be assigned a missing value.

2) If the month is unknown, then:

    i) If the year matches the first dose date year, then impute the month and day of the first dose date.

    ii) Otherwise, assign ‘January.’

3) If the day is unknown, then:

    i) If the month and year match the first dose date month and year, then impute the day of the first dose date.

    ii) Otherwise, assign the first day of the month.

B. Stop Dates

1) If the year is unknown, then the date will not be imputed and will be assigned a missing value.

2) If the month is unknown, then assign ‘December.’

3) If the day is unknown, then assign the last day of the month.

7.3.2.2. Imputation Methods

The primary analyses for the primary and secondary efficacy variables will be conducted on observed data only; the MMRMs will employ appropriate covariance structures to accommodate missing data as described in Section 7.6. Missing efficacy data will be imputed for sensitivity analyses on the primary and secondary efficacy endpoints using the following methods:

- Multiple Imputations using Markov Chain Monte Carlo (MCMC); and
- Multiple Imputations using a Control-based Pattern Mixture Model (PMM).

The pattern and type of missing data will be summarized by visit. For each visit, the data will be classified as available, or missing. The missing data will be further classified as intermittent (missing value is followed by an observed value) or as measurement dropouts (all subsequent values after the missing value are missing). The intermittent missing data will

be further classified as missing due to COVID-19 or due to other reasons. The measurement dropouts will be further classified as inability of the subject to perform or complete the test due to disease-related disability, due to COVID-19, or due to other reasons. Once a subject is no longer able to perform or complete a test due to disability, all subsequent values to the missing values are missing. In conversion to velocity, all missing values due to inability to perform the test are imputed as "0" (this imputation is included in the endpoint analyses on TTSTAND velocity, TTRW velocity, and TTCLIMB velocity, and is carried out prior to the MCMC and PMM imputation for the sensitivity analyses of the primary endpoint TTSTAND and secondary endpoint TTRW velocity). The number and proportion of subjects in each category will be summarized by visit.

MCMC (assuming data are missing at random- primary efficacy endpoint only, using all subjects):

For multiple imputations (MI) using MCMC, 1000 "complete" (imputed) datasets will be produced and analyzed using all subjects (i.e., subjects without post-baseline assessments will be included in this MCMC imputation modeling). The MCMC imputation will be performed within treatment group and include variables for age at baseline, and response at baseline, Week 6, Week 12, and Week 24 (note that age is included as a surrogate for the randomization factor age group because the MCMC imputation in Proc MI assumes a multivariate normal distribution). After obtaining 1000 "complete" datasets of response values, change from baseline will be calculated, and each of these 1000 datasets will be analyzed using the MMRM described in Section 7.6 for the primary efficacy endpoint (i.e., the MMRM will be employed for each imputation separately on change from baseline). PROC MIANALYZE will then be used to combine the results across imputations. The SAS Proc MI code for performing this MCMC imputation is provided here:

```
proc mi data=tadft out=tadftmcmc1 seed=5346434 minimum=0 nimpute=1000;
      by treatment;
      mcmc chain=multiple impute=full;
      var age baseline week_6 week_12 week_24;
run;
***********************************************
MINIMUM=0 sets a lower limit on imputed values.
NIMPUTE specifies the number of fully imputed datasets to create.
BY statement requires imputation to be done within treatment group.
VAR statement lists variables in model, including response at each visit.
```

Note: if any of the treatment levels has no missing data, the data from the treatment level in question will be excluded from the PROC MI procedure above (using WHERE statement). If this is done, the observed data from the treatment level in question (without any missing data) will be added to the output dataset produced above 1000 times, i.e. once for each round of imputation.

Control-based PMM (assuming data are missing not at random- primary and secondary efficacy endpoints using mITT population):

The primary efficacy analysis model (MMRM) makes the assumption that missing data are “missing at random” (MAR). When data are MAR, the missingness of the data does not depend on the missing value after conditioning on the observed data (i.e., prior assessments and baseline covariates). Note that when the missingness of the data depends on the values of the missing variables after conditioning on the observed data, the data are called “missing not at random” (MNAR). In order to assess the MAR assumption, a control-based PMM will be utilized following the method discussed in Ratitch and O’Kelly (2011) for the primary efficacy endpoint. For this study, missing data due to COVID-19 is always assumed to be MAR. For the remaining missing data, assumption of MNAR will be used as shown below, using Copy-Reference imputation.

The following provides an overview, followed by a detailed description, of the imputation and analysis steps.

1. Imputation of all intermittent missing data with MI assuming MAR, in order to generate a dataset with monotone missing data structure.
2. Based on dataset generated in Step 1: imputation of all monotone missing data with MI assuming MAR, in order to generate dataset with no missing data.
3. Based on dataset generated in Step 2 (with no missing data): all visits that have monotone missing data that are not due to COVID-19 will be set back as missing.
4. Based on dataset generated in Step 3: imputation of remaining monotone missing data (not related to COVID-19) with multiple imputation assuming MNAR (Copy-Reference imputation).
5. Based on dataset generated in Step 4: analysis of imputed data with MMRMs.
6. Combination of results from the MMRMs.

Step 1: Imputation of intermittent missing data
MI techniques will be applied in the mITT analysis set. For patients with intermittent missing values, a monotone missingness pattern will be generated. Intermittent missing values will be imputed using the MCMC methodology which assumes a multivariate normal distribution over all variables included in the imputation model. The MI procedure in SAS will be used for this purpose and this first MI step will be repeated 1000 times, generating 1000 different datasets with a monotone missing data structure. A random seed value will be used in the MI procedure and documented in this SAP, to allow replication of the analysis. Seed value of 5346434 will be used. The imputation is based on the MAR assumption, i.e. the missing data are assumed to follow the same model as the other patients in their respective treatment arm. Age is included as a factor in the model because the randomization is stratified by age.

The following SAS code will be used to generate the monotone missing data pattern:

```
proc mi data=&data out=datamono nimpute=1000 seed=5346434 minimum=0;
by trtp;
var age base w6 w12 w24;
```

```
mcmc chain=multiple impute=monotone;
run;
```

Step 2: Imputation of all monotone missing data
Using the dataset generated in Step 1, all remaining missing data will be imputed based on the assumption of MAR. The following SAS code will be used for the imputation assuming MAR.

```
proc mi data=monotone out=imputed1 nimpute=1 seed=5346434 minimum=0;
class trtp;
by _imputation_;
var age base w6 w12 w24;
monotone regression;
run;
```

Step 3: Setting monotone missing data that are not due to COVID-19 back as missing
After Step 2, information on reason for missing data (by patient and visit) will be merged to the dataset that contains no missing data. Within each patient, data from the visits that have monotone missing data with a reason for missing that are not due to COVID-19 will be set back as missing.

The reason for missing data (by patient and visit) will be determined as follows:

- According to the “VBP15-004 eCRF Guidelines Addendum for COVID-19 Protocol Clarification V.1.0”, sites were instructed to report the reason for not performing an assessment due to COVID-19 as “Other” and then provide a COVID-19 related reason as free text. If this free text field includes the string “COVID”, the reason will be classified as being due to COVID-19. If the free text does not include the string “COVID”, the reason will be classified as other. Before conducting this classification, the free text field will be checked for any possible spelling errors.

Step 4: Imputation of remaining (non-COVID-19 related) missing data with assumption of MNAR
The dataset generated in Step 3 (which contains monotone missing data) will be imputed based on the assumption of MNAR, using Copy-Reference imputation. The MNAR imputation will be based on trajectories in the placebo group, i.e. data from the placebo group only will be used to generate the imputations. For this, the MNAR statement will be used as described below.

```
proc mi data=imputed2 out=imputed3 nimpute=1 seed=5346434 minimum=0;
class trtp;
by _imputation_;
var age base w6 w12 w24;
monotone regression;
mnar model(w6 w12 w24 / modelobs=(trtp='Placebo'));
run;
```

Step 5: Analysis of imputed data with MMRM

After the missing data imputation is completed using the above steps, change from baseline values will be calculated in each of the imputed datasets at each visit. These 1000 datasets will be analyzed using the MMRM described in Section 7.6 for the primary efficacy endpoint. Treatment effects (difference in least squares [LS] means between treatments) from these 1000 analyses will then be combined using Rubin's Method via the SAS PROC MIANALYZE procedure for each endpoint.

7.3.2.3. Assessment of impact of COVID-19 on the primary endpoint

The following COVID-19 related potential data issues will be addressed with additional sensitivity analyses:

- Impact of assessments that are missing or delayed due to COVID-19
- Impact of alternative assessment methods (motor assessment using video assessment method).

In both these sensitivity analyses, the data that are missing (or set as missing) due to COVID-19 are assumed to be missing at random (MAR), while the other missing data are assumed to be missing not at random (MNAR) (Meyer et al., 2020).

Sensitivity analysis assessing the impact of assessments that are missing or delayed due to COVID-19

In this sensitivity analysis, the assessments that were early or delayed by more than 21 days from the scheduled visit date due to COVID-19 will be set as missing. Scheduled visit date will be calculated from the first dose date. In case there is no documentation of whether the rescheduling by more than 21 days occurred is due to COVID-19 or due to other reasons, it will be classified as being due to COVID-19. After this step, all missing data will be categorized as being due to COVID-19 (either missing or set as missing due to delay) or being due to other causes. In this sensitivity analysis, the data assessed with alternative assessment methods will be treated as non-missing. The control-based PMM modelling approach defined in Section 7.3.2.2 will be used for this analysis.

Sensitivity analysis assessing the impact of alternative assessment methods

In this sensitivity analysis, the assessments that were performed using alternative methods (i.e., video assessments) due to COVID-19 will be set as missing. After this step, all missing data will be categorized as being missing due to COVID-19 (including alternative assessment methods) or being missing due to other causes (including the remaining missing data for any reason). Note that the data from visits that were delayed due to COVID-19 will be treated as non-missing. The control-based PMM modelling approach defined in Section 7.3.2.2 will be used for this analysis.

7.3.3. Handling of Early Termination Visit Information

In the event that a subject is terminated early from this study, the early termination visit data will be analyzed at the closest scheduled visit where the assessments are to be measured. If the closest scheduled visit has valid data, the early termination data will be assigned to the next available scheduled visit where the assessments are to be measured.

7.3.4. Pooling of Investigational Sites

The data from all study centers will be pooled together for analyses.

7.3.5. Coding Conventions for Events and Medications

All adverse events, and medical history will be mapped to the Medical Dictionary for Regulatory Activities (MedDRA version 20.0 or later) system for reporting (preferred term and body system).

Prior and Concomitant medications will be coded using World Health Organization (WHO) Drug classification (Version 01 JUN 2017 or later).

7.3.6. Baseline Visits

Unless otherwise noted, Baseline will be the last response before first dose of study medication. For example, labs taken over multiple days for the Day 1 visit for a participant where there is not a complete Day 1 pre-dose lab panel completed at a single date will use individual labs taken closest to, but prior to, first dose as baseline.

7.3.7. Calculating Derived Variables

Age in years will be calculated as (informed consent date – birth date)/365.25. In case a patient has multiple informed consent dates, the date closest to and prior to baseline (i.e. the latest date) will be used.

<u>Efficacy Measures</u>

TTSTAND, TTRW, AND TTCLIMB in seconds, 6MWT in meters, NSAA subscores, hand-held myometry, and ROM are captured directly via CRF. However, TTSTAND, TTCLIMB, and TTRW velocity results will be converted from seconds to velocities using the following formulas

- TTSTAND velocity = 1 / TTSTAND and is expressed as rises/sec
- TTCLIMB velocity = 1 / TTCLIMB and is expressed as tasks/sec
- TTRW velocity = 10 / TTRW and is expressed as meters/sec

Velocity will be set to 0 for responses determined to be missing due to disease progression (inability to do the test). Moreover, at the first visit a subject cannot perform the test due to disease progression, and at ALL subsequent visits, the raw score will be left as missing, and velocity will be imputed as 0. Note that a subject cannot have a missing response due to disease progression followed by visits with non-missing responses. In this scenario, the missing response will not be considered missing due to disease progression. Furthermore, the CRF captures missing due to disease progression in some cases explicitly as missing due to “Disease progression”. Also, subjects with TTSTAND graded as “Unable to stand from supine, even with use of chair” or “Assisted Gowers” will have that test and all later test velocities imputed as 0 due to disease progression. Similarly, subjects with TTRW graded as “Unable to walk independently” or “Unable to walk independently but can walk with knee-ankle-foot or support from a person” will have that test and all later test velocities imputed as 0 due to disease progression. And subjects with TTCLIMB graded as “Unable to climb 4 standard stairs” will have that test and all later test velocities imputed as 0 due to disease progression. Note that for the primary and secondary analyses, velocity is imputed as described here only for unmissed subject visits. For example, if a subject discontinued the study at Week 12, and was unable to perform these tests at that visit due to disease progression, this subject would not have Week 24 velocity imputed.

For elbow flexors and knee extensors, hand-held myometry measurements were collected unilaterally using the dominant side, if known. The best of 3 collected test results at each visit will be summarized.

NSAA total score is calculated as the sum of all NSAA subscores only when none of the subscores are missing.

Safety Measures

For BMI Z-scores, the following computational example given age and sex for children aged 2 to 20 years uses the algorithm presented on the Centers for Disease Control and Prevention (CDC) webpage “Percentile Data Files with LMS Values”. For a detailed discussion on the derivation of the computational algorithm and reference materials, visit the webpage at https://www.cdc.gov/growthcharts/percentile_data_files.htm. To obtain the Z-score (Z) for a given BMI measurement X, use the following equation:

Z= [((X/M)^L) – 1] / (LS), where L≠0

or

Z = ln(X/M)/S, where L=0

where L, M, and S are the values from the BMIAGE.xls reference table (growth chart 8 linked to on the aforementioned CDC webpage).

For example, for a 24-month-old male (coded sex value = 1) who has a BMI of 17.2864, the BMIAGE.xls reference table presents values of L=-2.01118, M=16.57503, and S=0.080592. Plugging those parameter values into the Z formula above results in a Z-score of 0.5.

For height percentiles, the same computational formula for Z provided above in the BMI Z-score example is used. However the reference table, given age and sex for children aged 2 to 20 years, is the STATAGE.xls reference table (Stature-for-age charts, 2 to 20 years, LMS parameters and selected smoothed stature percentiles in centimeters, by sex and age at webpage "Percentile Data Files with LMS Values" https://www.cdc.gov/growthcharts/percentile_data_files.htm). Once the Z-score is calculated, the percentile is then derived from the standard normal cumulative distribution.

For DXA scan data, 9 endpoints will be presented. Three of the 9 endpoints are derived endpoints, which use the following formulas:

- Total body composition fat-free mass (g) = total body bone mineral content (BMC) (g) + total body lean mass (g)
- Total body composition lean mass index (kg/m^2) = (total body BMC + total body lean mass) / $height^2$ (note that total body lean mass and total body BMC must be converted to kg, and height must be converted to m for this formula)
- Total body composition fat mass index (kg/m^2) = total body fat mass / $height^2$ (note that total body fat mass must be converted to kg, and height must be converted to m for this formula)

Exploratory Measures

The TSQM Version II has 11 items, resulting in four specific domains of Effectiveness, Side Effects, Convenience, and Global Satisfaction. Scores for each domain are computed by adding the TSQM items in each domain and then transforming the composite score into a value ranging from 0 to 100. Of note, a score can be computed for a domain only if no more than one item is missing from that domain. The calculations specific to each domain are presented in detail below.

- Global Satisfaction

  ([Sum(Item 10 to Item 11) – 2] divided by 12) * 100

  If one item is missing

  ([(Use the completed item)) – 1] divide by 6) * 100

- Effectiveness
  ([(Item 1 + Item 2) – 2] divide by 12) * 100
  If one item is missing
  ([(Use the completed item)) – 1] divide by 6) * 100

- Side Effects
  All 'NA' responses are coded as '5' indicating 'Not at all Dissatisfied'
  ([Sum(Item 4 to Item 6) – 3] divide by 12) * 100
  If one item is missing

([(Sum(the two completed items)) – 2] divide by 8) * 100

- Convenience
  ([Sum(Item 7 to Item 9) – 3] divided by 18) * 100
  If one item is missing
  ([(Sum(the two completed items)) – 2] divided by 12) * 100

The PARS III (Stein R et al., 1990) is a brief parent-completed measure of psychosocial adjustment composed of 28 questions. The PARS III includes 6 psychosocial subscales (Peer Relations, Dependency, Hostility, Productivity, Anxiety/Depression, and Withdrawal) and a Total Score. The 4 PARS III subscales for Peer Relations, Dependency, Anxiety and Depression, and Withdrawal will be calculated and analyzed in this SAP using the sum of the score of the questions for each subscale. All 28 questions use a 4-point interval rating scale, ranging from 1 = "never or rarely" to 4 = "always". For calculating subscales, some questions are recoded so that higher scores always indicate better adjustment. Hostility and Productivity are not measured as they have not shown changes in DMD populations, and Total Score will not be analyzed. If a 4-item subscale is missing more than one item or a 6-item subscale is missing more than two items, it will not be scored. If less than one or two items, respectively, is missing, the subscale will be scored by replacing the missing item with the subject's mean of the non-missing items for that subscale.

The questions summed for each subscale presented in this SAP are as follows:
Peer Relations

- Spent time with friends?
- Made friends without difficulty?
- Joined others of own accord?
- Had many different friends?

Dependency

- Wanted help in things he could have done on own?
- Been unable to decide things for self?
- Asked for help when could have figured things out?
- Asked unnecessary questions instead of working on own?

Anxiety and Depression

- Complained about problems?
- Seemed restless, tense?
- Said people didn't care about him?
- Seemed sad?
- Said he couldn't do things right?
- Acted afraid or apprehensive?

Withdrawal

- Sat and stared without doing anything?
- Appeared listless and apathetic?
- Seemed unaware of things going on around?
- Shown little interest in things, had to be pushed into activity?

For PODCI subscale Upper Extremity and Physical Function, the following 8 questions will be summed to provide a raw score where a minimum of 4 question responses must be non-missing and valid:

- Lift heavy books?
- Pour a half gallon of milk?
- Open a jar that has been opened before?
- Use a fork and spoon?
- Comb his hair?
- Button buttons?
- Write with a pencil?
- Turn doorknobs?

The standardized Upper Extremity and Physical Function score is calculated as [(4 - mean of non-missing questions used to calculate the raw score) / 3] * 100. Note that response scores of 5 indicate that the subject was too young for the activity and are set to missing by Data Management when calculating the subscale score (see POSNA (PODCI) Child Proxy Scoring Outcomes Instrument).

For PODCI subscale Transfers and Basic Mobility, the following 11 questions will be summed to provide a raw score where a minimum of 7 question responses must be non-missing and valid:

- Put on his coat?
- Climb one flight of stairs?
- Walk one block?
- Get on and off a bus?
- Stand while washing his hands and face at a sink?
- Sit in a regular chair without holding on?
- Get on and off a toilet or chair?
- Get in and out of bed?
- Bend over from a standing position and pick up something off the floor?
- How often does your child need help from another person for sitting and standing?
- How often does your child use assistive devices (such as braces, crutches, or wheelchair) for sitting and standing?

The standardized Transfers and Basic Mobility score is calculated as [(4 - mean of non-missing questions used to calculate the raw score) / 3] * 100. Note that the responses to the last 2 questions are rescaled if used in calculating the sum and standardize score as follows: rescaled = [(response - 1) * 3/4] + 1. Note that response scores of 5 indicate that the subject

was too young for the activity and are set to missing by Data Management when calculating the subscale score (see POSNA (PODCI) Child Proxy Scoring Outcomes Instrument).

7.3.8. Analysis Software

Data manipulation, tabulation of descriptive statistics, and graphical representations will be performed primarily using SAS (release 9.4 or higher) for Windows. If the use of other software is warranted, the final clinical study report will detail what software was used and for what purposes.

7.3.9. Study Data

Study data identified in the schedule for time and events (Table 2 Schedule of Study Activities) are collected, and source verified, on the electronic data capture tool: OpenClinica. Laboratory data, including PK and PD test results, are not collected in the EDC tool and are provided from external laboratories.

All study data will be formulated into regulatory compliant data sets to provide transparency, traceability, and integrity of trial analysis results from the collection source. Observed study data will be mapped to the CDISC Study Data Tabulation Model (SDTM) and serve as the source data from the trial (note that any hardcoding of data will be captured in notes-to-file requiring Sponsor approval and detailed in the relevant reviewer guide). All study analyses will be completed using analysis data sets that are derived from the SDTM and follow the CDISC Analysis Data Model (ADaM) architecture.

The methods for programming the CDISC SDTM and ADaM data sets in this SAP are described in Figure 1. Note that the methods described here for Period #1 may need to be modified in order to incorporate the 2 periods of the study in the whole Study SAP.

Figure 1 **SDTM, ADaM, and TFL Development and Validation**

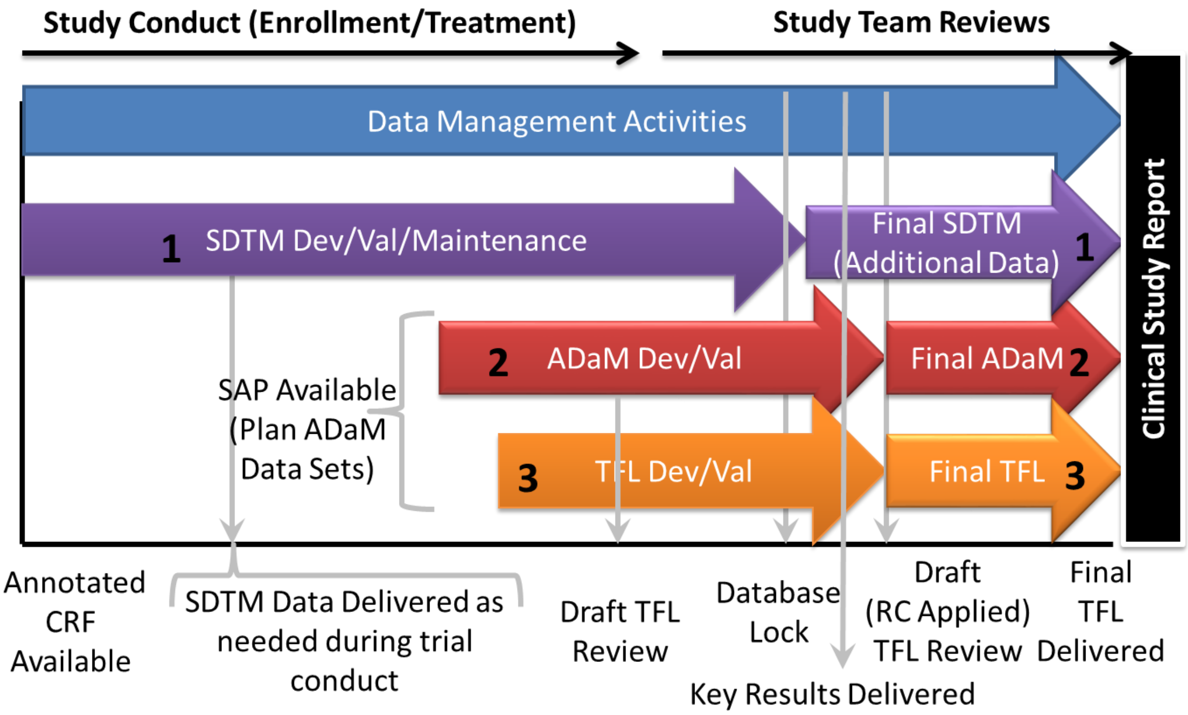

Where:

1. Development, Validation, and Maintenance of SDTM domains
2. Development and Validation of Analysis Data Sets (ADaM), with input source the appropriate SDTM domains.
3. Development and Validation of draft and then final Tables, Figures, and Listings (TFL), with input data source the SDTM domains and analysis specific ADaM data sets and randomization code (RC) applied.

7.3.10. Period #1 Data Management Study Data Cutoff

As detailed in the Data Management Plan v2.0, raw datasets will be separated by Data Management into data captured on CRFs up to Week 24, which will include Week 24 F/U data, and data captured on CRFs after Week 24. Only the “up to Week 24” datasets will be transferred to Summit Analytical for analysis in this SAP.

Data from “up to Week 24 visit” events will be selected from *non-visit* eCRFs (informed consent, adverse events, concomitant medications, and concomitant non-drug treatment form) using the study event variable, including only visits labeled “up to Week 24”. The Unscheduled Visit and End of Study event (Discharge from Study CRF) data will be separated using the event date of the Unscheduled Visit and the Last Date of Study

Participation, respectively, and the first date of dose tapering from the Study Drug Return eCRF at the Week 28 visit for each participant during the transition period to Treatment Period #2. For participants lost to follow up, screen failures, and participants lost to follow up during Treatment Period #1, all data is included. The other event data will be separated using the study event variable, including only visits up to and including Week 24.

7.4. General Statistical Methodology

7.4.1. Statistical Summaries: Descriptive and Inferential

Unless otherwise specified, all statistical tests will be two-sided and a resultant p-value of less than or equal to 0.05 will be considered statistically significant. See Section 7.4.5 for more details on the handling of multiple comparisons for the efficacy analyses. All p-values will be rounded to and displayed in four decimals. If a p-value less than 0.0001 occurs, it will be shown in tables as <0.0001.

Descriptive summaries of variables will be provided where appropriate. In general, for continuous variables, the number of non-missing values (n) and the mean, standard deviation, median, minimum, and maximum will be tabulated. For categorical variables, the counts and proportions of each value will be tabulated.

All collected data will be presented in listings. Data not subject to analysis according to this plan will not appear in any tables or graphs but will be included in the data listings.

7.4.2. Interim Analyses and Data Monitoring

No interim analyses are planned for this study.

The independent Data and Safety Monitoring Board (DSMB) will be consulted to review safety data. The DSMB will meet at regular intervals to review all pertinent safety data. The DSMB may request summaries at other points in time. In addition, the Medical Monitor may request at any time that the DSMB review safety data if the Medical Monitor has specific concerns.

In all cases, data will be compiled by the Coordinating Center and presented to the DSMB in a format that allows for complete review of all compiled safety data. The DSMB can recommend to the Sponsor altering or terminating the trial for safety or other study integrity-related issues.

The primary safety endpoints that the DSMB will review are safety labs and adverse events. Refer to the DSMB charter for complete details. Analysis and reporting of safety endpoint information is specified in the DSMB Charter, and not repeated herein. Note that all DSMB reports will be included in the final CSR.

7.4.3. Week 24 Analysis

The primary analyses described in this SAP are the analyses which will be performed after all subjects complete the Week 24 F/U Visit (or complete the Week 24 Visit for subjects who were unable to complete the Week 24 F/U Visit) (end of Treatment Period #1). The results from these analyses will be provided to regulatory authorities. Investigators, study subjects, study staff, and monitors will remain blinded throughout the duration of the 52-week study. The Sponsor, including data management and statistical personnel, will be unblinded after all subjects have completed the Week 24 F/U Visit to provide analysis of Period #1.

7.4.4. Week 48 Analysis

The Week 48 analyses will be performed after all subjects complete Week 48 of Treatment Period #2 and the subsequent 4-week double-blind Dose-tapering Period, if applicable, and will be the subject of a second SAP. The results from these analyses will be provided to regulatory authorities. All study staff may be unblinded after database lock of data from the entire study.

7.4.5. Multiple Testing Procedures

The primary efficacy endpoint tests vamorolone 6.0 mg/kg/day dose group vs. placebo using TTSTAND velocity at Week 24 and occurs during Treatment Period #1 of the study. The primary efficacy endpoint supports the primary objective of this study. The study is thus powered for the efficacy comparison as described in Section 6 using an alpha level of 0.05 for success.

The secondary efficacy endpoints will be tested at Week 24 along with the primary efficacy endpoint using a fixed sequential testing process where the fixed sequence of testing will be done in the following order:

1. TTSTAND velocity, vamorolone 2.0 mg/kg/day vs placebo
2. 6 Minute Walk Test (6MWT) meters walked, vamorolone 6.0 mg/kg/day vs. placebo
3. 6 Minute Walk Test (6MWT) meters walked, vamorolone 2.0 mg/kg/day vs. placebo
4. Time to Run/Walk (TTRW) 10 meters velocity, vamorolone 6.0 mg/kg/day vs. placebo
5. Time to Run/Walk (TTRW) 10 meters velocity, vamorolone 2.0 mg/kg/day vs. placebo
6. 6MWT meters walked, vamorolone 6.0 mg/kg/day vs. prednisone
7. 6MWT meters walked, vamorolone 2.0 mg/kg/day vs. prednisone

Each test in the sequence will be carried out using a two-sided alpha level of 0.05. Statistical testing of the primary/secondary efficacy endpoints will stop if a p-value >0.05 occurs or if a p-value ≤0.05 occurs in the wrong direction. In case the fixed sequential testing process stops, the results of the subsequent tests will be reported with nominal p-values, but p-values

≤0.05 in the right direction will not be considered proof of statistical testing success in these subsequent tests.

All other analyses will not be corrected for multiple comparisons (tests will be performed at a nominal alpha level of 0.05), as they will be viewed and handled in the perspective of not testing a formal hypothesis.

7.5. Summary of Study Data

7.5.1. Subject Summary Grouping

In general, and unless otherwise noted, data summaries will be presented by treatment group.

7.5.2. Study Disposition

Subject counts will be summarized by analysis population.

The number of subjects enrolled (i.e., signed informed consent form), screened following enrollment, randomized following screening, completing Week 24 and Week 24 F/U, discontinuing early, and the reason for discontinuation from the study will be summarized.

The number of subjects randomized by country and site will be tabulated in a summary table by analysis population.

A by-subject data listing of study completion information including the reason for premature study withdrawal, if applicable, will be presented. In addition, the reasons for failing screening will be listed. Also, the duration of treatment (time from first dose to last dose during Period #1) will be presented in the listing.

7.5.3. Protocol Deviations

All protocol deviations will be presented in a by-subject data listing. Deviations will be presented by subject and site. Deviations incurred due to COVID-19 pandemic study modifications will be noted. Important deviations will be classified.

Protocol deviations will be summarized descriptively, categorically, by treatment group for all analysis populations.

7.5.4. Demographics and Baseline Characteristics

Subject demographics (age, race, and ethnicity), baseline characteristics (height, height percentile, weight, body mass index [BMI], BMI Z-score, and months since first DMD symptoms noticed), and baseline efficacy data for TTSTAND in seconds, 6MWT, and NSAA total score will be summarized descriptively, either continuously or categorically by treatment group for all analysis populations.

Subjects that indicate more than 1 race will be counted under “Multiple” in the race tabulation.

All demographic and baseline information, including DMD mutation type, will be presented in by-subject listings (note that date of birth will be excluded from the listings).

7.5.5. Medical History

Subject medical and surgical history will be collected during the screening period and reviewed throughout the study. The dates and descriptions of past events will be documented in source documents and captured in the relevant eCRF. Medical history will be coded using MedDRA (version 20.0 or later). The number and percentage of subjects with any medical history events will be summarized by system organ class and preferred term by treatment group (and overall) for the Safety Population. At each level of tabulation subjects will be counted once if they had 1 or more of each such event.

Subject medical history data will be presented in a by-subject listing.

7.5.6. Prior and Concomitant Medications

A categorical summary of prior and all concomitant medications and non-pharmacological treatments taken prior to and during the course of the study will be presented in tabular form summarizing the number and percentage of subjects by anatomical therapeutic chemical classification level and preferred name by treatment group (and overall) using the World Health Organization (WHO) Drug classification (Version 01 JUN 2017 or later) for the Safety Population. At each level of tabulation subjects will be counted once if they had 1 or more of instance of medication usage.

A concomitant medication is defined as any medication taken on or after the day of first exposure to study drug up until the Week 52 Visit. Concomitant medications taken on or after the first exposure to study drug until the end of the Week 24 F/U Visit will be summarized in the Period #1 analyses.

Prior medications are defined as any medications that are taken prior to the day of first exposure to study drug, collected from up to 3 months prior to Screening.

Medications missing year from date information will be considered concomitant medications unless other date information shows clearly that the medication was not concomitant.

All prior medications, concomitant medications, and non-pharmacological treatments will be presented in by-subject listings.

7.5.7. Treatment Compliance and Study Drug Exposure

Study drug administration and treatment compliance, and study drug exposure will be summarized descriptively for suspension and tablets separately for the Safety Population and the mITT Population.

Suspension administration and compliance will be calculated based on the weight of suspension in grams. For each subject, the total suspension administered will be estimated from the total weight of the suspension bottles dispensed and returned (amount dispensed g – amount returned g), as collected on the CRF. Further, the amount of suspension prescribed is captured on the CRF in mL. This data will be used to calculate the total suspension prescribed for each subject and converted to grams (1 mL suspension = 1 g suspension). Compliance percentage will then be calculated as the total suspension administered g / total suspension prescribed g x 100%. These endpoints will be summarized for all treatment groups and overall.

Tablet administration and compliance will calculated be based on the count of tablets dispensed and returned, and the count of tablets prescribed. The count of tablets dispensed and returned is captured on the CRF and will be used to calculate the total tablets administered for each subject. Further, for each subject, the prescribed tablet dosing is captured on the CRF in mg, which will be converted to a count of tablets (1 tablet = 5 mg). Compliance percentage will then be calculated as the total tablets administered count / total tablets prescribed count x 100%. These endpoints will be summarized for all treatment groups and overall.

For vamorolone, exposure will be presented only for the vamorolone treatment groups. For the 6.0 mg/kg/day treatment group, vamorolone exposure will be calculated as the total suspension administered g x 40 mg/g (there are 40 mg vamorolone per g of 6 mg suspension). Similarly for the 2.0 mg/kg/day treatment group, vamorolone exposure will be calculated as the total suspension administered g x 13.3 mg/g (there are 13.3 mg vamorolone per g of 2 mg suspension).

For prednisone, exposure will be presented only for the prednisone treatment group. Prednisone exposure will be calculated as the total tablets administered x 5 mg.

Treatment administration, compliance, and exposure data will be presented in by-subject listings.

7.6. Efficacy Analyses

The evaluations of clinical efficacy will be performed using the mITT Population and Per Protocol Population, unless otherwise noted. Analyses will be done as per randomized treatment. Only data captured during Treatment Period #1 (Screening to Week 24) will be analyzed in this SAP.

All efficacy data will be summarized descriptively for observed and change from baseline by treatment group and visit, and will be presented in by-subject listings. Where considered relevant, plots will be created.

The primary efficacy outcome, TTSTAND (velocity) change from baseline to Week 24, will be compared between the 6.0 mg/kg/day vamorolone group and the placebo group using a restricted maximum likelihood (REML)-based MMRM. This model includes fixed effects for treatment (vamorolone 2.0 mg/kg/day, vamorolone 6.0 mg/kg/day, prednisone 0.75 mg/kg/day, and placebo), age group (<6 years; ≥6 years), week, baseline TTSTAND velocity, and the treatment-by-week interaction. Study week will be included in the model as a categorical variable (Weeks 6, 12, and 24) along with the treatment-by-week interaction. Within this model, pairwise comparisons (using LS mean contrasts) will be made to compare TTSTAND velocity at 24 weeks for vamorolone 6.0 mg/kg/day dose level with placebo (primary efficacy outcome).

For secondary and exploratory efficacy outcomes, as detailed in Sections 4.2.3.2 and 4.2.3.3, the same models will be used as for primary outcome (REML-based MMRM including fixed effects for treatment [vamorolone 2.0 mg/kg/day, vamorolone 6.0 mg/kg/day, prednisone 0.75 mg/kg/day, and placebo], age group [<6 years; ≥6 years], week, response variable baseline, and the treatment-by-week interaction).

Additional tests are done in exploratory analyses. See Section 7.4.5 for more details on the handling of multiple comparisons of secondary analyses.

An unstructured covariance matrix will be used, and underlying modelling assumptions will be checked. If this analysis fails to converge, Akaike's information criterion will be used to select the best covariance structure from compound symmetry and autoregressive-1 (AR(1)). The Kenward-Roger approximation will be used to estimate denominator degrees of freedom. If differences between baseline characteristics exist between the treatment groups, it will be investigated if adjustment for these characteristics is clinically relevant and necessary. The secondary outcome measures will be compared using similar models.

The following sensitivity analyses will be performed using the MMRM analysis described above for the primary outcome (TTSTAND velocity vamorolone 6.0 mg/kg/day dose group versus placebo and presented for Week 24), and the secondary outcomes, presented for Week 24, (TTSTAND velocity vamorolone 2.0 mg/kg/day vs placebo; 6MWT meters walked vamorolone 6.0 mg/kg/day vs. placebo; 6MWT meters walked vamorolone 2.0 mg/kg/day vs. placebo; TTRW 10 meters velocity vamorolone 6.0 mg/kg/day vs. placebo; TTRW 10 meters velocity vamorolone 2.0 mg/kg/day vs. placebo; 6MWT meters walked vamorolone 6.0 mg/kg/day vs. prednisone; and 6MWT meters walked vamorolone 2.0 mg/kg/day vs. prednisone) (see Section 7.3.2.2 for more details):

- Multiple imputation using MCMC methods for missing data on TTSTAND velocity and 6MWT, using the mITT population;
- Multiple imputation using MCMC methods for missing data on TTSTAND velocity and 6MWT, using all subjects, including those with no baseline or post-baseline assessment;
- Multiple imputation using MCMC methods for missing data on TTSTAND velocity and 6MWT, using randomized subjects who have non-missing baseline data;
- No imputation on TTSTAND velocity and 6MWT, using randomized subjects with no missing assessments;
- Multiple imputation using a Control-based Pattern-Mixture Model for missing data on the primary and secondary efficacy endpoints, using the mITT population.

The following COVID-19 sensitivity analyses will be performed using the MMRM analysis described above for the primary outcome (TTSTAND velocity vamorolone 6.0 mg/kg/day dose group versus placebo and presented for Week 24 using the mITT population) (see Section 7.3.2.3 for more details):

- Impact of assessments that are missing or delayed due to COVID-19
- Impact of alternative assessment methods (motor assessment using video assessment method)

The following sensitivity analysis assessing the impact of the most influential TTSTAND observations will be performed using the MMRM analysis described above for the primary outcome TTSTAND velocity vamorolone 6.0 mg/kg/day dose group versus placebo and presented for Week 24 using the mITT population: All TTSTAND values that are <2.5 seconds (0.4 rises per second) will be imputed as 2.5 seconds. After this imputation, the data will be analyzed similarly as for the primary analysis. The rationale of this sensitivity analysis is provided below.

- The time to rise testing protocol caps the time allowed to rise at 30 seconds (0.033 rises per second). The velocity transformation diminishes the impact of large fluctuations in timed performance at the more impaired end of the spectrum. When a patient cannot perform the test anymore, a standardized value is assigned (velocity=0). In this case, e.g. a 45 second actual performance is imputed as unable to perform the test (velocity=0).
- A challenge of the velocity transformation in the less impaired spectrum of DMD is that variability in reaction time of the subject or the variability in initiating the stopwatch by the clinical evaluator (typically about 0.5 seconds) can impact the velocity substantially even though a small change in time to rise at the less impaired end of the scale is not clinically meaningful in terms of disease progression or improvement. For example, a deterioration from 2.0 seconds to 2.5 seconds in rise time would translate to a deterioration of 0.1 rises per second in velocity which is twice the pre-specified effect size of this study.
- The lower limit of normal for time to rise from the floor for 4 to 6 year old or older healthy typically developing patients (defined as 5th percentile or better) from a natural history cohort from Belgium (Hoskens, et al. 2019) was found to be 2.5

seconds. Therefore, incremental small changes in rise times below 2.5 seconds would be deemed to be not clinically meaningful and are observed in virtually all healthy typically developing children with normal phenotype. There is likelihood that the signal to noise ratio below 2.5 seconds would be suboptimal as large changes in velocity would be calculated with small changes in absolute times.

- Therefore, to mitigate the effect of reaction time on the calculated velocity at the less impaired end of the scale and to diminish the likelihood that a 0.2 to 0.5 second change in rise from floor time occurring in the normal range of functioning would result in a large change in velocity, a sensitivity analysis will be performed that transforms all values below 2.5 seconds to a value of 2.5 seconds. For example, a 0.5 second change in the normal end of the spectrum from 2.5 to 2.0 seconds or from 2 to 2.5 seconds, which would result in a calculated change in velocity of 0.1 rises per second will be treated as a change of 0 (2.5 seconds on both measures), for the purposes of this sensitivity analysis.

The following sensitivity analysis due to missing 6MWT data, which pools other motor function tests, will be presented:

- As reported by McDonald et al. (2013), the motor function tests TTSTAND, TTRW, TTCLIMB, and 6MWT are correlated in patients with DMD as they all measure timed motor function. In order to evaluate the impact of vamorolone treatment on timed motor function using as complete dataset as possible, an additional sensitivity analysis will be conducted by pooling the data from all the motor function tests except TTSTAND (i.e., TTRW velocity, TTCLIMB velocity, and 6MWT). The rationale for this sensitivity analysis is to use as much data as possible to estimate the impact of missing data. Due to the correlation both between the parameters and within the repeated longitudinal assessments, this analysis is expected to provide further information on the consistency of the effects. The concept of pooling data from multiple motor function endpoints in DMD has been introduced by Li et al. (2020). The analysis defined below uses similar concept of pooling data from the timed tests used in DMD, using these data in a sensitivity analysis based on a multivariate repeated measures model.
    - First, the test data will be standardized by using the percentage change from baseline as the endpoint. The percentage change will be calculated as (value at visit – baseline value) / baseline value x 100%, calculated from the endpoints as used in the original statistical analysis, i.e. from velocities or distances. All the percentual change values from the three tests, TTRW, TTCLIMB, and 6MWT, will be entered to a single statistical model.
    - A model similar to the MMRM model presented in this SAP will be used, but in addition to the fixed factors included in the MMRM detailed above, the parameter (TTRW velocity, TTCLIMB velocity, or 6MWT) and all 2- and 3-level interaction terms between treatment group, visit and parameter will be added. The model will use the Kronecker product covariance structure, as proposed by Galecki (1994). Accordingly, the covariance structure will be set

as type=un@ar(1) in the REPEATED statement of the MIXED procedure. The SAS code for implementing this kind of model with the MIXED procedure is provided by Gao et al. (2006) and outlined below.

```
proc mixed;
class usubjid age paramcd trtp avisit;
model chg=age paramcd trtp avisit paramcd*trtp paramcd*avisit trtp*avisit
      paramcd*trtp*avisit / ddfm=kr;
repeated paramcd avisit / subject=usubjid type=un@ar(1);
run;
```

As support for the primary and secondary efficacy endpoint analyses, the pattern and type of missing data will be summarized for the primary and secondary efficacy endpoints by visit. For each visit, the data will be classified as available, or missing. The missing data will be further classified as intermittent (missing value is followed by an observed value) or as measurement dropouts (all subsequent values after the missing value are missing). The intermittent missing data will be further classified as missing due to COVID-19 or due to other reasons. The measurement dropouts will be further classified as inability of the subject to perform the test due to disease-related disability, due to COVID-19, or due to other reasons.

Furthermore, a supportive responder analysis of the TTSTAND velocity primary and secondary efficacy endpoints (comparison of vamorolone 6.0 mg/kg/day dose group versus the placebo group and 2.0 mg/kg/day dose group versus the placebo group at Week 24 assessment) will be conducted by classifying the endpoint data as improvements (better velocity compared to baseline) versus non-improvements (same or worse velocity compared to baseline). The responder endpoints will be tabulated at Week 24 by treatment group, and the percentage of responders will be compared between vamorolone 6.0 mg/kg/day dose group versus placebo group and 2.0 mg/kg/day dose group versus placebo group using descriptive statistics and Fisher's exact test. This analysis will be performed on the mITT population. Further, this analysis will be done 2 ways, first on observed cases only, and then again assuming subjects with a missing Week 24 assessment will be considered non-improvers.

In addition to the summary tables described above, TTSTAND velocity at Week 24 will be presented in 3 summary tables of continuous descriptive statistics on observed response and change from baseline by treatment group and time point using the mITT population by 3 subgroups: baseline age <=5.0 years vs. >5.0 years; baseline age <=6.0 years vs. >6.0 years; and TTSTAND seconds at baseline <=5.0 seconds vs. >5.0 seconds.

TTSTAND in seconds will be presented in a summary table of continuous descriptive statistics on observed response and change from baseline by treatment group and time point using the mITT population.

TTSTAND in seconds will be presented in a shift table for Week 24 shift from baseline for categories of <5 seconds, 5 to 10 seconds, and >10 seconds. Subject counts and percentages will be tabulated by treatment group for the mITT population. Percentages will be based on the number of subjects with a baseline and Week 24 assessment.

Additional supportive analyses for the primary efficacy endpoint may be performed. They include excluding subjects with large differences between the screening and baseline value TTSTAND velocity scores (larger than 2 standard deviations) and excluding subjects who had their TTSTAND times measured at a visit using live video remote streaming, instead of recorded and times measured from the live recording.

7.7. Safety Analyses

Safety analyses will be performed using the Safety Population and will be completed using the actual treatment a subject received. All safety data will be presented in by-subject listings as well as in tables and figures as described below. In general, descriptive statistics for each safety endpoint will be presented by time point and treatment group. Where considered relevant, plots will be created.

Safety data from Treatment Period #1 will be summarized together by treatment group.

7.7.1. Adverse Events

Adverse events will be coded using the Medical Dictionary for Regulatory Activities (MedDRA), version 20.0.

TEAEs are defined as any adverse event or worsening of an existing condition after initiation of the investigational product and through the subject’s last study visit (study completion or early termination). AEs with missing year from the start date will be considered treatment-emergent unless it is clear from non-missing date information that the AE started prior to first dose of study drug. For the Period #1 analyses described herein, AEs and TEAEs which occur through the subject’s Week 24 F/U Visit are included. Serious AEs will be recorded from the date of informed consent, throughout the clinical trial, and for up to 30 days after the final administration of study drug, with the cut-off for the Period #1 analyses at the Week 24 F/U Visit (or Week 24 Visit for subjects who did not have a Week 24 F/U Visit). If the onset of an AE is on Day 1 and its relationship to time of study drug administration is unknown, then the AE will be counted as treatment-emergent. If the onset of the AE is on Day 1 but is known to have onset prior to the time of the first administration of study drug, the AE will not be considered treatment emergent.

The number and percent of subjects with any TEAEs will be summarized by system organ class and preferred term by treatment group (and overall). At each level of tabulation (e.g., at the preferred term level) subjects will be counted only once if they had more than one such event reported during the AE collection period. (Note that the ADaM ADAE dataset will

include the coded terms for SOC, PT, Highest Level Term, Highest Level Group Term, and Lowest Level Term.)

Level of intensity will be assessed using the CTCAE grading (CTCAE v 4.03 grade).

The following summary tables and subject level listings will be presented for TEAE data:

- Overall summary of TEAEs
- Summary table of incidence of TEAEs by descending incidence by PT
- Summary table of incidence of TEAEs by SOC and PT (will include counts of subjects and events)
- Summary table of incidence of serious TEAEs by SOC and PT (will include counts of subjects and events)
- Summary table of incidence of TEAEs by maximum relatedness to treatment by SOC and PT
- Summary table of incidence of TEAEs by maximum intensity by SOC and PT
- Summary table of incidence of TEAEs leading to study drug discontinuation by SOC and PT
- Summary table of incidence of TEAEs by worst outcome (recovered/resolved vs. recovering/resolving vs. not recovered/not resolved vs. recovered/resolved with sequelae vs. fatal vs. unknown) by SOC and PT
- Table listing of SAEs
- Table listing of related SAEs
- Table listing of all AEs leading to death
- Table listing of all AEs leading to study discontinuation

A listing will present all AEs associated with suicidality (Maund et al., 2014), where adverse event listing preferred term and verbatim term data will be searched and reported for terms:

- “suic”, “overdos, “attempt”, “cut”, “gas”, “hang”, “hung”, “jump”, “mutilate”, “overdos”, “self damage”, “self harm”, “self inflict”, “self injur”, “shoot”, and “slash”
- “poi”, “emot”, “labi”, “hos”, “vio”, “agg”, “thought”, and “think”.

A listing will present all AEs associated with abuse potential (Bossard et al., 2016), where adverse event listing preferred term and verbatim term data will be searched and reported for terms:

- “drug”, “chemical”, “abuse”, “dependence”, “disuse”, “diversion”, “withdrawal”, “mood”, “substance”, “unapproved”, “polysubstance”, “euphoric”, “addict”, “dysthymic”

Obvious false positives (e.g., “gas” in “gastrointestinal, “cut” in “acute,” and “vio” in “behavior”) will be excluded as per FDA (Posner et al., 2007).

7.7.2. Vital Signs, 12-Lead ECG, and Laboratory Outcomes

Vital signs, including height and height percentile, weight, and BMI and BMI Z-score, clinical laboratory test results, and other laboratory test results not detailed elsewhere in this SAP will be summarized at each assessment time point by treatment group using descriptive statistics and presented for observed response as well as change from baseline. Descriptive statistics will include the typical statistics for continuous endpoints described in this SAP as well as interquartile range.

Height percentile change from baseline and BMI Z-score change from baseline results will be compared between treatment groups using an REML-based MMRM analysis with treatment group, week of the visit, and the treatment-by-week interaction as factors, and baseline response (height percentile or BMI Z-score) and age group as a covariate. Week will be included in the model as a categorical variable (Week 12 and 24) along with the treatment-by-week interaction. An unstructured within-subject covariance matrix will be used. If this analysis fails to converge, Akaike's information criterion will be used to select the best covariance structure from compound symmetry and autoregressive-1 (AR(1)). The Kenward-Roger approximation will be used to estimate denominator degrees of freedom.

Overall ECG interpretation will be summarized categorically by assessment response and via shift table presenting Normal/Abnormal Not Clinically Significant/Abnormal Clinically Significant and will also be presented in by-subject listings.

Clinical laboratory summary tables for continuous descriptive statistics will include only central lab data. Change from baseline in all continuous clinical laboratory test results will be tested using one sample t-tests with p-values presented along with the descriptive statistics at each assessment visit within each treatment group (note that pharmacodynamic biomarkers- creatine kinase [CK] for efficacy, fasting glucose and insulin for insulin resistance, and glutamate dehydrogenase [GLDH] for safety- will be of special interest).
Clinical laboratory test results will also be presented in shift tables for all laboratory parameters where low/normal/high or normal/abnormal status can be ascertained. These shift tables will include central and local lab data. Abnormal clinical lab test results will be presented in a table listing where low/normal/high or abnormal/normal status can be ascertained.

Low/normal/high or normal/abnormal flags will be imputed on local lab data only if the normal flag is missing and a lower and/or upper normal range are provided in the raw data for the observation so that the lab response can be assessed as low/normal/high or normal/abnormal depending on the normal range data provided. Note that this same imputation will also be carried out on vitamin D data from the central lab when possible.

For by-subject listings, clinically significant response (yes/no) will be presented as collected for local labs.

Lab results that are below the lower limit of quantification will be divided by 2 for analysis (ex., a lab response of "<0.1" will be analyzed as 0.05). Lab results that are above the upper level of quantification will be analyzed using the upper level of quantification (ex., a lab response of ">1500" will be analyzed as 1500).

Central lab results will be presented using both U.S. conventional units and SI units. Local lab results will be presented using the units as collected.

Except for lab results and follow-up eye and ECG examinations, data gathered at unscheduled visits will not be summarized but will be included in by-subject data listings. See Section 7.3.1 for unscheduled or repeated lab tests.

7.7.3. Physical Examination

Physical examination results will be presented in by-subject listings.

7.7.4. Other Safety Measures

Cushingoid features will be summarized by presenting the number and percentage of subjects by treatment group that develop them as adverse events.

2D-echocardiogram results will be summarized by treatment group at Baseline and Week 24. The number and percent of subjects who have normal, abnormal not clinically significant, or abnormal clinically significant findings will be summarized by treatment group using shift tables.

Eye examination results will be summarized at Baseline and Week 24. The number and percent of subjects who have a cataract present (yes/no) will be summarized by treatment group for left eye and right eye separately. The same summary will be performed for the presence of glaucoma (yes/no), though not broken out by left and right eye. Intraocular pressure readings will be presented in by-subject listings. Note that the window for the Week 24 eye exam data to be included in summary tables is the Week 24 scheduled visit date +-6 weeks.

ACTH Stimulation Test results will be summarized by treatment group at Baseline and Week 24 Follow-up. The peak cortisol measurement from the 30-minute and 60-minute tests will be used in the analysis. The number and percent of subjects who have peak cortisol levels <18 µg/dL (<500 nM) or ≥ 18 µg/dL (≥ 500 nM) will be summarized by treatment group. Pearson Chi-square or Fisher's exact tests will be used to compare each vamorolone dose with prednisone, and the two vamorolone doses with each other. Note that the analysis windows for the 30-minute and 60-minute tests are 30 minutes +-15 minutes and 60 minutes +-15 minutes, respectively. Assessments outside these windows will not be included in the analyses.

DXA scan data will be summarized by treatment group at Baseline and Week 24. Descriptive continuous statistics will be presented for observed response, change from baseline, and percent change from baseline. Further, each vamorolone dose group will be compared with prednisone on the percent change from baseline utilizing LS means from an ANCOVA model with treatment group (all treatment groups included) as a main effect and age group at study entry and baseline result as covariates.

Fracture questionnaire results will be presented in by-subject listings.

7.8. Exploratory Analyses

Patient Reported Outcomes including the TSQM, PARS III, and Ease of Study Medication Administration Assessment will be listed and presented using descriptive statistics by treatment and time point using the Safety Population. PODCI data will be presented similarly using the mITT Population. The Blindedness Assessment will be listed and presented using descriptive statistics by treatment at Week 24 using the Safety Population.

Each vamorolone dose group will be compared with prednisone on TSQM response at Week 24 utilizing LS means from an ANCOVA model with treatment group (all treatment groups included) as a main effect and age group at study entry as a covariate.

Each vamorolone dose group will be compared with prednisone and placebo on the change from baseline in PARS III utilizing a REML-based MMRM with treatment group (all treatment groups included), week of the visit, and the treatment-by-week interaction as factors, and age group at study entry and baseline result as covariates for PARS III subscores for peer relations, dependency, anxiety and depression, and withdrawal.

Physical functioning will be assessed by completion of the PODCI. Two subscales (Upper Extremity and Physical Function, and Transfers and Basic Mobility) will be summarized descriptively as a continuous endpoint by treatment group at each time point collected. Observed scores and change from baseline will be presented. Standardized scores will be used for the analyses. All PODCI data will be presented in a by-subject listing. Each vamorolone dose group will be compared with placebo on the change from baseline utilizing an ANCOVA model with treatment group (all treatment groups included) as a main effect and age group at study entry and baseline result as covariates.

7.9. Pharmacodynamic (PD) Serum and Other Biomarkers

The evaluations of PD will be performed using the Safety Population. Analyses will be done as per actually received treatment.

Serum PD biomarkers of adrenal axis suppression, insulin resistance, and bone turnover will be assessed at Baseline, Week 12, and Week 24. Additional exploratory PD biomarkers of

both safety and efficacy may be assessed. Vamorolone-treated groups will be compared to both prednisone-treated and placebo groups.

The descriptive summaries will include continuous descriptive statistics on observed and change from baseline at each week by treatment group. Continuous descriptive statistics will be provided along with interquartile range. One sample t-tests will be provided to test if the change from baseline mean values are different from zero within each treatment group.

PD biomarker change from baseline results will be compared between treatment groups using REML-based MMRM analyses with treatment group, week of the visit, and the treatment-by-week interaction as factors, and age group at study entry and baseline result as a covariate. Week will be included in the model as a categorical variable (Week 12 and 24) along with the treatment-by-week interaction. An unstructured within-subject covariance matrix will be used. If this analysis fails to converge, Akaike’s information criterion will be used to select the best covariance structure from compound symmetry and autoregressive-1 (AR(1)). The Kenward-Roger approximation will be used to estimate denominator degrees of freedom. (Note that HbA1c is collected only once following baseline. It will be analyzed using an ANCOVA model in a similar manner as described here for the MMRM analyses. The ANCOVA model will include treatment group as a main effect, and age group at study entry and baseline result as covariates.)

For first-in-morning serum cortisol levels, along with the statistics described above, the number and percent of subjects who have cortisol levels <3.6 µg/dL (<100 nM) or ≥ 3.6 µg/dL (≥ 100 nM) will be summarized by treatment group. Pearson Chi-square or Fisher’s exact tests will be used to compare each vamorolone dose with prednisone, and the two vamorolone doses with each other, similarly to how ACTH simulated cortisol levels are to be tested.

All PD biomarker data will be presented in by-subject listings. The listings will include normal range data, where available. Furthermore, a listing of out of range observations will be presented.

8. REPORTING CONVENTIONS

The following reporting conventions will be adopted for the presentation of study data. These conventions will enhance the review process and help to standardize presentation with common notations

8.1. General Reporting Conventions

- All tables and data listings will be developed in Landscape Orientation, unless presented as part of the text in a CSR.
- Figures will be presented in Landscape Orientation, unless presented as part of the text in a CSR.
- Legends will be used for all figures with more than one variable or item displayed.
- Figures will be in presented in color with treatment groups distinguished by different symbols and colors. Lines in figures should be wide enough to view the line after being photocopied.
- Specialized text styles, such as bolding, italics, borders, shading, superscripted and subscripted text will not be used in tables, figures, and data listings unless they add significant value to the table, figure, or data listing.
- Only standard keyboard characters should be used in tables and data listings. Special characters, such as non-printable control characters, printer specific, or font specific characters, will not be used on a table, figure, or data listing. Hexadecimal character representations are allowed (e.g.,μ,α,β).
- All titles will be centered on a page. The ICH numbering convention is to be used for all tables, figures, and data listings.
- All footnotes will be left justified and the bottom of a page. Footnotes must be present on the page where they are first referenced. Footnotes should be used sparingly and must add value to the table, figure, or data listing. If more than four footnote lines are planned, then a cover page may be used to display footnotes.
- Missing values for both numeric and character variables will be presented as blanks in a table or data listing. A zero (0) may be used if appropriate to identify when the frequency of a variable is not observed.
- All date values will be presented as YYYY-MM-DD (e.g., 2013-05-17) ISO 8601 format.
- All observed time values will be presented using a 24-hour clock HH:MM:SS format (e.g., 01:35:45 or 11:26). Seconds should only be reported if they were

measured as part of the study, also in ISO 8601 format.

- Time durations will be reported in mixed HHh MMm SSs notation (e.g., 5h 32m, or 27h 52m 31s). The use of decimal notation to present (display) time durations should be avoided (e.g. 0.083h = 5m) unless it is necessary to show the computation of time differences in a table, figure, or data listing, in which case both notations may be used to display the time duration.
- All tables, figures, and data listings will have the Table, Listing, or Graph status (DRAFT, FINAL), and a date/time stamp on the bottom of each output.
- All analysis programs developed for a table, figure, or data listing display will be self-contained to facilitate transfer of programs to multiple computing environments and transfer to a regulatory agency (if requested).

8.2. Population Summary Conventions

- Population(s) represented on the tables or data listings will be clearly identified in the last title of the Table as “Population: <name of population>” and will be identical in name to that identified in the protocol or SAP.
- Consistent terminology will be used to define and identify a population.
- Sub-population(s) or special population(s) descriptions will provide sufficient detail to ensure comprehension of the population (e.g., FAS Females, Per-Protocol Males >60 years of age) used for analysis in a table or figure.
- Population sizes may be presented for each treatment or dosing category as totals in the column header as (N=xxxx), where appropriate.
- Population sizes shown with summary statistics are the samples sizes (n) of subjects with non-missing values.
- All population summaries for categorical variables will include all categories that were planned and for which the subjects may have had a response. Percentages corresponding to null categories (cells) will be suppressed.
- All population summaries for continuous variables will include: N, mean, SD, median, minimum, and maximum. Other summaries (e.g. number missing, quartiles, 5%, 95% intervals, coefficient of variation [CV] or %CV) may be used as appropriate.
- All percentages are rounded and reported to xx.x%. A percentage of 100% will be reported as 100%. For categorical summaries presenting “n (%)”, a count of 0 will be presented as “0”. For continuous results an estimated % of 0 will be presented as “0%”.
- Population summaries that include p-values will report the p-value to four decimal places with a leading zero (0.0001). All p-values reported on default output from statistical software (ie., SAS® Software version 9.4 or

later) may be reported at the default level of precision. P-values <0.0001 should be reported as <0.0001 not 0.0000.

9. REFERENCES

Bello L, Kesari A, Gordish-Dressman H, et al. Genetic modifiers of ambulation in the Cooperative International Neuromuscular Research Group Duchenne natural history study. Ann Neurol. 2015; 77(4):684-696.

Bossard JB, Ponté C, Dupouy J, Lapeyre-Mestre M, Jouanjus E. Disproportionality Analysis for the Assessment of Abuse and Dependence Potential of Pregabalin in the French Pharmacovigilance Database. Clin Drug Investig. 2016;36(9):735 - 742.

Escolar DM, Hache LP, Clemens PR, et al. Randomized, blinded trial of weekend vs. daily prednisone in Duchenne muscular dystrophy. Neurology. 2011; 77(5):444-452.

Galecki AT. General class of covariance structures for two or more repeated factors in longitudinal data analysis. Communications in Statistics - Theory and Methods, 23:11, 3105-3119 (1994).

Henricson EK, Abresch RT, Cnaan A, et al. The Cooperative International Neuromuscular Research Group Duchenne natural history study: glucocorticoid treatment preserves clinically meaningful function milestones and reduces rate of disease progression as measured by manual muscle testing and other commonly used clinical trial outcome measures. Muscle Nerve. 2013; 48(1):55-67.

ICH E9 Expert Working Group. Statistical Principles for Clinical Trials: ICH Harmonized Tripartite Guideline, September 1998.

Li D, McDonald CM, Elfring GL, Souza M, McIntosh J, Kim DH, Wei LJ. Assessment of Treatment Effect With Multiple Outcomes in 2 Clinical Trials of Patients With Duchenne Muscular Dystrophy. JAMA Netw Open. 2020 Feb 5;3(2).

Maund E, Tendal B, Hróbjartsson A, Lundh A, Gøtzsche PC. Coding of adverse events of suicidality in clinical study reports of duloxetine for the treatment of major depressive disorder: descriptive study. BMJ. 2014;348:g3555.

McDonald CM, Henricson EK, Abresch RT, Duong T, Joyce NC, Hu F, Clemens PR, Hoffman EP, Cnaan A, Gordish-Dressman H; CINRG Investigators. Long-term effects of glucocorticoids on function, quality of life, and survival in patients with Duchenne muscular dystrophy: a prospective cohort study. Lancet. 2018 Feb 3;391(10119):451-461. doi: 10.1016/S0140-6736(17)32160-8.

McDonald CM, Henricson EK, Abresch RT, et al. The Cooperative International Neuromuscular Research Group Duchenne natural history study – a longitudinal

investigation in the era of glucocorticoid therapy: design of protocol and the methods used. Muscle Nerve. 2013; 48(1):32-54.

Meyer RD, Ratitch B, Wolbers M, Marchenko O, Quan H, Li D, Fletcher C, Li X, Wright D, Shentu Y, Englert S, Shen W, Dey J, Liu T, Zhou M, Bohidar N, Zhao PL & Hale M. Statistical Issues and Recommendations for Clinical Trials Conducted During the COVID-19 Pandemic, Statistics in Biopharmaceutical Research, 2020; 12 (4): 399-411. DOI: 10.1080/19466315.2020.1779122.

Posner K, Oquendo MA, Gould M, Stanley B, Davies M. Columbia Classification Algorithm of Suicide Assessment (C-CASA): classification of suicidal events in the FDA's pediatric suicidal risk analysis of antidepressants. Am J Psychiatry. 2007 Jul;164(7):1035-43. doi: 10.1176/ajp.2007.164.7.1035. PMID: 17606655; PMCID: PMC3804920.

Ratitch B, O’Kelly M. Imputation of Pattern-Mixture Models Using Standard SAS/STAT Procedures, PharmaSUG 2011 - Paper SP04.

Smith EC, Conklin LS, Hoffman EP, Clemens PR, Mah JK, Finkel RS, Guglieri M, Tulinius M, Nevo Y, Ryan MM, Webster R, Castro D, Kuntz NL, Kerchner L, Morgenroth LP, Arrieta A, Shimony M, Jaros M, Shale P, Gordish-Dressman H, Hagerty L, Dang UJ, Damsker JM, Schwartz BD, Mengle-Gaw LJ, McDonald CM; CINRG VBP15 and DNHS Investigators. Efficacy and safety of vamorolone in Duchenne muscular dystrophy: An 18-month interim analysis of a non-randomized open-label extension study. PLoS Med. 2020 Sep 21;17(9):e1003222. doi:10.1371/journal.pmed.1003222. PMID: 32956407; PMCID: PMC7505441.

Spurney C, Shimizu R, Morgenroth LP, et al. Cooperative International Neuromuscular Research Group Duchenne Natural History Study demonstrates insufficient diagnosis and treatment of cardiomyopathy in Duchenne muscular dystrophy. Muscle Nerve. 2014; Aug; 50(2):250-256.

Stein R, Jessop D. Manual for Personal Adjustment and Role Skills Scale, Albert Einstein College of Medicine of Yeshiva University. 1990.

10. APPENDICES

10.1. List of Tables, Listings, and Figures

A table of contents (TOC) of all SAP tables, listings, and figures will be provided in an external programming shell document.

10.2. SAP Amendment Summary of Important Changes

SAP Version	Section	Description of Change
Final v2.0	7.7.1 Adverse Events	The following clarification was added to the description of listings for AEs associated with suicidality and abuse potential:<br><br>Obvious false positives (e.g., “gas” in “gastrointestinal, “cut” in “acute,” and “vio” in “behavior”) will be excluded as per FDA (Posner et al., 2007).
Final v3.0	3.1 Preface	Clarified language to indicate a separate analysis plan **will** be developed to accommodate recommendations specified by EMA.<br><br>Updated language to indicate that for publication **and other commercial purposes** of the Period #1 VBP15-004 study data, this FDA-specific SAP will be applied.
Final v3.0	3.3 Summary of Statistical Analysis Changes to the Protocol	Clarified that spine x-ray analysis is discussed in the protocol. However, it will be presented in a separate SAP and is not included in this SAP.<br><br>Noted that analysis of DNA testing (Week 24) is mentioned as an additional exploratory endpoint in the protocol but will not be analyzed in this SAP.<br><br>Noted that exploratory biomarkers mentioned in the protocol will be stored and studied later and are not included in this SAP.
Final v3.0	4.2.1 Safety Endpoints	Clarified language to indicate that the peak cortisol level after ATCH stimulation will be used to assess adrenal suppression.<br><br>Clarified language by adding BMI (kg/m^2) as a safety endpoint to the list of safety endpoints.
Final	4.2.5	Clarified language to indicate first-in-morning serum

SAP Version	Section	Description of Change
v3.0	Pharmacodynamic Endpoints	cortisol levels can include fasting and non-fasting data.<br><br>Clarified language to indicate that the peak cortisol level 30 or 60 minutes after ATCH stimulation will be used to assess adrenal suppression.
Final v3.0	7.1.2 Modified Intent-to-Treat (mITT) Population	Clarified language defining the mITT population.
Final v3.0	7.1.3 Per Protocol Population	Clarified language defining the Per Protocol population.
Final v3.0	7.2.2 Subgroups	Added subgroup analyses to the TTSTAND velocity summary statististics.
Final v3.0	7.3.1 Data Handling	Added 2D-echo interpretation and eye examination data to rules on handling unscheduled data.
Final v3.0	7.3.2 Missing Data	Added clarification regarding classification of data missing due to COVID-19.
Final v3.0	7.3.2.1 Handling of Missing Date Values	Removed imputation rules for missing AE times because time is not collected for AEs.
Final v3.0	7.3.2.2 Imputation Methods	Added details to describe the MCMC and PMM multiple imputation analyses.
Final v3.0	7.3.2.3 Assessment of impact of COVID-19 on the primary endpoint	Added details to describe the multiple imputation analyses.
Final v3.0	7.3.7 Calculating Derived Variables	Clarified the calculation of age for the study.<br><br>Added language to clarify that the best of 3 collected results for elbow flexors and knee extensors will be analyzed.<br><br>Added details for BMI Z-score and height percentile calculation.<br><br>Added details for DXA endpoint calculations.

SAP Version	Section	Description of Change
		Added details for PARS III and PODCI questionnaires.
Final v3.0	7.3.10 Period #1 Data Management Study Data Cutoff	Added language detailing the data cutoff performed by Data Management.
Final v3.0	7.5.2 Study Disposition	Additional details added to clarify tabulations.
Final v3.0	7.5.4 Demographics and Baseline Characteristics	Additional details added to clarify tabulations.
Final v3.0	7.5.5 Medical History	Additional details added to clarify tabulations.
Final v3.0	7.5.6 Prior and Concomitant Medications	Additional details added to clarify tabulations.
Final v3.0	7.5.7 Treatment Compliance and Study Drug Exposure	Added description of administration, exposure, and compliance calculations.
Final v3.0	7.6 Efficacy Analyses	Added details on numerous sensitivity analyses for handling missing responses.<br><br>Added clarification on the TTSTAND velocity responder analysis.<br><br>Added detail on TTSTAND velocity subgroup summary tables.<br><br>Add summary tables for TTSTAND seconds.
Final v3.0	7.7.1 Adverse Events	Clarified that AEs with missing year from the start date will be considered treatment-emergent unless it is clear from non-missing date information that the AE started prior to first dose of study drug.
Final v3.0	7.7.2 Vital Signs, 12-Lead ECG, and Laboratory	Added language to clarify that height percentile will be analyzed using MMRM similar to BMI Z-score.<br><br>Clarified that only central lab data will be included in

SAP Version	Section	Description of Change
	Outcomes	summary tables of continuous descriptive statistics.<br><br>Clarified that laboratory shift tables will include central and local lab data.<br><br>Added language to clarify imputation of low/normal/high range flags and normal/abnormal range flags.<br><br>Clarified how lab results that are below or above limit of quantification will be handled.
Final v3.0	7.7.4 Other Safety Measures	Added detail for ACTH stimulation test windowing.<br><br>Added language to describe how DXA scan data will be analyzed.
Final v3.0	7.8 Exploratory Analyses	Added language to clarify the statistical analyses being performed to compare vamorolone treatment groups to prednisone and placebo on TSQM, PARS III, and PODCI.<br><br>Added clarification on the presentation of blindedness assessment data.
Final v3.0	7.9 Pharmacodynamic (PD) Serum and Other Biomarkers	Added details on HbA1c analysis.<br><br>Added language to clarify the statistical analysis for assessing adrenal suppression being performed on first-in-morning cortisol.
Final v3.0	9 References	Additional references added.
