# Supplementary material for: Efficacy and Safety of Vamorolone vs Placebo and Prednisone Among Boys With Duchenne Muscular Dystrophy: A Randomized Clinical Trial
Source: JAMA Neurol. 2022 Aug 29;79(10):1005–14. doi: 10.1001/jamaneurol.2022.2480 (PMC9425287; doi:10.1001/jamaneurol.2022.2480)
Supplement: Supplement 8. — eTable 1. Study Populations eTable 2. Parent-Reported PARS III Outcome of Anxiety and Depression eFigure. Post hoc Analysis of Percentage Change in Motor End Points at 24 Weeks vs Baseline eTable 3. Treatment-Emergent Adverse Events (TEAEs) eTable 4. Laboratory Measures of Pharmacodynamic Safety Biomarkers eTable 5. Central Laboratory “LOW” Calls for Cortisol Measures in Placebo Group and at Screening in Drug-Treated Groups eTable 6. Percentage of Participants With Peak ACTH-Stimulated Cortisol Measures <500 nmol/L at Both 30 Minutes and 60 Minutes [file jamaneurol-e222480-s008.pdf]

Supplementary Online Content

Guglieri M, Clemens PR, Perlman SJ, et al. Efficacy and safety of vamorolone vs placebo and prednisone among boys with Duchenne muscular dystrophy: a randomized clinical trial. *JAMA Neurol*. Published online August 29, 2022. doi:10.1001/jamaneurol.2022.2480

eTable 1. Study Populations

eTable 2. Parent-Reported PARS III Outcome of Anxiety and Depression

eFigure. Post hoc Analysis of Percentage Change in Motor End Points at 24 Weeks vs Baseline

eTable 3. Treatment-Emergent Adverse Events (TEAEs)

eTable 4. Laboratory Measures of Pharmacodynamic Safety Biomarkers

eTable 5. Central Laboratory “LOW” Calls for Cortisol Measures in Placebo Group and at Screening in Drug-Treated Groups

eTable 6. Percentage of Participants With Peak ACTH-Stimulated Cortisol Measures <500 nmol/L at Both 30 Minutes and 60 Minutes

This supplementary material has been provided by the authors to give readers additional information about their work.

© 2022 Guglieri M et al. *JAMA Neurology.*

eTable 1. Study Populations

			Treatments		
	Placebo n (%)	Prednisone 0.75 mg/kg/day n (%)	Vamorolone 2 mg/kg/day n (%)	Vamorolone 6 mg/kg/day n (%)	Total n (%)
Study Populations					
Safety Population [1]	29 (96.7)	31 (100)	30 (100)	28 (93.3)	118 (97.5)
mITT Population [1]	28 (93.3)	31 (100)	30 (100)	28 (93.3)	117 (96.7)
PP Population [1]	28 (93.3)	30 (96.8)	28 (93.3)	27 (90.0)	113 (93.4)

eTable 2. Parent-Reported PARS III Outcome of Anxiety and Depression

Visit	Comparison	n	LSM (SE)	LSM Difference (SE)	LSM Difference 95% CI	p-Value
**Week 12 Change from Baseline**	Vamorolone 2 mg/kg/day vs. Placebo	23 vs. 25	0.68 (0.44) vs. 0.03 (0.42)	0.65 (0.59)	-0.53, 1.83	>.05
	Vamorolone 6 mg/kg/day vs. Placebo	24 vs. 25	-0.81 (0.43) vs. 0.03 (0.42)	-0.84 (0.59)	-2.00, 0.33	>.05
	Vamorolone 2 mg/kg/day vs. Prednisone	23 vs. 29	0.68 (0.44) vs. -1.06 (0.41)	1.74 (0.59)	0.57, 2.92	.004
	Vamorolone 6 mg/kg/day vs. Prednisone	24 vs. 29	-0.81 (0.43) vs. -1.06 (0.41)	0.25 (0.60)	-0.93, 1.43	>.05
**Week 24 Change from Baseline**	Vamorolone 2 mg/kg/day vs. Placebo	26 vs. 26	0.79 (0.39) vs. 0.27 (0.40)	0.52 (0.55)	-0.57, 1.61	>.05
	Vamorolone 6 mg/kg/day vs. Placebo	28 vs. 26	-1.32 (0.39) vs. 0.27 (0.40)	-1.59 (0.53)	-2.65, -0.53	.004
	Vamorolone 2 mg/kg/day vs. Prednisone	26 vs. 29	0.79 (0.39) vs. -0.49 (0.39)	1.28 (0.54)	0.21, 2.35	.02
	Vamorolone 6 mg/kg/day vs. Prednisone	28 vs. 29	-1.32 (0.39) vs. -0.49 (0.39)	-0.83 (0.54)	-1.90, 0.25	>.05

© 2022 Guglieri M et al. *JAMA Neurology.*

eFigure. Post hoc Analysis of Percentage Change in Motor End Points at 24 Weeks vs Baseline

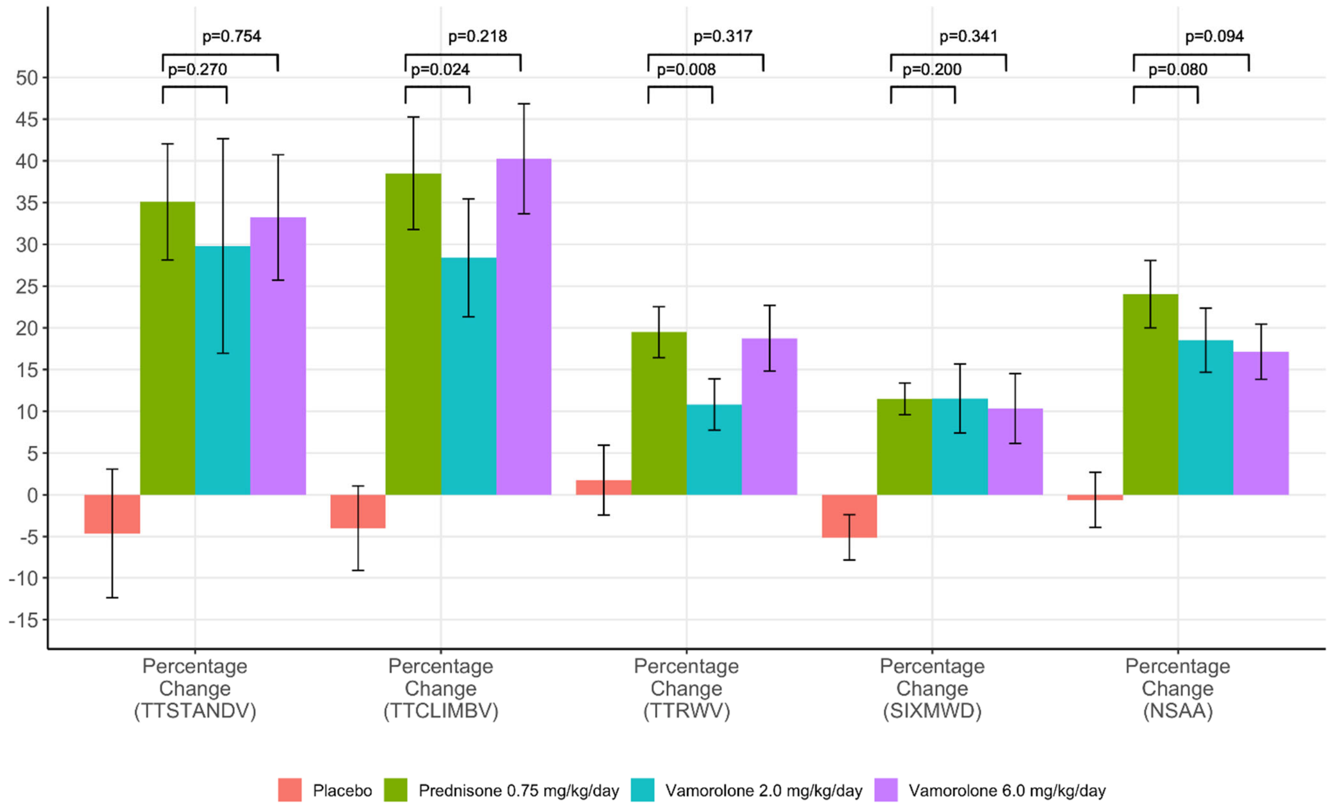

The unadjusted mean relative percentage change at 24 weeks is plotted for time to stand from supine velocity, time to climb 4 steps velocity, time to run/walk 10m velocity, six-minute walk distance, and NorthStar Ambulatory Assessment. The p-values are from a restricted maximum likelihood (REML)-based MMRM of percentage change over time run with treatment (vamorolone 2 mg/kg/day,vamorolone 6 mg/kg/day, prednisone 0.75 mg/kg/day, and placebo), enrollment stratification age group (4-5 years; 6-<7 years), week (as a categorical variable), baseline response, and the treatment-by-week interaction. The Kenward-Roger approximation was used to estimate denominator degrees of freedom as recommended for SAS Proc Mixed when

© 2022 Guglieri M et al. *JAMA Neurology.*

used for repeated measures (MMRM) (SAS for Mixed Models, second edition, by Littell, Milliken, Stroup, Wolfinger, and Schabenberger 2006; SBN 978-1-59047-500-3). Error bars are ±SE.

eTable 3. Treatment-Emergent Adverse Events (TEAEs)

Event type	Placebo (N=29) n (%); F	Prednisone 0.75 mg/kg/day (N=31) n (%); F	Vamorolone 2 mg/kg/day (N=30) n (%); F	Vamorolone 6 mg/kg/day (N=28) n (%); F
**All TEAEs**	23 (79.3); 77	26 (83.9); 121	25 (83.3); 97	25 (89.3); 91
**Severe TEAEs (CTCAE Grade ≥3)**	---	1 (3.2); 1	---	---
**Deaths**	---	---	---	---
**Serious adverse events**	---	---	1 (3.3); 1	---
**TEAEs leading to discontinuation**	---	1 (3.2); 1	---	---

eTable 4. Laboratory Measures of Pharmacodynamic Safety Biomarkers

		Placebo Group	Prednisone Group	Vamorolone 2 mg/kg/day Group	Vamorolone 6 mg/kg/day Group
Osteocalcin ng/mL (SD)	Baseline	55 (14)	56 (13)	57 (18)	60 (15)
	12-weeks	53 (14)	43 (9)***	59 (16)	58 (12)
	24-weeks	50 (17)	41 (9)***	63 (15)	60 (13)
P1NP ng/mL (SD)	Baseline	483 (161)	480 (116)	521 (204)	490 (145)
	12-weeks	475 (139)	315 (68)***	478 (137)	424 (92)***
	24-weeks	452 (139)	335 (85)***	512 (133)	493 (146)
CTX pg/mL (SD)	Baseline	1079 (258)	1125 (162)	1127 (382)	1074 (206)
	12-weeks	1092 (220)	859 (177)***	1192 (284)	1116 (202)
	24-weeks	1066 (266)	805 (187)***	1234 (299)	1176 (224)
Cortisol Morning nmol/L (SD)	Baseline	199 (62)	212 (66)	238 (83)	235 (67)
	12-weeks	221 (95)	85 (56)***	141 (46)***	43 (36)***
	24-weeks	218 (84)	76 (43)***	128 (56)***	36 (41)***
Standard Dose ACTH Stimulation Test nmol/L (SD)	Baseline<br>30 min<br>60 min	 550 (104)<br>628 (112)	 532 (101)<br>612 (97)	 553 (86)<br>648 (94)	 547 (119)<br>659 (105)
	24-weeks<br>30 min<br>60 min	 500 (76)<br>584 (103)	 250 (78)<br>274 (83)	 332 (102)<br>393 (119)	 158 (147)<br>181 (170)

Mean (SD). ***p<0.001 Within-group paired T test vs. baseline.

© 2022 Guglieri M et al. *JAMA Neurology.*

eTable 5. Central Laboratory “LOW” Calls for Cortisol Measures in Placebo Group and at Screening in Drug-Treated Groups

Morning cortisol and ACTH challenge tests were not done on the same days.

		**ACTH challenge**		
	**Morning cortisol** (<138 nmol/L) % (n: low/total)	Basal (<138 nmol/L) % (n: low/total)	30 minute (<497 nmol/L) % (n: low/total)	60 minute (<497 nmol/L) % (n: low/total)
**Placebo group[1]**	11% (9/179)	25% (12/46)	33% (16/49)	15% (8/52)
**Drug-treated at Screening**	8% (7/88)	14% (12/88)	25% (22/88)	8% (7/87)

[1]Data from both Screening and Week 24 in placebo group.

eTable 6. Percentage of Participants With Peak ACTH-Stimulated Cortisol Measures <500 nmol/L at Both 30 Minutes and 60 Minutes

	Placebo Group n (%)	Prednisone Group n (%)	Vamorolone 2 mg/kg/day Group n (%)	Vamorolone 6 mg/kg/day Group n (%)
Baseline	3 (11%)	5 (17%)	2 (7%)	0 (0%)
24-weeks	4 (20%)	26 (100%)	18 (86%)	20 (95%)

© 2022 Guglieri M et al. *JAMA Neurology.*
