# Supplementary material for: Efficacy and Safety of Vamorolone vs Placebo and Prednisone Among Boys With Duchenne Muscular Dystrophy: A Randomized Clinical Trial
Source: JAMA Neurol. 2022 Aug 29;79(10):1005–14. doi: 10.1001/jamaneurol.2022.2480 (PMC9425287; doi:10.1001/jamaneurol.2022.2480)
Supplement: Supplement 9. — Data Sharing Statement [file jamaneurol-e222480-s009.pdf]

# Data Sharing Statement

Guglieri. Efficacy and Safety of Vamorolone vs Placebo and Prednisone Among Boys With Duchenne Muscular Dystrophy. *JAMA Neurol*. Published August 29, 2022. doi:10.1001/jamaneurol.2022.2480

**Data**
**Data available:** Yes
**Data types:** Deidentified participant data, Data dictionary
**How to access data:** https://cinrgresearch.org/publications/data-summary-requests/
**When available:** With publication

**Supporting Documents**
**Document types:** None

**Additional Information**
**Who can access the data:** Anyone requesting the data approved by the CINRG publications committee
**Types of analyses:** For any purpose
**Mechanisms of data availability:** After approval of a proposal, with a signed data access agreement
